# Grignard Reagent Addition to Pyridinium Salts: A Catalytic Approach to Chiral 1,4-Dihydropyridines

**DOI:** 10.1021/acscatal.4c03520

**Published:** 2024-08-15

**Authors:** Siriphong Somprasong, Marta Castiñeira Reis, Syuzanna R. Harutyunyan

**Affiliations:** † Stratingh Institute for Chemistry, University of Groningen, Nijenborgh 4, Groningen 9747 AG, The Netherlands; ‡ Centro Singular de Investigación en Química Biolóxica e Materiais Moleculares (CIQUS), Universidade de Santiago de Compostela, C/ Jenaro de la Fuente s/n, Campus Vida, Santiago de Compostela 15782, Spain

**Keywords:** dihydropyridines, pyridines, dearomatization, copper, chiral heterocycle

## Abstract

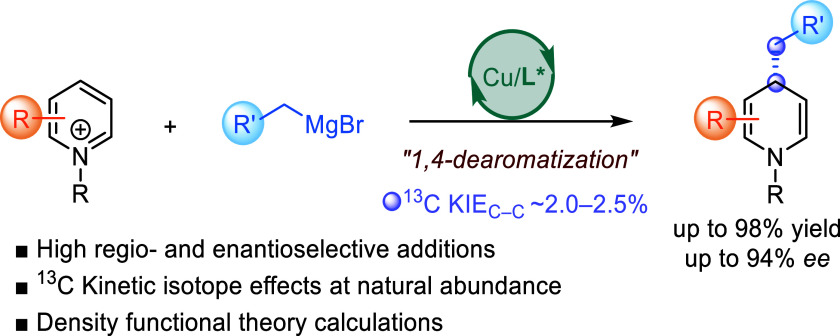

Catalytic dearomatization of pyridinium salts is a powerful technique for constructing chiral *N*-heterocycles, which are crucial in alkaloid natural products and drugs. Despite its potential, progress in metal-catalyzed asymmetric dearomatization of pyridinium derivatives has been limited. Here, we present the enantioselective 1,4-dearomatization of pyridinium salts using Grignard reagents and chiral copper catalysis. This approach yields enantioenriched functionalized 1,4-dihydropyridines. Experimental kinetic isotope effects and density functional theory calculations provide insights into the reaction mechanism, regio- and enantioselectivity, and the rate-limiting step.

## Introduction

**Scheme 1:**
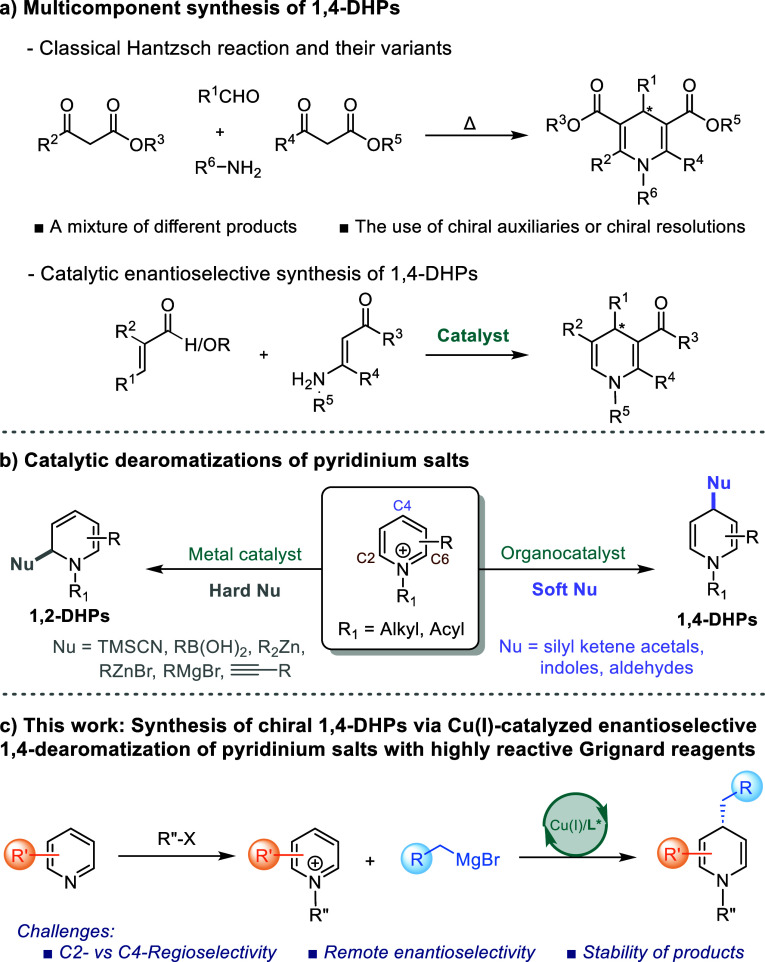
Asymmetric Synthesis of 1,4-DHPs with a Carbon Stereocenter

1,4-Dihydropyridines (1,4-DHPs) serve as the structural basis of various alkaloid natural products and pharmaceuticals, exhibiting a diverse array of biological activities, including antihypertensive, calcium channel blocking, anticancer, anti-inflammatory, antimicrobial, antioxidant, neurotropic, analgesic, and antidiabetic properties.^1^ Due to their structural resemblance to nicotinamide adenine dinucleotide, 1,4-DHPs offer numerous applications in synthetic and medicinal chemistry as well as in drug discovery.^2^ Traditionally, these molecules are synthesized using the Hantzsch condensation^3^ or a modified version of this transformation (Scheme 1a).^4,5^ However, this multicomponent method typically yields only symmetrical 1,4-DHPs and a variety of different products.^6^ Over the years, asymmetric approaches to the formation of 1,4-DHPs have primarily relied on asymmetric auxiliaries^7^ or chiral resolutions.^8^ Additionally, various strategies in asymmetric organocatalysis, including BINOL-derived phosphoric acid,^9^ (thio)urea,^10^ cinchona-alkaloid^11^ and amine catalysis,^12^ have been developed for the construction of enantiopure 1,4-DHPs (Scheme 1a). In addition, *N*,*N′*-dioxide/Ni^II^ or Ni^III^ complex catalysts were applied to synthesize Hantzsch-dihydropyridines, as well.^13^ However, most of these methods depend on the use of highly reactive carbonyl compounds such as aldehydes and ketones. Overall, the development of asymmetric metal catalysis remains a significant challenge in the preparation of chiral 1,4-DHPs.

Direct catalytic dearomatization of readily available pyridinium salts has become increasingly popular for the preparation of functionalized chiral three-dimensional molecular architectures, providing access to partially or fully saturated *N*-heterocycles. Some of the most developed routes for dearomatization of pyridinium salts utilize carbon nucleophiles and involve either the stoichiometric incorporation of chiral auxiliaries onto the pyridine core^14^ or the use of preformed chiral nucleophiles^15^ to obtain stereoselectivity. However, controlling the regio- and stereoselectivity of the dearomatization reaction remains a significant synthetic challenge.

In particular, the stereocontrolled C4-regioselective functionalization of pyridinium salts with carbon nucleophiles has proven to be especially difficult to develop. Until recently, the catalytic asymmetric 1,4-dearomatization of *N*-alkyl/acylpyridinium salts was unknown. The only known precedents involved organocatalysts such as triazole-based H-bond donors,^16^ bifunctional tertiary amino-thioureas,^17^*N*-heterocyclic carbenes,^18^ and amine catalysis^19^ in the presence of soft nucleophiles and an example of carbon–silicon bond formation^20^ (Scheme 1b). Alternatively, transition metal-catalyzed asymmetric nucleophilic dearomatization reactions of pyridinium salts have been achieved using various nucleophiles, such as silyl cyanide,^21^ terminal alkynes,^22^ organoboron,^23^ organometallic reagents.^24^ However, these transformations typically provide access to a limited reaction scope and install the stereocenter at the C2- or C6-positions without yielding any C4-addition products (Scheme 1b). The only example of catalytic enantioselective dearomative C4-functionalization of pyridinium species with carbon nucleophiles is the reaction of 2-methoxypyridines to access chiral δ-lactams, the C4-regioselectivity in this case was controlled by the substituent present at C2-position.^25^

Herein, we present a method for the catalytic asymmetric C4-selective dearomative C–C bond forming functionalization of pyridinium salts offering regio- and enantioselective access to chiral 1,4-DHP derivatives (Scheme 1c). This transformation is enabled by the synergistic action of a chiral copper catalyst and highly reactive Grignard reagents. Experimental ^13^C kinetic isotope effect (KIE) studies and density functional theory (DFT) calculations have provided insights into the reaction mechanism, elucidating the origin of the regio- and enantioselectivity of the reaction, as well as the rate-limiting step in the catalytic process.

## Results and Discussion

Initially, we tested the reaction of *N*-benzyl-3-cyanopyridinium salt **1a** with ethylmagnesium bromide (EtMgBr) in CH_2_Cl_2_ at −78 °C (Table 1). After 16 h, we obtained a mixture of the C4-addition product **3a** (1,4-DHP) and the C2-addition product **3a′** (1,2-DHP) in 46% yield with a low regioisomeric ratio (57:43, entry 1). To improve the regioselectivity, we introduced a catalytic amount of copper(I) salt. This adjustment led to a significantly improved yield of 82% and an enhanced regioselectivity of 73:27 (entry 2). Motivated by these results, we next examined different chiral ligands in combination with the Cu(I) salt (see Supporting Information for full details). Several chiral BOX (**L1** and **L2**), phosphoramidite (**L3**), ferrocene-based bis-phosphine (**L4**), and diphosphine Ph-BPE (**L5**) ligands provided DHP products in high yields but with poor stereocontrol (entries 3–8). Notably, we found that only biaryl bis-phosphine ligands (**L6–L11**) improved the enantioselectivity of the reaction (entries 9–15; 35–78% ee). Among these ligands, (*R*)-Tol-Binap (**L11**) emerged as the best one for this transformation, affording product **3a** with 78% ee.

**Table 1:**
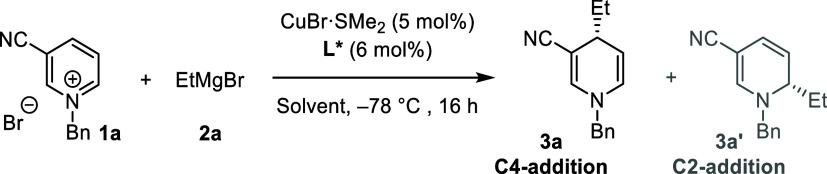

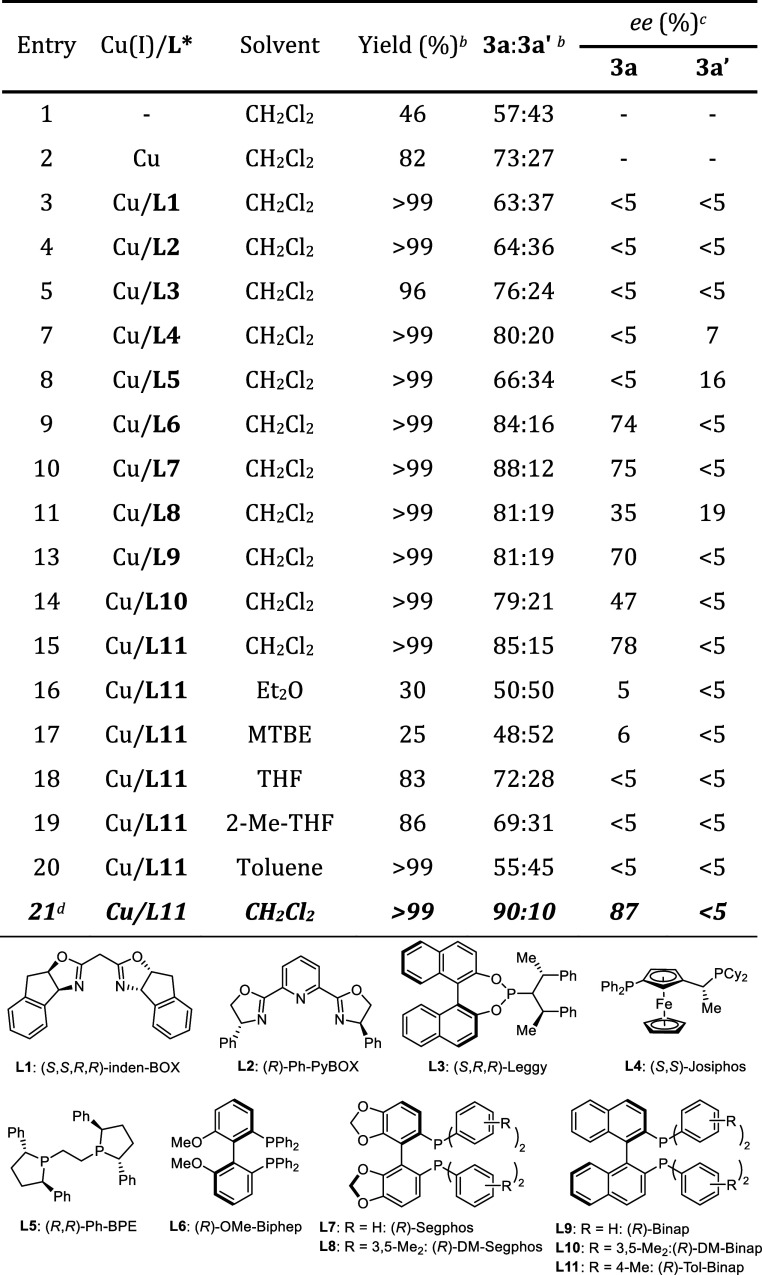
Optimisation of the Reaction Conditions for the Cu(I)-Catalyzed 1,4-Dearomatization of **1a** with EtMgBr^a^

a **Reaction conditions: 1a** (0.2 mmol, 1.0 equiv), CuBr·SMe_2_ (5 mol %), chiral ligand L* (6 mol %), EtMgBr in Et_2_O (3 M; 0.24 mmol, 1.2 equiv) in solvent (2 mL) for 16 h.

b The yields of **3a** and **3a′** were determined by ^1^H nuclear magnetic resonance (NMR) spectra of the reaction crude using 1,3,5-trimethoxybenzene as an internal standard.

c Enantiomeric excess (ee) was determined by supercritical-fluid chromatography (SFC) with a chiral stationary phase.

d In this case CuTC (10 mol %) and (*R*)-**L11** (12 mol %) were used.

At this point, we looked into the solvent effect. While reactions in Et_2_O, methyl *tert*-butyl ether (MTBE), THF, 2-methyltetrahydrofuran (2-Me-THF), and toluene resulted in racemic mixtures of products **3a** and **3a′** (entries 16–20), CH_2_Cl_2_ was found to be the most suitable solvent for this reaction, yielding 85% of **3a** with 78% ee (entry 15).

We then screened different copper salts, but none provided improved results (see the Supporting Information). Notably, increasing the catalytic loading to 10 mol % gave the products in high yield (>99%) with good regioselectivity (90:10) and high enantioselectivity (87% ee). Based on these findings, we established the following optimal reaction conditions: CuTC (10.0 mol %), (*R*)-**L11** (12.0 mol %), and Grignard reagent (1.2 equiv) in CH_2_Cl_2_ at −78 °C for 16 h.

With optimized the reaction conditions, we next investigated how the *N*-substituent in alkyl-pyridinium salts (Table 2) affected the reaction. *N*-Benzylpyridinium salts with *ortho* Me and ^*t*^Bu substituents yielded 1,4-DHP products **3b** and **3c** with high yields (82–87%) and enantiopurities (85–86% ee). The reaction also tolerated electron-withdrawing and halogen groups, producing **3d** and **3e** with good yields (75–82%) but moderate ee’s (59–73%). Introducing a naphthyl aryl substituent led to **3f** with similar results.

**Table 2:**
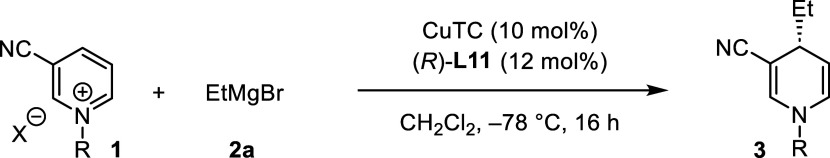

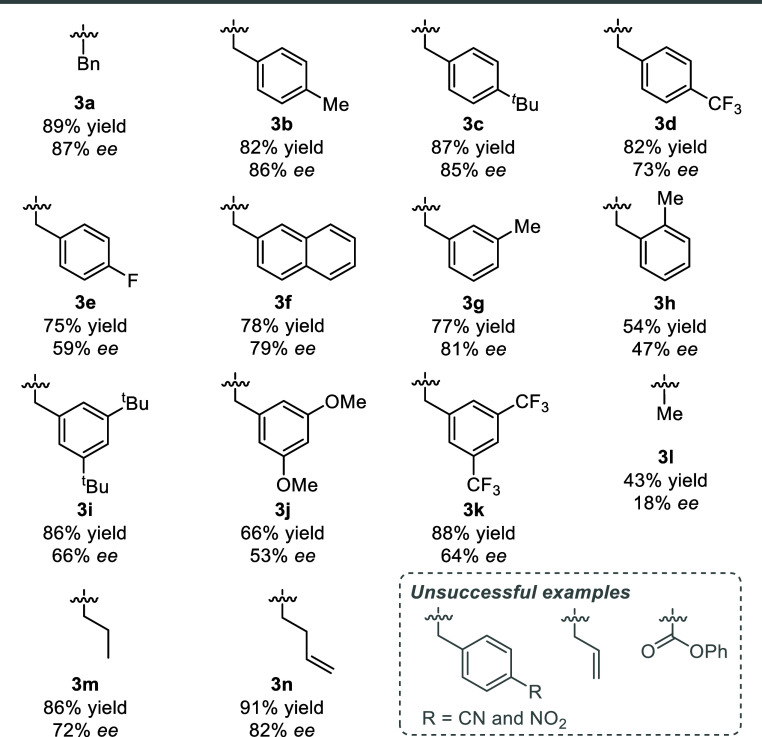
Variation of N-Substituent in the 3-Cyanopyridinium Salts^a^

a **Reaction conditions: 1a** (0.2 mmol, 1.0 equiv), CuTC (10 mol %), chiral ligand (*R*)-**L11** (12 mol %), EtMgBr in Et_2_O (3 M; 0.24 mmol, 1.2 equiv) in CH_2_Cl_2_ (2.0 mL) for 16 h. Reported yields correspond to the isolated yields. For each compound, ee values were determined by SFC on a chiral stationary phase.

A methyl group at the *meta*-position of the *N*-benzyl group led to **3g** (77% yield, 81% ee), while *ortho*-substitutions produced **3h** with a 54% yield and 47% ee. Disubstitutions with electron-rich and electron-deficient groups resulted in the corresponding products with diminished enantiopurities (**3i**–**3k**; 66–88% yield, 53–66% ee). Using *N*-methyl-3-cyanopyridinium iodide resulted in **3l** in moderate yield (43%) and low enantioselectivity (18% ee). Changing the *N*-substituent to *n*-propyl and 1-butenyl resulted in products **3m** and **3n** with improved yields (86–91%) and ees (72–82%).

Based on these results, we selected *N*-benzyl-3-cyanopyridinium salt **1a** as the model substrate to investigate the scope of Grignard reagents (Table 3). Various linear Grignard reagents yielded products **4a**–**4f** with high yields (up to 91%) and enantioselectivities (up to 94% ee). The reaction also tolerated β- and γ-branched Grignard reagents, producing **4g**–**4i** with yields of up to 92% and enantiomeric purities of up to 91% ee.

**Table 3:**
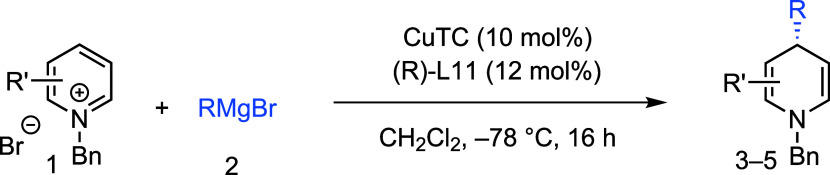

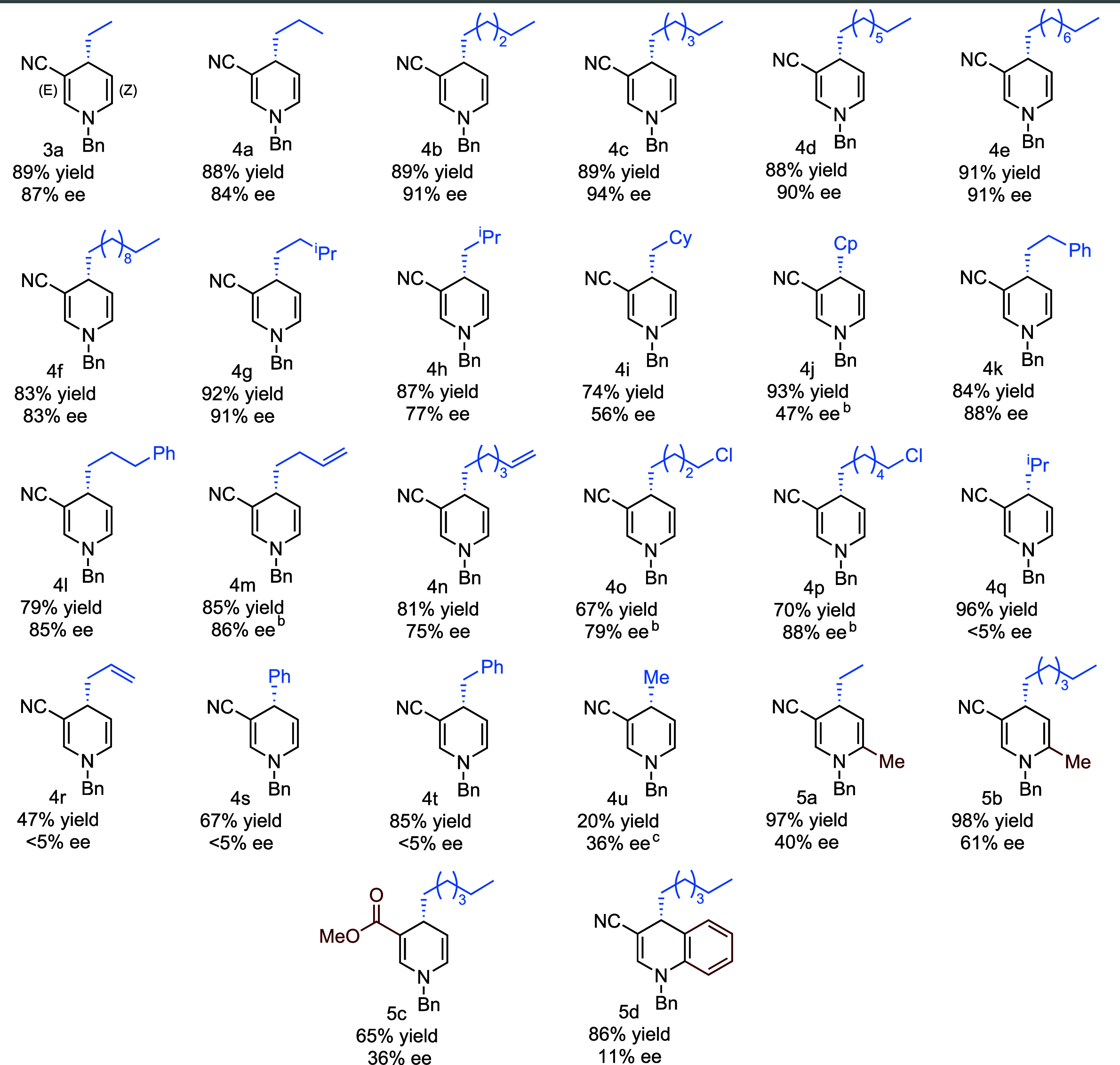
Product Scope of Cu(I)-Catalyzed Asymmetric 1,4-Dearomatization of Pyridinium Salts with Grignard Reagents^a^^,^^b^^,^^c^

a **Reaction conditions: 1** (0.2 mmol, 1.0 equiv), CuTC (10 mol %), (*R*)-**L11** (12 mol %), Grignard reagent (0.24 mmol, 1.2 equiv) in CH_2_Cl_2_ (2.0 mL) for 16 h.

b In this case CuTC (20 mol %) and (*R*)-**L11** (24 mol %) were used.

c In this case the reaction was performed at −40 °C. Reported yields correspond to the isolated yields. ee values were determined by SFC on a chiral stationary phase. Absolute configuration was determined for **4c** (see the Supporting Information). The configuration of other compounds was assigned by analogy.

Cyclopentylmagnesium bromide, a sterically demanding α-branched Grignard reagent, reacted with **1a** to yield **4j** with high yield but lower ee (47%). Functionalized Grignard reagents with β- and γ-phenyl substituents, terminal olefins, and halogen groups afforded products **4k**–**4p** in high yields (up to 84%) and high enantiopurities (up to 88%). When using MeMgBr, no product was formed at the optimized reaction temperature due to its lower reactivity. However, conversion to the addition product (C4:C2 = 1:3) was observed at an increased reaction temperature of −40 °C, allowing for the isolation of the C4 addition product **4u** with a 20% isolated yield and 36% enantiopurity. Other Grignard reagents, such as ^*i*^PrMgBr, AllylMgBr, PhMgBr, and BnMgBr, led to racemic products.

The evaluation of the reaction scope revealed that the activating cyano substituent at the C3 position of the pyridinium salt is the key structural element required for the reactivity of this class of substrates. Substrates with additional substituents on the pyridine moiety or with the CN group replaced were compatible with the optimized reaction protocol, albeit leading to the corresponding products **5a**–**5d** with lower enantioselectivities.

At this stage, to enhance our understanding of this transformation, we conducted both experimental and in silico mechanistic studies. Our findings revealed that once the **L11CuEt** complex is formed between the Cu(I) salt and EtMgBr (see the Supporting Information), it will coordinate to **1a**. To corroborate that monomeric species is formed, we studied the dependence of enantiomeric excess of the product **4g** on the enantiomeric excess of the chiral diphosphine ligand **L11**. We have found that there is a linear correlation, supporting the likely monomeric identity of copper species (see the Supporting Information).

**Figure 1:**
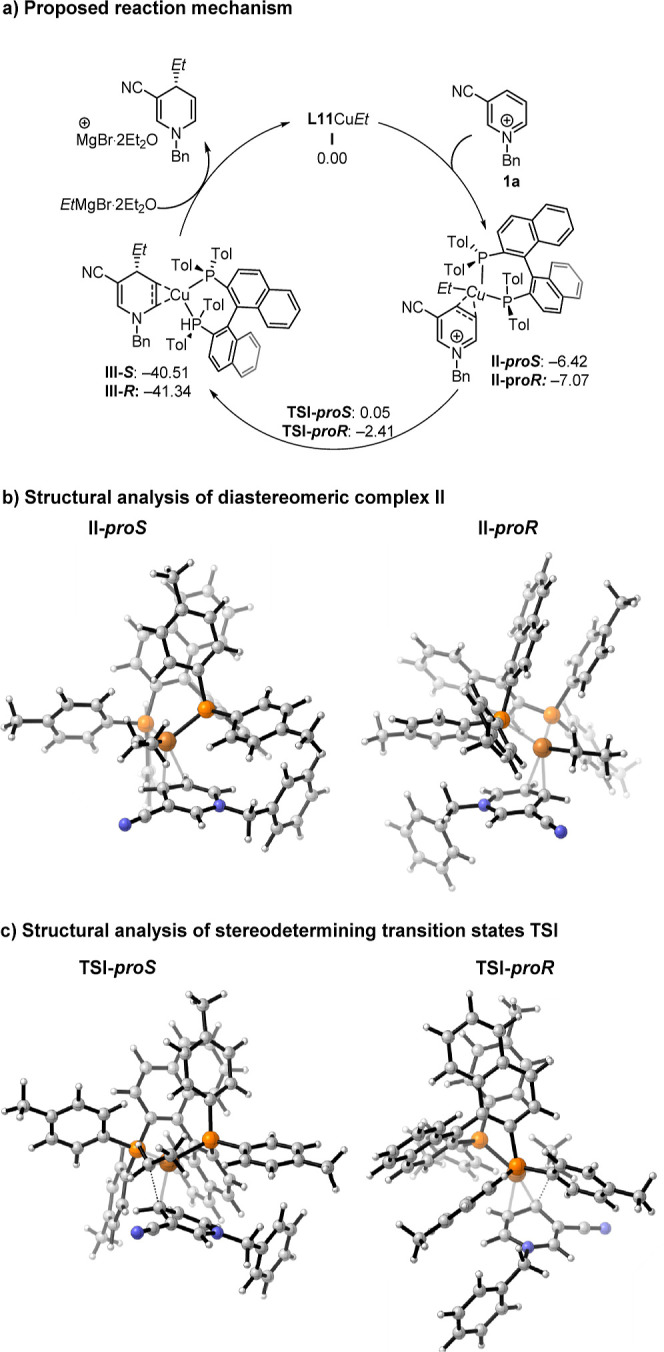
a) DFT calculation for Cu(I)-catalyzed enantioselective 1,4-dearomatization of pyridinium salts; free energy in kcal/mol. 3D images of the (b) diastereomeric complexes and (c) diastereomeric transition states. For computational details see the Supporting Information.

Using molecular modeling, we explored the interaction between the substrate **1a** and the **L11CuEt** copper complex. We found that the copper complex can coordinate with the substrate without the decoordination of any of the phosphorus groups. This result contrasts sharply with our findings for quinolines and other pyridine derivatives.^24d,26^ We attribute this discrepancy to the coordination of both phosphorus atoms around the metal center, which is influenced by the presence of a second ligand characterized by a significant electron deficiency: the pyridinium salt. The electron deficiency of the substrate allows the phosphorus groups to remain coordinated to the metal core. The coordination of the substrate with the copper complex results in the formation of two diastereomeric species depending on which face of the substrate is coordinated. Our findings show minimal energy difference between the resulting diastereomeric complexes (**II**-***proS***: −6.42 kcal/mol and **II**-***proR***: −7.07 kcal/mol) (Figure 1a). Once the complex coordinates to the substrate, the ethyl group is delivered to the C4 position, forming copper-DHP intermediate **III**. This step is most likely the stereodetermining. In **TSI-*proR***, the Cu to methylenic carbon distance is 2.04 Å, compared to 2.23 Å in **TSI**-***proS***. The activation energy of **TSI-*proR*** is 4.66 kcal/mol lower than that of **TSI-*proS*** (6.47 kcal/mol) (Figure 1b,c).

Next, we investigated ^13^C isotope effects both experimentally and computationally. Determining KIEs can reveal major bonding changes in the rate-limiting step of a reaction.^27^ We used high-precision NMR measurements of ^13^C KIEs at natural isotopic abundance, as developed by Singleton.^28^ To detect KIEs from the Grignard reagent, we studied intermolecular ^13^C KIEs via product analysis.^29^ We conducted three independent reactions of **1a** and ^*i*^BuMgBr under optimized conditions on an 8 mmol scale, and stopped at low conversions to the product **4h** (12, 15, and 7%). The product **4h** was isolated, and its ^13^C isotopic composition was compared to those of unreacted samples of **1a** and ^*i*^BuMgBr.

From changes in relative isotopic composition and fractional conversion, we determined ^13^C KIEs (details in the Supporting Information). We found a primary KIE of ∼2.4% on C4 of the 1,4-DHP ring and ∼2.0% on C1′ of the isobutyl moiety, indicating that C4 and C1′ are involved in the rate-determining step or the first irreversible step in the catalytic process, while negligible isotope effects on other carbons suggest their lack of involvement in the rate-determining step. The observed KIEs on both carbons strongly support that transferring the carbon nucleophile of the Grignard reagent to the electrophilic site C4 of the pyridinium salt is the rate-determining step in the catalytic cycle. The experimental ^13^C KIEs align perfectly with the predicted KIEs of our proposed catalytic cycle (Table 4), reinforcing our mechanistic proposal.

**Table 4:**
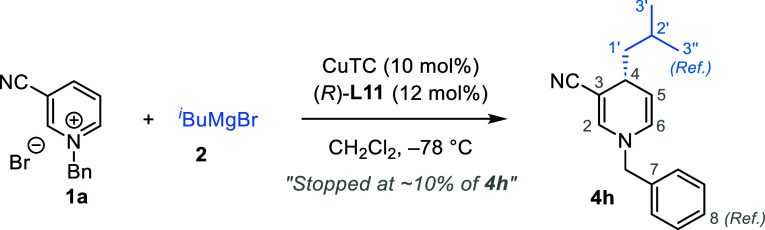
Comparison of Experimental and Predicted ^13^C KIEs for the Addition of Grignard Reagents to **1a** from Product Analysis

	experimental^a^			predicted^b^
	exp. 1	exp. 2	exp. 3	
CN	1.000 (1)	1.000 (1)	1.000 (1)	1.005
C2	1.000 (1)	1.000 (1)	1.000 (1)	1.001
C3	1.001 (1)	1.002 (1)	1.002 (1)	1.008
***C4***	***1.025 (1)***	***1.024 (2)***	***1.024 (1)***	***1.025***
C5	1.005 (1)	1.005 (2)	1.006 (1)	1.010
C6	1.001 (1)	1.000 (1)	1.000 (1)	1.006
C7	1.000 (1)	1.000 (1)	1.000 (1)	1.004
C8	1.000 (reference)			
***C1***′	***1.020 (2)***	***1.020 (2)***	***1.021 (3)***	***1.024***
C2′	1.000 (1)	1.000 (2)	1.000 (1)	0.995
C3′	0.999 (1)	1.000 (1)	0.999 (1)	1.000
C3″	1.000 (reference)			

a The numbers in parentheses represent the standard deviation in the last digit as determined from five independent measurements.

b Predicted KIEs were computed at the B3LYP-D3/def2tzvpp//B3LYP-D3/def2svpp computational level, based on the transition structure **TSI-*proR*** of product **4h**.

**Scheme 2:**
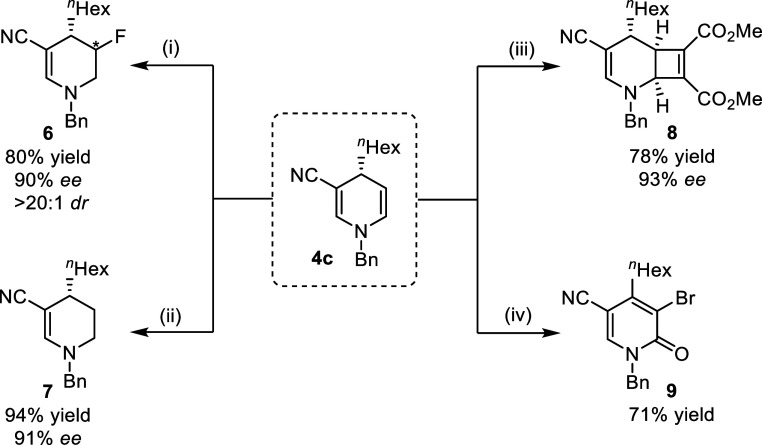
Synthetic Transformations Reaction conditions: (i) Selecfluor, BH_3_NMe_3_, CH_2_Cl_2_, rt, 16 h; (ii) Pd/C (10 mol %), H_2_ (1 atm), MeOH, rt, 16 h; (iii) dimethyl acetylenedicarboxylate, MeCN, 90 °C, 4 h; (iv) NBS, CH_2_Cl_2_, rt, 16 h.

To demonstrate the methodology’s robustness, a gram–scale reaction was performed under standard conditions, yielding the 1,4-DHP product **4c** in 90% yield with 92% ee (see the Supporting Information). This product can serve as a versatile building block for various transformations (Scheme 2). For instance, hydrofluorination of 1,4-DHP **4c** to **6** was achieved in 80% yield as a >20:1 mixture of diastereomers using Selecfluor and BH_3_·NMe_3_.^30^ Additionally, Pd-catalyzed hydrogenation of the alkene in **4c** led to the selective reduction of tetrahydropyridine product **7** in nearly quantitative yield. The bicyclic product **8** was formed from a formal [2 + 2] cycloaddition between **4c** and dimethyl acetylenedicarboxylate. Furthermore, the reaction of *N*-bromosuccinimide (NBS) with **6** proceeded smoothly, yielding bromo-substituted pyridone **9** in a good yield. These successful transformations highlight the potential applicability of saturated *N*-heterocyclic molecules.

## Conclusions

In conclusion, we developed the highly regio- and enantioselective copper-catalyzed nucleophilic 1,4-dearomatization of pyridinium salts using Grignard reagents. This method produces enantioenriched chiral 1,4-DHP derivatives. The synthetic utility was demonstrated through easy scale-up and successful product transformations. Combined experimental and computational studies provided insights into the reaction mechanism, regio- and enantioselectivity, and rate-limiting steps.

## Supporting Information Available

Experimental procedures and characterization data; mechanistic studies; computational details; NMR spectra; and Cartesian coordinates (PDF)
