# Supplementary material for: Grignard Reagent Addition to Pyridinium Salts: A Catalytic Approach to Chiral 1,4-Dihydropyridines
Source: ACS Catal. 2024 Aug 15;14(17):13030–9. doi: 10.1021/acscatal.4c03520 (PMC11385375; doi:10.1021/acscatal.4c03520)
Supplement: Supplementary file 1 — cs4c03520_si_001.pdf [file cs4c03520_si_001.pdf]

Supporting Information

Grignard Reagent Addition to Pyridinium Salts: A Catalytic Approach to Chiral 1,4-Dihydropyridines

Siriphong Somprasong, Marta Castiñeira Reis, and Syuzanna R. Harutyunyan*

Syuzanna R. Harutyunyan – *Stratingh Institute for Chemistry, University of Groningen, 9747 AG, Groningen, The Netherlands;* orcid.org/0000-0003-2411-1250; Email: s.harutyunyan@rug.nl
Siriphong Somprasong – *Stratingh Institute for Chemistry, University of Groningen, 9747 AG Groningen, The Netherlands*; orcid.org/0000-0001-6323-5817
Marta Castiñeira Reis – *Centro Singular de Investigación en Química Biolóxica e Materiais Moleculares (CIQUS), C/ Jenaro de la Fuente s/n, Campus Vida, Universidade de Santiago de Compostela, 15782 Santiago de Compostela (Spain)*; orcid.org/0000-0003-4204-3474

Table of contents

1. General experimental information 3

2. Chemicals 4

3. Computational details 5

4. Synthesis of the pyridine derivatives 6

    4.1 General procedures 6

    4.2 Specific experimental details for the synthesis of pyridinium salts 6

5. Cu(I)-catalysed nucleophilic dearomatization of pyridines 15

    5.1 General procedure for asymmetric dearomatization 15

    5.2 Optimization of the reaction conditions 16

    5.3 Specific experimental details and product characterization 19

    5.4 Synthetic transformations 58

6. Mechanistic studies 63

    6.1 DFT calculations 63

    6.2 Nonlinear relationship study 67

    6.3 Racemization study 68

    6.4 Corroboration of the mechanistic proposal via quantitative KIE studies 69

7. Determination of absolute configuration 74

8. NMR spectra 75

9. Cartesian coordinates 146

10. References 172

1. General experimental information

All reactions using oxygen- and/or moisture-sensitive materials were carried out with anhydrous solvents under nitrogen atmosphere using standard Schlenk techniques. Analytical thin layer chromatography (TLC) was performed using 0.25 mm E. Merk silica plates (60F-254). Compounds were visualized under UV light or by staining with an aqueous Hanessian's stain (Cerium Ammonium Molybdate solution; CAM stain). Purification of the products was carried out by flash column chromatography, using Merck 60 Å 230–400 mesh silica gel. NMR data was collected on a Varian NMR spectrometer (^{1}H at 400 MHz; ^{13}C at 101 MHz; ^{19}F at 376 MHz) or a Bruker Avance 600 MHz NMR spectrometer (^{1}H at 600 MHz; ^{13}C at 151 MHz; ^{19}F at 565 MHz) with a 5 mm z-gradient broadband probe. Chemical shifts are reported in parts per million (ppm) relative to the resonance of the residual solvent peak (^{1}H: $CDCl_3$, δ = 7.26 ppm; DMSO-d_6, δ = 2.50 ppm and ^{13}C: $CDCl_3$, δ = 77.16 ppm; DMSO-d_6, δ = 39.52 ppm). Multiplicity is reported with the usual abbreviations: (brs = broad, s = singlet, d = doublet, dd = doublet of doublets, dt = doublet of triplets, dq = doublet of quartets, t = triplet, td = triplet of doublets, q = quartet, p = pentet and m = multiplet. Coupling constants, *J*, are reported in Hertz (Hz). Please note in some carbon NMR spectra symmetric carbons cannot be discerned and they are listed as one signal. If an apparent multiplicity is observed the actual multiplicity will be noted in brackets. Quantitative ^{13}C NMR spectra were recorded at 151 MHz on a Bruker Avanue 600 MHz NMR spectrometer with inverse-gated decoupling. High resolution mass spectra (HRMS) were recorded on a LTQ Orbitrap XL apparatus in the ESI and APCI ionization modes. Enantiomeric excess (*ee*) was determined a chiral SFC analysis using a Waters Acquity UntraPerformance Chromatography (UPC^{2}) equipped with a PDA detector. Circular dichroism (CD) were recorded on a JASCO810. Unless otherwise noted, quantitative ^{1}H NMR conversions and yields were determined from crude reaction mixtures using 1,3,5-trimethoxybenzene as an internal standard.

2. Chemicals

Unless otherwise indicated, reagents and substrates were purchased from commercial sources and used as received. Substrate **1a–1t** were synthesized according to the reported literature procedures. Dry solvents were freshly collected from a dry solvent purification system prior to use. Inert atmosphere experiments were performed with standard Schlenk techniques with dried (P_2O_5) nitrogen gas. ^{n}NonMgBr (1.5 M in Et_2O), ^{n}UndecMgBr (2.5 M in Et_2O), CyclohexCH$_2$MgBr (2.3 M in Et_2O), CH_2=CH(CH_2)$_2$MgBr (2.5 M in Et_2O), CH_2=CH(CH_2)$_4$MgBr (1.6 M in Et_2O), ClCH$_2$(CH_2)$_3$MgBr (2.3 M in Et_2O), ClCH$_2$(CH_2)$_5$MgBr (2.2 M in Et_2O), Ph(CH_2)$_2$MgBr (2.5 M in Et_2O), and Ph(CH_2)$_3$MgBr (3.3 M in Et_2O) were prepared from the corresponding alkyl bromides and Mg activated with I_2 in Et_2O. The synthesized Grignard reagents were titrated by ^{1}H NMR before use. MeMgBr, EtMgBr and PhMgBr (3.0 M in Et_2O); ^{n}PrMgBr, ^{n}PentMgBr, ^{n}HexMgBr, ^{n}OctMgBr, ^{i}BuMgBr, ^{i}PentMgBr, CyclopentylMgBr and ^{i}PrMgCl (2.0 M in Et_2O); AllylMgBr, BnMgCl (1.0 M in Et_2O) were purchased from Sigma-Aldrich. Chiral ligands were purchased from Sigma-Aldrich, TCI and Strem Chemicals. All reported compounds were characterized by ^{1}H and ^{13}C NMR and compared with literature data. All new compounds were fully characterized by ^{1}H NMR, ^{13}C NMR and HRMS techniques.

3. Computational details

We have used the Density Functional Theory (DFT) in the Kohn-Sham formulation[1] to optimize all the stationary points presented in this manuscript. Geometries of all the stationary points were fully optimized at the B3LYP-D3/def2tzvpp//B3LYP-D3/def2svpp[2] computational level. The effect of solvent (CH_2Cl_2) was modelled using the polarizable continuum model (PCM)[3] with the default parameters implemented in the Gaussian 16 package.[4] All geometry optimizations have been performed using tight convergence criteria in the SCF and requesting a pruned (99,590) grid to guarantee the accuracy of the reported results. Moreover, calculations were performed using the default parameters implemented in the Gaussian suite. The conditions of temperature and pressure used for the computations were 1 atm and 195 K, to simulate accurately the reaction conditions.

Harmonic analysis was used to establish the nature of all optimized structures as either minima or transition state structures. For all stationary points, the stability of the wave function was also confirmed.[5]

IRC calculations[6] were conducted for transition states to ensure their connectivity with the expected reactants and products. The stability of the wave function was analysed for all the presented stationary points. Conformational analysis was performed manually, it must be indicated that only the most stable conformer of each stationary point was considered and reported unless otherwise indicated.

The visualization of the reported structures was performed using MOLDEN.[7] The representation of the structures here presented were generated using CYLView or ChemDraw.[8]

To simulate the Quantitative Kinetic Isotope Effects, we used the program QUIVER,[9] QKIES were computed at -78˚C using the carbon 8 at the benzyl group as the reference in the case of the data obtained when EtMgBr was explored as a Grignard reagent.

The ECD spectra simulation was performed at the CAM-B3LYP[10]/def2TZVP[2] computational level. Fifty excited states were computed and a shift of 120 nm applied in the representation.

4. Synthesis of pyridinium salts

4.1 General procedures

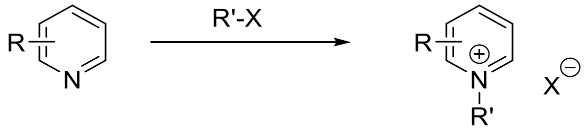

General procedure A

In a round bottom flask equipped with a magnetic stirring bar and a reflux condenser, the appropriate pyridine (1.0 equiv.) and the appropriate alkyl halide (1.2 equiv.) were dissolved in MeCN (1 M). The resulting mixture was stirred at 90 °C for 18 h. Here after, the mixture was cooled to 0 °C and Et_2O was added dropwise with stirring until a precipitate was formed. The solid was then filtered and washed repeatedly with cold Et_2O to afford the desired pyridinium salt. If no precipitation occurred, the mixture was purified by flash column chromatography (SiO_2, CH_2Cl_2:MeOH = 9:1).[11]

General procedure B

In a round bottom flask equipped with a magnetic stirring bar and a reflux condenser, the flask was charged with the appropriate pyridines (1.0 equiv.). The appropriate alkyl halide (1.5 equiv.) was added dropwise, and the resulting mixture was stirred at 90 °C for 48 h. The mixture was cooled to room temperature and cooled to 0 °C and stirred vigorously for 5 min. Et_2O was added, and the mixture was stirred vigorously at 0 °C for 15 min, at which point the product precipitated. The solid was then filtered and washed repeatedly with cold Et_2O to afford the desired pyridinium salt.

4.2 Specific experimental details for the synthesis of pyridinium salts

***N*-Benzyl-3-cyanopyridinium bromide (1a)**

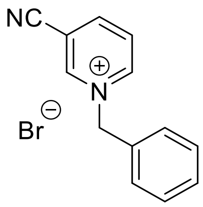

Product **1a** was synthesized following **General procedure A** with 3-cyanopyridine (937.0 mg, 9.0 mmol, 1.0 equiv.) and benzyl bromide (1.3 mL, 10.8 mmol, 1.2 equiv.) in MeCN (9.0 mL) to obtain **1a** as a white solid in 2.38 g (96% yield).

^{1}H NMR (DMSO-*d₆*, 400 MHz): δ 10.08 (t, *J* = 1.6 Hz, 1H, CH_{Py}), 9.50 (dt, *J* = 6.3 and 1.4 Hz, 1H, CH_{Py}), 9.13 (dt, *J* = 8.2 and 1.4 Hz, 1H, CH_{Py}), 8.38 (dd, *J* = 8.2 and 6.3 Hz, 1H, CH_{Py}), 7.66–7.61 (m, 2H, 2 × CH_{Ar}), 7.48–7.43 (m, 3H, 3 × CH_{Ar}), 5.96 (s, 2H, CH_2). NMR data are in agreement with literature precedents.[12]

^{13}C NMR (DMSO-d_6, 101 MHz): δ 149.0, 149.0, 148.1, 133.4, 129.6, 129.3 (2 × C), 129.1 (2 × C), 128.9, 113.9, 113.2, 63.8.

LC-HRMS (APCI): m/z $[M–Br]^+$ calcd. for $C_{13}H_{11}N_2^+$: 195.0917; found 195.0877.

***N*-(4-Methylbenzyl)-3-cyanopyridinium bromide (1b)**

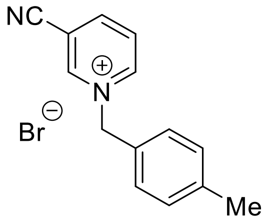

Product **1b** was synthesized following **General procedure A** with 3-cyanopyridine (937.0 mg, 9.0 mmol, 1.0 equiv.) and 4-methylbenzyl bromide (2.0 g, 10.8 mmol, 1.2 equiv.) in MeCN (9.0 mL) to obtain **1b** as a white solid in 2.53 g (97% yield).

^{1}H NMR (DMSO-*d$_6$*, 400 MHz): δ 10.06 (s, 1H, CH_{Py}), 9.48 (dt, *J* = 6.3 and 1.4 Hz, 1H, CH_{Py}), 9.12 (dt, *J* = 8.1 and 1.4 Hz, 1H, CH_{Py}), 8.37 (dd, *J* = 8.1 and 6.3 Hz, 1H, CH_{Py}), 7.53 (d, *J* = 8.0 Hz, 2H, 2 × CH_{Py}), 7.26 (d, *J* = 8.0 Hz, 2H, 2 × CH_{Py}), 5.91 (s, 2H, CH_2), 2.30 (s, 3H, CH_3).

^{13}C NMR (DMSO-*d$_6$*, 101 MHz): δ 148.9, 148.9, 147.9, 139.2, 130.4, 129.7 (2 × C), 129.3 (2 × C), 128.8, 113.9, 113.1, 63.7, 20.8.

LC-HRMS (APCI): m/z [M–Br]$^+$ calcd. for $C_{14}H_{13}N_2^+$: 209.1073; found 209.0856.

***N*-(4-(*Tert*-butyl)benzyl)-3-cyanopyridinium bromide (1c)**

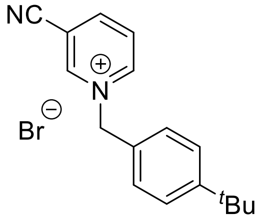

Product **1c** was synthesized following **General procedure A** with 3-cyanopyridine (937.0 mg, 9.0 mmol, 1.0 equiv.) and 4-*tert*-butylbenzyl bromide (2.0 mL, 10.8 mmol, 1.2 equiv.) in MeCN (9.0 mL) to obtain **1c** as a white solid in 2.81 g (94% yield). NMR data are in agreement with literature precedents.[13]

^{1}H NMR (DMSO-*d$_6$*, 400 MHz): δ 10.03 (s, 1H, CH_{Py}), 9.45 (dt, *J* = 6.3 and 1.4 Hz, 1H, CH_{Py}), 9.11 (dt, *J* = 8.2 and 1.4 Hz, 1H, CH_{Py}), 8.36 (dd, *J* = 8.2 and 6.3 Hz, 1H, CH_{Py}), 7.60–7.51 (m, 2H, 2 × CH_{Ar}), 7.51–7.43 (m, 2H, 2 × CH_{Ar}), 5.87 (s, 2H, CH_2), 1.26 (s, 9H, 3 × CH_3).

^{13}C NMR (DMSO-*d$_6$*, 101 MHz): δ 152.2, 149.0, 148.9, 148.0, 130.5, 129.1 (2 × C), 128.9, 126.0 (2 × C), 113.9, 113.3, 63.8, 34.5, 31.0.

LC-HRMS (APCI): m/z [M–Br]$^+$ calcd. for $C_{17}H_{19}N_2^+$: 251.1543; found 251.1540.

***N*-(4-(Trifluoromethyl)benzyl)-3-cyanopyridinium bromide (1d)**

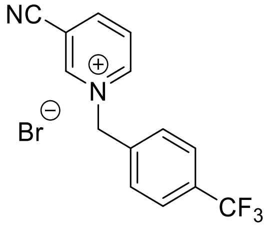

Product **1d** was synthesized following **General procedure A** with 3-cyanopyridine (520.0 mg, 5.0 mmol, 1.0 equiv.) and 4-(trifluoromethyl)benzyl bromide (1.43 g, 6.0 mmol, 1.2 equiv.) in MeCN (5.0 mL) to obtain **1d** as a white solid in 1.67 g (97% yield).

^{1}H NMR (DMSO-d_6, 400 MHz): δ 10.10 (s, 1H, CH_{Py}), 9.55 (dt, J = 6.3 and 1.4 Hz, 1H, CH_{Py}), 9.16 (dt, J = 8.2 and 1.4 Hz, 1H, CH_{Py}), 8.41 (dd, J = 8.2 and 6.3 Hz, 1H, CH_{Py}), 7.88–7.81 (m, 4H, 4 × CH_{Ar}), 6.10 (s, 2H, CH_2).
^{13}C NMR (DMSO-d_6, 101 MHz): δ 149.4, 149.1, 148.4, 137.7, 137.7, 130.2, 130.0, 129.7, 128.9, 126.0 (q, J = 3.8 Hz), 125.3, 122.6, 119.9, 113.9, 113.3, 62.9.
^{19}F NMR (DMSO-d_6, 376 MHz): δ –61.31.
LC-HRMS (APCI): m/z [M–Br]$^+$ calcd. for $C_{14}H_{10}F_3N_2^+$: 263.0791; found 263.1472.

N-(4-Fluorobenzyl)-3-cyanopyridinium bromide (1e)

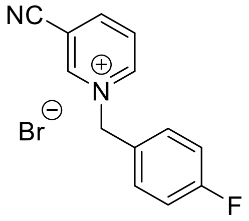

Product **1e** was synthesized following **General procedure A** with 3-cyanopyridine (937.0 mg, 9.0 mmol, 1.0 equiv.) and 4-fluorobenzyl bromide (1.3 mL, 10.8 mmol, 1.2 equiv.) in MeCN (9.0 mL) to obtain **1e** as a white solid in 2.45 g (93% yield).
^{1}H NMR (DMSO-d_6, 400 MHz): δ 10.05 (s, 1H, CH_{Py}), 9.50 (dt, J = 6.2 and 1.4 Hz, 1H, CH_{Py}), 9.13 (dd, J = 8.2 and 1.4 Hz, 1H, CH_{Py}), 8.38 (dd, J = 8.2 and 6.3 Hz, 1H, CH_{Py}), 7.76–7.72 (m, 2H, 2 × CH_{Ar}), 7.33–7.29 (m, 2H, 2 × CH_{Ar}), 5.95 (s, 2H, CH_2).
^{13}C NMR (DMSO-d_6, 101 MHz): δ 164.0, 161.5, 149.1, 149.0, 148.0, 132.1, 132.0, 129.6, 129.5, 128.8, 116.2, 116.0, 113.9, 113.2, 63.0.
^{19}F NMR (DMSO-d_6, 376 MHz): δ –111.78 (tt, J = 9.0 and 5.5 Hz)..
LC-HRMS (APCI): m/z [M–Br]$^+$ calcd. for $C_{13}H_{10}FN_2^+$: 213.0823; found 213.0776.

N-(Naphthalen-2-ylmethyl)-3-cyanopyridinium bromide (1f)

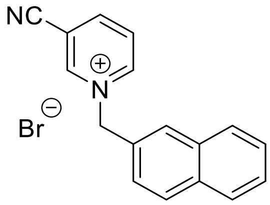

Product **1f** was synthesized following **General procedure A** with 3-cyanopyridine (520.0 mg, 5.0 mmol, 1.0 equiv.) and 2-(bromomethyl)naphthalene (1.33 g, 6.0 mmol, 1.2 equiv.) in MeCN (5.0 mL) to obtain **1f** as a white solid in 1.54 g (95% yield).
^{1}H NMR (DMSO-d_6, 400 MHz): δ 10.14 (s, 1H, CH_{Py}), 9.58 (dt, J = 6.3 and 1.4 Hz, 1H, CH_{Py}), 9.14 (dt, J = 8.2 and 1.4 Hz, 1H, CH_{Py}), 8.39 (dd, J = 8.2 and 6.3 Hz, 1H, CH_{Py}), 8.20 (s, 1H, CH_{Ar}), 8.00 (d, J = 8.5 Hz, 1H), 7.97–7.92 (m, 2H, 2 × CH_{Ar}), 7.72, (dd, J = 8.5 and 1.8 Hz, 1H, CH_{Ar}), 7.60–7.56 (m, 2H, 2 × CH_{Ar}), 6.15 (s, 2H, CH_2).
^{13}C NMR (DMSO-d_6, 101 MHz): δ 149.2, 149.0, 148.2, 133.0, 132.6, 130.8, 129.1, 129.0, 128.8, 128.1, 127.7, 127.2, 126.9, 126.1, 113.9, 113.2, 64.0.
LC-HRMS (APCI): m/z [M–Br]$^+$ calcd. for $C_{17}H_{13}N_2^+$: 245.1073; found 245.1258.

N-(3-Methylbenzyl)-3-cyanopyridinium bromide (1g)

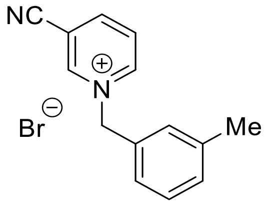

Product **1g** was synthesized following **General procedure A** with 3-cyanopyridine (937.0 mg, 9.0 mmol, 1.0 equiv.) and 3-methylbenzyl bromide (1.5 mL, 10.8 mmol, 1.2 equiv.) in MeCN (9.0 mL) to obtain **1g** as a white solid in 2.41 g (93% yield).

^{1}H NMR (DMSO-d_6, 400 MHz): δ 10.07 (s, 1H, CH_{Py}), 9.49 (dt, J = 6.3 and 1.4 Hz, 1H, CH_{Py}), 9.13 (dt, J = 8.2 and 1.4 Hz, 1H, CH_{Py}), 8.38 (dd, J = 8.2 and 6.3 Hz, 1H, CH_{Py}), 7.46–7.41 (m, 2H, 2 × CH_{Ar}), 7.33 (t, J = 7.6 Hz, 1H, CH_{Ar}), 7.26–7.23 (m, 1H, CH_{Ar}), 5.91 (s, 2H, CH_2), 2.31 (s, 3H, CH_3).

^{13}C NMR (DMSO-d_6, 101 MHz): δ 149.0, 149.0, 148.0, 138.5, 133.3, 130.2, 129.8, 129.1, 128.8, 126.3, 113.9, 113.2, 63.8, 20.9.

LC-HRMS (APCI): m/z [M–Br]$^+$ calcd. for $C_{14}H_{13}N_2^+$: 209.1073; found 209.1072.

N-(2-Methylbenzyl)-3-cyanopyridinium bromide (1h)

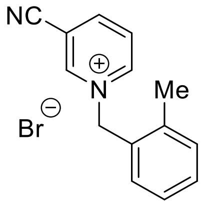

Product **1h** was synthesized following **General procedure A** with 3-cyanopyridine (937.0 mg, 9.0 mmol, 1.0 equiv.) and 2-methylbenzyl bromide (1.4 mL, 10.8 mmol, 1.2 equiv.) in MeCN (9.0 mL) to obtain **1h** as a white solid in 2.29 g (88% yield). NMR data are in agreement with literature precedents.[14]

^{1}H NMR (DMSO-d_6, 400 MHz): δ 9.90 (s, 1H, CH_{Py}), 9.29 (dt, J = 6.3 and 1.4 Hz, 1H, CH_{Py}), 9.16 (dt, J = 8.1 and 1.4 Hz, 1H, CH_{Py}), 8.39 (dd, J = 8.1 and 6.3 Hz, 1H, CH_{Py}), 7.39–7.27 (m, 4H, 4 × CH_{Ar}), 6.00 (s, 2H, CH_2), 2.33 (s, 3H, CH_3).

^{13}C NMR (DMSO-d_6, 101 MHz): δ 149.1, 148.2, 137.3, 131.3, 130.9, 130.9, 129.7, 129.6, 128.8, 126.6, 113.9, 113.3, 62.1, 18.9.

LC-HRMS (APCI): m/z [M–Br]$^+$ calcd. for $C_{14}H_{13}N_2^+$: 209.1073; found 209.1072.

N-(3,5-Di-*tert*-butylbenzyl)-3-cyanopyridinium bromide (1i)

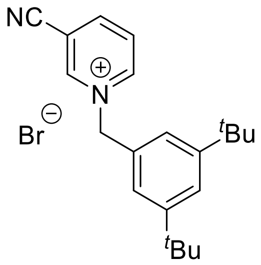

Product **1i** was synthesized following **General procedure A** with 3-cyanopyridine (312.3 mg, 3.0 mmol, 1.0 equiv.) and 3,5-di-*tert*-butylbenzyl bromide (1.02 g, 3.6 mmol, 1.2 equiv.) in MeCN (3.0 mL) to obtain **1i** as a white solid in 1.01 g (87% yield).

^{1}H NMR (DMSO-d_6, 400 MHz): δ 10.08 (s, 1H, CH_{Py}), 9.49 (dt, J = 6.3 and 1.4 Hz, 1H, CH_{Py}), 9.11 (dt, J = 8.2 and 1.4 Hz, 1H, CH_{Py}), 8.37 (dd, J = 8.2 and 6.3 Hz, 1H, CH_{Py}), 7.50 (d, J = 1.8 Hz, 2H, 2 × CH_{Ar}), 7.45 (t, J = 1.8 Hz, 1H, CH_{Ar}), 5.86 (s, 2H, CH_2), 1.28 (s, 18H, 6 × CH_3).
^{13}C NMR (DMSO-d_6, 101 MHz): δ 151.5 (2 × C), 149.0, 149.0, 147.9, 132.6, 128.9, 123.7 (2 × C), 123.3, 114.0, 113.2, 64.7, 34.8 (2 × C), 31.2 (6 × C).
LC-HRMS (APCI): m/z [M–Br]$^+$ calcd. for $C_{21}H_{27}N_2^+$: 307.2169; found 307.1431.

***N*-(3,5-Dimethoxybenzyl)-3-cyanopyridinium bromide (1j)**

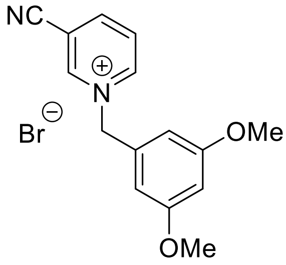

Product **1j** was synthesized following **General procedure A** with 3-cyanopyridine (312.3 mg, 9.0 mmol, 1.0 equiv.) and 3,5-dimethoxybenzyl bromide (2.50 g, 10.8 mmol, 1.2 equiv.) in MeCN (9.0 mL) to obtain **1j** as a pale yellow solid in 2.88 g (95% yield).
^{1}H NMR (DMSO-d_6, 400 MHz): δ 10.03 (s, 1H, CH_{Py}), 9.48 (dt, J = 6.3 and 1.4 Hz, 1H, CH_{Py}), 9.12 (dt, J = 8.1 and 1.4 Hz, 1H, CH_{Py}), 8.36 (dd, J = 8.1 and 6.3 Hz, 1H, CH_{Py}), 6.84 (d, J = 2.3 Hz, 2H, 2 × CH_{Ar}), 6.56 (t, J = 2.3 Hz, 1H, CH_{Ar}), 5.81 (s, 2H, CH_2), 3.76 (s, 6H, 2 × OCH_3).
^{13}C NMR (DMSO-d_6, 101 MHz): δ 160.9 (2 × C), 149.0, 149.0, 148.0, 135.1, 128.8, 113.9, 113.2, 107.5 (2 × C), 101.0, 63.9, 55.5 (2 × C).
LC-HRMS (APCI): m/z [M–Br]$^+$ calcd. for $C_{15}H_{15}N_2O_2^+$: 255.1128; found 255.1101.

***N*-(3,5-Bis(trifluoromethyl)benzyl)-3-cyanopyridinium bromide (1k)**

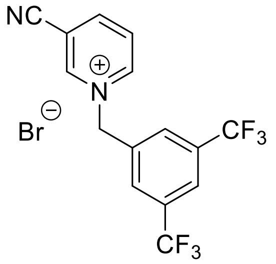

Product **1k** was synthesized following **General procedure A** with 3-cyanopyridine (312.3 mg, 3.0 mmol, 1.0 equiv.) and 3,5-bis(trifluoromethyl)benzyl bromide (0.66 mL, 3.6 mmol, 1.2 equiv.) in MeCN (3.0 mL) to obtain **1k** as a white solid in 1. 81 g (96% yield).
^{1}H NMR (DMSO-d_6, 400 MHz): δ 10.04 (s, 1H, CH_{Py}), 9.56 (dt, J = 6.4 and 1.4 Hz, 1H, CH_{Py}), 9.15 (dt, J = 8.2 and 1.4 Hz, 1H, CH_{Py}), 8.46 (s, 2H, 2 × CH_{Ar}), 8.37 (dd, J = 8.2 and 6.3 Hz, 1H, CH_{Py}), 8.21 (d, J = 1.8 Hz, 1H, CH_{Ar}), 6.12 (s, 2H, CH_2).
^{13}C NMR (DMSO-d_6, 101 MHz): δ 149.7, 149.2, 148.5, 135.8, 131.18 (q, J = 4.7 Hz), 130.9, 130.6, 130.3, 128.9, 127.2, 124.5, 123.6 (m), 121.8, 119.1, 114.0, 113.2, 62.3.
^{19}F NMR (DMSO-d_6, 376 MHz): δ –61.19.
LC-HRMS (APCI): m/z [M–Br]$^+$ calcd. for $C_{15}H_9F_6N_2^+$: 331.0664; found 331.1486.

N-Methyl-3-cyanopyridinium iodide (1l)

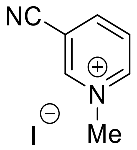

Product **1h** was synthesized following **General procedure B** with 3-cyanopyridine (937.0 mg, 9.0 mmol, 1.0 equiv.) and iodomethane (0.67 mL, 10.8 mmol, 2.0 equiv.) to obtain **1l** as a yellow solid in 2.18 g (99% yield).
^{1}H NMR (DMSO-d_6, 400 MHz): δ 9.74 (s, 1H, CH_{Py}), 9.26 (dt, J = 6.2 and 1.6 Hz, 1H, CH_{Py}), 9.07 (dt, J = 8.2 and 1.6 Hz, 1H, CH_{Py}), 8.34 (dd, J = 8.2 and 6.2 Hz, 1H, CH_{Py}), 4.38 (s, 3H, NCH_3).
^{13}C NMR (DMSO-d_6, 101 MHz): δ 149.7, 149.1, 148.1, 128.0, 113.9, 112.1, 48.7.
LC-HRMS (APCI): m/z [M–I]$^+$ calcd. for $C_7H_7N_2^+$: 119.0604; found 119.0686.

N-Propyl-3-cyanopyridinium bromide (1m)

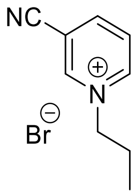

Product **1m** was synthesized following **General procedure B** with 3-cyanopyridine (937.0 mg, 9.0 mmol, 1.0 equiv.) and 1-bromopropane (1.2 mL, 13.5 mmol, 1.5 equiv.) to obtain **1m** as a yellow solid in 1.64 g (80% yield).
^{1}H NMR (DMSO-d_6, 400 MHz): δ 9.87 (s, 1H, CH_{Py}), 9.41 (dt, J = 6.2 and 1.4 Hz, 1H, CH_{Py}), 9.11 (dt, J = 8.1 and 1.4 Hz, 1H, CH_{Py}), 8.38 (dd, J = 8.1 and 6.2 Hz, 1H, CH_{Py}), 4.63 (t, J = 7.4 Hz, 2H, NCH_2), 1.98 (h, J = 7.4 Hz, 2H, 2 × CH_2), 0.90 (t, J = 7.4 Hz, 3H, 3 × CH_3).
^{13}C NMR (DMSO-d_6, 101 MHz): δ 149.0, 148.4, 148.2, 128.4, 114.0, 112.8, 62.8, 23.8, 10.2.
LC-HRMS (APCI): m/z [M–Br]$^+$ calcd. for $C_9H_{11}N_2^+$: 147.0917; found 147.0792.

N-(But-3-en-1-yl)-3-cyanopyridinium bromide (1n)

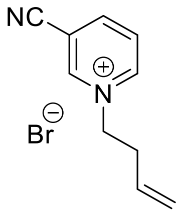

Product **1n** was synthesized following **General procedure B** with 3-cyanopyridine (937.0 mg, 9.0 mmol, 1.0 equiv.) and 4-bromo-1-butene (1.4 mL, 13.5 mmol, 1.5 equiv.) to obtain **1n** as a brown solid in 2.01 g (93% yield).
^{1}H NMR (DMSO-d_6, 400 MHz): δ 9.86 (s, 1H, CH_{Py}), 9.37 (dt, J = 6.2 and 1.4 Hz, 1H, CH_{Py}), 9.11 (dt, J = 8.2 and 1.4 Hz, 1H, CH_{Py}), 8.38 (dd, J = 8.2 and 6.2 Hz, 1H, CH_{Py}), 5.88–5.77 (m, 1H, CH=CH$_2$), 5.09–5.00 (m, 2H, CH=CH_2), 4.76 (t, J = 7.0 Hz, NCH_2), 2.75 (q, J = 7.0 Hz, 2H, CH_2).
^{13}C NMR (DMSO-d_6, 101 MHz): δ 149.0, 148.6, 148.3, 132.7, 128.2, 119.4, 113.9, 112.6, 60.5, 34.3.
LC-HRMS (APCI): m/z [M–Br]$^+$ calcd. for $C_{10}H_{11}N_2^+$: 159.0917; found 159.0790.

N-Benzyl-3-cyanopyridinium bromide (1o)

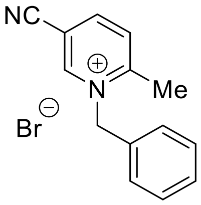

Product **1o** was synthesized following **General procedure A** with 5-methyl-3-cyanopyridine (1.06 g, 9.0 mmol, 1.0 equiv.) and benzyl bromide (1.3 mL, 10.8 mmol, 1.2 equiv.) in MeCN (9.0 mL) to obtain **1o** as a brown solid in 2.51 g (96% yield).

^{1}H NMR (DMSO-*d*$_6$, 400 MHz): δ 9.94 (d, *J* = 1.8 Hz, 1H, CH_{Py}), 9.05 (dd, *J* = 8.3 and 1.8 Hz, 1H, CH_{Py}), 8.31 (d, *J* = 8.3 Hz, 1H, CH_{Py}), 7.47–7.37 (m, 5H, 5 × CH_{Ar}), 5.97 (s, 2H, CH_2), 2.81 (s, 3H, CH_3).

^{13}C NMR (DMSO-*d*$_6$, 101 MHz): δ 160.2, 150.4, 147.9, 132.2, 130.9, 129.2 (2 × C), 129.0, 127.9 (2 × C), 114.1, 111.0, 61.3, 20.6.

LC-HRMS (APCI): m/z [M–Br]$^+$ calcd. for $C_{14}H_{13}N_2^+$: 209.1073; found 209.0820.

N-Benzyl-3-(methoxycarbonyl)pyridinium bromide (1p)

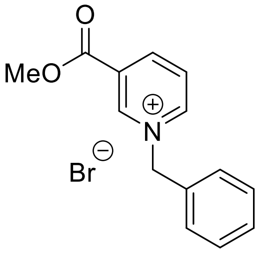

Product **1p** was synthesized following **General procedure A** with methyl nicotinate (1.23 g, 9.0 mmol, 1.0 equiv.) and benzyl bromide (1.3 mL, 10.8 mmol, 1.2 equiv.) in MeCN (9.0 mL) to obtain **1p** as a white solid in 2.55 g (92% yield). NMR data are in agreement with literature precedents.[15]

^{1}H NMR (DMSO-*d*$_6$, 400 MHz): δ 9.80 (s, 1H, CH_{Py}), 9.42 (dt, *J* = 6.2 and 1.4 Hz, 1H, CH_{Py}), 8.01 (dt, *J* = 8.1 and 1.5 Hz, 1H, CH_{Py}), 8.32 (dd, *J* = 8.1 and 6.1 Hz, 1H, CH_{Py}), 7.60–7.57 (m, 2H, 2 × CH_{Ar}), 7.47–7.43 (m, 2H, 2 × CH_{Ar}), 6.02 (s, 2H, CH_2), 3.98 (s, 3H, OCH_3).

^{13}C NMR (DMSO-*d*$_6$, 101 MHz): δ 162.1, 147.9, 145.9, 145.6, 134.0, 130.2, 129.4, 129.2 (2 × C), 128.9 (2 × C), 128.8, 63.4, 53.5.

LC-HRMS (APCI): m/z [M–Br]$^+$ calcd. for $C_{14}H_{14}NO_2^+$: 228.1019; found 228.0993.

N-benzyl-3-cyanoquinolinium bromide (1q)

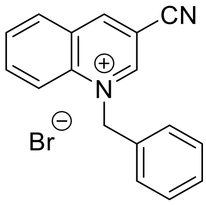

Product **1o** was synthesized following **General procedure A** with quinoline-3-carbonitrile (1.39 g, 9.0 mmol, 1.0 equiv.) and benzyl bromide (1.3 mL, 10.8 mmol, 1.2 equiv.) in MeCN (9.0 mL) to obtain **1q** as a white solid in 2.79 g (95% yield).

^{1}H NMR (DMSO-d_6, 400 MHz): δ 10.53 (d, *J* = 1.8 Hz, 1H, CH_{Ar}), 10.07 (d, *J* = 0.8 Hz, 1H, CH_{Ar}), 8.59–8.56 (m, 2H, 2 × CH_{Ar}), 8.38–8.34 (m, 1H, CH_{Ar}), 8.15–8.11 (m, 1H, CH_{Ar}), 7.55–7.53 (m, 2H, 2 × CH_{Ar}), 7.41–7.33 (m, 3H, 3 × CH_{Ar}), 6.47 (s, 2H, CH_2).
^{13}C NMR (DMSO-d_6, 101 MHz): δ 152.6, 152.6, 138.7, 137.9, 133.3, 131.9, 131.3, 129.0 (2 × C), 128.9, 128.8, 127.6 (2 × C), 119.8, 114.6, 107.2, 60.7.
LC-HRMS (APCI): m/z $[M–Br]^+$ calcd. for $C_{14}H_{13}N_2^+$: 245.1073; found 245.0822.

***N*-(4-Cyanobenzyl)-3-cyanopyridinium bromide (1r)**

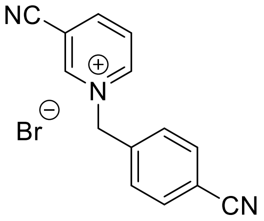

Product **1r** was synthesized following **General procedure A** with 3-cyanopyridine (937.0 mg, 9.0 mmol, 1.0 equiv.) and 4-cyanobenzyl bromide (2.12 g, 10.8 mmol, 1.2 equiv.) in MeCN (9.0 mL) to obtain **1r** as a white solid in 1.90 g (70% yield).
^{1}H NMR (DMSO-d_6, 400 MHz): δ 10.01 (t, *J* = 1.6 Hz, 1H, CH_{Py}), 9.47 (dt, *J* = 6.3 and 1.4 Hz, 1H, CH_{Py}), 9.15 (dt, *J* = 8.2 and 1.4 Hz, 1H, CH_{Py}), 8.40 (dd, *J* = 8.2 and 6.3 Hz, 1H, CH_{Py}), 7.97–7.94 (m, 2H, 2 × CH_{Ar}), 7.79–7.77 (m, 2H, 2 × CH_{Ar}), 6.03 (s, 2H, CH_2).
^{13}C NMR (DMSO-d_6, 101 MHz): δ 149.5, 149.2, 148.4, 138.4, 132.9 (2 × C), 130.1 (2 × C), 128.9, 118.3, 113.9, 113.4, 112.2, 63.1.
LC-HRMS (APCI): m/z $[M–Br]^+$ calcd. for $C_{14}H_{10}N_3^+$: 220.0869; found 220.1054.

***N*-(4-Nitrobenzyl)-3-cyanopyridinium bromide (1s)**

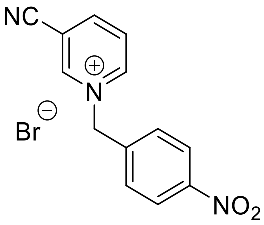

Product **1s** was synthesized following **General procedure A** with 3-cyanopyridine (937.0 mg, 9.0 mmol, 1.0 equiv.) and 4-nitrobenzyl bromide (2.33 g, 10.8 mmol, 1.2 equiv.) in MeCN (9.0 mL) to obtain **1s** as a white solid in 2.74 g (95% yield).
^{1}H NMR (DMSO-d_6, 400 MHz): δ 10.08 (s, 1H, CH_{Py}), 9.55 (dt, *J* = 6.3 and 1.4 Hz, 1H, CH_{Py}), 9.17 (dt, *J* = 8.2 and 1.4 Hz, 1H, CH_{Py}), 8.43 (dd, *J* = 8.2 and 6.3 Hz, 1H, CH_{Py}), 8.29 (d, *J* = 8.8 Hz, 2H, 2 × CH_{Ar}), 7.89 (d, *J* = 8.8 Hz, 2H, 2 × CH_{Ar}), 6.14 (s, 2H, CH_2).
^{13}C NMR (DMSO-d_6, 101 MHz): δ 149.6, 149.2, 148.4, 148.0, 140.2, 130.7, 128.9, 124.0, 113.9, 113.3, 62.6.
LC-HRMS (APCI): m/z $[M–Br]^+$ calcd. for $C_{13}H_{10}N_3O_2^+$: 240.0768; found 240.0877.

***N*-Allyl-3-cyanopyridinium bromide (1t)**

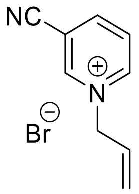

Product **1t** was synthesized following **General procedure A** with 3-cyanopyridine (937.0 mg, 9.0 mmol, 1.0 equiv.) and allyl bromide (0.9 mL, 10.8 mmol, 1.2 equiv.) to obtain **1t** as a brown solid in 1.53 g (76% yield).

^{1}H NMR (DMSO-*d*$_6$, 400 MHz): δ 9.84 (s, 1H, CH_{Py}), 9.34 (dt, J = 6.2 and 1.4 Hz, 1H, CH_{Py}), 9.13 (dt, J = 8.2 and 1.4 Hz, 1H, CH_{Py}), 8.39 (dd, J = 8.2 and 6.2 Hz, 1H, CH_{Py}), 6.22–6.12 (m, 1H, C*H*=CH$_2$), 5.51–5.48 (m, 2H, CH=CH_2), 4.76 (d, J = 6.3 Hz, 2H, NCH_2).

^{13}C NMR (DMSO-*d*$_6$, 101 MHz): δ 149.1, 148.9, 148.2, 130.8, 128.6, 123.0, 113.9, 112.9, 63.1.

LC-HRMS (APCI): m/z [M–Br]$^+$ calcd. for $C_9H_9N_2^+$: 145.0760; found 145.0761.

5. Cu(I)-catalysed nucleophilic 1,4-dearomatization of pyridiniums

5.1 General procedure for asymmetric dearomatization

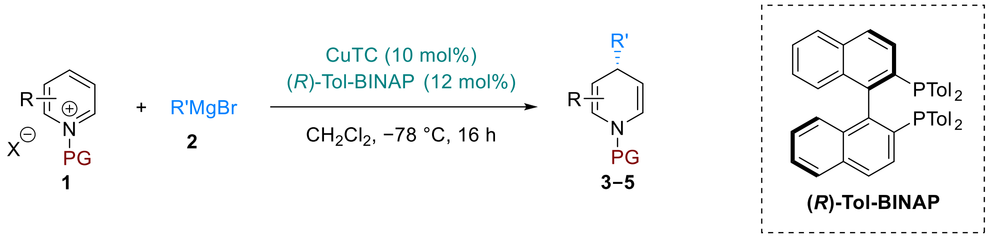

5.1.1 General procedure for the enantioselective Cu(I)-catalysed dearomatization of pyridinium salts

In a flame-dried Schlenk tube equipped with a septum and a magnetic stirring bar, the pyridinium substrate (0.2 mmol, 1.0 equiv.), CuTC (10.0 mol%) and (*R*)-Tol-BINAP (12.0 mol%) were dissolved in dry CH_2Cl_2 (1.0 mL) and stirred under N_2 atmosphere for 30 min at room temperature. The mixture was cooled to −78 °C and the Grignard reagent (0.24 mmol, 1.2 equiv.) was added dropwise. After stirring for 16 h, at −78 °C, the resulting reaction mixture was quenched with saturated NH_4Cl (2 mL) and left stirring for 2 h. The reaction mixture was extracted with CH_2Cl_2 (3 x 5 mL). The combined organic layers were dried over anhydrous $MgSO_4$, filtered, and concentrated under reduced pressure in a rotatory evaporator. The product mixture was purified by flash column chromatography.

5.1.2 General procedure for the synthesis of racemic 1,4-dihydropyridines

In a flame-dried Schlenk tube equipped with a septum and a magnetic stirring bar, the substrate (0.2 mmol, 1.0 equiv.) and CuTC (10.0 mol%) in dry CH_2Cl_2 (1.0 mL) were stirred under N_2 atmosphere for 30 min at room temperature. The mixture was cooled to −78 °C and the Grignard reagent (0.4 mmol, 2.0 equiv.) was added dropwise. After stirring for 16 h, at −78 °C, the resulting reaction mixture was quenched with saturated NH_4Cl (2 mL) and left stirring for 2 h. The reaction mixture was extracted with CH_2Cl_2 (3 × 5 mL). The combined organic layers were dried over anhydrous $MgSO_4$, filtered, and concentrated under reduced pressure in a rotatory evaporator. The product mixture was purified by flash column chromatography.

5.2 Optimization of the reaction conditions

The selection of the ligand set to study as well as the choice of the initial reaction conditions was done in analogy with previous reports from our group.[16]

Table S1. Optimization of the ligand for the enantioselective addition of EtMgBr to pyridinium salt **1a**.[a]

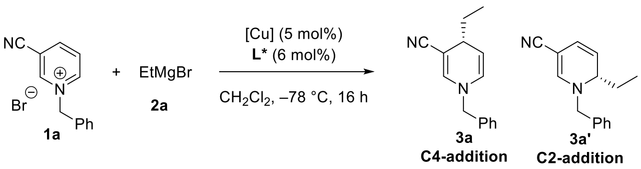

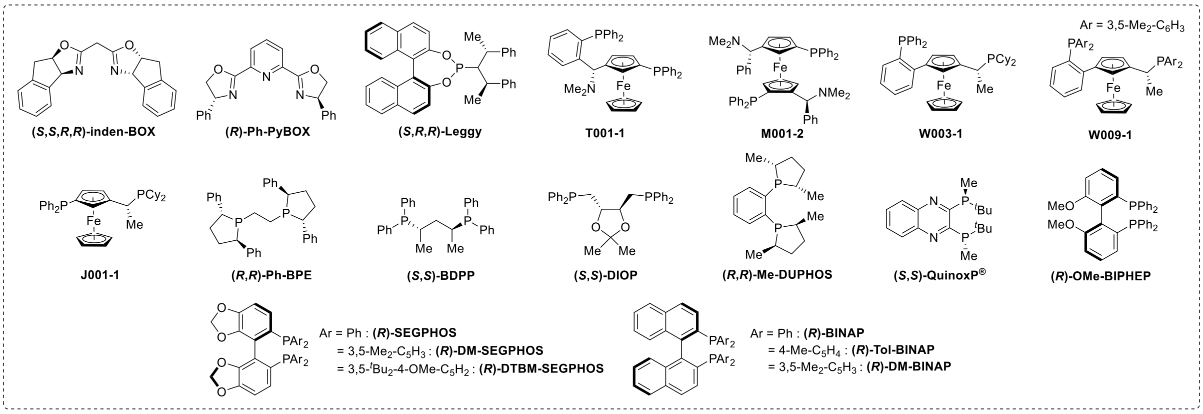

Entry	Cu salt	Ligand	Solvent	Yield (%) [b]	3a: 3a' [b]	*ee* (%) [c] 3a	*ee* (%) [c] 3a'
1	-	-	CH_2Cl_2	46	57:43	-	-
2	CuBr·SMe2	-	CH_2Cl_2	82	73:27	-	-
3	CuBr·SMe2	(*S*,*S*,*R*,*R*)-indenBOX	CH_2Cl_2	>99	63:37	1	-2
4	CuBr·SMe2	(*R*)-Ph-PyBOX	CH_2Cl_2	>99	64:36	4	-2
5	CuBr·SMe2	(*S*,*R*,*R*)-Leggy	CH_2Cl_2	96	76:24	3	3
6	CuBr·SMe2	T001-1	CH_2Cl_2	>99	64:36	3	-3
7	CuBr·SMe2	M001-2	CH_2Cl_2	>99	58:42	5	0
8	CuBr·SMe2	W003-1	CH_2Cl_2	50	46:54	2	-2
9	CuBr·SMe2	W009-1	CH_2Cl_2	85	55:45	6	2
10	CuBr·SMe2	J001-1	CH_2Cl_2	>99	80:20	-4	7
11	CuBr·SMe2	(*S*,*S*)-Ph-BPE	CH_2Cl_2	>99	66:34	1	16
12	CuBr·SMe2	(*S*,*S*)-BDPP	CH_2Cl_2	84	76:24	-4	0
13	CuBr·SMe2	(*S*,*S*)-DIOP	CH_2Cl_2	>99	71:29	-7	-3
14	CuBr·SMe2	(*R*,*R*)-Me-DUPHOS	CH_2Cl_2	>99	63:37	4	4
15	CuBr·SMe2	*(R)*-QuinoxP®	CH_2Cl_2	97	55:45	2	-2
16	CuBr·SMe2	(*R*)-OMe-BIPHEP	CH_2Cl_2	>99	84:16	74	-1
17	CuBr·SMe2	(*R*)-SEGPHOS	CH_2Cl_2	>99	88:12	75	1
18	CuBr·SMe2	(*R*)-DM-SEGPHOS	CH_2Cl_2	>99	81:19	35	19
19	CuBr·SMe2	(*R*)-DTBM-SEGPHOS	CH_2Cl_2	>99	60:40	3	3
20	CuBr·SMe2	(*R*)-Tol-BINAP	CH_2Cl_2	>99	85:15	78	2
21	CuBr·SMe2	(*R*)-DM-BINAP	CH_2Cl_2	>99	79:21	47	2
22	CuBr·SMe2	(*R*)- BINAP	CH_2Cl_2	>99	81:19	70	-1

[a] **Reaction conditions:** 3-cyanopyridinium salt (0.2 mmol, 1.0 equiv.), Cu salt (5 mol%), chiral ligand (6 mol%), EtMgBr in Et_2O (3 M; 0.24 mmol, 1.2 equiv.) in solvent (2.0 mL) for 16 h. [b] The yields of **3a** and **3a'** were determined by analysis of ^1H NMR spectra of the reaction crude using 1,3,5-trimethoxybenzene as an internal standard. [c] Enantiomeric excess (*ee*) was determined by SFC on a chiral stationary phase.

Table S2. Optimization of copper salt for the enantioselective addition of EtMgBr to **1a**. [a]

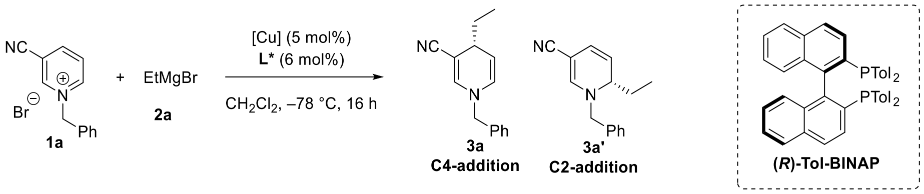

Entry	Cu salt	Ligand	Solvent	Yield (%) [b]	3a: 3a' [b]	*ee* (%) [c]	
						3a	3a'
1	$CuBr·SMe_2$	(*R*)-Tol-BINAP	CH_2Cl_2	>99	85:15	78	2
2	CuCl	(*R*)-Tol-BINAP	CH_2Cl_2	>99	84:16	79	3
3	CuBr	(*R*)-Tol-BINAP	CH_2Cl_2	>99	87:13	77	2
4	CuI	(*R*)-Tol-BINAP	CH_2Cl_2	>99	67:32	53	0
5	$Cu(MeCN)_4PF_6$	(*R*)-Tol-BINAP	CH_2Cl_2	>99	82:18	70	-1
6	$(CuOTf)_2·Tol$	(*R*)-Tol-BINAP	CH_2Cl_2	>99	86:14	64	1
7	CuTC	(*R*)-Tol-BINAP	CH_2Cl_2	>99	87:13	78	0
8	$Cu(OTf)_2$	(*R*)-Tol-BINAP	CH_2Cl_2	>99	81:19	75	0
9	$Cu(OAc)_2$	(*R*)-Tol-BINAP	CH_2Cl_2	>99	80:20	75	2

[a] **Reaction conditions:** 3-cyanopyridinium salt (0.2 mmol, 1.0 equiv.), Cu salt (5 mol%), chiral ligand (6 mol%), EtMgBr in Et_2O (3 M; 0.24 mmol, 1.2 equiv.) in solvent (2.0 mL) for 16 h. [b] The yields of **3a** and **3a'** were determined by analysis of ^1H NMR spectra of the reaction crude using 1,3,5-trimethoxybenzene as an internal standard. [c] Enantiomeric excess (*ee*) was determined by SFC on a chiral stationary phase.

Table S3. Optimization of solvent and catalyst loading for the enantioselective addition of EtMgBr to **1a**. [a]

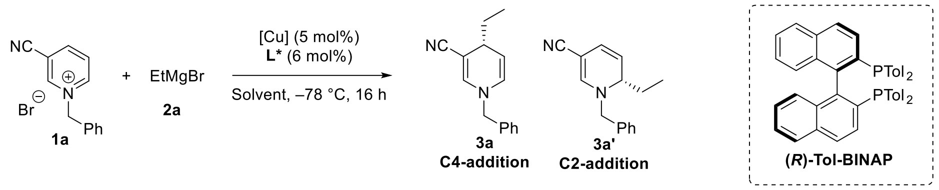

Entry	Cu salt	Ligand	Solvent	Yield (%) [b]	3a: 3a' [b]	ee (%) [c]	
						3a	3a'
1	$CuBr{\cdot}SMe_2$	(*R*)-Tol-BINAP	CH_2Cl_2	>99	85:15	78	2
2	$CuBr{\cdot}SMe_2$	(*R*)-Tol-BINAP	Et_2O	30	50:50	5	0
3	$CuBr{\cdot}SMe_2$	(*R*)-Tol-BINAP	MTBE	25	48:52	6	3
4	$CuBr{\cdot}SMe_2$	(*R*)-Tol-BINAP	Toluene	>99	55:45	4	-2
5	$CuBr{\cdot}SMe_2$	(*R*)-Tol-BINAP	2-Me-THF	86	69:31	1	2
6	$CuBr{\cdot}SMe_2$	(*R*)-Tol-BINAP	THF	83	72:28	1	1
7	$CuBr{\cdot}SMe_2$	(*R*)-Tol-BINAP	CH_2Cl_2:Tol (3:1)	>99	84:16	78	-2
8 [d]	CuCl	(*R*)-Tol-BINAP	CH_2Cl_2	>99	85:15	80	0
9 [d]	$Cu(OTf)_2$	(*R*)-Tol-BINAP	CH_2Cl_2	>99	83:17	81	3
10 [d]	CuTC	(*R*)-Tol-BINAP	CH_2Cl_2	>99	90:10	85	0

[a] **Reaction conditions:** 3-cyanopyridinium salt (0.2 mmol, 1.0 equiv.), Cu salt (5 mol%), chiral ligand (6 mol%), EtMgBr in Et_2O (3 M; 0.24 mmol, 1.2 equiv.) in solvent (2.0 mL) for 16 h. [b] The yields of **3a** and **3a'** were determined by analysis of ^1H NMR spectra of the reaction crude using 1,3,5-trimethoxybenzene as an internal standard. [c] Enantiomeric excess (*ee*) was determined by SFC on a chiral stationary phase. [d] In this case Cu salt (10 mol%) and (*R*)-Tol-BINAP (12 mol%) were used.

Incompatible pyridiniums*; no dearomative products was observed.*

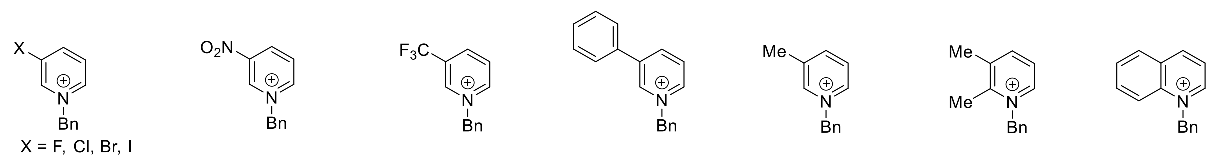

5.3 Specific experimental details and product characterization

(*R*)-1-Benzyl-4-ethyl-1,4-dihydropyridine-3-carbonitrile (3a)

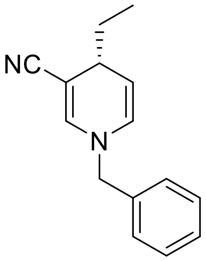

The reaction was performed with **1a** (55.0 mg, 0.2 mmol, 1.0 equiv.), CuTC (3.8 mg, 10.0 mol%), (*R*)-Tol-BINAP (16.3 mg, 12.0 mol%), EtMgBr (3.0 M in Et_2O, 80 μL, 0.24 mmol, 1.2 equiv.) in CH_2Cl_2 (2.0 mL) at −78 °C for 16 h. Product **3a** was obtained as a yellow oil after column chromatography (SiO_2, pentane:EtOAc = 3:1) [>99% conversion, 40.1 mg, 89% yield, 85% *ee*, (*R*)-configuration].

^{1}H NMR ($CDCl_3$, 400 MHz): δ 7.36–7.29 (m, 3H, 3 × C*H*$_{Ar}$), 7.21–7.17 (m, 2H, 2 × C*H*$_{Ar}$), 6.63 (d, *J* = 1.5 Hz, 1H, C=C*H*), 5.81 (dt, *J* = 8.2 and 1.5 Hz, 1H, C*H*=CH), 4.62 (dd, *J* = 8.2 and 4.1 Hz, 1H, CH=C*H*), 4.28 (s, 2H, NC*H*$_2$), 3.24–3.21 (m, , 1H, C*H*), 1.65–1.54 (m, 1H, C*H*H), 1.53–1.44 (m, 1H, CH*H*), 0.95 (t, *J* = 7.5 Hz, 3H, CH_3).

^{13}C NMR ($CDCl_3$, 101 MHz): δ 143.2, 136.6, 129.1 (2 × C), 128.4, 128.2, 127.2 (2 × C), 121.5, 106.0, 82.5, 57.5, 34.2, 30.1, 9.2.

LC-HRMS (ESI-TOF): m/z $[M+H]^+$ calcd. for $C_{15}H_{16}N_2H^+$: 225.1386; found 225.1381.

SFC: Trefoil CEL2, CO_2/MeOH with gradient from 97% to 90% in 10 min, 1.8 mL/min., 40 °C, detection at 324 nm. Retention time (min.): 3.83 (minor) and 4.01 (major).

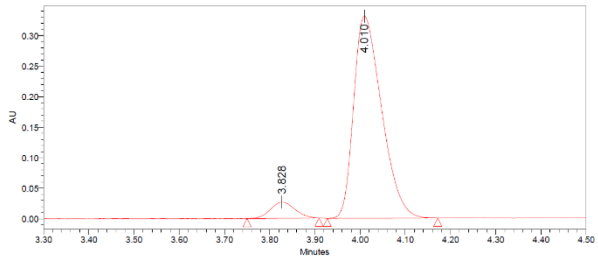

Peak Results

	Name	RT	Area	% Area	Height
1		3.828	99397	6.53	26364
2		4.010	1423522	93.47	330992

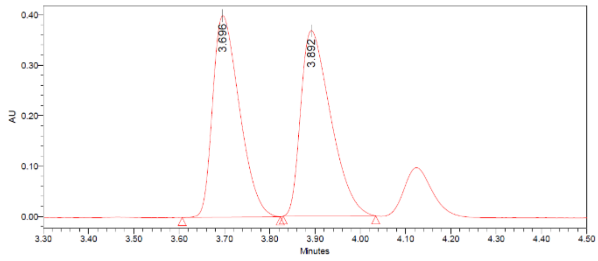

Peak Results

	Name	RT	Area	% Area	Height
1		3.696	1640502	49.21	399540
2		3.892	1693301	50.79	367579

SFC of (*R*)-1-benzyl-4-ethyl-1,4-dihydropyridine-3-carbonitrile (**3a**)

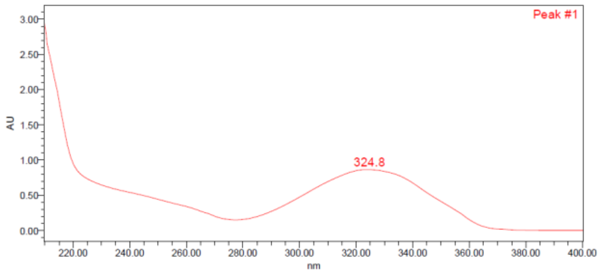

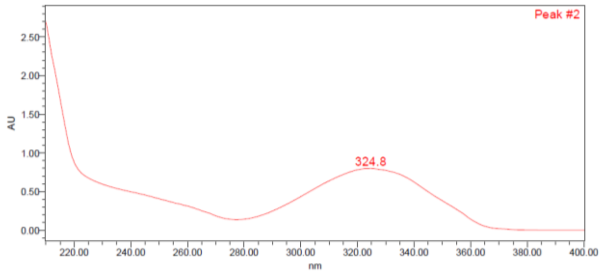

UV-visible spectra of (*R*)-1-benzyl-4-ethyl-1,4-dihydropyridine-3-carbonitrile (**3a**)

(*S*)-1-Benzyl-6-ethyl-1,6-dihydropyridine-3-carbonitrile (3a’)

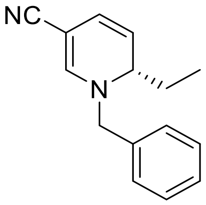

Product **3a’** was obtained as a yellow oil after column chromatography (SiO_2, pentane:EtOAc = 3:1) [>99% conversion, 3.5 mg, 8% yield].

^{1}H NMR ($CDCl_3$, 400 MHz): δ 7.41–7.32 (m, 3H, 3 × CH_{Ar}), 7.27–7.24 (m, 2H, 2 × CH_{Ar}), 6.87 (d, *J* = 1.1 Hz, 1H, C=C*H*), 5.93 (dd, *J* = 9.7 and 1.4Hz, 1H, C*H*=CH), 4.94 (dd, *J* = 9.7 and 5.0Hz, 1H, CH=C*H*), 4.37–4.27 (m, 2H, NCH_2), 4.05–4.01 (m, 1H, C*H*), 1.76 (dt, *J* = 13.8 and 7.3 Hz, 1H, CH*H*), 1.45–1.35 (m, 1H, C*H*H), 0.94 (t, *J* = 7.5 Hz, 3H, CH_3).

^{13}C NMR ($CDCl_3$, 101 MHz): δ 148.1, 135.6, 129.2 (2 × C), 128.5, 127.7 (2 × C), 121.8, 121.7, 113.5, 77.4, 57.8, 57.3, 27.0, 7.8.

LC-HRMS (ESI-TOF): m/z $[M+H]^+$ calcd. for $C_{15}H_{16}N_2H^+$: 225.1386; found 225.1383.

SFC: Trefoil CEL2, CO_2/MeOH with gradient from 97% to 40% in 4.5 min, 1.8 mL/min., 40 °C, detection at 324 nm. Retention time (min.): 2.13 (major) and 2.18 (minor).

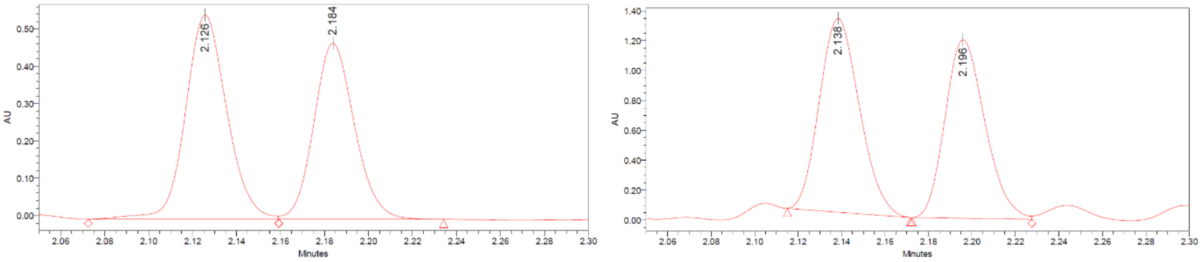

Peak Results

	Name	RT	Area	% Area	Height
1		2.126	716853	54.20	547537
2		2.184	605711	45.80	471961

Peak Results

	Name	RT	Area	% Area	Height
1		2.138	1665937	52.20	1299147
2		2.196	1525439	47.80	1196246

SFC of (*S*)-1-benzyl-6-ethyl-1,6-dihydropyridine-3-carbonitrile (**3a’**)

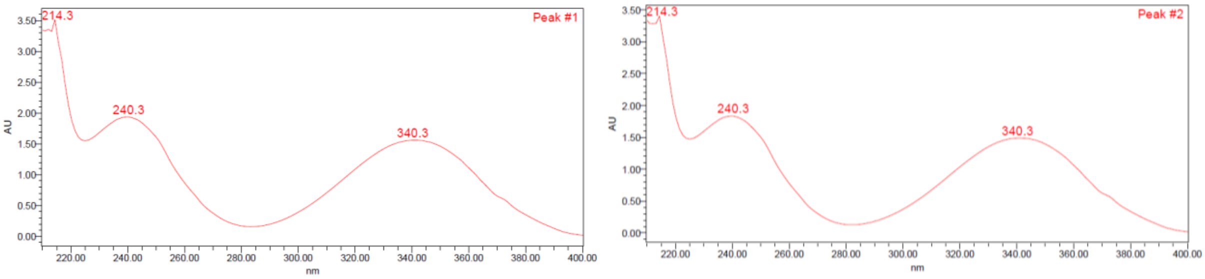

UV-visible spectra of (*S*)-1-benzyl-6-ethyl-1,6-dihydropyridine-3-carbonitrile (**3a’**)

(*R*)-4-Ethyl-1-(4-methylbenzyl)-1,4-dihydropyridine-3-carbonitrile (3b)

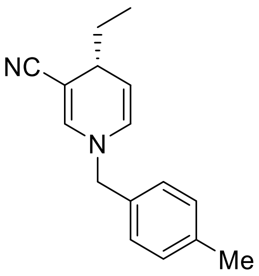

The reaction was performed with **1b** (68.6 mg, 0.2 mmol, 1.0 equiv.), CuTC (3.8 mg, 10.0 mol%), (*R*)-Tol-BINAP (16.3 mg, 12.0 mol%), EtMgBr (3.0 M in Et_2O, 80 µL, 0.24 mmol, 1.2 equiv.) in CH_2Cl_2 (2.0 mL) at −78 °C for 16 h. Product **3b** was obtained as a yellow oil after column chromatography (SiO_2, pentane:EtOAc = 3:1) [>99% conversion, 37.1 mg, 78% yield, 86% *ee*, (*R*)-configuration].

^{1}H NMR ($CDCl_3$, 400 MHz): δ 7.17 (d, *J* = 7.8 Hz, 2H, 2 × C*H*$_{Ar}$), 7.08 (d, *J* = 7.8 Hz, 2H, 2 × C*H*$_{Ar}$), 6.63 (d, *J* = 0.7 Hz, 1H, C=C*H*), 5.80 (d, *J* = 8.0 Hz, 1H, C*H*=CH), 4.61 (dd, *J* = 8.0 and 3.8 Hz, 1H, CH=C*H*), 4.23 (s, 2H, NC*H*$_2$), 3.24–3.21 (m, 1H, C*H*), 2.35 (s, 3H, C*H*$_3$), 1.64–1.43 (m, 2H, C*HH*), 0.95 (t, *J* = 7.4 Hz, 3H, C*H*$_3$).

^{13}C NMR ($CDCl_3$, 101 MHz): δ 143.2, 138.1, 133.5, 129.8 (2 × C), 128.4, 127.3 (2 × C), 121.6, 105.9, 82.3, 57.3, 34.2, 30.1, 21.2, 9.2.

LC-HRMS (ESI-TOF): m/z $[M+H]^+$ calcd. for $C_{16}H_{18}N_2H^+$: 239.1543; found 239.1540.

SFC: Trefoil CEL2, CO_2/MeOH with gradient from 97% to 90% in 10 min, 1.8 mL/min., 40 °C, detection at 324 nm. Retention time (min.): 3.82 (minor) and 4.02 (major).

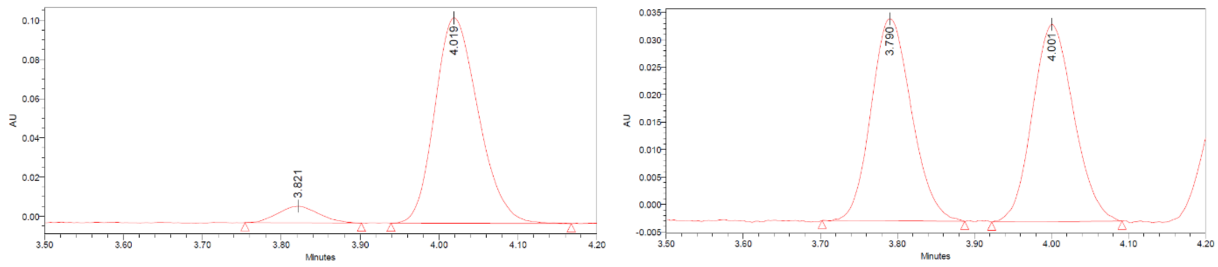

Peak Results

	Name	RT	Area	% Area	Height
1		3.821	30572	7.07	8465
2		4.019	401686	92.93	104752

Peak Results

	Name	RT	Area	% Area	Height
1		3.790	134658	50.10	36829
2		4.001	134147	49.90	35945

SFC of (*R*)-4-ethyl-1-(4-methylbenzyl)-1,4-dihydropyridine-3-carbonitrile (**3b**)

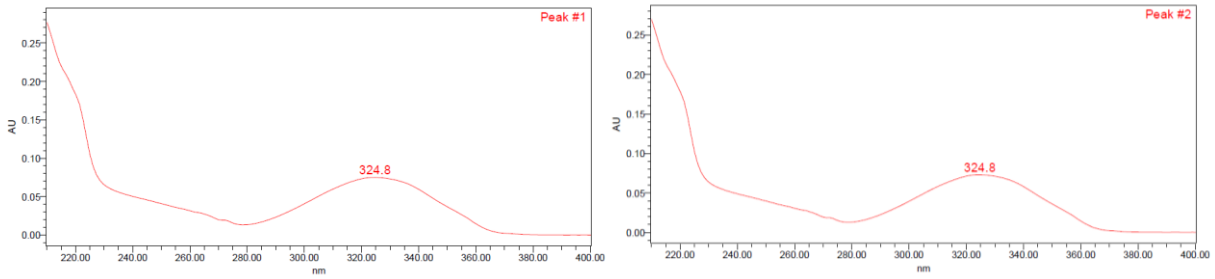

UV-visible spectra of (*R*)-4-ethyl-1-(4-methylbenzyl)-1,4-dihydropyridine-3-carbonitrile (**3b**)

(*R*)-1-(4-(*Tert*-butyl)benzyl)-4-ethyl-1,4-dihydropyridine-3-carbonitrile (3c)

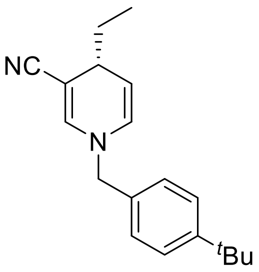

The reaction was performed with **1c** (66.3 mg, 0.2 mmol, 1.0 equiv.), CuTC (3.8 mg, 10.0 mol%), (*R*)-Tol-BINAP (16.3 mg, 12.0 mol%), EtMgBr (3.0 M in Et_2O, 80 μL, 0.24 mmol, 1.2 equiv.) in CH_2Cl_2 (2.0 mL) at −78 °C for 16 h. Product **3c** was obtained as a yellow oil after column chromatography (SiO_2, pentane:EtOAc = 3:1) [>99% conversion, 49.0 mg, 87% yield, 85% *ee*, (*R*)-configuration].

^{1}H NMR ($CDCl_3$, 400 MHz): δ 7.39 (d, *J* = 8.3 Hz, 2H, 2 × CH_{Ar}), 7.12 (d, *J* = 8.3 Hz, 2H, 2 × CH_{Ar}), 6.62 (d, *J* = 1.6 Hz, 1H, C=C*H*), 5.81 (d, *J* = 8.2 Hz, 1H, C*H*=CH), 4.62 (dd, *J* = 8.2 and 4.1 Hz, 1H, CH=C*H*), 4.25 (s, 2H, NCH_2), 3.25–3.21 (m, 1H, C*H*), 1.65–1.54 (m, 1H, C*H*H), 1.53–1.44 (m, 1H, CH*H*), 1.32 (s, 9H, 3 × CH_3), 0.96 (t, *J* = 7.5 Hz, 3H, CH_3).

^{13}C NMR ($CDCl_3$, 101 MHz): δ 151.3, 143.3, 133.6, 128.6, 127.0 (2 × C), 126.0 (2 × C), 121.6, 105.8, 82.4, 57.2, 34.7, 34.3, 31.4 (3 × C), 30.2, 9.3.

LC-HRMS (ESI-TOF): m/z $[M+H]^+$ calcd. for $C_{19}H_{24}N_2H^+$: 281.2013; found 281.1362.

SFC: Trefoil CEL2, CO_2/MeOH with gradient from 97% to 90% in 10 min, 1.8 mL/min., 40 °C, detection at 324 nm. Retention time (min.): 2.56 (minor) and 2.65 (major).

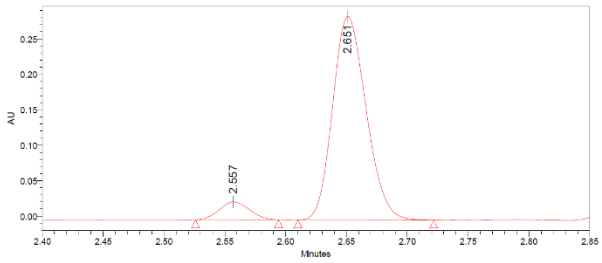

Peak Results

	Name	RT	Area	% Area	Height
1		2.557	43230	7.30	25206
2		2.651	548967	92.70	286944

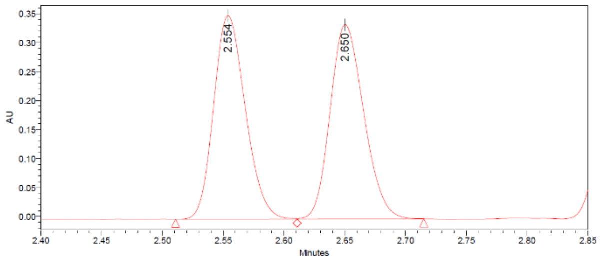

Peak Results

	Name	RT	Area	% Area	Height
1		2.554	636636	49.68	351569
2		2.650	644763	50.32	335632

SFC of (*R*)-1-(4-(*tert*-butyl)benzyl)-4-ethyl-1,4-dihydropyridine-3-carbonitrile (**3c**)

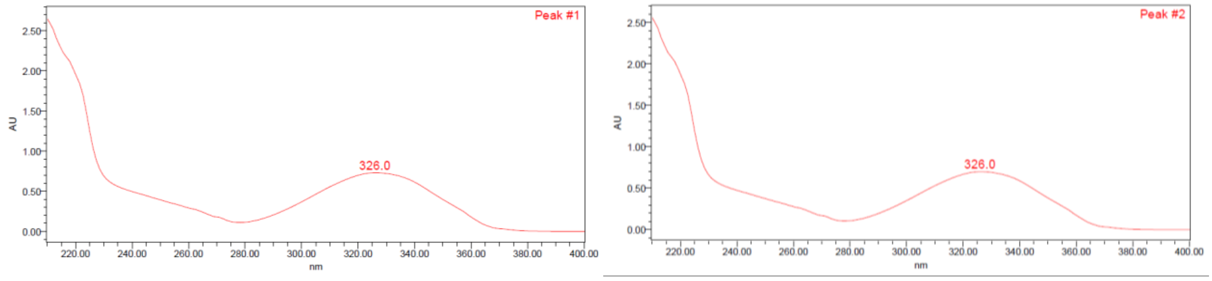

UV-visible spectra of (*R*)-1-(4-(*tert*-butyl)benzyl)-4-ethyl-1,4-dihydropyridine-3-carbonitrile (**3c**)

(*R*)-4-Ethyl-1-(4-(trifluoromethyl)benzyl)-1,4-dihydropyridine-3-carbonitrile (3d)

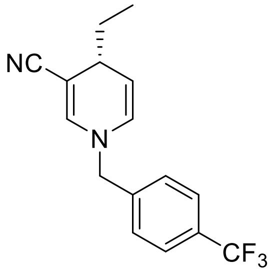

The reaction was performed with **1d** (68.6 mg, 0.2 mmol, 1.0 equiv.), CuTC (3.8 mg, 10.0 mol%), (*R*)-Tol-BINAP (16.3 mg, 12.0 mol%), EtMgBr (3.0 M in Et_2O, 80 μL, 0.24 mmol, 1.2 equiv.) in CH_2Cl_2 (2.0 mL) at −78 °C for 16 h. Product **3d** was obtained as a yellow oil after column chromatography (SiO_2, pentane:EtOAc = 3:1) [>99% conversion, 47.9 mg, 82% yield, 73% *ee*, (*R*)-configuration].

^{1}H NMR ($CDCl_3$, 400 MHz): δ 7.64 (d, J = 8.0 Hz, 2H, 2 × C*H*$_{Ar}$), 7.32 (d, *J* = 8.0 Hz, 2H, 2 × C*H*$_{Ar}$), 6.63 (d, *J* = 1.7 Hz, 1H, C=C*H*), 5.78 (dt, *J* = 8.1 and 1.4 Hz, 1H, C*H*=CH), 4.66 (dd, *J* = 8.1 and 4.1 Hz, 1H, CH=C*H*), 4.35 (s, 2H, NC*H*$_2$), 3.27–3.23 (m, 1H, C*H*), 1.61–1.56 (m, 1H, C*H*H), 1.55–1.45 (m, 1H, CH*H*), 0.97 (t, *J* = 7.5 Hz, 3H, C*H*$_3$).

^{13}C NMR ($CDCl_3$, 101 MHz): δ 142.8, 140.6, 128.0 (2 × C), 127.2 (2 × C), 126.0 (q, J = 3.8 Hz), 120.9, 118.2, 106.3, 83.4, 56.8, 34.0, 29.9, 29.7, 9.1.

^{19}F NMR ($CDCl_3$, 376 MHz): δ −62.68.

LC-HRMS (ESI-TOF): m/z $[M+H]^+$ calcd. for $C_{16}H_{15}F_3N_2H^+$: 293.1260; found 293.1359.

SFC: Trefoil CEL2, CO_2/MeOH with gradient from 97% to 90% in 10 min, 1.8 mL/min., 40 °C, detection at 324 nm. Retention time (min.): 2.20 (minor) and 2.27 (major).

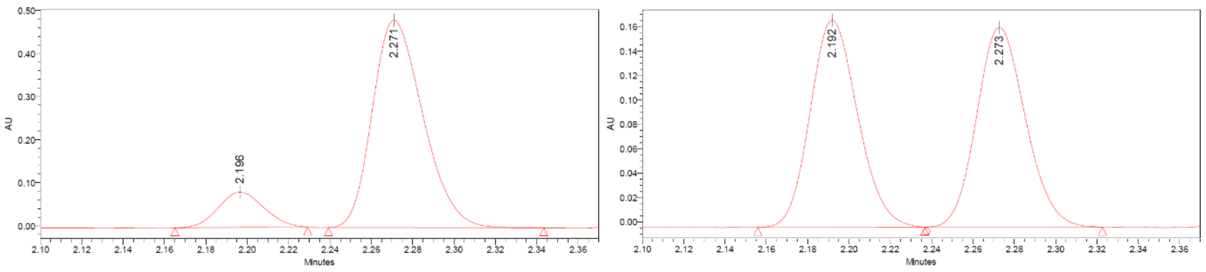

Peak Results

	Name	RT	Area	% Area	Height
1		2.196	125092	13.27	81995
2		2.271	817546	86.73	481819

Peak Results

	Name	RT	Area	% Area	Height
1		2.192	270893	50.13	169687
2		2.273	269519	49.87	163671

SFC of (*R*)-4-ethyl-1-(4-(trifluoromethyl)benzyl)-1,4-dihydropyridine-3-carbonitrile (**3d**)

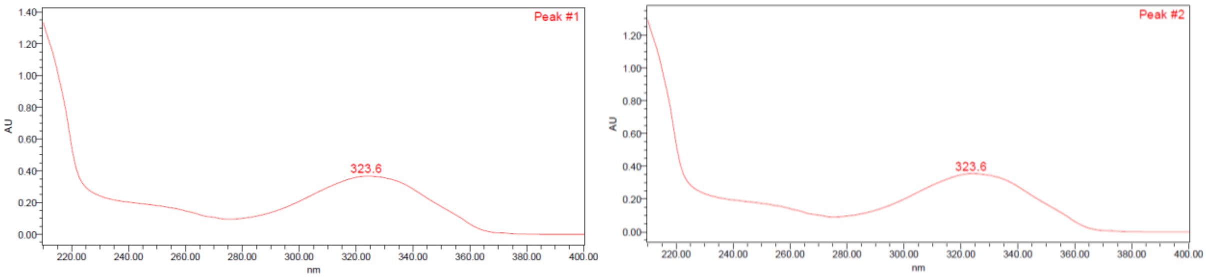

UV-visible spectra of (*R*)-4-ethyl-1-(4-(trifluoromethyl)benzyl)-1,4-dihydropyridine-3-carbonitrile (**3d**)

(*R*)-4-Ethyl-1-(4-fluorobenzyl)-1,4-dihydropyridine-3-carbonitrile (3e)

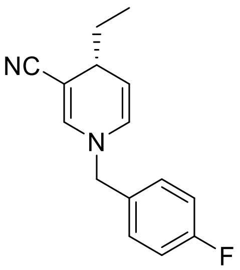

The reaction was performed with **1e** (68.6 mg, 0.2 mmol, 1.0 equiv.), CuTC (3.8 mg, 10.0 mol%), (*R*)-Tol-BINAP (16.3 mg, 12.0 mol%), EtMgBr (3.0 M in Et_2O, 80 µL, 0.24 mmol, 1.2 equiv.) in CH_2Cl_2 (2.0 mL) at −78 °C for 16 h. Product **3e** was obtained as a yellow oil after column chromatography (SiO_2, pentane:EtOAc = 3:1) [>99% conversion, 36.2 mg, 75% yield, 59% *ee*, (*R*)-configuration].

^{1}H NMR ($CDCl_3$, 400 MHz): δ 7.16 (dt, *J* = 6.2 and 3.7 Hz, 2H, 2 × CH_{Ar}), 7.06 (td, *J* = 8.6 and 1.3 Hz, 2H, 2 × CH_{Ar}), 6.62 (s, 1H, C=C*H*), 5.78 (d, *J* = 8.1 Hz, 1H, C*H*=CH), 4.66 (dd, *J* = 8.1 and 4.1 Hz, 1H, CH=C*H*), 4.25 (s, 2H, NCH_2), 3.24–3.21 (m, 1H, C*H*), 1.64–1.43 (m, 2H, C*HH*), 0.94 (t, *J* = 7.4 Hz, 3H, CH_3).

^{13}C NMR ($CDCl_3$, 101 MHz): δ 163.9, 161.4, 143.0, 132.4, 132.3, 129.0, 128.9, 128.2, 121.4, 116.2, 116.0, 106.2, 82.9, 56.8, 34.2, 30.1, 9.2.

^{19}F NMR ($CDCl_3$, 376 MHz): δ −113.88 (td, *J* = 8.4 and 4.3 Hz).

LC-HRMS (ESI-TOF): m/z $[M+H]^+$ calcd. for $C_{15}H_{15}FN_2H^+$: 243.1292; found 243.1290.

SFC: Trefoil CEL2, CO_2/MeOH with gradient from 97% to 90% in 10 min, 1.8 mL/min., 40 °C, detection at 324 nm. Retention time (min.): 3.69 (minor) and 3.90 (major).

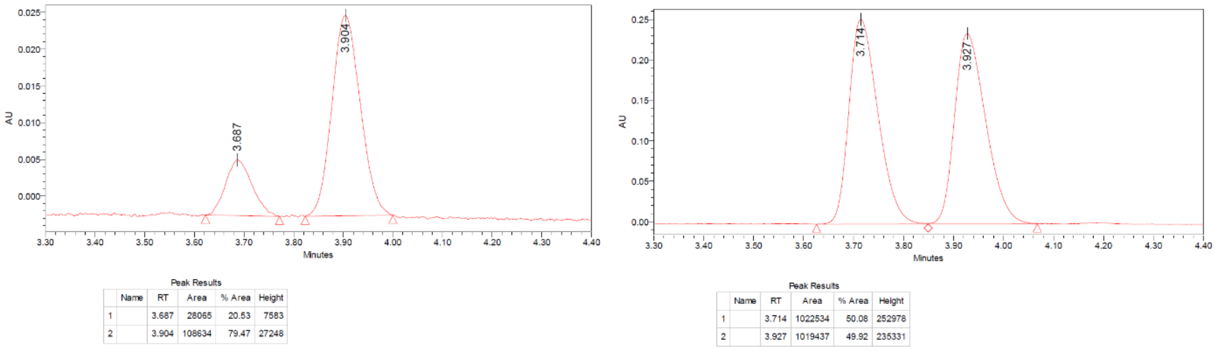

SFC of (*R*)-4-ethyl-1-(4-fluorobenzyl)-1,4-dihydropyridine-3-carbonitrile (**3e**)

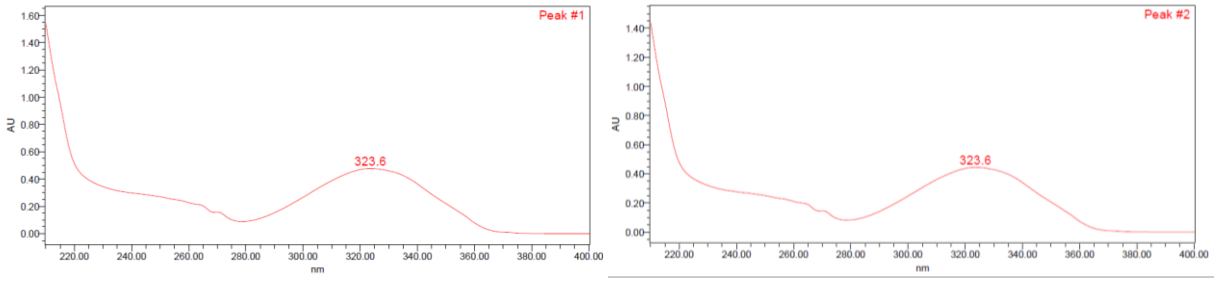

UV-visible spectra of (*R*)-4-ethyl-1-(4-fluorobenzyl)-1,4-dihydropyridine-3-carbonitrile (**3e**)

(*R*)-4-Ethyl-1-(naphthalen-2-ylmethyl)-1,4-dihydropyridine-3-carbonitrile (3f)

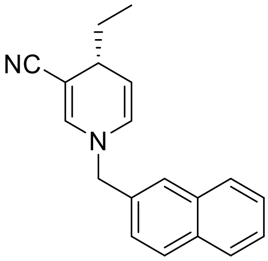

The reaction was performed with **1f** (65.0 mg, 0.2 mmol, 1.0 equiv.), CuTC (3.8 mg, 10.0 mol%), (*R*)-Tol-BINAP (16.3 mg, 12.0 mol%), EtMgBr (3.0 M in Et_2O, 80 μL, 0.24 mmol, 1.2 equiv.) in CH_2Cl_2 (2.0 mL) at −78 °C for 16 h. Product **3f** was obtained as a yellow oil after column chromatography (SiO_2, pentane:EtOAc = 3:1) [>99% conversion, 42.8 mg, 78% yield, 79% *ee*, (*R*)-configuration].

^{1}H NMR ($CDCl_3$, 400 MHz): δ 7.87–7.82 (m, 3H, 2 × C*H*$_{Ar}$), 7.64 (d, *J* = 8.0 Hz, 2H, 2 × C*H*$_{Ar}$), 7.54–7.49 (m, 2H, 2 × C*H*$_{Ar}$), 7.29 (dd, *J* = 8.4 and 1.8 Hz, 1H, C*H*$_{Ar}$), 6.71 (d, *J* = 1.6 Hz, 1H, C=C*H*), 5.86 (dt, *J* = 8.2 and 1.4 Hz, 1H, C*H*=CH), 4.64 (dd, *J* = 8.1 and 4.1 Hz, 1H, CH=C*H*), 4.43 (s, 2H, NC*H*$_2$), 3.28–3.25 (m, 1H, C*H*), 1.67–1.56 (m, 1H, C*H*H), 1.56–1.46 (m, 1H, CH*H*), 0.98 (t, *J* = 7.5 Hz, 3H, CH_3).

^{13}C NMR ($CDCl_3$, 101 MHz): δ 143.3, 134.0, 133.4, 133.1, 129.2, 128.4, 128.0, 127.9, 126.8, 126.5, 126.3, 124.8, 121.5, 106.1, 82.7, 57.8, 34.3, 30.1, 9.3.

LC-HRMS (ESI-TOF): m/z [M+Na]$^+$ calcd. for $C_{19}H_{18}N_2Na^+$: 297.1362; found 297.1361.

SFC: Trefoil CEL2, CO_2/MeOH with gradient from 97% to 90% in 10 min, 1.8 mL/min., 40 °C, detection at 324 nm. Retention time (min.): 4.14 (minor) and 4.31 (major).

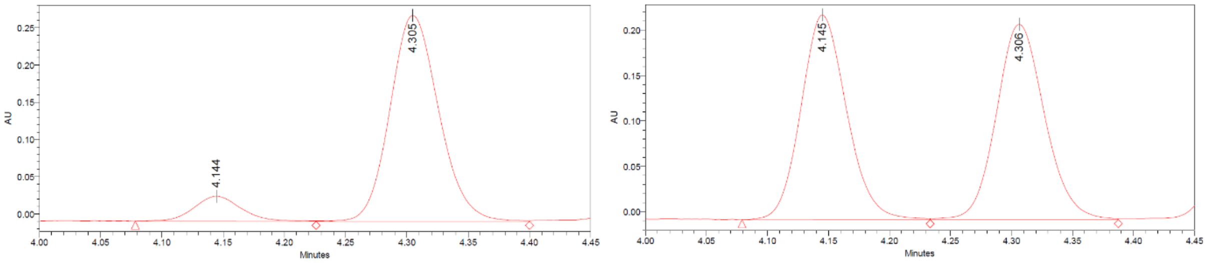

Peak Results

	Name	RT	Area	% Area	Height
1		4.144	89812	10.50	33752
2		4.305	765742	89.50	276424

Peak Results

	Name	RT	Area	% Area	Height
1		4.145	588032	49.82	225635
2		4.306	592378	50.18	215002

SFC of (*R*)-4-ethyl-1-(naphthalen-2-ylmethyl)-1,4-dihydropyridine-3-carbonitrile (**3f**)

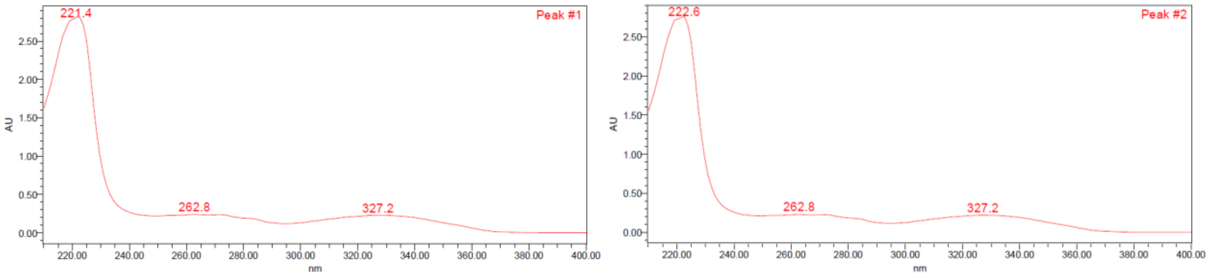

UV-visible spectra of (*R*)-4-ethyl-1-(naphthalen-2-ylmethyl)-1,4-dihydropyridine-3-carbonitrile (**3f**)

(*R*)-4-Ethyl-1-(3-methylbenzyl)-1,4-dihydropyridine-3-carbonitrile (3g)

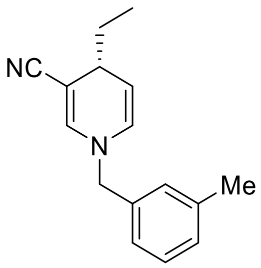

The reaction was performed with **1g** (68.6 mg, 0.2 mmol, 1.0 equiv.), CuTC (3.8 mg, 10.0 mol%), (*R*)-Tol-BINAP (16.3 mg, 12.0 mol%), EtMgBr (3.0 M in Et_2O, 80 μL, 0.24 mmol, 1.2 equiv.) in CH_2Cl_2 (2.0 mL) at −78 °C for 16 h. Product **3g** was obtained as a yellow oil after column chromatography (SiO_2, pentane:EtOAc = 3:1) [>99% conversion, 36.5 mg, 77% yield, 81% *ee*, (*R*)-configuration].

^{1}H NMR ($CDCl_3$, 400 MHz): δ 7.27–7.25 (m, 1H, CH_{Ar}), 7.13 (d, J = 7.6 Hz, 1H, CH_{Ar}), 6.99–6.98 (m, 2H, 2 × CH_{Ar}), 6.63 (s, 1H, C=C*H*), 5.80 (dd, J = 8.1 and 1.4 Hz, 1H, C*H*=CH), 4.61 (dd, J = 8.1 and 4.1 Hz, 1H, CH=C*H*), 4.24 (s, 2H, NCH_2), 3.26–3.22 (m, 1H, C*H*), 2.36 (s, 3H, CH_3), 1.65–1.43 (m, 2H, C*HH*), 0.96 (t, J = 7.5 Hz, 3H, CH_3).

^{13}C NMR ($CDCl_3$, 101 MHz): δ 143.3, 138.9, 136.5, 129.0, 129.0, 128.5, 127.9, 124.3, 121.6, 105.9, 82.4, 57.5, 34.2, 30.1, 21.5, 9.2.

LC-HRMS (ESI-TOF): m/z $[M+H]^+$ calcd. for $C_{16}H_{18}N_2H^+$: 239.1543; found 239.1541.

SFC: Trefoil CEL2, CO_2/MeOH with gradient from 97% to 90% in 10 min, 1.8 mL/min., 40 °C, detection at 324 nm. Retention time (min.): 3.70 (minor) and 3.90 (major).

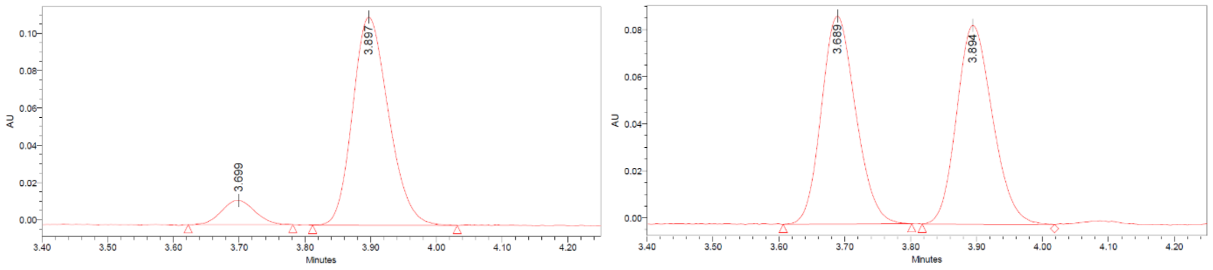

Peak Results

	Name	RT	Area	% Area	Height
1		3.699	45483	9.69	12971
2		3.897	423836	90.31	111581

Peak Results

	Name	RT	Area	% Area	Height
1		3.689	320805	49.99	88463
2		3.894	320937	50.01	84607

SFC of (*R*)-4-ethyl-1-(3-methylbenzyl)-1,4-dihydropyridine-3-carbonitrile (**3g**)

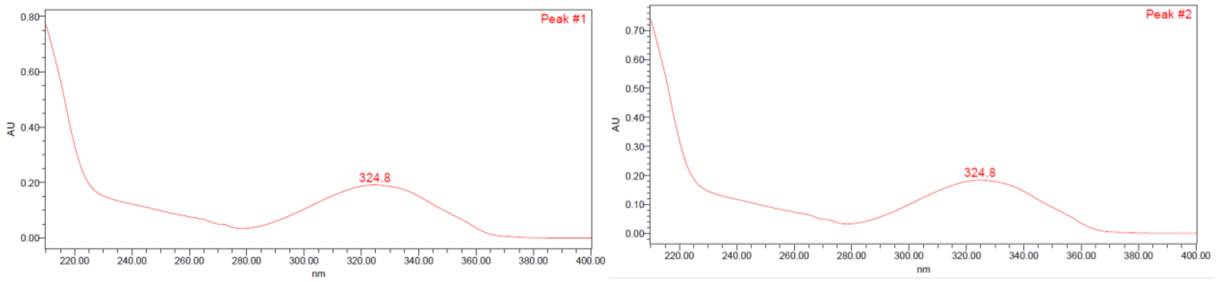

UV-visible spectra of (*R*)-4-ethyl-1-(3-methylbenzyl)-1,4-dihydropyridine-3-carbonitrile (**3g**)

(*R*)-4-Ethyl-1-(2-methylbenzyl)-1,4-dihydropyridine-3-carbonitrile (3h)

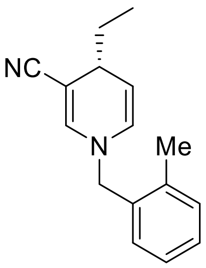

The reaction was performed with **1h** (68.6 mg, 0.2 mmol, 1.0 equiv.), CuTC (3.8 mg, 10.0 mol%), (*R*)-Tol-BINAP (16.3 mg, 12.0 mol%), EtMgBr (3.0 M in Et_2O, 80 μL, 0.24 mmol, 1.2 equiv.) in CH_2Cl_2 (2.0 mL) at −78 °C for 16 h. Product **3h** was obtained as a yellow oil after column chromatography (SiO_2, pentane:EtOAc = 3:1) [>99% conversion, 25.8 mg, 54% yield, 47% *ee*, (*R*)-configuration].

^{1}H NMR ($CDCl_3$, 400 MHz): δ 7.27–7.18 (m, 3H, 3 × CH_{Ar}), 7.15–7.13 (m, 1H, CH_{Ar}), 6.56 (d, *J* = 1.6 Hz, 1H, C=C*H*), 5.79 (dt, *J* = 8.2 and 1.3 Hz, 1H, C*H*=CH), 4.62 (dd, *J* = 8.2 and 4.1 Hz, 1H, CH=C*H*), 4.26 (s, 2H, NCH_2), 3.27–3.23 (m, 1H, C*H*), 2.26 (s, 3H, CH_3), 1.65–1.54 (m, 1H, C*H*H), 1.54–1.44 (m, 1H, CH*H*), 0.96 (t, *J* = 7.5 Hz, 3H, CH_3).

^{13}C NMR ($CDCl_3$, 101 MHz): δ 143.0, 136.3, 134.1, 131.0, 128.5, 128.4, 128.2, 126.6, 121.6, 105.9, 82.4, 55.6, 34.4, 30.2, 19.3, 9.3.

LC-HRMS (ESI-TOF): m/z $[M+H]^+$ calcd. for $C_{16}H_{18}N_2H^+$: 239.1543; found 239.1537.

SFC: Trefoil CEL2, CO_2/MeOH with gradient from 97% to 90% in 10 min, 1.8 mL/min., 40 °C, detection at 324 nm. Retention time (min.): 3.89 (minor) and 4.09 (major).

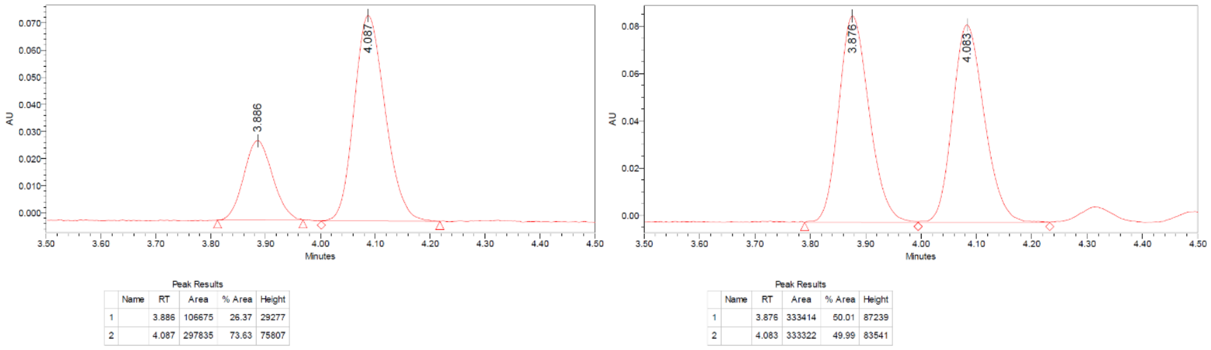

SFC of (*R*)-4-ethyl-1-(2-methylbenzyl)-1,4-dihydropyridine-3-carbonitrile (**3h**)

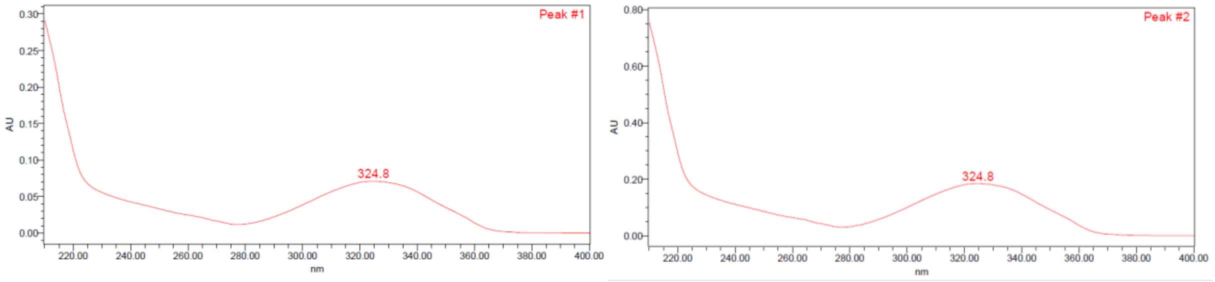

UV-visible spectra of (*R*)-4-ethyl-1-(2-methylbenzyl)-1,4-dihydropyridine-3-carbonitrile (**3h**)

(*R*)-1-(3,5-Di-*tert*-butylbenzyl)-4-ethyl-1,4-dihydropyridine-3-carbonitrile (3i)

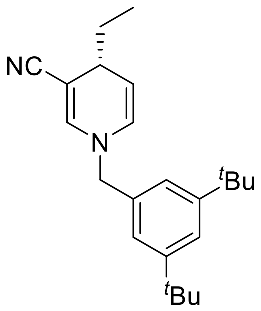

The reaction was performed with **1i** (77.5 mg, 0.2 mmol, 1.0 equiv.), CuTC (3.8 mg, 10.0 mol%), (*R*)-Tol-BINAP (16.3 mg, 12.0 mol%), EtMgBr (3.0 M in Et_2O, 80 µL, 0.24 mmol, 1.2 equiv.) in CH_2Cl_2 (2.0 mL) at −78 °C for 16 h. Product **3i** was obtained as a white solid after column chromatography (SiO_2, pentane:EtOAc = 3:1) [>99% conversion, 57.9 mg, 86% yield, 66% *ee*, (*R*)-configuration].

^{1}H NMR ($CDCl_3$, 400 MHz): δ 7.38 (t, *J* = 1.8 Hz, 1H, CH_{Ar}), 7.01 (d, *J* = 1.8 Hz, 2H, 2 × CH_{Ar}), 6.66 (d, *J* = 1.7 Hz, 1H, C=C*H*), 5.48 (dt, *J* = 8.2 and 1.4 Hz, 1H, C*H*=CH), 4.63 (dd, *J* = 8.2 and 4.1 Hz, 1H, CH=C*H*), 4.29 (s, 2H, NCH_2), 3.29–3.25 (m, 1H, C*H*), 1.67–1.56 (m, 1H, C*H*H), 1.54–1.44 (m, 1H, CH*H*), 1.33 (s, 18H, 9 × CH_3), 0.98 (t, *J* = 7.5 Hz, 3H, CH_3).

^{13}C NMR ($CDCl_3$, 101 MHz): δ 151.7 (2 × C), 143.5, 135.9, 128.8, 122.2, 121.7, 121.2 (2 × C), 105.6, 82.0, 58.0, 35.0, 34.2, 31.5, 30.0, 9.3.

LC-HRMS (ESI-TOF): m/z $[M+H]^+$ calcd. for $C_{23}H_{32}N_2H^+$: 337.2638; found 337.2629.

SFC: Trefoil CEL2, CO_2/MeOH with gradient from 97% to 90% in 10 min, 1.8 mL/min., 40 °C, detection at 324 nm. Retention time (min.): 2.42 (minor) and 2.60 (major).

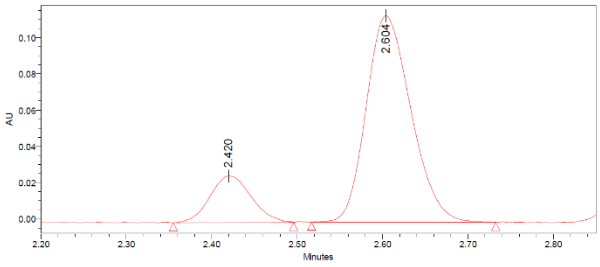

Peak Results

	Name	RT	Area	% Area	Height
1		2.420	86071	17.22	25631
2		2.604	413716	82.78	113797

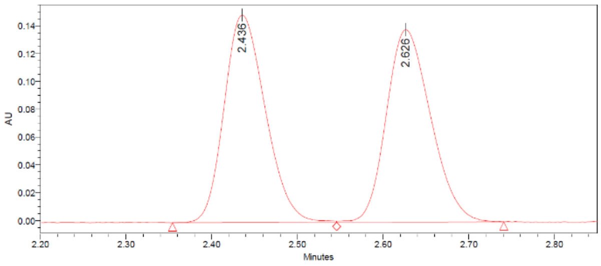

Peak Results

	Name	RT	Area	% Area	Height
1		2.436	500266	50.11	149213
2		2.626	498162	49.89	138315

SFC of (*R*)-1-(3,5-di-*tert*-butylbenzyl)-4-ethyl-1,4-dihydropyridine-3-carbonitrile (**3i**)

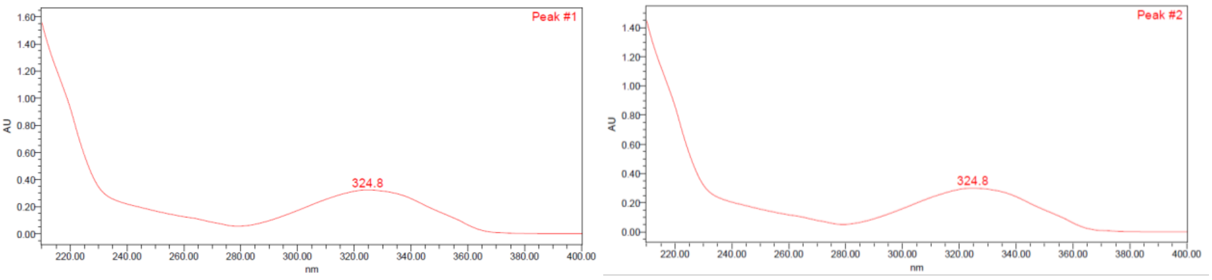

UV-visible spectra of (*R*)-1-(3,5-di-*tert*-butylbenzyl)-4-ethyl-1,4-dihydropyridine-3-carbonitrile (**3i**)

(*R*)-1-(3,5-dimethoxybenzyl)-4-ethyl-1,4-dihydropyridine-3-carbonitrile (3j)

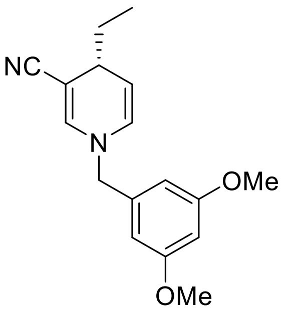

The reaction was performed with **1j** (67.0 mg, 0.2 mmol, 1.0 equiv.), CuTC (3.8 mg, 10.0 mol%), (*R*)-Tol-BINAP (16.3 mg, 12.0 mol%), EtMgBr (3.0 M in Et_2O, 80 µL, 0.24 mmol, 1.2 equiv.) in CH_2Cl_2 (2.0 mL) at −78 °C for 16 h. Product **3j** was obtained as a yellow oil after column chromatography (SiO_2, pentane:EtOAc = 3:1) [>99% conversion, 37.7 mg, 66% yield, 53% *ee*, (*R*)-configuration].

^{1}H NMR ($CDCl_3$, 400 MHz): δ 6.62 (d, *J* = 1.7 Hz, 1H, C=C*H*), 6.39 (t, *J* = 2.3 Hz, 1H, CH_{Ar}), 6.31 (d, *J* = 2.3 Hz, 2H, 2 × CH_{Ar}), 5.79 (d, *J* = 8.1 Hz, 1H, C*H*=CH), 4.62 (dd, *J* = 8.1 and 4.1 Hz, 1H, CH=C*H*), 4.20 (s, 2H, NCH_2), 3.78 (s, 6H, 2 × OCH_3), 3.26–3.22 (m, 1H, C*H*), 1.64–1.53 (m, 1H, C*H*H), 1.52–1.43 (m, 1H, CH*H*), 0.96 (t, *J* = 7.5 Hz, 3H, CH_3).

^{13}C NMR ($CDCl_3$, 101 MHz): δ 161.4 (2 × C), 143.3, 139.1, 128.5, 121.5, 105.9, 105.1, 99.8, 82.5, 57.5, 55.5 (2 × C), 34.2, 30.0, 9.3.

LC-HRMS (ESI-TOF): m/z [M+H]$^+$ calcd. for $C_{17}H_{20}N_2O_2H^+$: 285.1598; found 285.1594.

SFC: Trefoil CEL2, CO_2/MeOH with gradient from 97% to 90% in 10 min, 1.8 mL/min., 40 °C, detection at 324 nm. Retention time (min.): 3.42 (minor) and 3.61 (major).

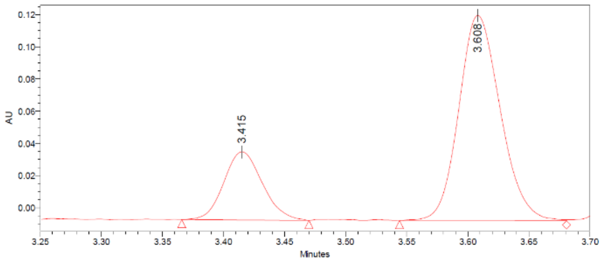

Peak Results

	Name	RT	Area	% Area	Height
1		3.415	92887	23.53	42322
2		3.608	301857	76.47	127588

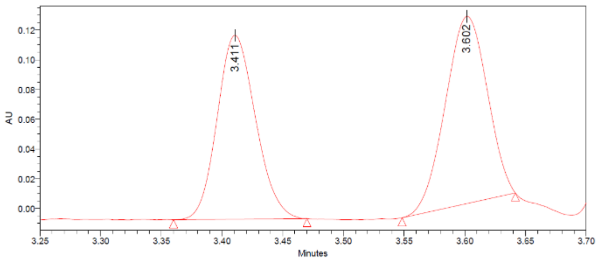

Peak Results

	Name	RT	Area	% Area	Height
1		3.411	271979	47.41	123692
2		3.602	301658	52.59	125922

SFC of (*R*)-1-(3,5-dimethoxybenzyl)-4-ethyl-1,4-dihydropyridine-3-carbonitrile (**3j**)

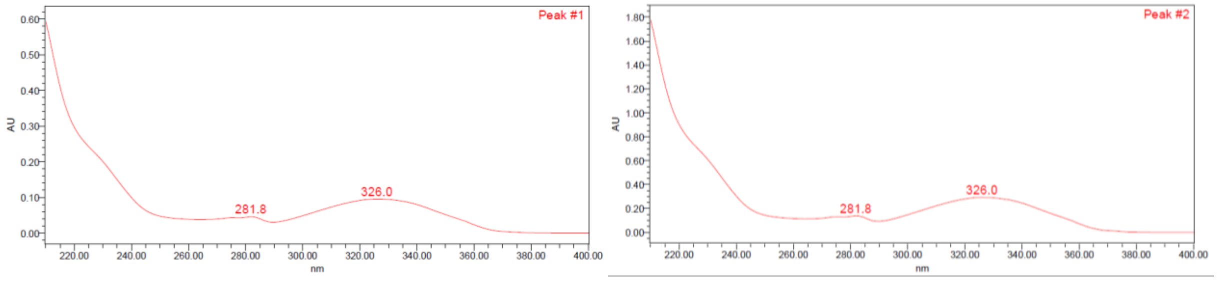

UV-visible spectra of (*R*)-1-(3,5-dimethoxybenzyl)-4-ethyl-1,4-dihydropyridine-3-carbonitrile (**3j**)

(*R*)-1-(3,5-bis(trifluoromethyl)benzyl)-4-ethyl-1,4-dihydropyridine-3-carbonitrile (3k)

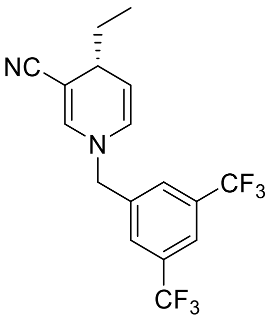

The reaction was performed with **1k** (82.2 mg, 0.2 mmol, 1.0 equiv.), CuTC (3.8 mg, 10.0 mol%), (*R*)-Tol-BINAP (16.3 mg, 12.0 mol%), EtMgBr (3.0 M in Et_2O, 80 µL, 0.24 mmol, 1.2 equiv.) in CH_2Cl_2 (2.0 mL) at −78 °C for 16 h. Product **3k** was obtained as a yellow oil after column chromatography (SiO_2, pentane:EtOAc = 3:1) [>99% conversion, 63.7 mg, 88% yield, 64% *ee*, (*R*)-configuration].

^{1}H NMR ($CDCl_3$, 400 MHz): δ 7.85 (s, 1H, CH_{Ar}), 7.66 (s, 2H, 2 × CH_{Ar}), 6.63 (d, *J* = 1.7 Hz, 1H, C=C*H*), 5.78 (dt, *J* = 8.1 and 1.5 Hz, 1H, C*H*=CH), 4.70 (dd, *J* = 8.1 and 4.1 Hz, 1H, CH=C*H*), 4.42 (s, 2H, NCH_2), 3.29 (td, *J* = 4.7 and 3.5 Hz, 1H, C*H*), 1.69–1.56 (m, 1H, C*H*H), 1.55–1.46 (m, 1H, CH*H*), 0.98 (t, *J* = 7.5 Hz, 3H, CH_3).

^{13}C NMR ($CDCl_3$, 101 MHz): δ 142.7, 139.7, 132.7, (q, *J* = 33 Hz), 127.9, 127.1, 127.05 (m), 122.5, 122.4, 122.3, 122.3, 119.0, 107.1, 84.4, 56.5, 34.1, 29.6, 9.1.

^{19}F NMR ($CDCl_3$, 376 MHz): δ −62.99.

LC-HRMS (ESI-TOF): m/z $[M+H]^+$ calcd. for $C_{17}H_{14}F_6N_2H^+$: 361.1134; found 361.1984.

SFC: Trefoil CEL2, CO_2/MeOH with gradient from 97% to 40% in 5 min, 1.8 mL/min., 40 °C, detection at 324 nm. Retention time (min.): 1.31 (minor) and 1.35 (major).

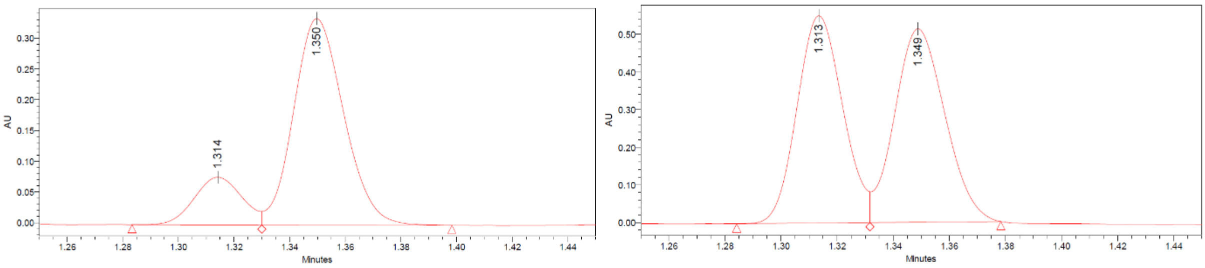

Peak Results

	Name	RT	Area	% Area	Height
1		1.314	92204	17.98	77411
2		1.350	420471	82.02	335832

Peak Results

	Name	RT	Area	% Area	Height
1		1.313	623302	49.24	551106
2		1.349	642482	50.76	514116

SFC of (*R*)-1-(3,5-bis(trifluoromethyl)benzyl)-4-ethyl-1,4-dihydropyridine-3-carbonitrile (**3k**)

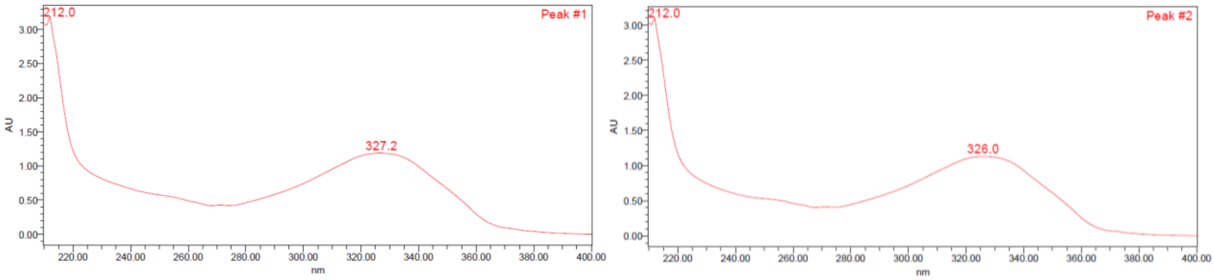

UV-visible spectra of (*R*)-1-(3,5-bis(trifluoromethyl)benzyl)-4-ethyl-1,4-dihydropyridine-3-carbonitrile (**3k**)

(*R*)-4-ethyl-1-methyl-1,4-dihydropyridine-3-carbonitrile (3l)

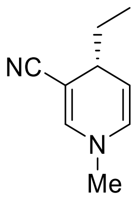

The reaction was performed with **1i** (82.2 mg, 0.2 mmol, 1.0 equiv.), CuTC (3.8 mg, 10.0 mol%), (*R*)-Tol-BINAP (16.3 mg, 12.0 mol%), EtMgBr (3.0 M in Et_2O, 80 µL, 0.24 mmol, 1.2 equiv.) in CH_2Cl_2 (2.0 mL) at −78 °C for 16 h. Product **3l** was obtained as a yellow oil after column chromatography (SiO_2, pentane:EtOAc = 3:1) [>99% conversion, 12.7 mg, 43% yield, 18% *ee*, (*R*)-configuration].

^{1}H NMR ($CDCl_3$, 400 MHz): δ 6.50 (d, *J* = 1.7 Hz, C=C*H*), 5.71 (dt, *J* = 8.1 and 1.3 Hz, 1H, C*H*=CH), 4.58 (dd, *J* = 8.1 and 4.1 Hz, 1H, CH=C*H*), 3.18–3.15 (m, 1H, C*H*), 2.92 (s, 3H, NCH_3), 1.58–1.40 (m, 2H, CH_2), 0.91 (t, *J* = 7.5 Hz, 3H, CH_3).

^{13}C NMR ($CDCl_3$, 101 MHz): δ 143.6, 129.0, 121.7, 105.5, 81.6, 40.9, 33.9, 30.2, 9.2.

LC-HRMS (ESI-TOF): m/z [M+Na]$^+$ calcd. for $C_9H_{12}N_2Na^+$: 171.0893; found 171.0992.

SFC: Trefoil CEL2, CO_2/MeOH with gradient from 97% to 40% in 5 min, 1.8 mL/min., 40 °C, detection at 324 nm. Retention time (min.): 1.52 (minor) and 1.57 (major).

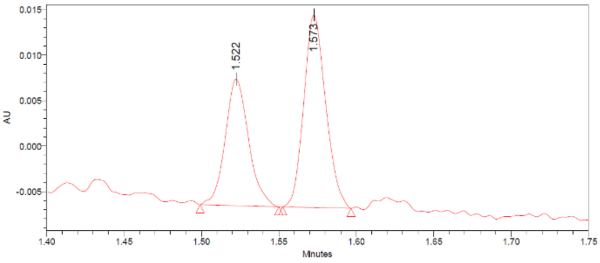

Peak Results

	Name	RT	Area	% Area	Height
1		1.522	14640	41.20	13934
2		1.573	20895	58.80	21182

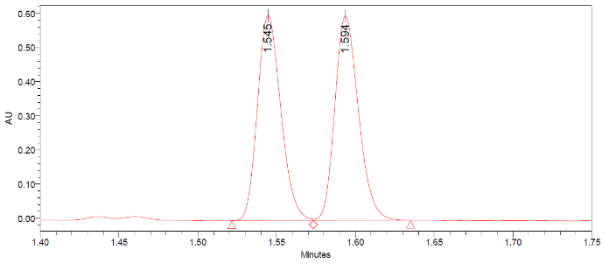

Peak Results

	Name	RT	Area	% Area	Height
1		1.545	620311	49.87	601571
2		1.594	623513	50.13	599620

SFC of (*R*)-4-ethyl-1-methyl-1,4-dihydropyridine-3-carbonitrile (**3l**)

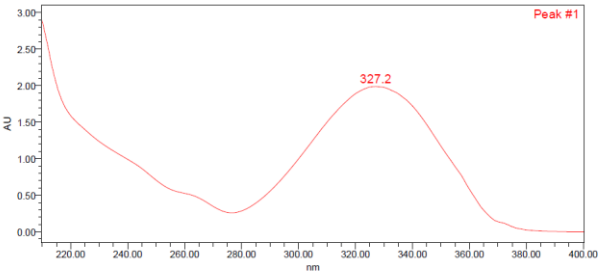

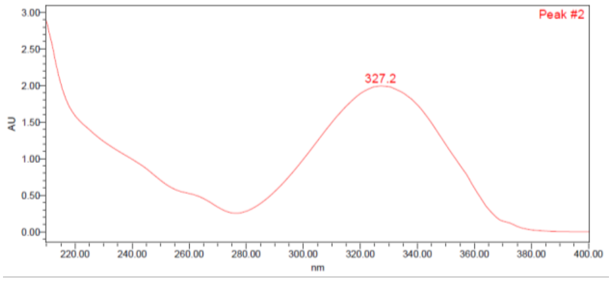

UV-visible spectra of (*R*)-4-ethyl-1-methyl-1,4-dihydropyridine-3-carbonitrile (**3l**)

(*R*)-4-ethyl-1-propyl-1,4-dihydropyridine-3-carbonitrile (3m)

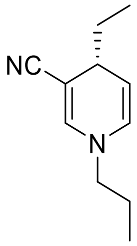

The reaction was performed with **1m** (45.4 mg, 0.2 mmol, 1.0 equiv.), CuTC (3.8 mg, 10.0 mol%), (*R*)-Tol-BINAP (16.3 mg, 12.0 mol%), EtMgBr (3.0 M in Et_2O, 80 μL, 0.24 mmol, 1.2 equiv.) in CH_2Cl_2 (2.0 mL) at −78 °C for 16 h. Product **3m** was obtained as a yellow oil after column chromatography (SiO_2, pentane:EtOAc = 3:1) [>99% conversion, 30.4 mg, 86% yield, 72% *ee*, (*R*)-configuration].

^{1}H NMR (CDCl$_3$, 400 MHz): δ 6.54 (d, *J* = 1.7 Hz, C=C*H*), 5.78 (dt, *J* = 8.1 and 1.3 Hz, 1H, C*H*=CH), 4.57 (dd, *J* = 8.1 and 4.1 Hz, 1H, CH=C*H*), 3.22–3.18 (m, 1H, C*H*), 3.04 (td, *J* = 6.9 and 2.1 Hz, 2H, NCH_2), 1.60–1.50 (m, 3H, CH_2 and C*H*H), 1.50–1.39 (m, 1H, CH*H*), 0.93 (t, *J* = 7.1 Hz, 3H, CH_3), 0.89 (t, *J* = 7.1 Hz, 3H, CH_3).

^{13}C NMR (CDCl$_3$, 101 MHz): δ 143.2, 128.2, 121.9, 105.4, 81.2, 55.8, 34.2, 30.2, 23.3, 10.9, 9.2.

LC-HRMS (ESI-TOF): m/z $[M+H]^+$ calcd. for $C_{11}H_{16}N_2H^+$: 177.1386; found 177.1382.

SFC: Trefoil CEL1, CO_2/MeOH with gradient from 97% to 90% in 10 min, 1.8 mL/min., 40 °C, detection at 324 nm. Retention time (min.): 1.17 (minor) and 1.25 (major).

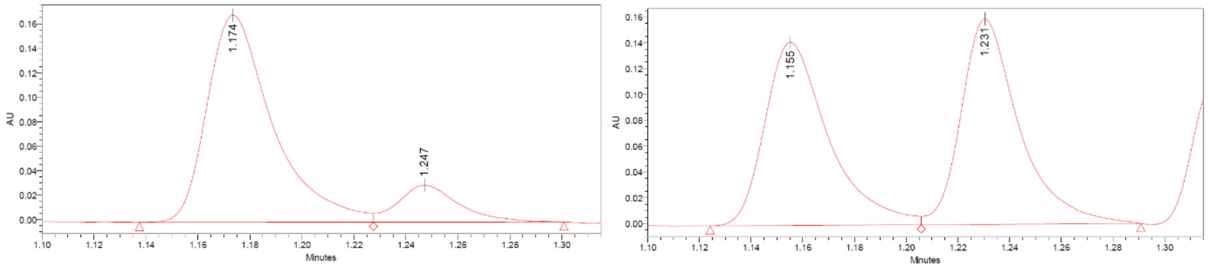

Peak Results

	Name	RT	Area	% Area	Height
1		1.174	297347	85.86	169200
2		1.247	48952	14.14	30141

Peak Results

	Name	RT	Area	% Area	Height
1		1.155	243418	49.46	141939
2		1.231	248773	50.54	159107

SFC of (*R*)-4-ethyl-1-propyl-1,4-dihydropyridine-3-carbonitrile (**3m**)

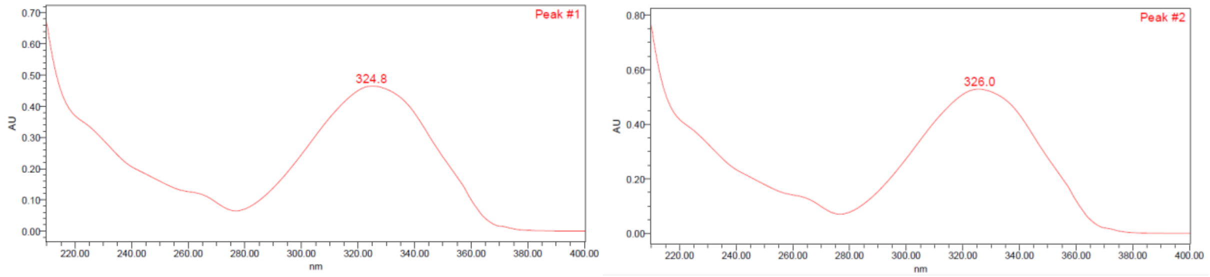

UV-visible spectra of (*R*)-4-ethyl-1-propyl-1,4-dihydropyridine-3-carbonitrile (**3m**)

(*R*)-1-(but-3-en-1-yl)-4-ethyl-1,4-dihydropyridine-3-carbonitrile (3n)

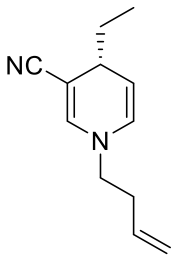

The reaction was performed with **1n** (47.8 mg, 0.2 mmol, 1.0 equiv.), CuTC (3.8 mg, 10.0 mol%), (*R*)-Tol-BINAP (16.3 mg, 12.0 mol%), EtMgBr (3.0 M in Et_2O, 80 µL, 0.24 mmol, 1.2 equiv.) in CH_2Cl_2 (2.0 mL) at −78 °C for 16 h. Product **3n** was obtained as a yellow oil after column chromatography (SiO_2, pentane:EtOAc = 3:1) [>99% conversion, 34.2 mg, 91% yield, 82% *ee*, (*R*)-configuration].

^{1}H NMR ($CDCl_3$, 400 MHz): δ 6.53 (d, *J* = 1.6 Hz, 1H, C=C*H*), 5.76 (d, *J* = 8.0 Hz, 1H, C*H*=CH), 5.74–5.65 (m, 1H, C*H*=CH_2), 5.13–5.08 (m, 2H, CH=CH_2), 4.58 (dd, *J* = 8.0 and 4.1 Hz, 1H, CH=C*H*), 3.19 (q, *J* = 4.4 Hz, 1H, C*H*), 3.14 (t, *J* = 7.0 Hz, 2H, NCH_2), 2.28–2.23 (m, 2H, CH_2), 1.60–1.49 (m, 1H, C*H*H), 1.49–1.39 (m, 1H, CH*H*), 0.92 (t, *J* = 7.5 Hz, 3H, CH_3).

^{13}C NMR ($CDCl_3$, 101 MHz): δ 143.1, 133.8, 128.1, 121.7, 118.4, 105.6, 81.5, 53.7, 34.5, 34.1, 30.1, 9.2.

LC-HRMS (ESI-TOF): m/z $[M+H]^+$ calcd. for $C_{12}H_{13}N_2H^+$: 189.1386; found 189.1385.

SFC: Trefoil CEL1, CO_2/MeOH with gradient from 97% to 90% in 10 min, 1.8 mL/min., 40 °C, detection at 324 nm. Retention time (min.): 1.27 (major) and 1.43 (minor).

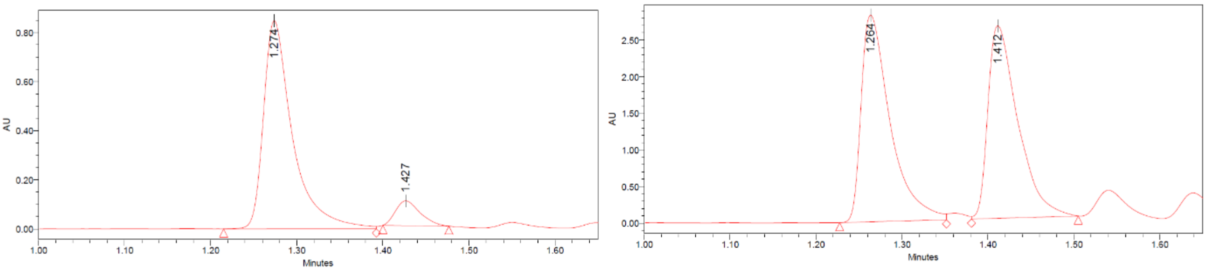

Peak Results

	Name	RT	Area	% Area	Height
1		1.274	1991808	90.87	848013
2		1.427	200090	9.13	100639

Peak Results

	Name	RT	Area	% Area	Height
1		1.264	6648410	51.14	2821825
2		1.412	6351693	48.86	2626544

SFC of (*R*)-1-(but-3-en-1-yl)-4-ethyl-1,4-dihydropyridine-3-carbonitrile (**3n**)

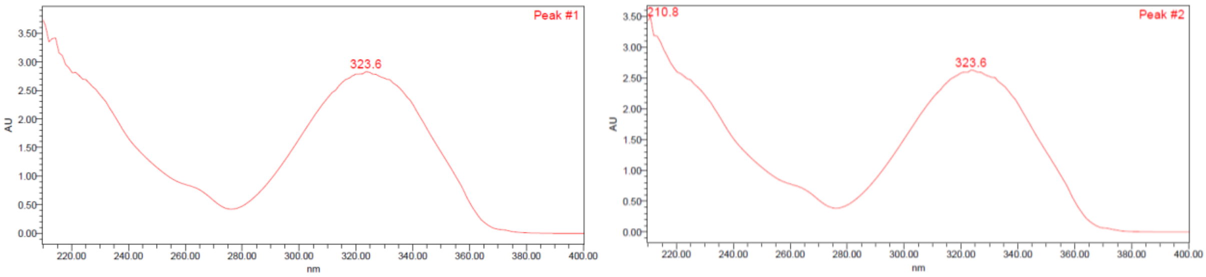

UV-visible spectra of (*R*)-1-(but-3-en-1-yl)-4-ethyl-1,4-dihydropyridine-3-carbonitrile (**3n**)

(*R*)-1-Benzyl-4-propyl-1,4-dihydropyridine-3-carbonitrile (4a)

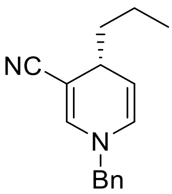

The reaction was performed with **1a** (55.0 mg, 0.2 mmol, 1.0 equiv.), CuTC (3.8 mg, 10.0 mol%), (*R*)-Tol-BINAP (16.3 mg, 12.0 mol%), ^{n}PrMgBr (2.0 M in Et_2O, 120 μL, 0.24 mmol, 1.2 equiv.) in CH_2Cl_2 (2.0 mL) at −78 °C for 16 h. Product **4a** was obtained as a yellow oil after column chromatography (SiO_2, pentane:EtOAc = 3:1) [>99% conversion, 42.0 mg, 88% yield, 84% *ee*, (*R*)-configuration].

^{1}H NMR ($CDCl_3$, 400 MHz): δ 7.40–7.29 (m, 3H, 3 × CH_{Ar}), 7.20–7.18 (m, 2H, 2 × CH_{Ar}), 6.61 (d, *J* = 1.6 Hz, 1H, C=C*H*), 5.77 (dt, *J* = 8.1 and 1.6 Hz, 1H, C*H*=CH), 4.66 (dd, *J* = 8.1 and 4.1 Hz, 1H, CH=C*H*), 4.28 (s, 2H, NCH_2), 3.24–3.20 (m, 1H, C*H*), 1.59–1.30 (m, 4H, 4 × CH_2), 0.94 (t, *J* = 7.1 Hz, 3H, CH_3).

^{13}C NMR ($CDCl_3$, 101 MHz): δ 143.0, 136.6, 129.1 (2 × C), 128.2, 128.1, 127.2 (2 × C), 121.6, 106.4, 83.1, 57.5, 40.4, 33.0, 18.3, 14.2.

LC-HRMS (ESI-TOF): m/z $[M+H]^+$ calcd. for $C_{16}H_{19}N_2^+$: 239.1543; found 239.1544.

SFC: Trefoil CEL2, CO_2/MeOH with gradient from 97% to 90% in 10 min, 1.8 mL/min., 40 °C, detection at 324 nm. Retention time (min.): 3.81 (minor) and 4.02 (major).

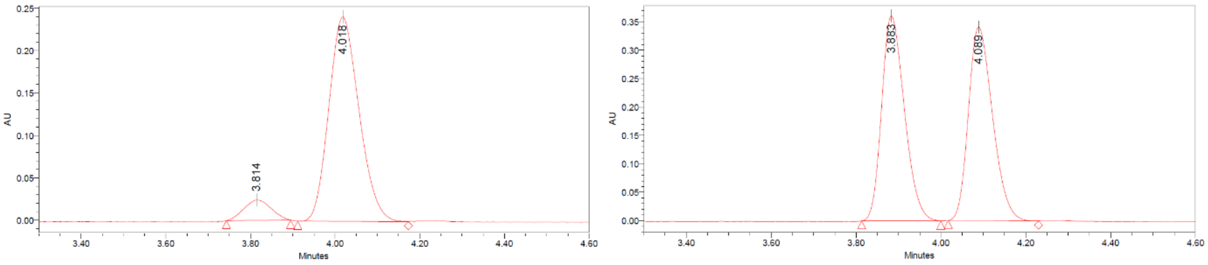

Peak Results

	Name	RT	Area	% Area	Height
1		3.814	106856	8.23	24013
2		4.018	1191877	91.77	240824

Peak Results

	Name	RT	Area	% Area	Height
1		3.883	1347799	49.99	360870
2		4.089	1348283	50.01	340916

SFC of (*R*)-1-benzyl-4-propyl-1,4-dihydropyridine-3-carbonitrile (**4a**)

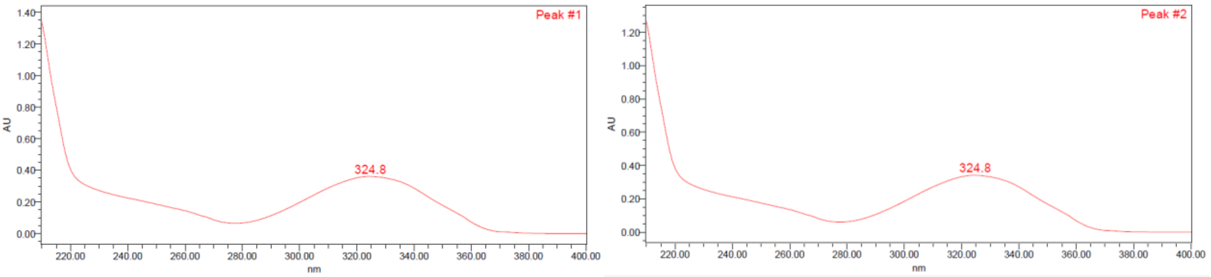

UV-visible spectra of (*R*)-1-benzyl-4-propyl-1,4-dihydropyridine-3-carbonitrile (**4a**)

(R)-1-Benzyl-4-pentyl-1,4-dihydropyridine-3-carbonitrile (4b)

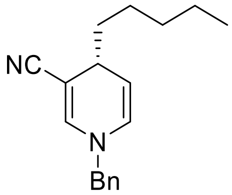

The reaction was performed with **1a** (55.0 mg, 0.2 mmol, 1.0 equiv.), CuTC (3.8 mg, 10.0 mol%), (*R*)-Tol-BINAP (16.3 mg, 12.0 mol%), ^{n}PentMgBr (2.0 M in Et_2O, 120 µL, 0.24 mmol, 1.2 equiv.) in CH_2Cl_2 (2.0 mL) at −78 °C for 16 h. Product **4b** was obtained as a yellow oil after column chromatography (SiO_2, pentane:EtOAc = 3:1) [>99% conversion, 47.6 mg, 89% yield, 91% *ee*, (*R*)-configuration].

^{1}H NMR ($CDCl_3$, 400 MHz): δ 7.39–7.30 (m, 3H, 3 × CH_{Ar}), 7.21–7.18 (m, 2H, 2 × CH_{Ar}), 6.61 (d, *J* = 1.7 Hz, 1H, C=C*H*), 5.78 (dt, *J* = 8.1 and 0.9 Hz, 1H, C*H*=CH), 4.65 (dd, *J* = 8.1 and 4.1 Hz, 1H, CH=C*H*), 4.28 (s, 2H, NCH_2), 3.23 (dt, *J* = 7.8 and 4.0 Hz, 1H, C*H*), 1.58–1.26 (m, 8H, 8 × CH_2), 0.90 (t, *J* = 6.8 Hz, 3H, CH_3).

^{13}C NMR ($CDCl_3$, 101 MHz): δ 143.0, 126.7, 129.1 (2 × C), 128.2, 128.1, 127.2 (2 × C), 121.6, 106.4, 83.1, 57.5, 37.9, 33.2, 32.0, 24.8, 22.8, 14.2.

LC-HRMS (ESI-TOF): m/z $[M+H]^+$ calcd. for $C_{18}H_{23}N_2^+$: 267.1856; found 267.1128.

SFC: Trefoil CEL2, CO_2/MeOH with gradient from 97% to 90% in 10 min, 1.8 mL/min., 40 °C, detection at 324 nm. Retention time (min.): 4.19 (minor) and 4.41 (major).

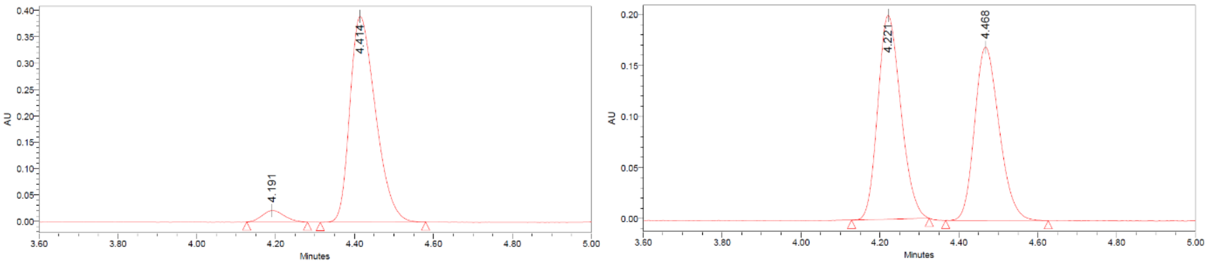

Peak Results

	Name	RT	Area	% Area	Height
1		4.191	84030	4.54	21542
2		4.414	1765334	95.46	390262

Peak Results

	Name	RT	Area	% Area	Height
1		4.221	810045	52.36	199563
2		4.468	737110	47.64	169742

SFC of (*R*)-1-benzyl-4-pentyl-1,4-dihydropyridine-3-carbonitrile (**4b**)

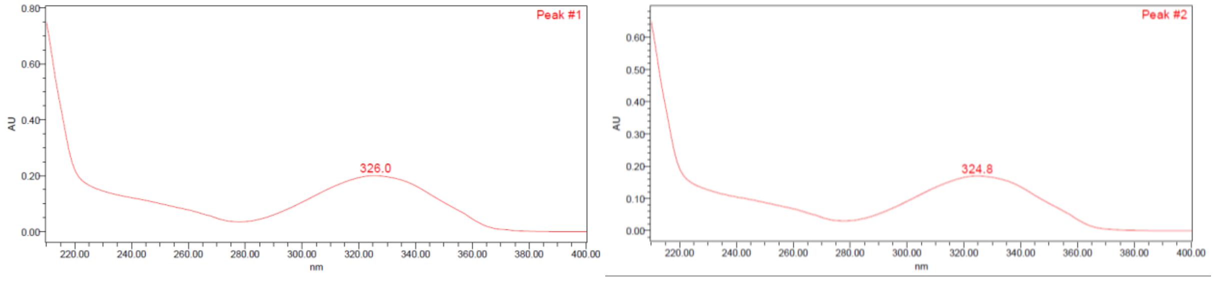

UV-visible spectra of (*R*)-1-benzyl-4-pentyl-1,4-dihydropyridine-3-carbonitrile (**4b**)

(*R*)-1-Benzyl-4-hexyl-1,4-dihydropyridine-3-carbonitrile (4c)

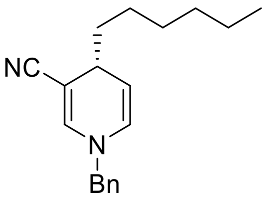

The reaction was performed with **1a** (55.0 mg, 0.2 mmol, 1.0 equiv.), CuTC (3.8 mg, 10.0 mol%), (*R*)-Tol-BINAP (16.3 mg, 12.0 mol%), ^{n}HexMgBr (2.0 M in Et_2O, 120 µL, 0.24 mmol, 1.2 equiv.) in CH_2Cl_2 (2.0 mL) at −78 °C for 16 h. Product **4c** was obtained as a yellow oil after column chromatography (SiO_2, pentane:EtOAc = 3:1) [>99% conversion, 50.1 mg, 89% yield, 94% *ee*, (*R*)-configuration].

^{1}H NMR ($CDCl_3$, 400 MHz): δ 7.39–7.29 (m, 3H, 3 × CH_{Ar}), 7.21–7.18 (m, 2H, 2 × CH_{Ar}), 6.61 (d, *J* = 1.7 Hz, 1H, C=C*H*), 5.78 (dt, *J* = 8.1 and 1.4 Hz, 1H, C*H*=CH), 4.65 (dd, *J* = 8.1 and 4.1 Hz, 1H, CH=C*H*), 4.28 (s, 2H, NCH_2), 3.24–3.21 (m, 1H, C*H*), 1.58–1.25 (m, 10H, 10 × CH_2), 0.88 (t, *J* = 6.8 Hz, 3H, CH_3).

^{13}C NMR ($CDCl_3$, 101 MHz): δ 143.0, 136.7, 129.1 (2 × C), 128.2, 128.1, 127.2 (2 × C), 121.5, 106.4, 83.0, 57.5, 38.0, 33.2, 32.0, 29.5, 25.1, 22.7, 14.2.

LC-HRMS (ESI-TOF): m/z $[M+H]^+$ calcd. for $C_{19}H_{25}N_2^+$: 281.2012; found 281.1650.

SFC: Trefoil CEL2, CO_2/MeOH with gradient from 97% to 90% in 10 min, 1.8 mL/min., 40 °C, detection at 324 nm. Retention time (min.): 4.37 (minor) and 4.56 (major).

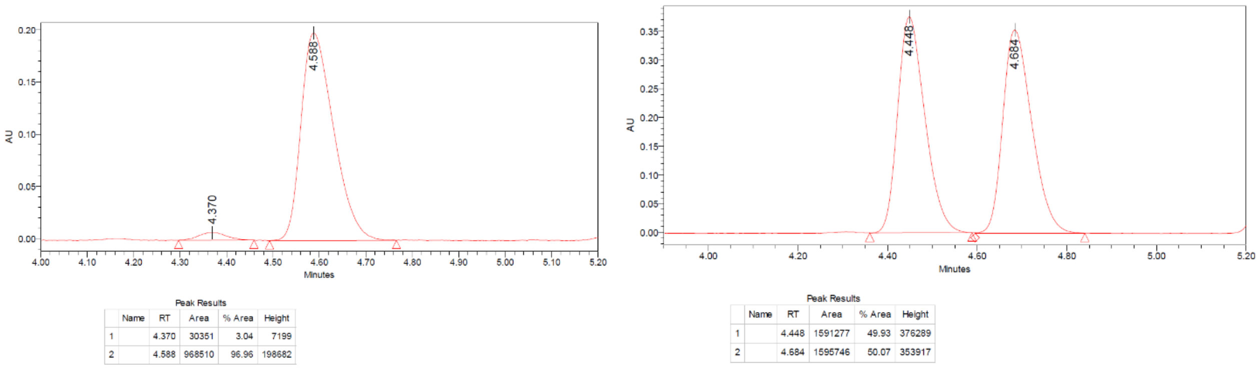

	Name	RT	Area	% Area	Height
1		4.370	30351	3.04	7199
2		4.588	968510	96.96	198682

	Name	RT	Area	% Area	Height
1		4.448	1591277	49.93	376289
2		4.684	1595746	50.07	353917

SFC of (*R*)-1-benzyl-4-hexyl-1,4-dihydropyridine-3-carbonitrile (**4c**)

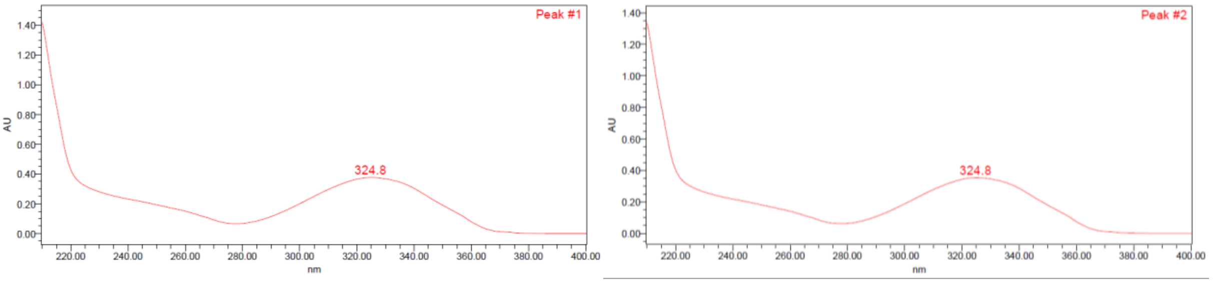

UV-visible spectra of (*R*)-1-benzyl-4-hexyl-1,4-dihydropyridine-3-carbonitrile (**4c**)

(*R*)-1-Benzyl-4-octyl-1,4-dihydropyridine-3-carbonitrile (4d)

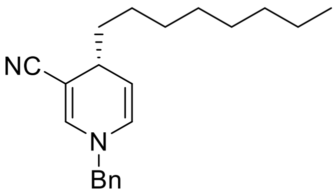

The reaction was performed with **1a** (55.0 mg, 0.2 mmol, 1.0 equiv.), CuTC (3.8 mg, 10.0 mol%), (*R*)-Tol-BINAP (16.3 mg, 12.0 mol%), ^{n}OctMgBr (2.0 M in Et_2O, 120 μL, 0.24 mmol, 1.2 equiv.) in CH_2Cl_2 (2.0 mL) at −78 °C for 16 h. Product **4d** was obtained as a yellow oil after column chromatography (SiO_2, pentane:EtOAc = 3:1) [>99% conversion, 54.4 mg, 88% yield, 90% *ee*, (*R*)-configuration].

^{1}H NMR ($CDCl_3$, 400 MHz): δ 7.39–7.30 (m, 3H, 3 × CH_{Ar}), 7.21–7.18 (m, 2H, 2 × CH_{Ar}), 6.61 (d, *J* = 1.7 Hz, 1H, C=C*H*), 5.78 (d, *J* = 8.1 Hz, 1H, C*H*=CH), 4.65 (dd, *J* = 8.1 and 4.1 Hz, 1H, CH=C*H*), 4.28 (s, 2H, NCH_2), 3.24–3.21 (m, 1H, C*H*), 1.59–1.21 (m, 14H, 7 × CH_2), 0.89 (t, *J* = 6.7 Hz, 3H, CH_3).

^{13}C NMR ($CDCl_3$, 101 MHz): δ 143.0, 136.7, 129.1 (2 × C), 128.2, 128.1, 127.2 (2 × C), 121.6, 106.5, 83.1, 57.5, 38.0, 33.2, 32.0, 29.8, 29.8, 29.4, 25.1, 22.8, 14.3.

LC-HRMS (ESI-TOF): m/z $[M+H]^+$ calcd. for $C_{21}H_{29}N_2^+$: 309.2325; found 309.1602.

SFC: Trefoil CEL2, CO_2/MeOH with gradient from 97% to 90% in 10 min, 1.8 mL/min., 40 °C, detection at 324 nm. Retention time (min.): 4.78 (minor) and 5.02 (major).

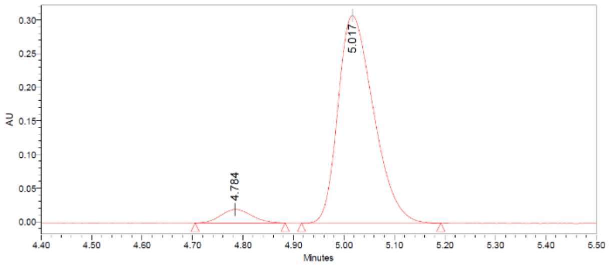

Peak Results

	Name	RT	Area	% Area	Height
1		4.784	87385	5.47	20429
2		5.017	1510129	94.53	308581

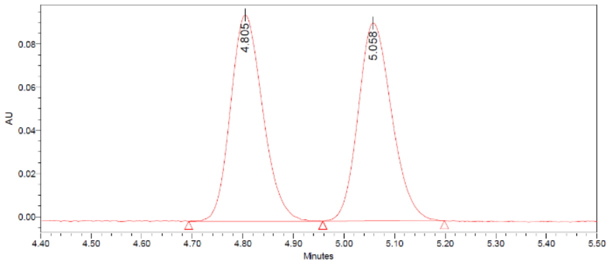

Peak Results

	Name	RT	Area	% Area	Height
1		4.805	423529	50.02	95604
2		5.058	423179	49.98	91702

SFC of (*R*)-1-benzyl-4-octyl-1,4-dihydropyridine-3-carbonitrile (**4d**)

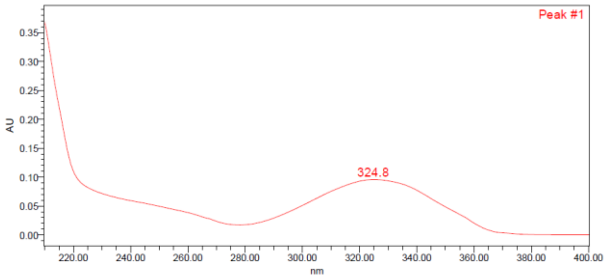

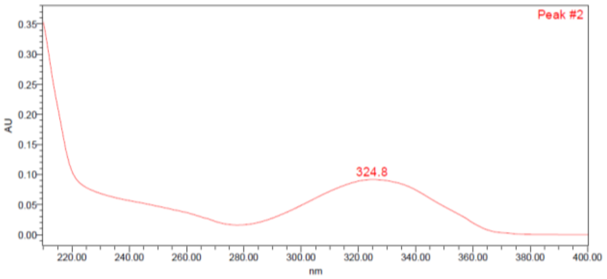

UV-visible spectra of (*R*)-1-benzyl-4-octyl-1,4-dihydropyridine-3-carbonitrile (**4d**)

(*R*)-1-Benzyl-4-nonyl-1,4-dihydropyridine-3-carbonitrile (4e)

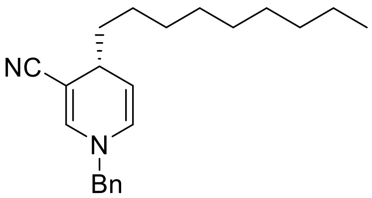

The reaction was performed with **1a** (55.0 mg, 0.2 mmol, 1.0 equiv.), CuTC (3.8 mg, 10.0 mol%), (*R*)-Tol-BINAP (16.3 mg, 12.0 mol%), ^{n}NonMgBr (1.5 M in Et_2O, 160 µL, 0.24 mmol, 1.2 equiv.) in CH_2Cl_2 (2.0 mL) at −78 °C for 16 h. Product **4e** was obtained as a yellow oil after column chromatography (SiO_2, pentane:EtOAc = 3:1) [>99% conversion, 58.9 mg, 91% yield, 91% *ee*, (*R*)-configuration].

^{1}H NMR ($CDCl_3$, 400 MHz): δ 7.39–7.30 (m, 3H, 3 × CH_{Ar}), 7.21–7.18 (m, 2H, 2 × CH_{Ar}), 6.61 (d, *J* = 1.6 Hz, 1H, C=C*H*), 5.78 (d, *J* = 8.1 Hz, 1H, C*H*=CH), 4.65 (dd, *J* = 8.1 and 4.1 Hz, 1H, CH=C*H*), 4.28 (s, 2H, NCH_2), 3.24–3.21 (m, 1H, C*H*), 1.61–1.19 (m, 16H, 8 × CH_2), 0.89 (t, *J* = 6.7 Hz, 3H, CH_3).

^{13}C NMR ($CDCl_3$, 101 MHz): δ 143.0, 136.7, 129.1 (2 × C), 128.2, 128.1, 127.2 (2 × C), 121.6, 106.5, 83.1, 57.5, 38.0, 33.2, 32.0, 29.8, 29.8, 29.7, 29.5, 25.1, 22.8, 14.3.

LC-HRMS (APCI): m/z $[M]^+$ calcd. for $C_{22}H_{30}N_2^+$: 322.2409; found 322.1205.

SFC: Trefoil CEL2, CO_2/MeOH with gradient from 97% to 90% in 10 min, 1.8 mL/min., 40 °C, detection at 324 nm. Retention time (min.): 4.97 (minor) and 5.21 (major).

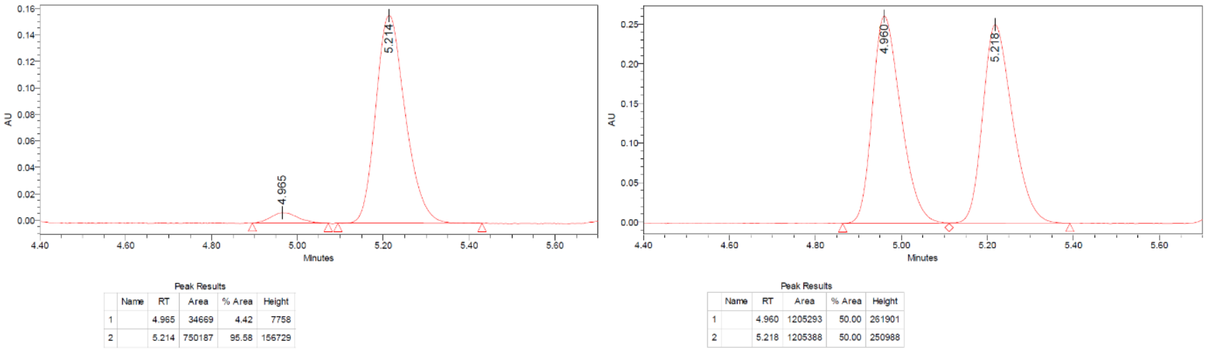

SFC of (*R*)-1-Benzyl-4-nonyl-1,4-dihydropyridine-3-carbonitrile (**4e**)

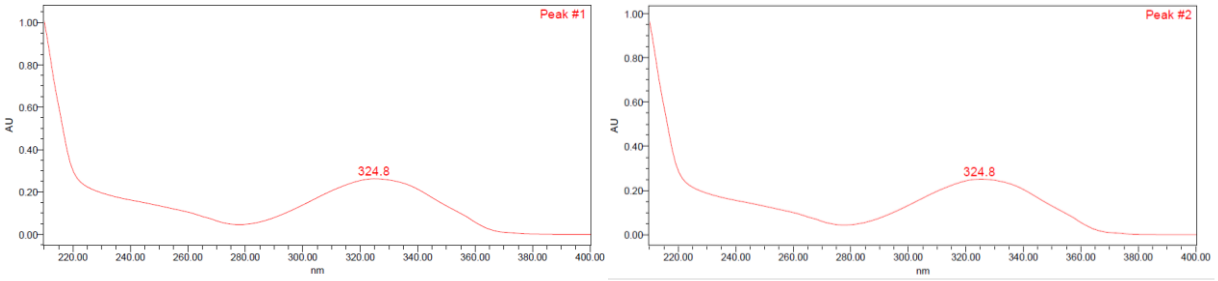

UV-visible spectra of (*R*)-1-Benzyl-4-nonyl-1,4-dihydropyridine-3-carbonitrile (**4e**)

(*R*)-1-Benzyl-4-undecyl-1,4-dihydropyridine-3-carbonitrile (4f)

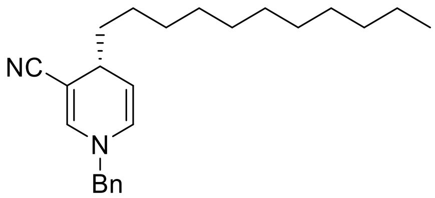

The reaction was performed with **1a** (55.0 mg, 0.2 mmol, 1.0 equiv.), CuTC (3.8 mg, 10.0 mol%), (*R*)-Tol-BINAP (16.3 mg, 12.0 mol%), ^{n}UndecMgBr (2.5 M in Et_2O, 96 μL, 0.24 mmol, 1.2 equiv.) in CH_2Cl_2 (2.0 mL) at −78 °C for 16 h. Product **4f** was obtained as a yellow oil after column chromatography (SiO_2, pentane:EtOAc = 3:1) [>99% conversion, 58.1 mg, 83% yield, 84% *ee*, (*R*)-configuration].

^{1}H NMR ($CDCl_3$, 400 MHz): δ 7.39–7.30 (m, 3H, 3 × CH_{Ar}), 7.21–7.19 (m, 2H, 2 × CH_{Ar}), 6.61 (d, *J* = 1.7 Hz, 1H, C=C*H*), 5.78 (d, *J* = 8.1 Hz, 1H, C*H*=CH), 4.65 (dd, *J* = 8.1 and 4.1 Hz, 1H, CH=C*H*), 4.28 (s, 2H, NCH_2), 3.24–3.21 (m, 1H, C*H*), 1.60–1.19 (m, 20H, 10 × CH_2), 0.88 (t, *J* = 6.7 Hz, 3H, CH_3).

^{13}C NMR ($CDCl_3$, 101 MHz): δ 143.0, 136.7, 129.1 (2 × C), 128.2, 128.1, 127.2 (2 × C), 121.6, 106.5, 83.1, 57.5, 38.0, 33.2, 32.1, 29.9, 29.8, 29.8, 29.8, 29.8, 29.5, 25.2, 22.8, 14.3.

LC-HRMS (ESI-TOF): m/z $[M]^+$ calcd. for $C_{24}H_{34}N_2^+$: 350.2722; found 350.2667.

SFC: Trefoil CEL2, CO_2/MeOH with gradient from 97% to 90% in 10 min, 1.8 mL/min., 40 °C, detection at 324 nm. Retention time (min.): 5.26 (minor) and 5.53 (major).

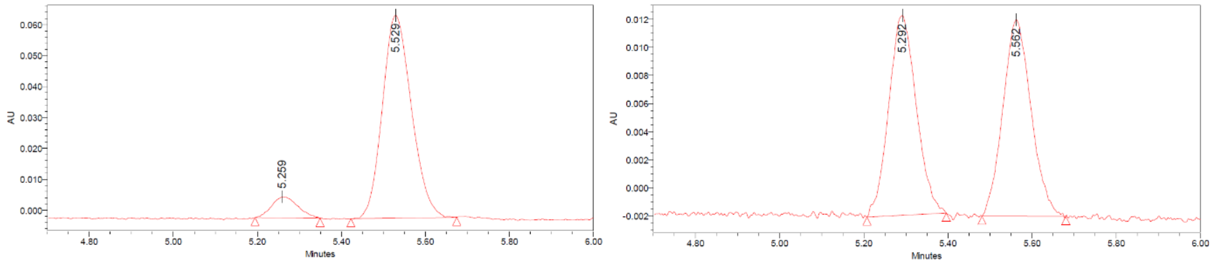

Peak Results

	Name	RT	Area	% Area	Height
1		5.259	29499	8.56	6791
2		5.529	315269	91.44	65548

Peak Results

	Name	RT	Area	% Area	Height
1		5.292	64810	49.83	14223
2		5.562	65261	50.17	13991

SFC of (*R*)-1-Benzyl-4-undecyl-1,4-dihydropyridine-3-carbonitrile (**4f**)

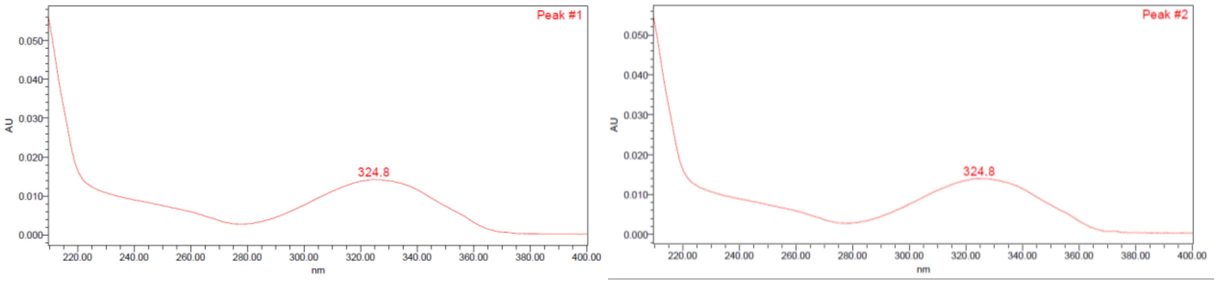

UV-visible spectra of (*R*)-1-Benzyl-4-undecyl-1,4-dihydropyridine-3-carbonitrile (**4f**)

(*R*)-1-Benzyl-4-isopentyl-1,4-dihydropyridine-3-carbonitrile (4g)

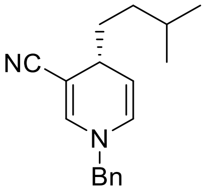

The reaction was performed with **1a** (55.0 mg, 0.2 mmol, 1.0 equiv.), CuTC (3.8 mg, 10.0 mol%), (*R*)-Tol-BINAP (16.3 mg, 12.0 mol%), ^{i}PentMgBr (2.0 M in Et_2O, 120 μL, 0.24 mmol, 1.2 equiv.) in CH_2Cl_2 (2.0 mL) at −78 °C for 16 h. Product **4g** was obtained as a yellow oil after column chromatography (SiO_2, pentane:EtOAc = 3:1) [>99% conversion, 49.1 mg, 92% yield, 91% *ee*, (*R*)-configuration].

^{1}H NMR ($CDCl_3$, 400 MHz): δ 7.39–7.30 (m, 3H, 3 × C*H*$_{Ar}$), 7.21–7.19 (m, 2H, 2 × C*H*$_{Ar}$), 6.61 (d, *J* = 1.7 Hz, 1H, C=C*H*), 5.78 (dt, *J* = 8.1 and 1.3 Hz, 1H, C*H*=CH), 4.64 (dd, *J* = 8.1 and 4.1 Hz, 1H, CH=C*H*), 4.28 (s, 2H, NC*H*$_2$), 3.25–3.21 (m, 1H, C*H*), 1.60–1.43 (m, 3H, 3 × C*H*), 1.35–1.18 (m, 2H, 2 × C*H*), 0.90 (dd, *J* = 6.6 and 2.5 Hz, 6H, 2 × C*H*$_3$).

^{13}C NMR ($CDCl_3$, 101 MHz): δ 143.1, 136.7, 129.1 (2 × C), 128.2, 128.2, 127.2 (2 × C), 121.5, 106.4, 83.0, 57.5, 35.7, 34.2, 33.3, 28.2, 22.9, 22.7.

LC-HRMS (ESI-TOF): m/z $[M+H]^+$ calcd. for $C_{18}H_{23}N_2^+$: 267.1856; found 267.1582.

SFC: Trefoil CEL2, CO_2/MeOH with gradient from 97% to 90% in 10 min, 1.8 mL/min., 40 °C, detection at 324 nm. Retention time (min.): 3.95 (minor) and 4.20 (major).

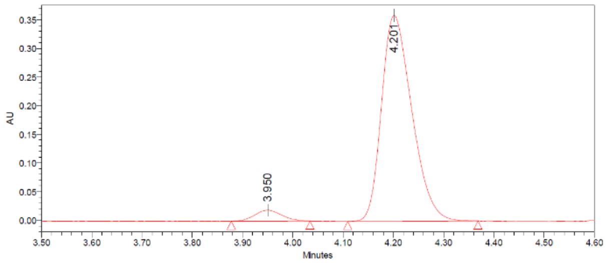

Peak Results

	Name	RT	Area	% Area	Height
1		3.950	70636	4.48	18923
2		4.201	1507713	95.52	359246

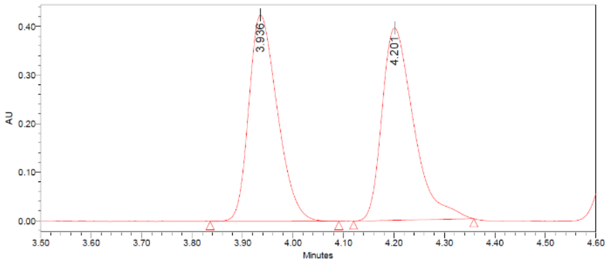

Peak Results

	Name	RT	Area	% Area	Height
1		3.936	1673881	49.23	424948
2		4.201	1726371	50.77	395885

SFC of (*R*)-1-benzyl-4-isopentyl-1,4-dihydropyridine-3-carbonitrile (**4g**)

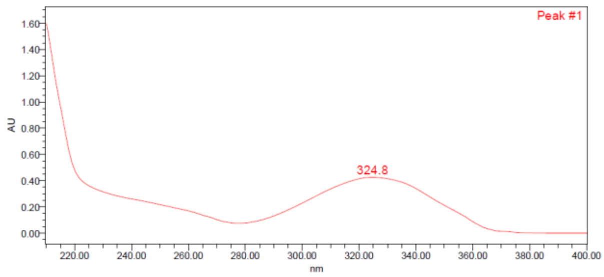

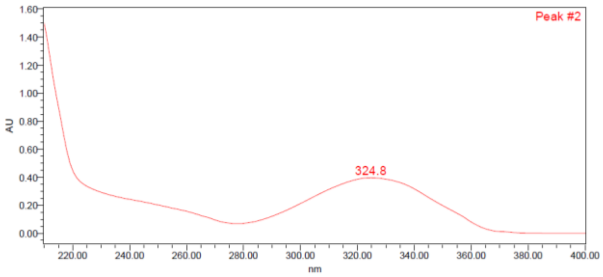

UV-visible spectra of (*R*)-1-benzyl-4-isopentyl-1,4-dihydropyridine-3-carbonitrile (**4g**)

(*R*)-1-Benzyl-4-isobutyl-1,4-dihydropyridine-3-carbonitrile (4h)

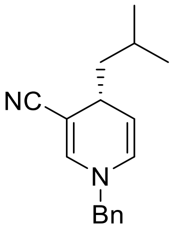

The reaction was performed with **1a** (55.0 mg, 0.2 mmol, 1.0 equiv.), CuTC (3.8 mg, 10.0 mol%), (*R*)-Tol-BINAP (16.3 mg, 12.0 mol%), ^{i}BuMgBr (2.0 M in Et_2O, 120 µL, 0.24 mmol, 1.2 equiv.) in CH_2Cl_2 (2.0 mL) at −78 °C for 16 h. Product **4h** was obtained as a yellow oil after column chromatography (SiO_2, pentane:EtOAc = 3:1) [>99% conversion, 44.0 mg, 87% yield, 77% *ee*, (*R*)-configuration].

^{1}H NMR ($CDCl_3$, 400 MHz): δ 7.40–7.30 (m, 3H, 3 × CH_{Ar}), 7.21–7.17 (m, 2H, 2 × CH_{Ar}), 6.60 (d, *J* = 1.7 Hz, 1H, C=C*H*), 5.76 (dt, *J* = 8.1 and 1.7 Hz, 1H, C*H*=CH), 4.72 (dd, *J* = 8.1 and 4.3 Hz, 1H, CH=C*H*), 4.29 (s, 2H, NCH_2), 3.21–3.16 (m, 1H, C*H*), 1.86–1.81 (m, 1H, C*H*), 1.46 (t, *J* = 6.6 Hz, 2H, CH_2), 0.94 (d, *J* = 6.6 Hz, 3H, CH_3), 0.89 (d, *J* = 6.6 Hz, 3H, CH_3).

^{13}C NMR ($CDCl_3$, 101 MHz): δ 142.8, 136.6, 129.1 (2 × C), 128.2, 127.7, 127.2 (2 × C), 121.7, 106.5, 83.7, 57.5, 48.9, 31.1, 23.6, 23.5, 22.1.

LC-HRMS (ESI-TOF): m/z $[M+H]^+$ calcd. for $C_{17}H_{21}N_2^+$: 253.1699; found 253.1699.

SFC: Trefoil CEL2, CO_2/MeOH with gradient from 97% to 90% in 10 min, 1.8 mL/min., 40 °C, detection at 324 nm. Retention time (min.): 3.72 (minor) and 3.94 (major).

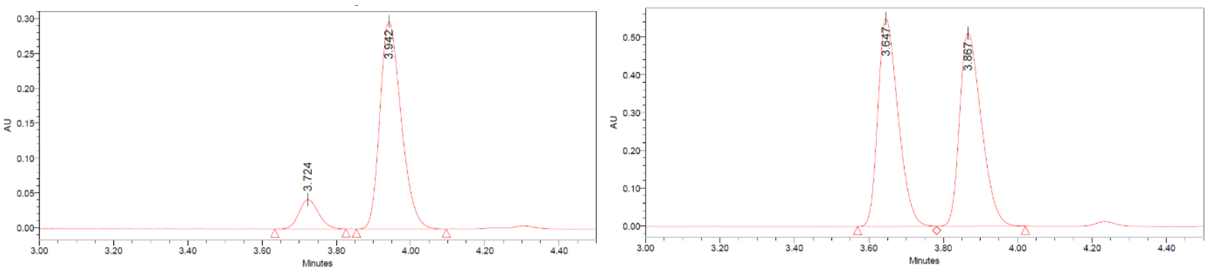

Peak Results

	Name	RT	Area	% Area	Height
1		3.724	154411	11.52	41927
2		3.942	1186339	88.48	297645

Peak Results

	Name	RT	Area	% Area	Height
1		3.647	2076668	49.96	550279
2		3.867	2079817	50.04	510772

SFC of (*R*)-1-benzyl-4-isobutyl-1,4-dihydropyridine-3-carbonitrile (**4h**)

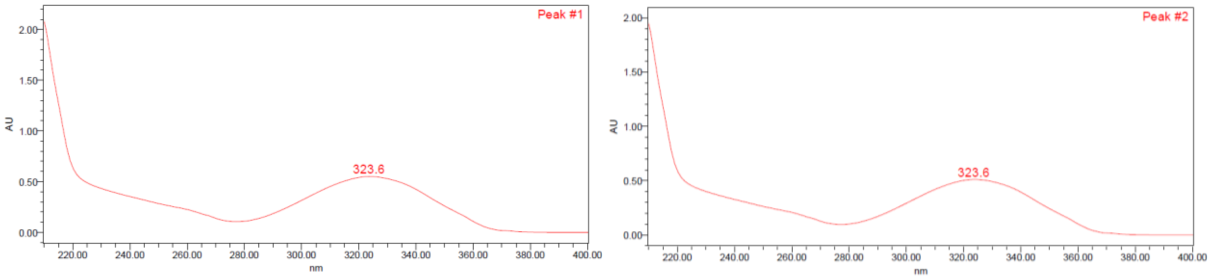

UV-visible spectra of (*R*)-1-benzyl-4-isobutyl-1,4-dihydropyridine-3-carbonitrile (**4h**)

(*R*)-1-Benzyl-4-(cyclohexylmethyl)-1,4-dihydropyridine-3-carbonitrile (4i)

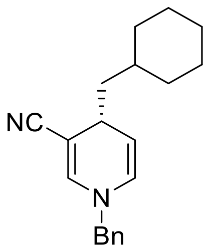

The reaction was performed with **1a** (55.0 mg, 0.2 mmol, 1.0 equiv.), CuTC (3.8 mg, 10.0 mol%), (*R*)-Tol-BINAP (16.3 mg, 12.0 mol%), CyclohexCH$_2$MgBr (2.3 M in Et$_2$O, 104 μL, 0.24 mmol, 1.2 equiv.) in CH$_2$Cl$_2$ (2.0 mL) at −78 °C for 16 h. Product **4i** was obtained as a yellow oil after column chromatography (SiO$_2$, pentane:EtOAc = 3:1) [>99% conversion, 43.3 mg, 74% yield, 56% *ee*, (*R*)-configuration].

^{1}H NMR (CDCl$_3$, 400 MHz): δ 7.40–7.30 (m, 3H, 3 × C*H*$_{Ar}$), 7.20–7.18 (m, 2H, 2 × C*H*$_{Ar}$), 6.59 (d, *J* = 1.6 Hz, 1H, C=C*H*), 5.76 (dt, *J* = 8.1 and 1.2 Hz, 1H, C*H*=CH), 4.71 (dd, *J* = 8.1 and 4.2 Hz, 1H, CH=C*H*), 4.29 (s, 2H, NC*H*$_2$), 3.25–3.21 (m, 1H, C*H*), 1.74–1.50 (m, 8H, 4 × C*H*$_2$), 1.21–1.40 (m, 3H, C*H* and C*H*$_2$), 0.99–0.83 (m, 2H, C*H*$_2$).

^{13}C NMR (CDCl$_3$, 101 MHz): δ 142.8, 136.7, 129.1 (2 × C), 128.2, 127.7, 127.2 (2 × C), 121.7, 106.7, 83.8, 57.5, 47.3, 34.2, 33.2, 33.0, 30.5, 26.7, 26.5, 26.4.

LC-HRMS (ESI-TOF): m/z [M+H]$^+$ calcd. for $C_{20}H_{24}N_2H^+$: 293.2012; found 293.1954.

SFC: Trefoil CEL2, CO$_2$/MeOH with gradient from 97% to 90% in 10 min, 1.8 mL/min., 40 °C, detection at 324 nm. Retention time (min.): 6.03 (minor) and 6.34 (major).

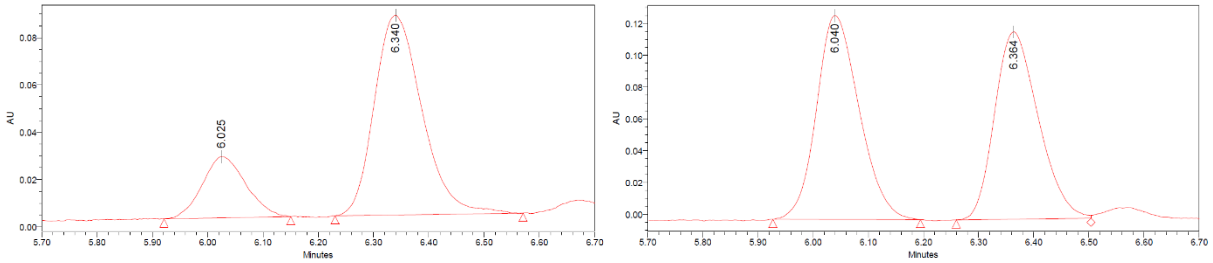

Peak Results

	Name	RT	Area	% Area	Height
1		6.025	141649	21.87	25925
2		6.340	506156	78.13	84486

Peak Results

	Name	RT	Area	% Area	Height
1		6.040	689958	51.59	127916
2		6.364	647332	48.41	117614

SFC of (*R*)-1-benzyl-4-(cyclohexylmethyl)-1,4-dihydropyridine-3-carbonitrile (**4i**)

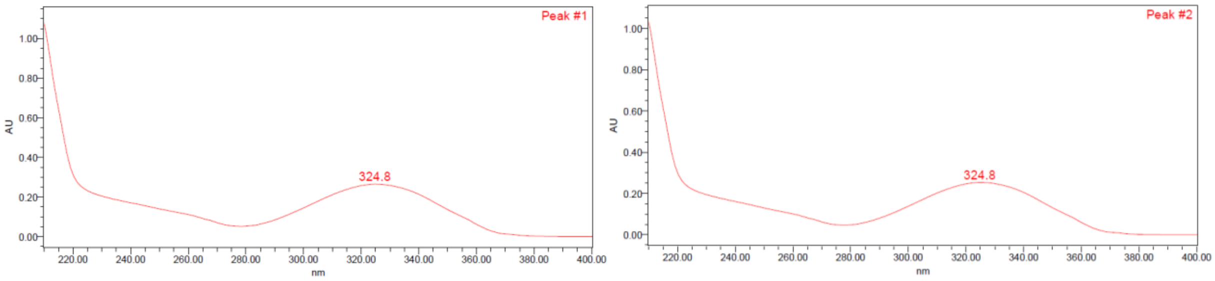

UV-visible spectra of (*R*)-1-benzyl-4-(cyclohexylmethyl)-1,4-dihydropyridine-3-carbonitrile (**4i**)

(*R*)-1-Benzyl-4-cyclopentyl-1,4-dihydropyridine-3-carbonitrile (4j)

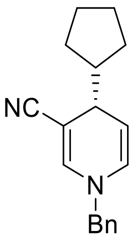

The reaction was performed with **1a** (55.0 mg, 0.2 mmol, 1.0 equiv.), CuTC (7.6 mg, 20.0 mol%), (*R*)-Tol-BINAP (32.6 mg, 24.0 mol%), CyclopentMgCl (2.0 M in Et_2O, 120 µL, 0.24 mmol, 1.2 equiv.) in CH_2Cl_2 (2.0 mL) at −78 °C for 16 h. Product **4j** was obtained as a yellow oil after column chromatography (SiO_2, pentane:EtOAc = 3:1) [>99% conversion, 48.9 mg, 93% yield, 47% *ee*, (*R*)-configuration].

^{1}H NMR ($CDCl_3$, 400 MHz): δ 7.39–7.29 (m, 3H, 3 × CH_{Ar}), 7.20–7.18 (m, 2H, 2 × CH_{Ar}), 6.63 (d, *J* = 1.7 Hz, 1H, C=C*H*), 5. 48 (dt, *J* = 8.1 and 1.4 Hz, 1H, C*H*=CH), 4.68 (dd, *J* = 8.1 and 4.5 Hz, 1H, CH=C*H*), 4.29 (s, 2H, NCH_2), 3.19 (t, *J* = 4.9 Hz, 1H, C*H*), 2.09–2.01 (m, 1H, CH_{cyclo}), 1.77–1.69 (m, 2H, 2 × CH_{cyclo}), 1.66–1.48 (m, 4H, 4 × CH_{cyclo}), 1.45–1.30 (m, 1H, 2 × CH_{cyclo}).

^{13}C NMR ($CDCl_3$, 101 MHz): δ 143.2, 136.6, 129.1 (2 × C), 128.8, 128.2, 127.2 (2 × C), 122.1, 104.5, 83.0, 57.5, 47.7, 37.0, 28.6, 28.0, 25.7, 25.3.

LC-HRMS (ESI-TOF): m/z $[M+H]^+$ calcd. for $C_{18}H_{21}N_2^+$: 265.1699; found 265.1695.

SFC: Trefoil CEL2, CO_2/MeOH with gradient from 97% to 90% in 10 min, 1.8 mL/min., 40 °C, detection at 324 nm. Retention time (min.): 5.92 (major) and 6.18 (minor).

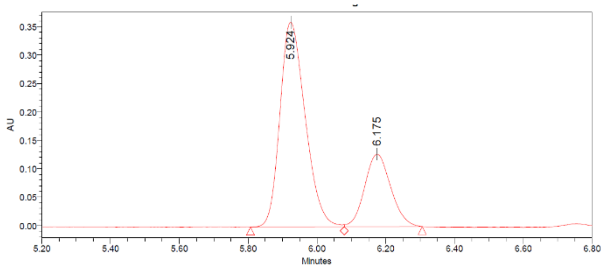

Peak Results

	Name	RT	Area	% Area	Height
1		5.924	1838963	73.26	359893
2		6.175	671063	26.74	127470

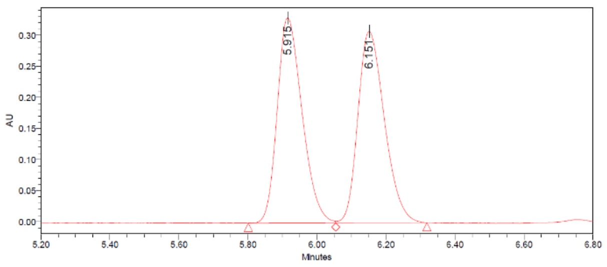

Peak Results

	Name	RT	Area	% Area	Height
1		5.915	1654548	50.17	330196
2		6.151	1643526	49.83	308056

SFC of (*R*)-1-benzyl-4-cyclopentyl-1,4-dihydropyridine-3-carbonitrile (**4j**)

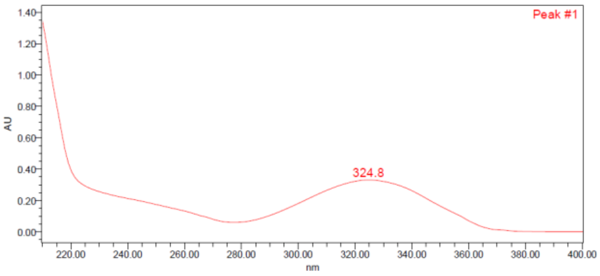

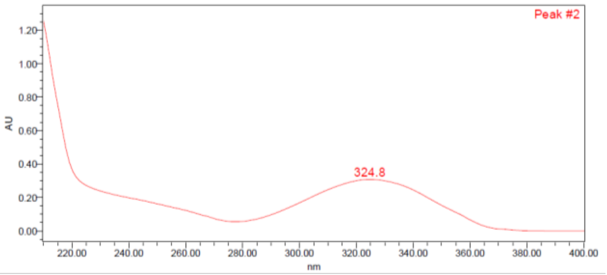

UV-visible spectra of (*R*)-1-benzyl-4-cyclopentyl-1,4-dihydropyridine-3-carbonitrile (**4j**)

(*R*)-1-Benzyl-4-phenethyl-1,4-dihydropyridine-3-carbonitrile (4k)

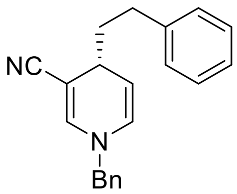

The reaction was performed with **1a** (55.0 mg, 0.2 mmol, 1.0 equiv.), CuTC (3.8 mg, 10.0 mol%), (*R*)-Tol-BINAP (16.3 mg, 12.0 mol%), $Ph(CH_2)_2MgBr$ (2.5 M in Et_2O, 96 μL, 0.24 mmol, 1.2 equiv.) in CH_2Cl_2 (2.0 mL) at −78 °C for 16 h. Product **4k** was obtained as a yellow oil after column chromatography (SiO_2, pentane:EtOAc = 3:1) [>99% conversion, 50.6 mg, 84% yield, 88% *ee*, *(R)-configuration]*.

^{1}H NMR ($CDCl_3$, 400 MHz): δ 7.41–7.27 (m, 5H, 5 × C*H*$_{Ar}$), 7.23–7.16 (m, 5H, 5 × C*H*$_{Ar}$), 6.67 (d, *J* = 1.6 Hz, 1H, C=C*H*), 5.85 (dt, *J* = 8.1 and 1.4 Hz, 1H, C*H*=CH), 4.71 (dd, *J* = 8.1 and 4.1 Hz, 1H, CH=C*H*), 4.31 (s, 2H, NC*H*$_2$), 3.35–3.31 (m, 1H, C*H*), 2.80–2.63 (m, 2H, C*H*$_2$), 1.94–1.76 (m, 2H, C*H*$_2$).

^{13}C NMR ($CDCl_3$, 101 MHz): δ 143.3, 142.2, 136.5, 129.2 (2 × C), 128.6 (2 × C), 128.5, 128.5 (2 × C), 128.3, 127.2 (2 × C), 125.9, 121.4, 106.0, 82.6, 57.6, 39.6, 33.0, 31.5.

LC-HRMS (ESI-TOF): m/z $[M+H]^+$ calcd. for $C_{21}H_{21}N_2^+$: 301.1699; found 301.1698.

SFC: Trefoil CEL2, CO_2/MeOH with gradient from 97% to 90% in 10 min, 1.8 mL/min., 40 °C, detection at 324 nm. Retention time (min.): 7.56 (minor) and 7.98 (major).

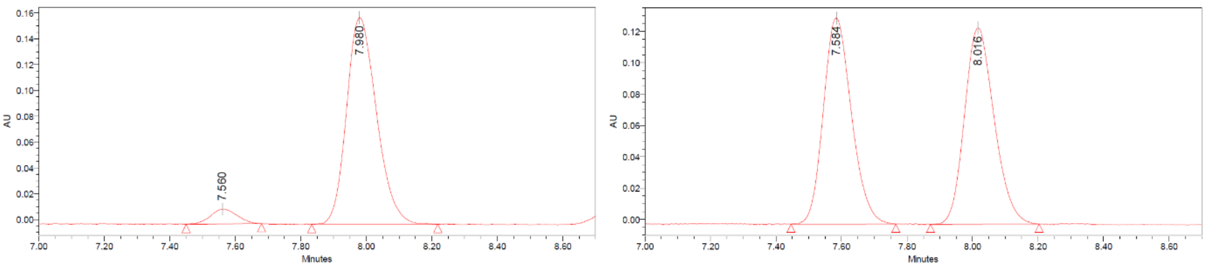

Peak Results

	Name	RT	Area	% Area	Height
1		7.560	64756	5.97	11511
2		7.980	1019780	94.03	160198

Peak Results

	Name	RT	Area	% Area	Height
1		7.584	786015	49.90	131494
2		8.016	789124	50.10	125083

SFC of (*R*)-1-benzyl-4-phenethyl-1,4-dihydropyridine-3-carbonitrile (**4k**)

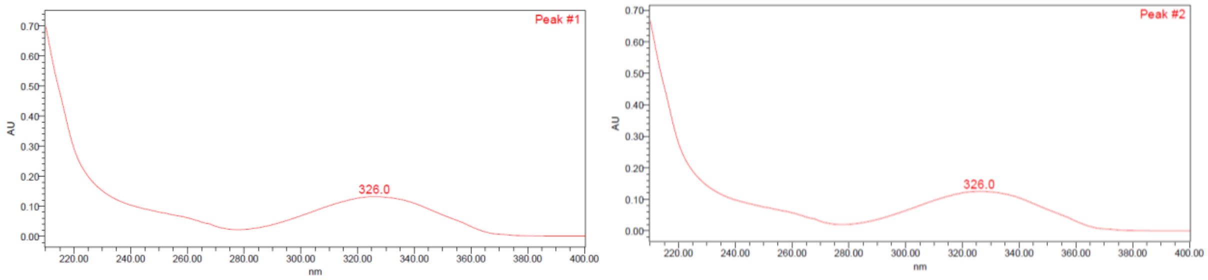

UV-visible spectra of (*R*)-1-benzyl-4-phenethyl-1,4-dihydropyridine-3-carbonitrile (**4k**)

(*R*)-1-Benzyl-4-(3-phenylpropyl)-1,4-dihydropyridine-3-carbonitrile (4l)

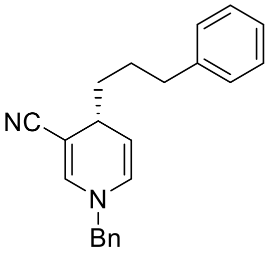

The reaction was performed with **1a** (55.0 mg, 0.2 mmol, 1.0 equiv.), CuTC (3.8 mg, 10.0 mol%), (*R*)-Tol-BINAP (16.3 mg, 12.0 mol%), $Ph(CH_2)_3MgBr$ (3.3 M in Et_2O, 73 µL, 0.24 mmol, 1.2 equiv.) in CH_2Cl_2 (2.0 mL) at −78 °C for 16 h. Product **4l** was obtained as a yellow oil after column chromatography (SiO_2, pentane:EtOAc = 3:1) [>99% conversion, 49.7 mg, 79% yield, 85% *ee*, (*R*)-configuration].

^{1}H NMR ($CDCl_3$, 400 MHz): δ 7.35–7.28 (m, 5H, 5 × CH_{Ar}), 7.22–7.15 (m, 5H, 5 × CH_{Ar}), 6.62 (d, *J* = 1.6 Hz, 1H, C=C*H*), 5.78 (dt, *J* = 8.1 and 1.3 Hz, 1H, C*H*=CH), 4.63 (dd, *J* = 8.1 and 4.1 Hz, 1H, CH=C*H*), 4.26 (s, 2H, NCH_2), 3.31–3.27 (m, 1H, C*H*), 2.69–2.63 (m, 2H, CH_2), 1.83–1.49 (m, 4H, 2 × CH_2).

^{13}C NMR ($CDCl_3$, 101 MHz): δ 143.2, 142.6, 136.5, 129.1 (2 × C), 128.5 (2 × C), 128.4 (2 × C), 128.4, 128.2, 127.2 (2 × C), 125.8, 121.5, 106.2, 82.7, 57.5, 37.5, 36.1, 33.1, 27.2.

LC-HRMS (ESI-TOF): m/z $[M+H]^+$ calcd. for $C_{22}H_{23}N_2^+$: 315.1856; found 315.2514.

SFC: Trefoil CEL2, CO_2/MeOH with gradient from 97% to 90% in 10 min, 1.8 mL/min., 40 °C, detection at 324 nm. Retention time (min.): 7.88 (minor) and 8.19 (major).

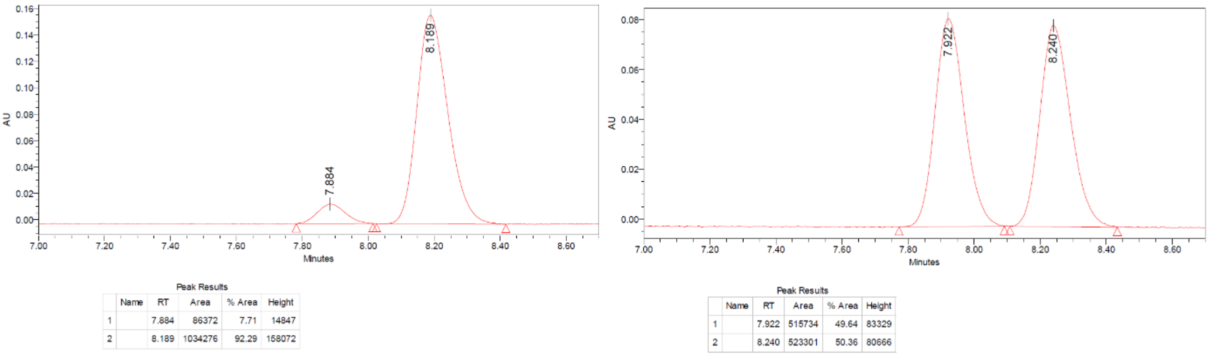

SFC of (*R*)-1-benzyl-4-(3-phenylpropyl)-1,4-dihydropyridine-3-carbonitrile (**4l**)

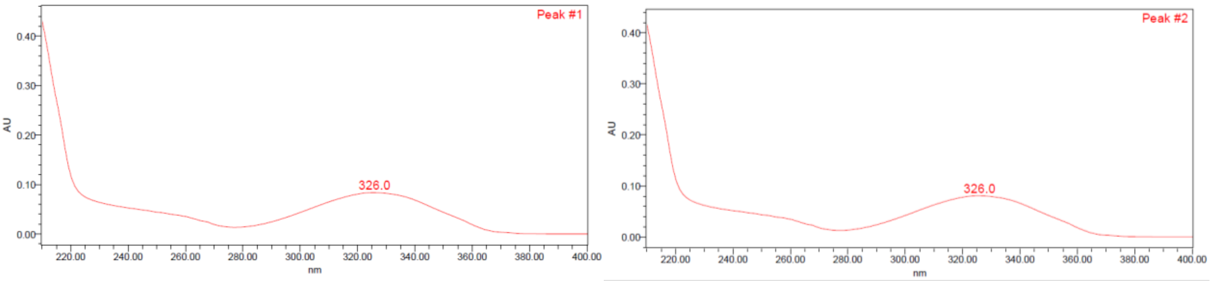

UV-visible spectra of (*R*)-1-benzyl-4-(3-phenylpropyl)-1,4-dihydropyridine-3-carbonitrile (**4l**)

(*R*)-1-Benzyl-4-(but-3-en-1-yl)-1,4-dihydropyridine-3-carbonitrile (4m)

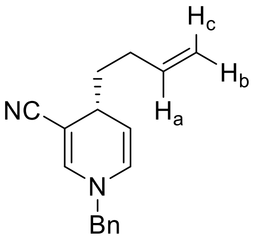

The reaction was performed with **1a** (55.0 mg, 0.2 mmol, 1.0 equiv.), CuTC (3.8 mg, 10.0 mol%), (*R*)-Tol-BINAP (16.3 mg, 12.0 mol%), $CH_2=CH(CH_2)_2MgBr$ (2.5 M in Et_2O, 96 µL, 0.24 mmol, 1.2 equiv.) in CH_2Cl_2 (2.0 mL) at −78 °C for 16 h. Product **4m** was obtained as a yellow oil after column chromatography (SiO_2, pentane:EtOAc = 3:1) [>99% conversion, 42.6 mg, 85% yield, 86% *ee*, (*R*)-configuration].

^{1}H NMR ($CDCl_3$, 400 MHz): δ 7.40–7.30 (m, 3H, 3 × CH_{Ar}), 7.21–7.18 (m, 2H, 2 × CH_{Ar}), 6.63 (d, *J* = 1.6 Hz, 1H, C=C*H*), 5.89–5.79 (m, 2H, C*H*=CH and CH_a=CH$_b$H$_c$), 5.05 (dq, *J* = 17.1 and 1.6 Hz, 1H, CH$_a$=CH$_b$$H_c$), 4.97 (dq, *J* = 10.2 and 1.6 Hz, 1H, CH$_a$=CH_bH$_c$), 4.66 (dd, *J* = 8.1 and 4.2 Hz, 1H, CH=C*H*), 4.29 (s, 2H, NCH_2), 3.29–3.25 (m, 1H, C*H*), 2.24–2.04 (m, 2H, CH_2), 1.70–1.54 (m, 2H, CH_2).

^{13}C NMR ($CDCl_3$, 101 MHz): δ 143.2, 138.4, 136.5, 129.1 (2 × C), 128.4, 128.3, 127.2 (2 × C), 121.4, 114.9, 106.0, 82.7, 57.5, 37.1, 32.8, 29.4.

LC-HRMS (ESI-TOF): m/z $[M+H]^+$ calcd. for $C_{17}H_{19}N_2^+$: 251.1543; found 251.1541.

SFC: Trefoil CEL2, CO_2/MeOH with gradient from 97% to 90% in 10 min, 1.8 mL/min., 40 °C, detection at 324 nm. Retention time (min.): 4.16 (minor) and 4.37 (major).

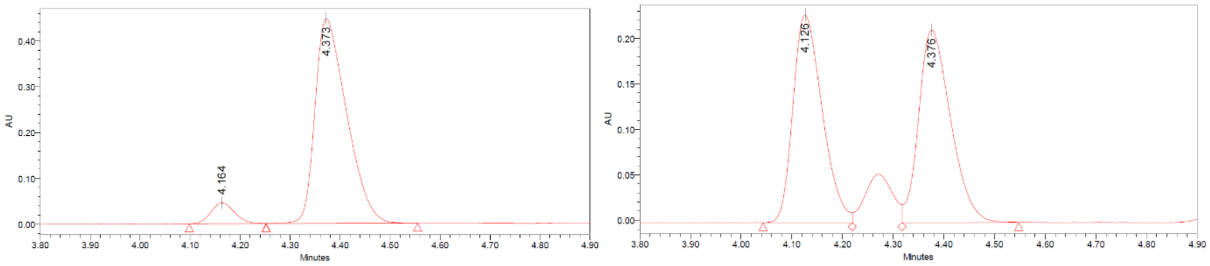

Peak Results

	Name	RT	Area	% Area	Height
1		4.164	148519	7.04	45253
2		4.373	1962162	92.96	446279

Peak Results

	Name	RT	Area	% Area	Height
1		4.126	915745	49.64	228109
2		4.376	928974	50.36	211446

SFC of (*R*)-1-benzyl-4-(but-3-en-1-yl)-1,4-dihydropyridine-3-carbonitrile (**4m**)

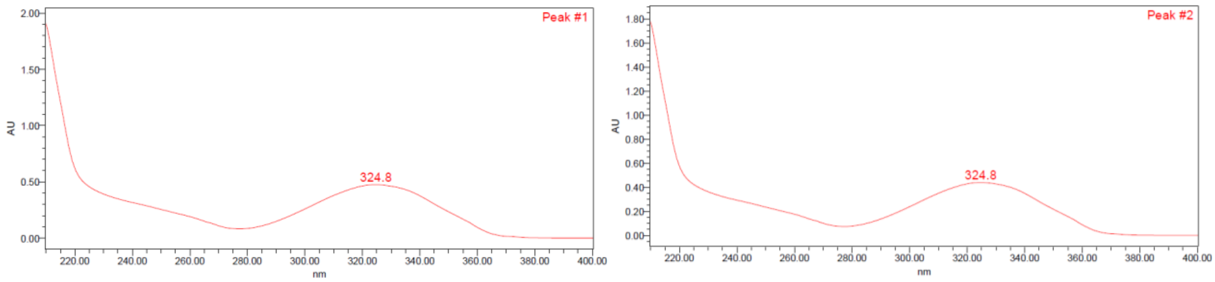

UV-visible spectra of (*R*)-1-benzyl-4-(but-3-en-1-yl)-1,4-dihydropyridine-3-carbonitrile (**4m**)

(*R*)-1-Benzyl-4-(hex-5-en-1-yl)-1,4-dihydropyridine-3-carbonitrile (4n)

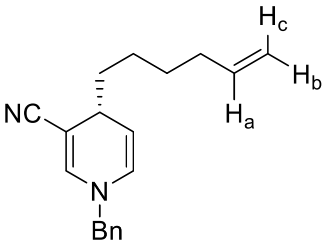

The reaction was performed with **1a** (55.0 mg, 0.2 mmol, 1.0 equiv.), CuTC (3.8 mg, 10.0 mol%), (*R*)-Tol-BINAP (16.3 mg, 12.0 mol%), CH_2=CH(CH_2)$_4$MgBr (1.6 M in Et_2O, 150 μL, 0.24 mmol, 1.2 equiv.) in CH_2Cl_2 (2.0 mL) at −78 °C for 16 h. Product **4n** was obtained as a yellow oil after column chromatography (SiO_2, pentane:EtOAc = 3:1) [>99% conversion, 45.0 mg, 81% yield, 75% *ee*, (*R*)-configuration].

^{1}H NMR ($CDCl_3$, 400 MHz): δ 7.39–7.30 (m, 3H, 3 × CH_{Ar}), 7.20–7.18 (m, 2H, 2 × CH_{Ar}), 6.61 (d, *J* = 1.5 Hz, 1H, C=C*H*), 5.85–5.76 (m, 2H, C*H*=CH and CH_a=CH$_b$H$_c$), 5.00 (dq, *J* = 17.1 and 2.3 Hz, 1H, CH$_a$=CH$_b$$H_c$), 4.97 (dq, *J* = 10.2 and 2.3 Hz, 1H, CH$_a$=CH_bH$_c$), 4.64 (dd, *J* = 8.1 and 4.1 Hz, 1H, CH=C*H*), 4.28 (s, 2H, NCH_2), 3.25–3.22 (m, 1H, C*H*), 2.08–2.04 (m, 2H, CH_2), 1.62–1.33 (m, 6H, 3 × CH_2).

^{13}C NMR ($CDCl_3$, 101 MHz): δ 143.1, 139.1, 136.6, 129.1 (2 × C), 128.3, 128.2, 127.2 (2 × C), 121.5, 114.5, 106.4, 82.9, 57.5, 37.8, 33.9, 33.1, 29.1, 24.6.

LC-HRMS (APCI): m/z $[M+H]^+$ calcd. for $C_{19}H_{23}N_2^+$: 279.1856; found 279.1497.

SFC: Trefoil CEL2, CO_2/MeOH with gradient from 97% to 90% in 10 min, 1.8 mL/min., 40 °C, detection at 324 nm. Retention time (min.): 4.55 (minor) and 4.81 (major).

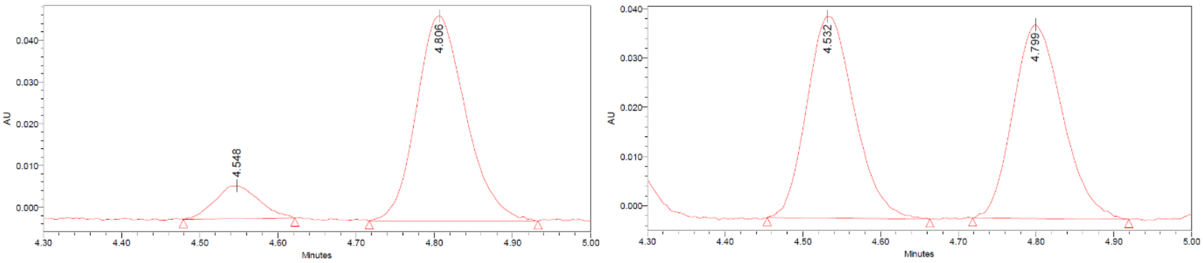

Peak Results

	Name	RT	Area	% Area	Height
1		4.548	31002	12.62	7891
2		4.806	214647	87.38	49048

Peak Results

	Name	RT	Area	% Area	Height
1		4.532	174267	50.03	40913
2		4.799	174081	49.97	39297

SFC of (*R*)-1-Benzyl-4-(hex-5-en-1-yl)-1,4-dihydropyridine-3-carbonitrile (**4n**)

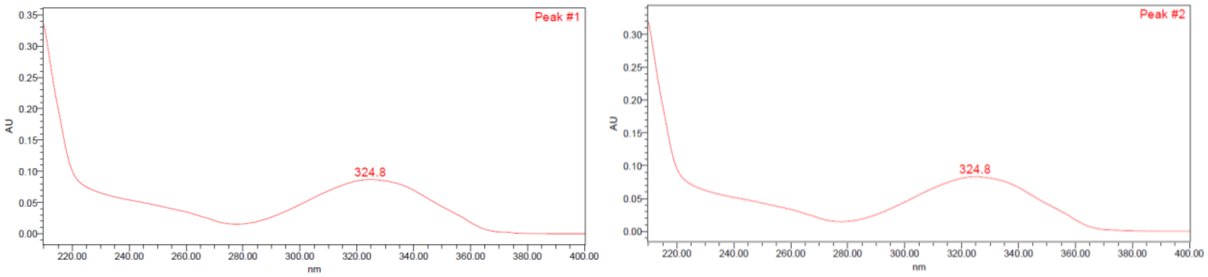

UV-visible spectra of (*R*)-1-Benzyl-4-(hex-5-en-1-yl)-1,4-dihydropyridine-3-carbonitrile (**4n**)

(*R*)-1-Benzyl-4-(4-chlorobutyl)-1,4-dihydropyridine-3-carbonitrile (4o)

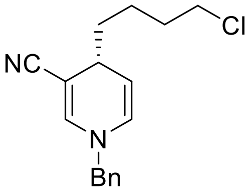

The reaction was performed with **1a** (55.0 mg, 0.2 mmol, 1.0 equiv.), CuTC (7.6 mg, 20.0 mol%), (*R*)-Tol-BINAP (16.3 mg, 12.0 mol%), $ClCH_2(CH_2)_3MgBr$ (2.3 M in Et_2O, 104 μL, 0.24 mmol, 1.2 equiv.) in CH_2Cl_2 (2.0 mL) at −78 °C for 16 h. Product **4o** was obtained as a yellow oil after column chromatography (SiO_2, pentane:EtOAc = 3:1) [>99% conversion, 38.5 mg, 67% yield, 79% *ee*, (*R*)-configuration].

^{1}H NMR ($CDCl_3$, 400 MHz): δ 7.40–7.32 (m, 3H, 3 × CH_{Ar}), 7.21–7.18 (m, 2H, 2 × CH_{Ar}), 6.64 (d, *J* = 1.7 Hz, 1H, C=C*H*), 5.81 (d, *J* = 8.1 Hz, 1H, C*H*=CH), 4.64 (dd, *J* = 8.1 and 4.1 Hz, 1H, CH=C*H*), 4.29 (s, 2H, NCH_2), 3.55 (t, *J* = 6.6 Hz, 2H, CH_2Cl), 3.30–3.27 (m, 1H, C*H*), 1.81 (p, *J* = 7.0 Hz, 2H, CH_2), 1.62–1.47 (m, 4H, 2 × CH_2).

^{13}C NMR ($CDCl_3$, 101 MHz): δ 143.2, 136.5, 129.2 (2 × C), 128.5, 128.3, 127.2 (2 × C), 121.4, 116.0, 82.9, 57.6, 45.2, 36.9, 33.1, 32.7, 22.5.

LC-HRMS (ESI-TOF): m/z $[M+H]^+$ calcd. for $C_{17}H_{20}ClN_2^+$: 287.1310; found 287.1122.

SFC: Trefoil CEL2, CO_2/MeOH with gradient from 97% to 90% in 10 min, 1.8 mL/min., 40 °C, detection at 324 nm. Retention time (min.): 5.86 (minor) and 6.23 (major).

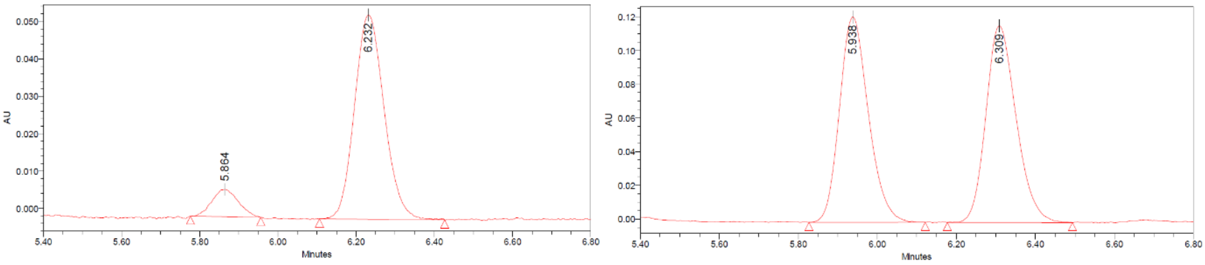

Peak Results

	Name	RT	Area	% Area	Height
1		5.864	34321	10.66	7291
2		6.232	287688	89.34	54608

Peak Results

	Name	RT	Area	% Area	Height
1		5.938	620116	49.91	121858
2		6.309	622245	50.09	116684

SFC of (*R*)-1-Benzyl-4-(4-chlorobutyl)-1,4-dihydropyridine-3-carbonitrile (**4o**)

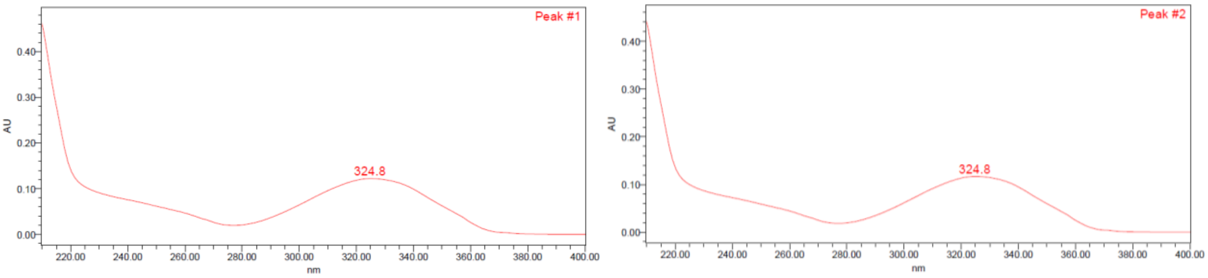

UV-visible spectra of (*R*)-1-benzyl-4-(4-chlorobutyl)-1,4-dihydropyridine-3-carbonitrile (**4o**)

(*R*)-1-Benzyl-4-(6-chlorohexyl)-1,4-dihydropyridine-3-carbonitrile (4p)

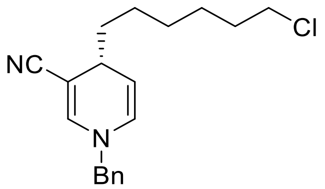

The reaction was performed with **1a** (55.0 mg, 0.2 mmol, 1.0 equiv.), CuTC (7.6 mg, 20.0 mol%), (*R*)-Tol-BINAP (16.3 mg, 12.0 mol%), $ClCH_2(CH_2)_5MgBr$ (2.2 M in Et_2O, 109 µL, 0.24 mmol, 1.2 equiv.) in CH_2Cl_2 (2.0 mL) at −78 °C for 16 h. Product **4p** was obtained as a yellow oil after column chromatography (SiO_2, pentane:EtOAc = 3:1) [>99% conversion, 44.0 mg, 70% yield, 88% *ee*, (*R*)-configuration].

^{1}H NMR ($CDCl_3$, 400 MHz): δ 7.40–7.32 (m, 3H, 3 × CH_{Ar}), 7.20–7.18 (m, 2H, 2 × CH_{Ar}), 6.62 (d, *J* = 1.6 Hz, 1H, C=C*H*), 5.79 (dt, *J* = 8.2 and 1.3 Hz, 1H, C*H*=CH), 4.64 (dd, *J* = 8.2 and 4.1 Hz, 1H, CH=C*H*), 4.28 (s, 2H, NCH_2), 3.54 (t, *J* = 6.6 Hz, 2H, CH_2Cl), 3.26–3.22 (m, 1H, C*H*), 1.77 (p, *J* = 6.9 Hz, 2H, CH_2), 1.62–1.25 (m, 8H, 4 × CH_2).

^{13}C NMR ($CDCl_3$, 101 MHz): δ 143.1, 136.6, 129.1 (2 × C), 128.3, 128.3, 127.2 (2 × C), 121.5, 106.3, 82.9, 57.6, 45.3, 37.8, 33.1, 32.7, 29.1, 27.0, 24.9.

LC-HRMS (ESI-TOF): m/z $[M+H]^+$ calcd. for $C_{19}H_{24}ClN_2^+$: 315.1623; found 315.1434.

SFC: Trefoil CEL2, CO_2/MeOH with gradient from 97% to 90% in 10 min, 1.8 mL/min., 40 °C, detection at 324 nm. Retention time (min.): 6.64 (minor) and 6.97 (major).

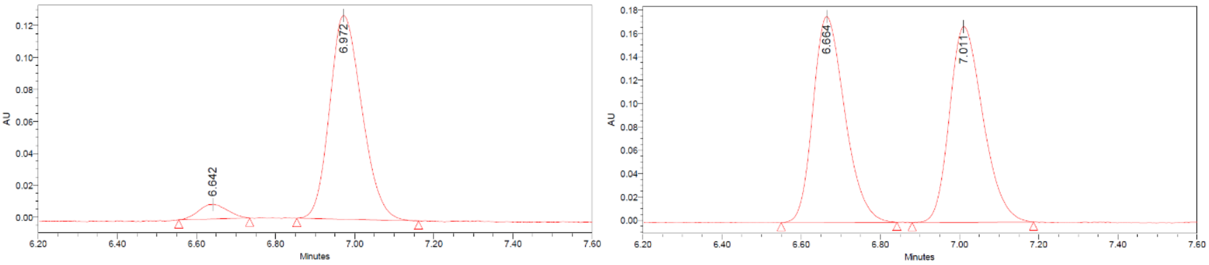

Peak Results

	Name	RT	Area	% Area	Height
1		6.642	46270	6.02	9133
2		6.972	722671	93.98	127583

Peak Results

	Name	RT	Area	% Area	Height
1		6.664	985164	50.00	176366
2		7.011	985270	50.00	167732

SFC of (*R*)-1-Benzyl-4-(6-chlorohexyl)-1,4-dihydropyridine-3-carbonitrile (**4p**)

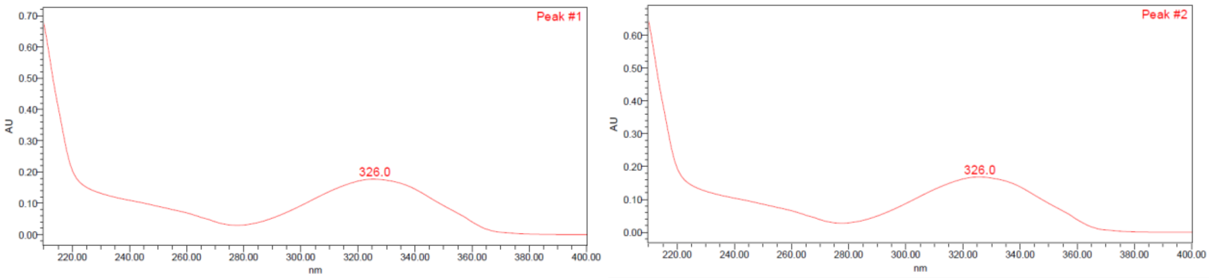

UV-visible spectra of (*R*)-1-Benzyl-4-(6-chlorohexyl)-1,4-dihydropyridine-3-carbonitrile (**4p**)

(*Rac*)-1-Benzyl-4-isopropyl-1,4-dihydropyridine-3-carbonitrile (4q)

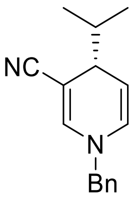

The reaction was performed with **1a** (55.0 mg, 0.2 mmol, 1.0 equiv.), CuTC (3.8 mg, 10.0 mol%), (*R*)-Tol-BINAP (16.3 mg, 12.0 mol%), ^{i}PrMgBr (3.0 M in Et_2O, 80 µL, 0.24 mmol, 1.2 equiv.) in CH_2Cl_2 (2.0 mL) at −78 °C for 16 h. Product **4q** was obtained as a yellow oil after column chromatography (SiO_2, pentane:EtOAc = 3:1) [>99% conversion, 45.7 mg, 96% yield, <5% *ee*].

^{1}H NMR ($CDCl_3$, 400 MHz): δ 7.39–7.29 (m, 3H, 3 × CH_{Ar}), 7.20–7.18 (m, 2H, 2 × CH_{Ar}), 6.68 (d, *J* = 1.6 Hz, 1H, C=C*H*), 5.86 (dt, *J* = 8.2 and 1.2 Hz, 1H, C*H*=CH), 4.61 (dd, *J* = 8.2 and 4.3 Hz, 1H, CH=C*H*), 4.28 (s, 2H, NCH_2), 3.16 (t, *J* = 6.6 Hz, 1H, C*H*), 1.81–1.72 (m, 1H, C*H*), 0.96 (d, *J* = 6.9 Hz, 3H, CH_3), 0.90 (d, *J* = 6.9 Hz, 3H, CH_3).

^{13}C NMR ($CDCl_3$, 101 MHz): δ 143.6, 136.6, 129.2, 129.1 (2 × C), 128.2, 127.2 (2 × C), 121.8, 103.2, 82.3, 57.6, 39.6, 33.7, 18.4, 17.8.

LC-HRMS (ESI-TOF): m/z [M+Na]$^+$ calcd. for $C_{16}H_{18}N_2Na^+$: 261.1362; found 261.1387.

SFC: Trefoil CEL2, CO_2/MeOH with gradient from 97% to 90% in 10 min, 1.8 mL/min., 40 °C, detection at 324 nm. Retention time (min.): 3.91 and 4.10.

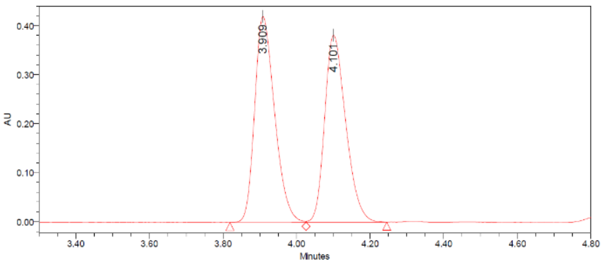

Peak Results

	Name	RT	Area	% Area	Height
1		3.909	1583419	50.98	420210
2		4.101	1522667	49.02	381729

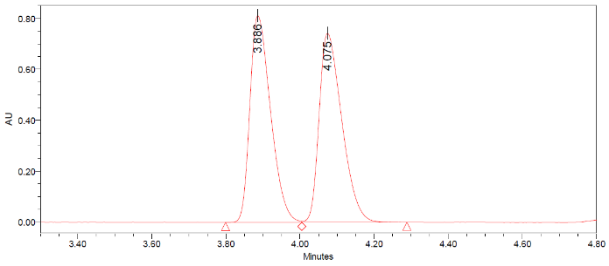

Peak Results

	Name	RT	Area	% Area	Height
1		3.886	3150889	49.94	811678
2		4.075	3158986	50.06	742522

SFC of (*rac*)-1-Benzyl-4-isopropyl-1,4-dihydropyridine-3-carbonitrile (**4q**)

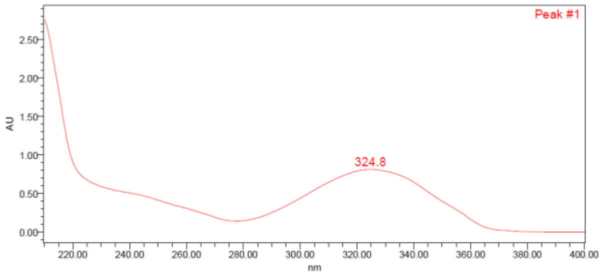

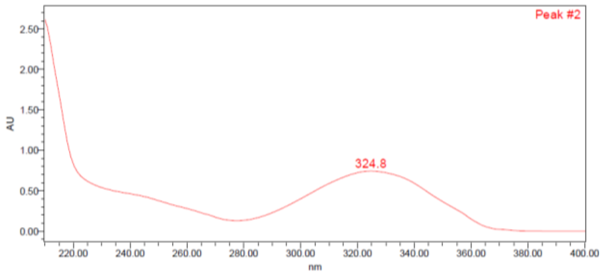

UV-visible spectra of (*rac*)-1-Benzyl-4-isopropyl-1,4-dihydropyridine-3-carbonitrile (**4q**)

(*Rac*)-4-Allyl-1-benzyl-1,4-dihydropyridine-3-carbonitrile (4r)

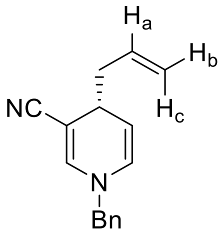

The reaction was performed with **1a** (55.0 mg, 0.2 mmol, 1.0 equiv.), CuTC (3.8 mg, 10.0 mol%), (*R*)-Tol-BINAP (16.3 mg, 12.0 mol%), AllylMgBr (1.0 M in Et_2O, 240 μL, 0.24 mmol, 1.2 equiv.) in CH_2Cl_2 (2.0 mL) at −78 °C for 16 h. Product **4r** was obtained as a yellow oil after column chromatography (SiO_2, pentane:EtOAc = 3:1) [>99% conversion, 22.1 mg, 47% yield, <5% *ee*].

^{1}H NMR ($CDCl_3$, 400 MHz): δ 7.40–7.32 (m, 3H, 3 × CH_{Ar}), 7.21–7.19 (m, 2H, 2 × CH_{Ar}), 6.62 (d, *J* = 1.6 Hz, 1H, C=C*H*), 5.87–5.76 (m, 2H, C*H*=CH and CH_a=C$H_b$$H_c$), 5.14–5.09 (m, 2H, C$H_a$=C$H_b$$H_c$), 4.67 (dd, *J* = 8.1 and 4.1 Hz, 1H, CH=C*H*), 4.28 (s, 2H, NCH_2), 3.3 (q, *J* = 5.1 Hz, 1H, C*H*), 2.30 (t, *J* = 6.4 Hz, 2H, CH_2).

^{13}C NMR ($CDCl_3$, 101 MHz): δ 143.2, 136.5, 134.5, 129.1 (2 × C), 128.3, 128.3, 127.3 (2 × C), 121.3, 118.0, 105.8, 82.5, 57.6, 42.4, 33.3.

LC-HRMS (ESI-TOF): m/z $[M+H]^+$ calcd. for $C_{16}H_{17}N_2^+$: 237.1386; found 237.1387.

SFC: Trefoil CEL2, CO_2/MeOH with gradient from 97% to 90% in 10 min, 1.8 mL/min., 40 °C, detection at 324 nm. Retention time (min.): 3.99 and 4.16.

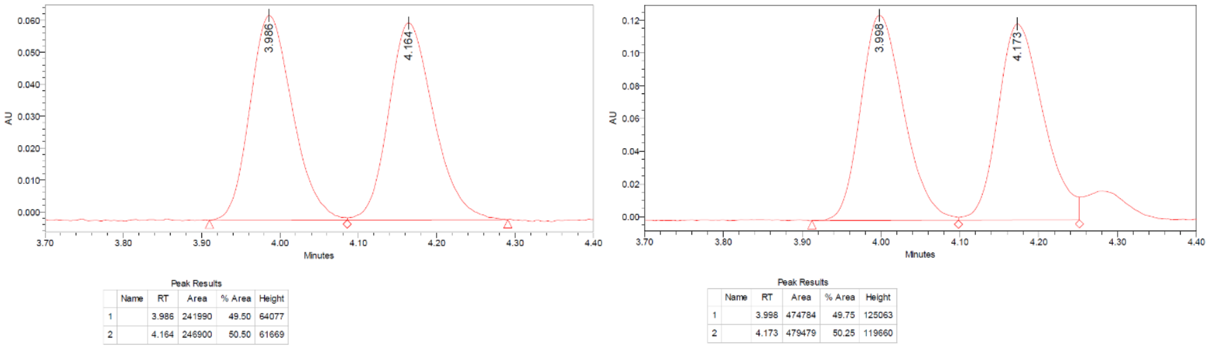

SFC of (*rac*)-4-Allyl-1-benzyl-1,4-dihydropyridine-3-carbonitrile (**4r**)

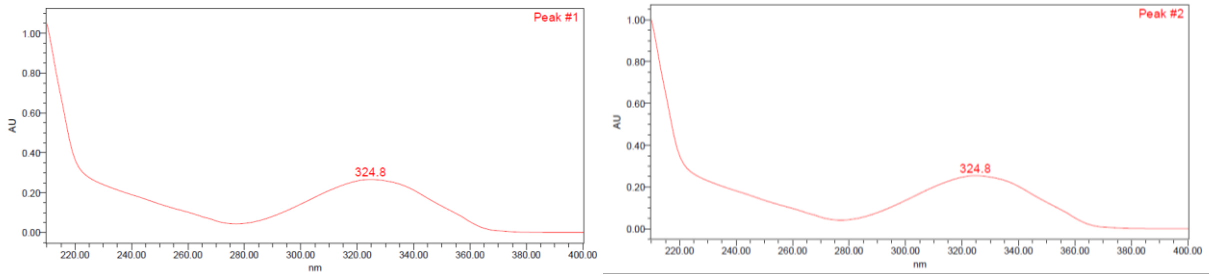

UV-visible spectra of (*rac*)-4-allyl-1-benzyl-1,4-dihydropyridine-3-carbonitrile (**4r**)

(*Rac*)-1-Benzyl-4-phenyl-1,4-dihydropyridine-3-carbonitrile (4s)

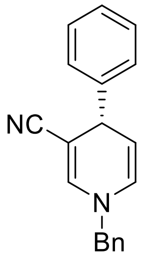

The reaction was performed with **1a** (55.0 mg, 0.2 mmol, 1.0 equiv.), CuTC (3.8 mg, 10.0 mol%), (*R*)-Tol-BINAP (16.3 mg, 12.0 mol%), PhMgBr (2.0 M in Et_2O, 120 µL, 0.24 mmol, 1.2 equiv.) in CH_2Cl_2 (2.0 mL) at −78 °C for 16 h. Product **4s** was obtained as a yellow oil after column chromatography (SiO_2, pentane:EtOAc = 3:1) [>99% conversion, 36.4 mg, 67% yield, <5% *ee*].

^{1}H NMR ($CDCl_3$, 400 MHz): δ 7.41–7.33 (m, 8H, 8 × CH_{Ar}), 7.22–7.20 (m, 2H, 2 × CH_{Ar}), 6.96 (d, *J* = 1.4 Hz, 1H, C=C*H*), 5.95–5.91 (m, 1H, C*H*=CH), 5.08–5.04 (m, 2H, CH=C*H* and C*H*), 4.18–4.05 (m, 2H, NCH_2).

^{13}C NMR ($CDCl_3$, 101 MHz): δ 147.7, 141.7, 134.6, 129.2 (2 × C), 129.2 (2 × C), 128.9, 128.6, 128.2 (2 × C), 127.3 (2 × C), 121.6, 119.5, 116.1, 76.0, 61.0, 57.5.

LC-HRMS (ESI-TOF): m/z $[M+H]^+$ calcd. for $C_{19}H_{16}N_2H^+$: 273.1386; found 273.1385.

SFC: Trefoil CEL2, CO_2/MeOH with gradient from 97% to 90% in 10 min, 1.8 mL/min., 40 °C, detection at 324 nm. Retention time (min.): 7.71 and 8.54.

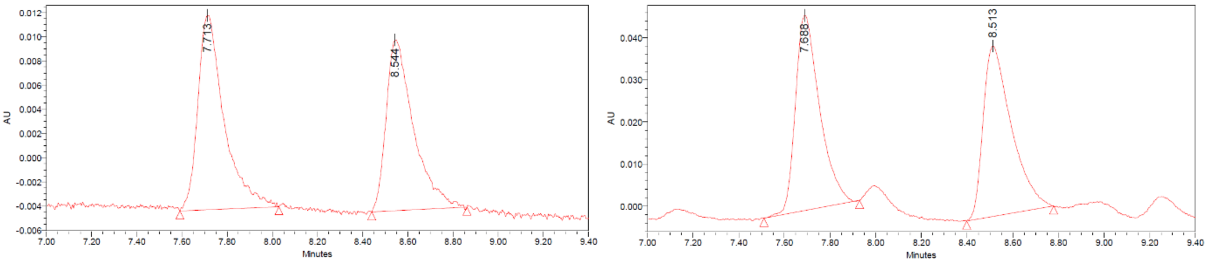

Peak Results

	Name	RT	Area	% Area	Height
1		7.713	130279	52.19	16065
2		8.544	119334	47.81	14116

Peak Results

	Name	RT	Area	% Area	Height
1		7.688	342793	49.64	46364
2		8.513	347812	50.36	40503

SFC of (*rac*)-1-Benzyl-4-phenyl-1,4-dihydropyridine-3-carbonitrile (**4s**)

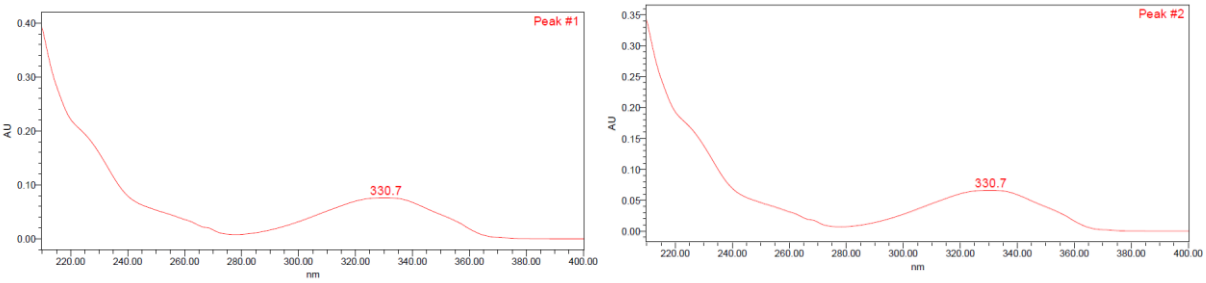

UV-visible spectra of (*rac*)-1-Benzyl-4-phenyl-1,4-dihydropyridine-3-carbonitrile (**4s**)

(*Rac*)-1,4-Dibenzyl-1,4-dihydropyridine-3-carbonitrile (4t)

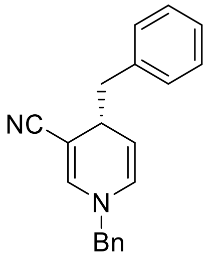

The reaction was performed with **1a** (55.0 mg, 0.2 mmol, 1.0 equiv.), CuTC (3.8 mg, 10.0 mol%), (*R*)-Tol-BINAP (16.3 mg, 12.0 mol%), BnMgCl (1.0 M in Et_2O, 240 μL, 0.24 mmol, 1.2 equiv.) in CH_2Cl_2 (2.0 mL) at −78 °C for 16 h. Product **4t** was obtained as a yellow oil after column chromatography (SiO_2, pentane:EtOAc = 3:1) [>99% conversion, 48.8 mg, 85% yield, <5% *ee*.

^{1}H NMR ($CDCl_3$, 400 MHz): δ 7.34–7.24 (m, 6H, 6 × CH_{Ar}), 7.21–7.19 (m, 2H, 2 × CH_{Ar}), 6.95–6.93 (m, 2H, 2 × CH_{Ar}), 6.54 (d, *J* = 1.7 Hz, 1H, C=C*H*), 5.69 (dt, *J* = 8.2 and 1.3 Hz, 1H, C*H*=CH), 4.59 (dd, *J* = 8.2 and 4.1 Hz, 1H, CH=C*H*), 4.18 (s, 2H, NCH_2), 3.60–3.56 (m, 1H, C*H*), 2.90–2.80 (m, 2H, CH_2).

^{13}C NMR ($CDCl_3$, 101 MHz): δ 143.3, 137.6, 136.3, 130.1 (2 × C), 129.0 (2 × C), 128.3, 128.1 (2 × C), 128.0, 126.9 (2 × C), 126.2, 121.3, 105.4, 82.2, 57.2, 44.0, 33.4.

LC-HRMS (ESI-TOF): m/z $[M+H]^+$ calcd. for $C_{20}H_{18}N_2H^+$: 287.1543; found 287.1180.

SFC: Trefoil CEL2, CO_2/MeOH with gradient from 97% to 90% in 10 min, 1.8 mL/min., 40 °C, detection at 324 nm. Retention time (min.): 6.93 and 7.25.

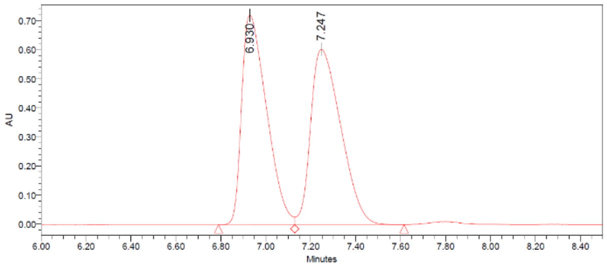

Peak Results

	Name	RT	Area	% Area	Height
1		6.930	5545370	49.62	720600
2		7.247	5630348	50.38	602746

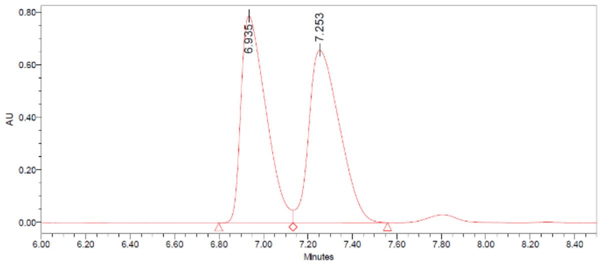

Peak Results

	Name	RT	Area	% Area	Height
1		6.935	6139148	49.37	786954
2		7.253	6294626	50.63	656301

SFC of (*rac*)-1,4-Dibenzyl-1,4-dihydropyridine-3-carbonitrile (**4t**)

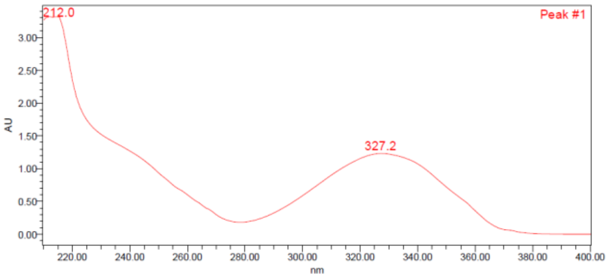

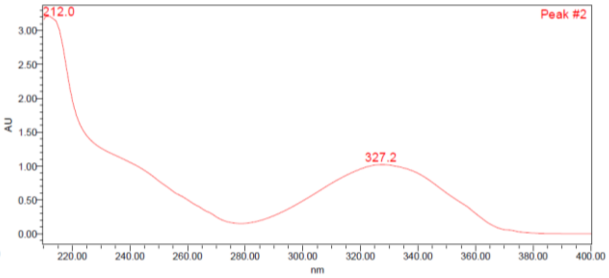

UV-visible spectra of (*rac*)-1,4-Dibenzyl-1,4-dihydropyridine-3-carbonitrile (**4t**)

(*R*)-1-benzyl-4-methyl-1,4-dihydropyridine-3-carbonitrile (4u)

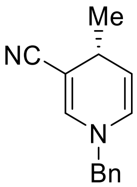

The reaction was performed with **1a** (55.0 mg, 0.2 mmol, 1.0 equiv.), CuTC (3.8 mg, 10.0 mol%), (*R*)-Tol-BINAP (16.3 mg, 12.0 mol%), MeMgBr (3.0 M in Et_2O, 80 μL, 0.24 mmol, 1.2 equiv.) in CH_2Cl_2 (2.0 mL) at −40 °C for 16 h. Product **4u** was obtained as a yellow oil after column chromatography (SiO_2, pentane:EtOAc = 9:1) [>99% conversion, 8.5 mg, 20% yield, 36% *ee*.

^{1}H NMR ($CDCl_3$, 600 MHz): δ 7.39–7.37 (m, 2H, 2 × CH_{Ar}), 7.34–7.31 (m, 1H, CH_{Ar}), 7.20 (d, *J* = 7.5 Hz, 2H, 2 × CH_{Ar}), 6.57 (d, *J* = 1.6 Hz, 1H, C=C*H*), 5.72 (d, *J* = 8.1 Hz, 1H, C*H*=CH), 4.65 (dd, *J* = 8.1 and 3.8 Hz, 1H, CH=C*H*), 4.28 (s, 2H, NCH_2), 3.28–3.24 (m, 1H, C*H*), 1.24 (d, *J* = 6.6 Hz,, 3H, CH_3).

^{13}C NMR ($CDCl_3$, 151 MHz): δ 142.4, 136.6, 129.2 (2 × C), 128.3, 127.3, 127.3 (2 × C), 121.4, 18.2, 84.4, 57.5, 28.3, 25.0.

SFC: Trefoil CEL2, CO_2/MeOH with gradient from 97% to 90% in 10 min, 1.8 mL/min., 40 °C, detection at 324 nm. Retention time (min.): 3.48 (minor) and 3.67 (major).

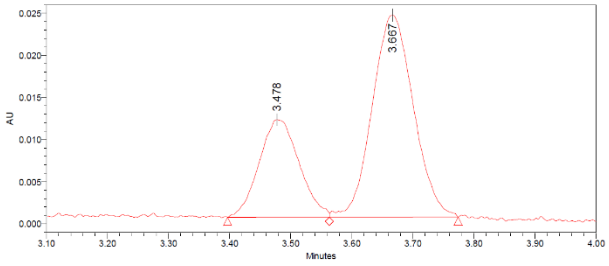

Peak Results

	Name	RT	Area	% Area	Height
1		3.478	52788	31.94	11583
2		3.667	112476	68.06	23971

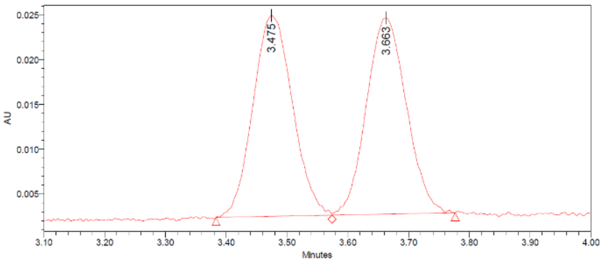

Peak Results

	Name	RT	Area	% Area	Height
1		3.475	101626	50.38	22450
2		3.663	100092	49.62	21919

SFC of (*rac*)-1,4-Dibenzyl-1,4-dihydropyridine-3-carbonitrile (**4t**)

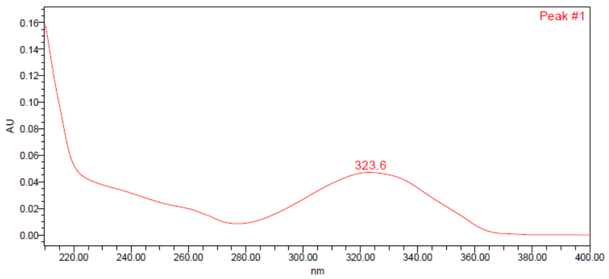

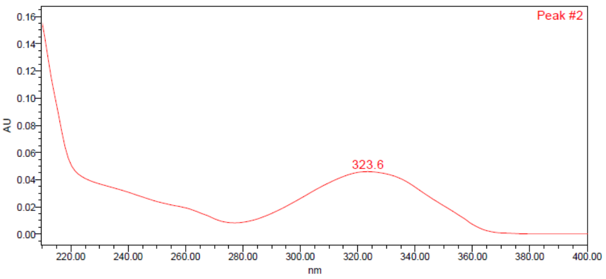

UV-visible spectra of (*rac*)-1,4-Dibenzyl-1,4-dihydropyridine-3-carbonitrile (**4t**)

(*R*)-1-Benzyl-4-ethyl-6-methyl-1,4-dihydropyridine-3-carbonitrile (5a)

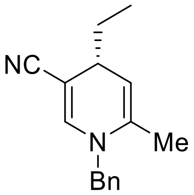

The reaction was performed with **1o** (55.0 mg, 0.2 mmol, 1.0 equiv.), CuTC (3.8 mg, 10.0 mol%), (*R*)-Tol-BINAP (16.3 mg, 12.0 mol%), EtMgBr (3.0 M in Et_2O, 80 μL, 0.24 mmol, 1.2 equiv.) in CH_2Cl_2 (2.0 mL) at −78 °C for 16 h. Product **5a** was obtained as a yellow oil after column chromatography (SiO_2, pentane:EtOAc = 3:1) [>99% conversion, 40.1 mg, 97% yield, 40% *ee*, (*R*)-configuration].

^{1}H NMR ($CDCl_3$, 400 MHz): δ 7.39–7.34 (m, 2H, 2 × CH_{Ar}), 7.31–7.27 (m, 1H, CH_{Ar}), 7.19–7.16 (m, 2H, 2 × CH_{Ar}), 6.65 (s, 1H, C=C*H*), 4.50–4.38 (m, 3H, NCH_2 and C=C*H*), 3.24–3.20 (m, 1H, C*H*), 1.75 (s, 3H, CH_3), 1.63–1.45 (m, 2H, CH_2), 0.96 (t, *J* = 7.4 Hz, 3H, CH_3).

^{13}C NMR ($CDCl_3$, 101 MHz): δ 144.9, 138.1, 134.1, 129.1 (2 × C), 127.8, 126.0 (2 × C), 121.5, 104.2, 83.2, 54.0, 35.3, 30.4, 18.8, 9.5.

LC-HRMS (APCI): m/z $[M+Na]^+$ calcd. for $C_{16}H_{18}N_2Na^+$: 261.1362; found 261.0787.

SFC: Trefoil CEL2, CO_2/MeOH with gradient from 97% to 90% in 10 min, 1.8 mL/min., 40 °C, detection at 324 nm. Retention time (min.): 4.27 (minor) and 4.48 (major).

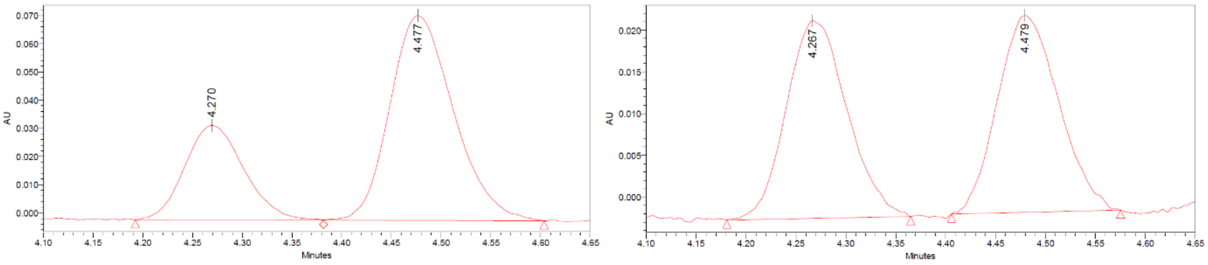

Peak Results

	Name	RT	Area	% Area	Height
1		4.270	138751	30.02	33532
2		4.477	323484	69.98	72678

Peak Results

	Name	RT	Area	% Area	Height
1		4.267	100399	49.94	23704
2		4.479	100647	50.06	23582

SFC of (*R*)-1-benzyl-4-ethyl-6-methyl-1,4-dihydropyridine-3-carbonitrile (**5a**)

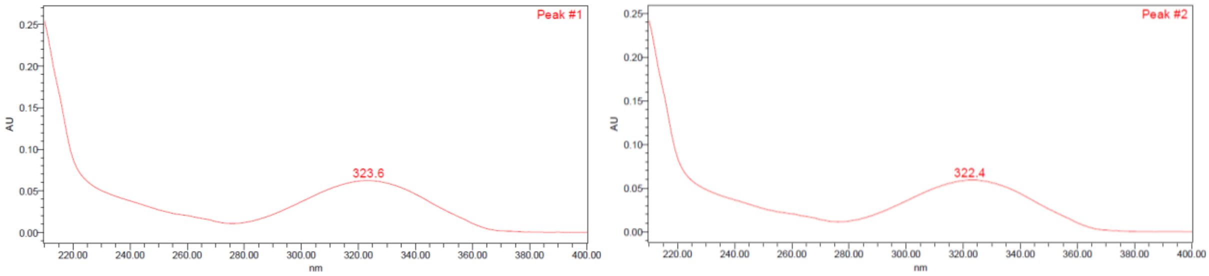

UV-visible spectra of (*R*)-1-benzyl-4-ethyl-6-methyl-1,4-dihydropyridine-3-carbonitrile (**5a**)

(*R*)-1-Benzyl-4-hexyl-6-methyl-1,4-dihydropyridine-3-carbonitrile (5b)

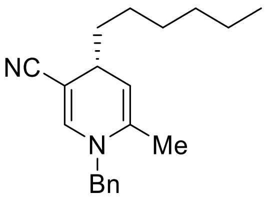

The reaction was performed with **1o** (55.0 mg, 0.2 mmol, 1.0 equiv.), CuTC (3.8 mg, 10.0 mol%), (*R*)-Tol-BINAP (16.3 mg, 12.0 mol%), ^{n}HexMgBr (2.0 M in Et_2O, 100 μL, 0.24 mmol, 1.2 equiv.) in CH_2Cl_2 (2.0 mL) at −78 °C for 16 h. Product **5b** was obtained as a yellow oil after column chromatography (SiO_2, pentane:EtOAc = 3:1) [>99% conversion, 40.1 mg, 98% yield, 61% *ee*, (*R*)-configuration].

^{1}H NMR ($CDCl_3$, 400 MHz): δ 7.38–7.27 (m, 3H, 3 × CH_{Ar}), 7.19–7.17 (m, 2H, 2 × CH_{Ar}), 6.63 (s, 1H, C=C*H*), 4.50–4.37 (m, 3H, NCH_2 and C=C*H*), 3.24–3.20 (m, 1H, C*H*), 1.74 (s, 3H, CH_3), 1.57–1.25 (m, 10H, 5 × CH_2), 0.89 (t, *J* = 7.4 Hz, 3H, CH_3).

^{13}C NMR ($CDCl_3$, 101 MHz): δ 144.7, 138.1, 133.7, 129.1 (2 × C), 127.8, 126.1 (2 × C), 121.6, 104.7, 83.6, 54.0, 38.2, 34.2, 32.1, 29.5, 25.3, 22.8, 18.8, 14.3.

LC-HRMS (ESI-TOF): m/z [M+Na]$^+$ calcd. for $C_{20}H_{20}N_2Na^+$: 317.1988; found 317.2034.

SFC: Trefoil CEL2, CO_2/MeOH with gradient from 97% to 90% in 10 min, 1.8 mL/min., 40 °C, detection at 324 nm. Retention time (min.): 4.82 (minor) and 5.11 (major).

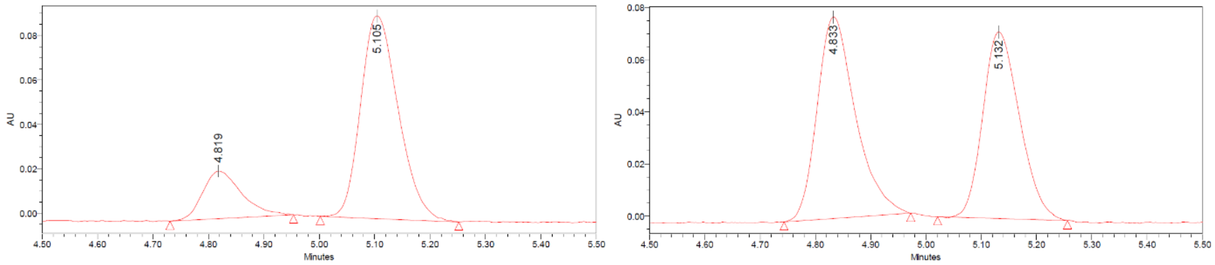

Peak Results

	Name	RT	Area	% Area	Height
1		4.819	104931	19.51	21413
2		5.105	432973	80.49	91273

Peak Results

	Name	RT	Area	% Area	Height
1		4.833	373768	53.01	77357
2		5.132	331379	46.99	71645

SFC of (*R*)-1-benzyl-4-hexyl-6-methyl-1,4-dihydropyridine-3-carbonitrile (**5b**)

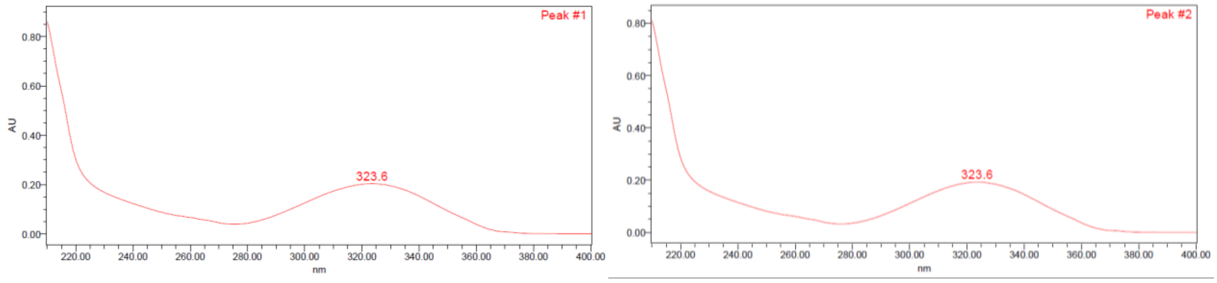

UV-visible spectra of (*R*)-1-benzyl-4-hexyl-6-methyl-1,4-dihydropyridine-3-carbonitrile (**5b**)

Methyl (*R*)-1-benzyl-4-hexyl-1,4-dihydropyridine-3-carboxylate (5c)

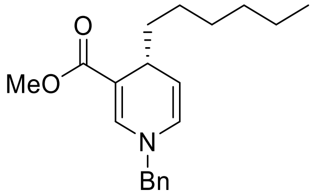

The reaction was performed with **1p** (55.0 mg, 0.2 mmol, 1.0 equiv.), CuTC (3.8 mg, 10.0 mol%), (*R*)-Tol-BINAP (16.3 mg, 12.0 mol%), ^{n}HexMgBr (2.0 M in Et_2O, 100 µL, 0.24 mmol, 1.2 equiv.) in CH_2Cl_2 (2.0 mL) at −78 °C for 16 h. Product **5c** was obtained as a yellow oil after column chromatography (SiO_2, pentane:EtOAc = 3:1) [>99% conversion, 40.9 mg, 65% yield, 24% *ee*, (*R*)-configuration].

^{1}H NMR ($CDCl_3$, 400 MHz): δ 7.37–7.20 (m, 6H, 5 × CH_{Ar} and C=C*H*), 5.82 (dd, *J* = 7.9 and 1.6 Hz, 1H, C*H*=CH), 4.81 (dd, *J* = 7.9 and 5.0 Hz, 1H, CH=C*H*), 4.35 (s, 2H, NCH_2), 3.68 (s, 3H, OCH_3), 3.41–3.37 (m, 1H, C*H*), 1.52–1.43 (m, 1H, C*H*H), 1.37–1.24 (m, 9H, 4 × CH_2 and CH*H*), 0.88 (t, *J* = 6.5 Hz, 3H, CH_3).

^{13}C NMR ($CDCl_3$, 101 MHz): δ 169.0, 141.7, 137.5, 128.9 (2 × C), 128.0, 127.9, 127.1 (2 × C), 108.6, 101.7, 57.7, 50.9, 38.2, 32.2, 31.7, 29.6, 24.9, 22.8, 14.3.

LC-HRMS (ESI-TOF): m/z $[M+H]^+$ calcd. for $C_{20}H_{28}ClNO_2^+$: 314.2115; found 314.2112.

SFC: Trefoil CEL2, CO_2/MeOH with gradient from 97% to 90% in 10 min, 1.8 mL/min., 40 °C, detection at 324 nm. Retention time (min.): 4.16 (minor) and 4.40 (major).

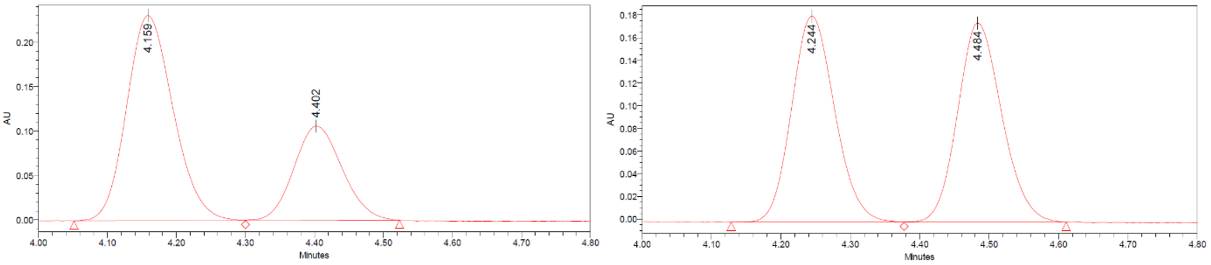

Peak Results

	Name	RT	Area	% Area	Height
1		4.159	1084485	67.79	230982
2		4.402	515308	32.21	106043

Peak Results

	Name	RT	Area	% Area	Height
1		4.244	784787	50.17	181775
2		4.484	779531	49.83	175528

SFC of methyl (*R*)-1-benzyl-4-hexyl-1,4-dihydropyridine-3-carboxylate (**5c**)

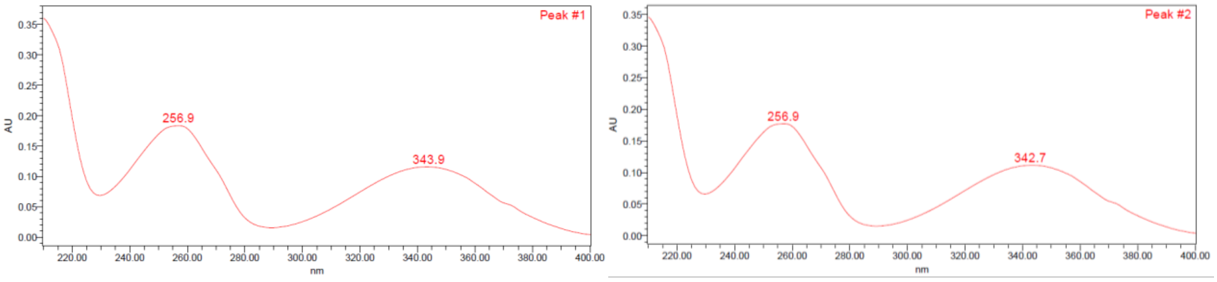

UV-visible spectra of methyl (*R*)-1-benzyl-4-hexyl-1,4-dihydropyridine-3-carboxylate (**5c**)

(*R*)-1-Benzyl-4-hexyl-1,4-dihydroquinoline-3-carbonitrile (5d)

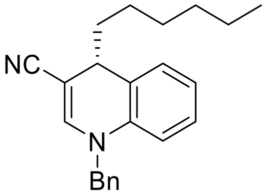

The reaction was performed with **1q** (55.0 mg, 0.2 mmol, 1.0 equiv.), CuTC (3.8 mg, 10.0 mol%), (*R*)-Tol-BINAP (16.3 mg, 12.0 mol%), ^{n}HexMgBr (2.0 M in Et_2O, 100 µL, 0.24 mmol, 1.2 equiv.) in CH_2Cl_2 (2.0 mL) at −78 °C for 16 h. Product **5d** was obtained as a yellow oil after column chromatography (SiO_2, pentane:EtOAc = 3:1) [>99% conversion, 57.1 mg, 86% yield, 11% *ee*, (*R*)-configuration].

^{1}H NMR ($CDCl_3$, 400 MHz): δ 7.37–7.27 (m, 3H, 3 × C*H*$_{Ar}$), 7.24–7.22 (m, 2H, 2 × C*H*$_{Ar}$), 7.09–6.98 (m, 3H, 3 × C*H*$_{Ar}$), 6.94 (s, 1H, C=C*H*), 6.70 (d, *J* = 8.0 Hz, 1H, C*H*$_{Ar}$), 4.89–4.69 (m, 2H, NC*H*$_2$), 3.79 (t, *J* = 5.3 Hz, 1H, C*H*), 1.73–1.65 (m, 2H, C*H*$_2$), 1.48–1.37 (m, 1H, C*H*H), 1.34–1.18 (m, 7H, 3 × C*H*$_2$ and CH*H*), 0.87 (t, *J* = 6.5 Hz, 3H, C*H*$_3$).

^{13}C NMR ($CDCl_3$, 101 MHz): δ 144.4, 137.3, 136.1, 129.2, 129.1 (2 × C), 128.0, 127.5, 126.4 (2 × C), 125.0, 123.8, 121.3, 113.5, 82.6, 54.9, 39.2, 37.8, 31.9, 29.4, 25.0, 22.7, 14.2.

LC-HRMS (ESI-TOF): m/z $[M+H]^+$ calcd. for $C_{23}H_{26}N_2H^+$: 331.2169; found 331.1780.

SFC: Trefoil CEL2, CO_2/MeOH with gradient from 97% to 90% in 10 min, 1.8 mL/min., 40 °C, detection at 324 nm. Retention time (min.): 7.04 (minor) and 7.67 (major).

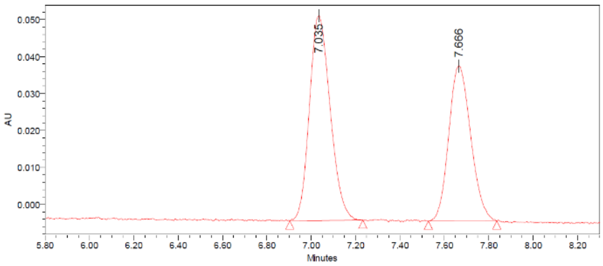

Peak Results

	Name	RT	Area	% Area	Height
1		7.035	360875	55.53	55454
2		7.666	289021	44.47	41976

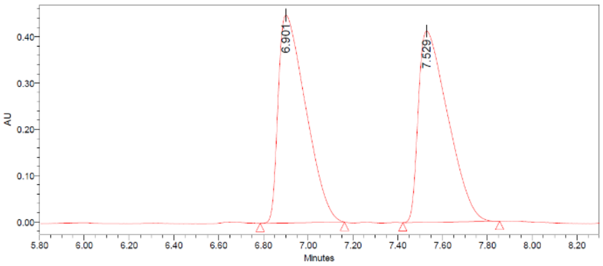

Peak Results

	Name	RT	Area	% Area	Height
1		6.901	3834195	50.28	449205
2		7.529	3790895	49.72	413447

SFC of (*R*)-1-benzyl-4-hexyl-1,4-dihydroquinoline-3-carbonitrile (**5d**)

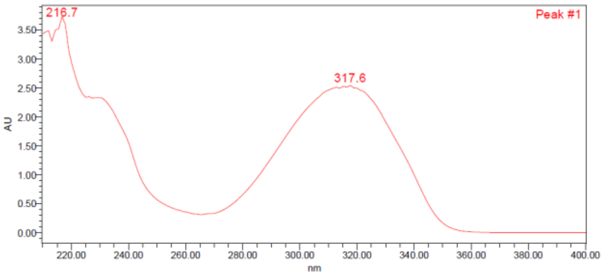

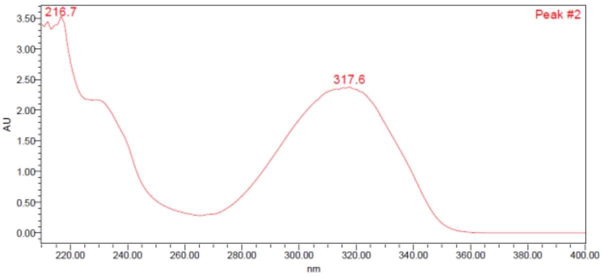

UV-visible spectra of (*R*)-1-benzyl-4-hexyl-1,4-dihydroquinoline-3-carbonitrile (**5d**)

5.4 Synthetic transformations

(4*S*,5*R*)-*N*-Benzyl-5-fluoro-4-hexyl-1,4,5,6-tetrahydropyridine-3-carbonitrile (6)

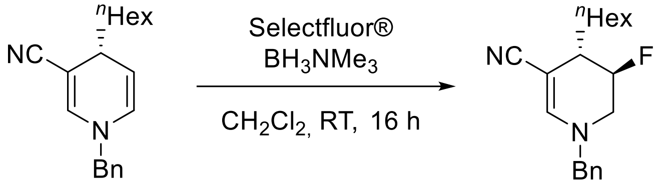

In a 4 mL screw-cap vial, equipped with magnetic stir bar, the 1,4-dihydropyridine **4c** (1.0 equiv) was dissolved in MeCN (1.0 mL). Selectfluor® (1.0 equiv) and NMe_3BH_3 (1.0 equiv) were added and the mixture was stirred for 16 h at room temperature. The mixture was purified by flash column chromatography (SiO_2, pentane:EtOAc = 3:1) to obtain product **6** as a yellow oil in 72.5 mg (80% yield) with 90% *ee*.

^{1}H NMR ($CDCl_3$, 600 MHz): δ 7.39–7.33 (m, 3H, 3 × C*H*$_{Ar}$), 7.20 (d, *J* = 7.4 Hz, 2H, 2 × C*H*$_{Ar}$), 6.97 (s, 1H, C=C*H*), 4.70 (dq, *J* = 46.4 and 2.9 Hz, 1H, C*H*F), 4.36–4.26 (m, 2H, NC*H*$_2$), 3.25–3.20 (m, 1H, C*H*H), 3.08 (dd, *J* = 38.7 and 14.0 Hz, 1H, CH*H*), 2.51–2.46 (m, 1H, C*H*), 1.51–1.23 (m, 10H, 5 × C*H*$_2$), 0.88 (t, *J* = 6.7 Hz, 3H, C*H*$_3$).

^{13}C NMR ($CDCl_3$, 151 MHz): δ 146.2, 135.8, 129.2 (2 × C), 128.4, 127.6 (2 × C), 123.0, 86.6, 85.4, 75.9, 59.6, 46.0 (d, *J* = 21.7 Hz), 37.5 (d, *J* = 19.8 Hz), 35.5 (d, *J* = 7.6 Hz), 31.8, 29.4, 26.4, 22.7, 14.2.

^{19}F NMR ($CDCl_3$, 565 MHz): δ −177.05 (ddt, *J* = 46.5, 38.3 and 13.9 Hz).

LC-HRMS (ESI-TOF): m/z $[M+Na]^+$ calcd. for $C_{19}H_{25}FN_2Na^+$: 323.1894; found 323.1892.

SFC: Trefoil CEL2, CO_2/MeOH with gradient from 97% to 90% in 10 min, 1.8 mL/min., 40 °C, detection at 269 nm. Retention time (min.): 5.55 (minor) and 6.17 (major).

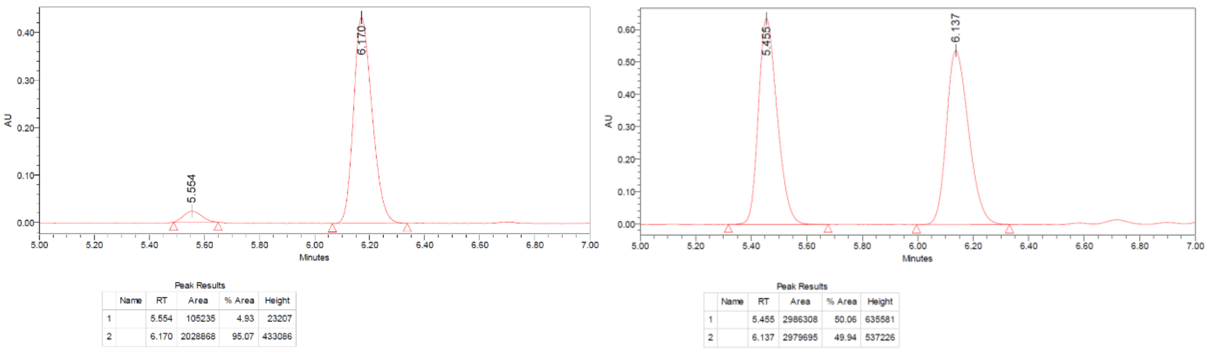

	Name	RT	Area	% Area	Height
1		5.554	105235	4.93	23207
2		6.170	2028868	95.07	433086

	Name	RT	Area	% Area	Height
1		5.455	2986308	50.06	635581
2		6.137	2979695	49.94	537226

SFC of (4*S*,5*R*)-*N*-benzyl-5-fluoro-4-hexyl-1,4,5,6-tetrahydropyridine-3-carbonitrile (**6**)

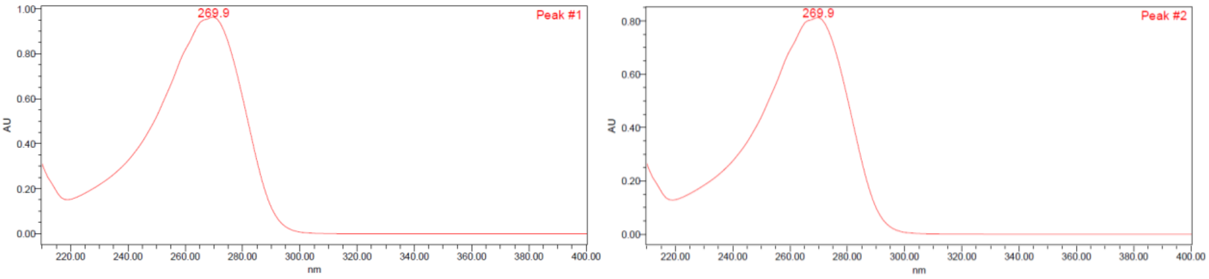

UV-visible spectra of (4*S*,5*R*)-*N*-benzyl-5-fluoro-4-hexyl-1,4,5,6-tetrahydropyridine-3-carbonitrile (**6**)

(*R*)-*N*-Benzyl-4-hexyl-1,4,5,6-tetrahydropyridine-3-carbonitrile (7)

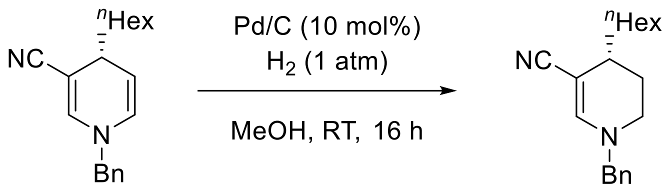

Palladium on carbon (Pd/C 10 wt%, 31.9 mg, 0.03 mmol) was added to a vial containing 1,4-dihydropyridine **4c** (84.1 mg, 0.3 mmol) and MeOH (6 mL) at room temperature under argon. The vial was then placed under vacuum and back-filled with argon two times. After removal of the argon atmosphere a third time with vacuum, a hydrogen balloon was placed in the vial and the reaction stirred was stirred for 16 h. The mixture was then filtered through Celite and the filtrate was evaporated. The mixture was column chromatography (SiO_2, pentane:EtOAc = 3:1) to obtain product **7** as a colourless oil in 80.0 mg (94% yield) with 91% *ee*, *(R)*-configuration.

^{1}H NMR ($CDCl_3$, 400 MHz): δ 7.37–7.35 (m, 2H, 2 × C*H*$_{Ar}$), 7.32–7.29 (m, 1H, C*H*$_{Ar}$), 7.19–7.17 (dd, *J* = 7.2 and 1.8 Hz, 2H, 2 × C*H*$_{Ar}$), 6.89 (d, *J* = 1.0 Hz, 1H, C=C*H*), 4.23 (s, 2H, NC*H*$_2$), 3.00–2.98 (m, 2H, C*H*$_2$), 2.29–2.24 (m, 1H, C*H*), 1.84–1.79 (m, 1H, C*H*H), 1.69–1.64 (m, 1H, CH*H*), 1.59–1.54 (m, 1H, C*H*H), 1.42–1.36 (m, 1H, CH*H*), 1.35–1.23 (m, 8H, 4 × C*H*$_2$), 0.87 (t, *J* = 6.7 Hz, 3H, C*H*$_3$).

^{13}C NMR ($CDCl_3$, 101 MHz): δ 147.2, 136.5, 129.0 (2 × C), 128.1, 127.5 (2 × C), 123.4, 79.1, 59.5, 43.2, 35.6, 31.9, 31.4, 29.5, 26.7, 26.2, 22.7, 14.2.

LC-HRMS (ESI-TOF): m/z $[M+H]^+$ calcd. for $C_{19}H_{26}N_2H^+$: 283.2169; found 283.2166.

SFC: Trefoil CEL2, CO_2/MeOH with gradient from 97% to 90% in 10 min, 1.8 mL/min., 40 °C, detection at 275 nm. Retention time (min.): 6.70 (minor) and 7.24 (major).

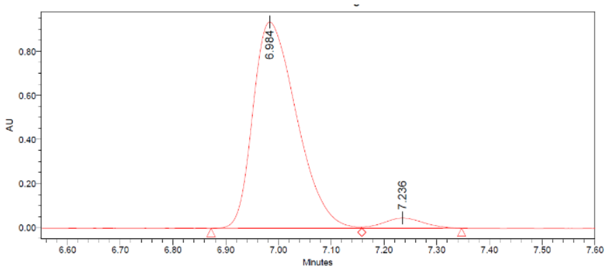

Peak Results

	Name	RT	Area	% Area	Height
1		6.984	5376429	95.74	937204
2		7.236	239387	4.26	46187

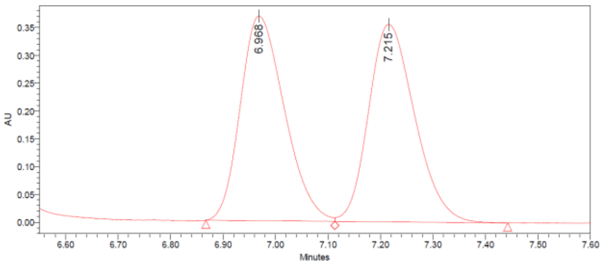

Peak Results

	Name	RT	Area	% Area	Height
1		6.968	2147498	49.74	367891
2		7.215	2169949	50.26	354965

SFC of (*R*)-*N*-benzyl-4-hexyl-1,4,5,6-tetrahydropyridine-3-carbonitrile (**7**)

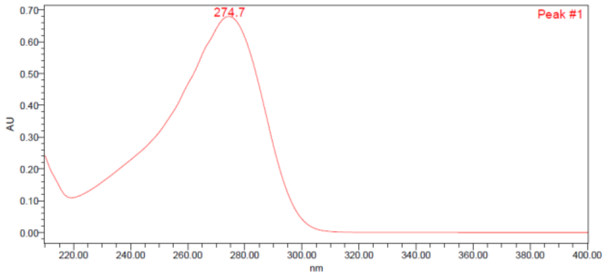

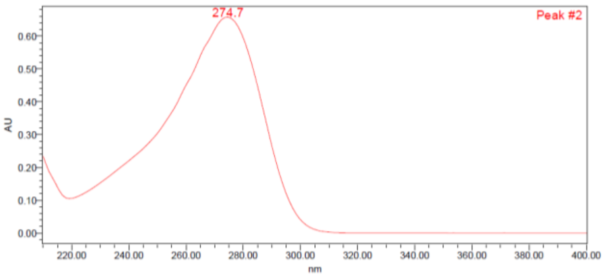

UV-visible spectra of (*R*)-*N*-benzyl-4-hexyl-1,4,5,6-tetrahydropyridine-3-carbonitrile (**7**)

Dimethyl (1*S*,5*R*,6*S*)-2-benzyl-4-cyano-5-hexyl-2-azabicyclo[4.2.0]octa-3,7-diene-7,8-dicarboxylate (8)

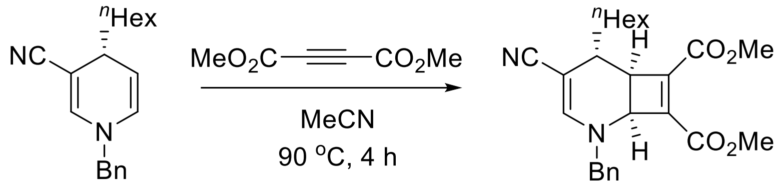

A mixture of dimethyl acetylenedicarboxylate (74 µL, 0.6 mmol, 2.0 equiv.), 1,4-dihydropyridine **4c** (84.1 mg, 0.3 mmol, 1.0 equiv.) in MeCN (1.5 mL) was heated a reflux for 4 hours. After removing the solvent by rotatory evaporation at reduced pressure, the mixture was purified by flash column chromatography (SiO_2, pentane:EtOAc = 3:1) to obtain product **8** as a yellow oil in 98.4 mg (78% yield) with 93% *ee*.

^{1}H NMR ($CDCl_3$, 400 MHz): δ 7.39–7.31 (m, 3H, 3 × CH_{Ar}), 7.27–7.25 (m, 2H, 2 × CH_{Ar}), 6.77 (s, 1H, C=C*H*), 4.61 (d, *J* = 15.0 Hz, 1H, NC*H*H), 4.26 (d, *J* = 15.0 Hz, 1H, NCH*H*), 4.18 (dd, *J* = 4.9 and 1.0 Hz, 1H, C*H*), 3.83 (s, 6H, 2 × OCH_3), 3.35 (dd, *J* = 4.9 and 1.4 Hz, 1H, C*H*), 2.69 (td, *J* = 6.2 and 1.4 Hz, 1H, C*H*), 1.63–1.54 (m, 1H, C*H*H), 1.51–1.25 (m, 9H, 4 × CH_2 and CH*H*), 0.89 (t, *J* = 6.4 Hz, 3H, CH_3).

^{13}C NMR ($CDCl_3$, 101 MHz): δ 161.5, 161.0, 146.0, 145.5, 136.4, 136.2, 128.9, (2 × C), 128.1, 128.0 (2 × C), 122.6, 81.8, 58.2, 53.9, 52.4, 52.3, 48.1, 34.5, 33.4, 31.8, 29.3, 26.6, 22.6, 14.1.

LC-HRMS (ESI-TOF): m/z $[M+H]^+$ calcd. for $C_{25}H_{30}N_2O_4H^+$: 423.2278; found 423.2275.

SFC: Trefoil CEL2, CO_2/MeOH with gradient from 97% to 90% in 10 min, 1.8 mL/min., 40 °C, detection at 324 nm. Retention time (min.): 6.00 (minor) and 6.41 (major).

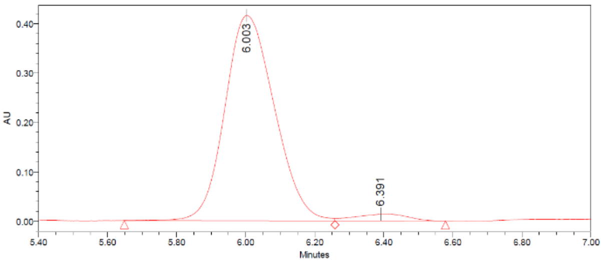

Peak Results

	Name	RT	Area	% Area	Height
1		6.003	4142382	96.68	415413
2		6.391	142050	3.32	13577

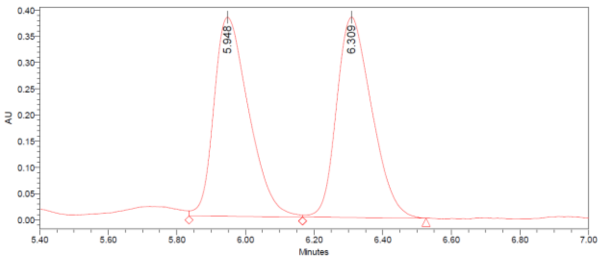

Peak Results

	Name	RT	Area	% Area	Height
1		5.948	2677961	49.62	379693
2		6.309	2718747	50.38	382077

SFC of dimethyl (1*S*,5*R*,6*S*)-2-benzyl-4-cyano-5-hexyl-2-azabicyclo[4.2.0]octa-3,7-diene-7,8-dicarboxylate (**8**)

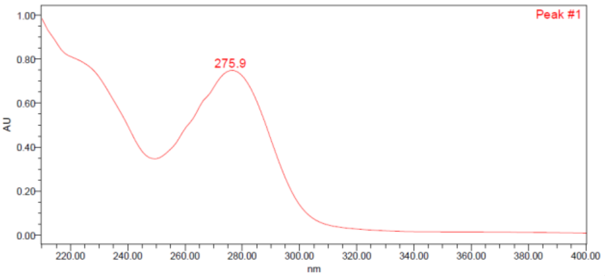

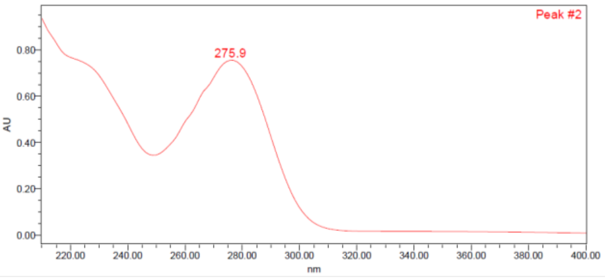

UV-visible spectra of dimethyl (1*S*,5*R*,6*S*)-2-benzyl-4-cyano-5-hexyl-2-azabicyclo[4.2.0]octa-3,7-diene-7,8-dicarboxylate (**8**)

***N*-Benzyl-5-bromo-4-hexyl-6-oxo-1,6-dihydropyridine-3-carbonitrile (9)**

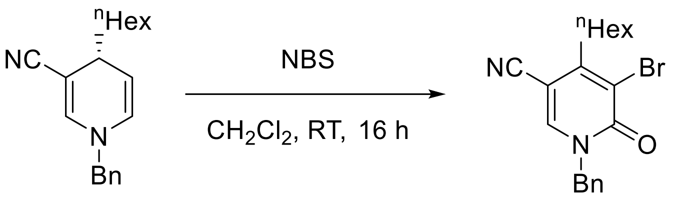

To solution of 1,4-dihydropyridine **4c** (84.1 mg g, 0.3 mmol, 1.0 equiv.) in DCM (3.0 mL) was added *N*-bromosuccinimide (NBS; 106.8 mg, 0.6 mmol, 2.0 equiv.). The reaction mixture was vigorously stirred for 12 h. The mixture was concentrated and purified by flash column chromatography (SiO_2, CH_2Cl_2) to obtain product **9** as a yellow oil in 63.0 mg (71% yield).

^{1}H NMR ($CDCl_3$, 400 MHz): δ 7.69 (s,1H, C=C*H*), 7.41–7.32 (m, 5H, 5 × CH_{Ar}), 5.16 (s, 2H, NCH_2), 2.84–2.80 (m, 2H, CH_2), 1.64–1.57 (m, 2H, CH_2), 1.47–1.42 (m, 2H, CH_2), 1.35–1.30 (m, 4H, 2 × CH_2), 0.90 (t, *J* = 6.7 Hz, 3H, CH_3).

^{13}C NMR ($CDCl_3$, 101 MHz): δ 158.1, 152.8, 142.3, 134.2, 129.5 (2 × C), 129.3, 129.1 (2 × C), 117.8, 115.3, 93.9, 51.1, 36.0, 31.5, 29.3, 28.0, 22.6, 14.2.

LC-HRMS (ESI-TOF): m/z $[M+H]^+$ calcd. for $C_{19}H_{21}BrN_2OH^+$: 373.0910; found 373.0908.

6. Mechanistic studies

6.1. DFT calculations

To get a deeper understanding on the nuances behind the presented enantioselective transformation, we performed some mechanistic studies using in-lab experiments, such as the non-linear relationship study, but also, molecular modelling and Quantitative Kinetic Isotopic Effects (QKIE). Starting with the DFT and the non-linear relationship study, we have shown previously that CuBr in the presence of (*R*)-Tol-BINAP **L11** renders the copper complex **L11**CuBr and that when EtMgBr·2Et_2O is added to the reaction mixture a transmetalation takes place yielding **L11**CuEt and $MgBr_2$·2Et_2O.[17] Hence, we started our studies by exploring the interaction of **L11**CuEt and **L11**CuBr with **1a**.

Initially, we explored whether dimeric copper species could be involved in the reaction. Using a non-linear relationship study we obtained that dimeric species are likely not involved in this reaction (**section 6.1.1**). Hence, we have only considered the interaction between the substrate and the monomeric forms of the copper complexes. Regarding the interaction of **L11CuBr** and **1a**, in-silico mechanistic studies reveal that the **L11CuBr** species does not coordinate **1a** (all our attempts to find this complex resulted in the decoordination of the copper centre and the substrate). We have assigned this result to the electron deficiency of the substrate and that of the copper complex (please note that while at the **L11CuEt** complex there is only one phosphorous coordinating the metal centre, when **1a** is complexated both phosphorous groups at the ligand coordinate the metal centre, revealing a greater electron deficiency at this metal core in the latter scenario).

Interestingly, when we moved to explore the interaction of the substrate (**1a**) with **L11**CuEt we have found that it only coordinates to positions C4-C5 and C5-C6, being the resulting C4-C5 complex more stable by approximately 3 kcal/mol that the C5-C6 (**Scheme S1a**).

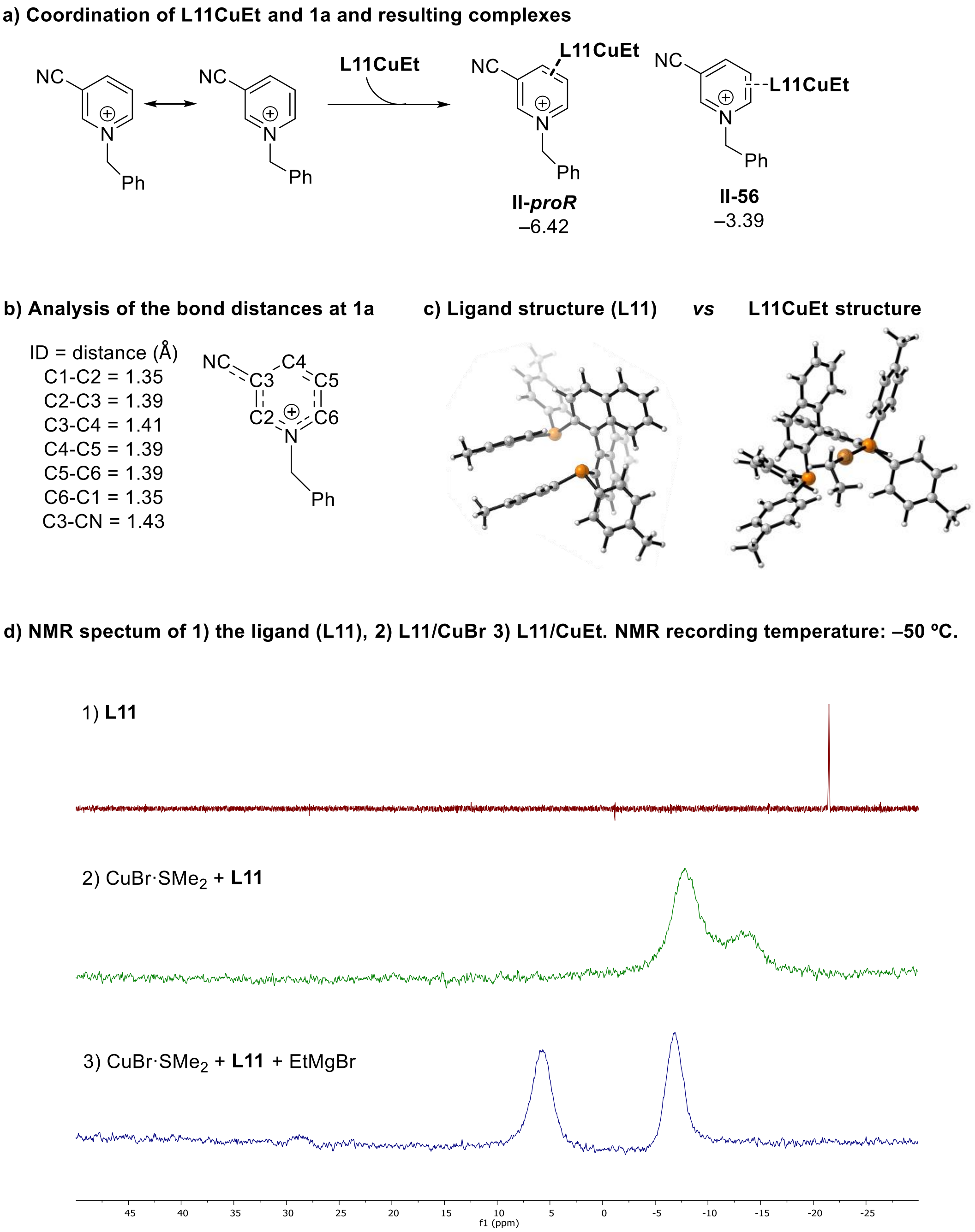

Scheme S1. a) Study of the coordination of **L11**CuEt to **1a**. b) Analysis of the bond distances at **1a**. c) Cartoon of the ligand structure and the **L11**CuEt complex. d) NMR studies on the coordination of **L11** to CuBr and CuEt.

Intrigued by the lack of coordination with positions N1-C2, C2-C3, C3-C4, C6-N1, we analysed charges and bond distances at the substrate. We noticed that the C2-C3 and C3-C4 bond distances are slightly elongated w.r.t. the other C=C bonds revealing the participation of resonance forms where these bonds have mostly single character. It is quite notorious the elongation of the C3-C4 bond, which has a bond distance that is closer to a single bond than to a double.[18] Additionally, the C2-N and C6-N bond distances are also slightly larger than those corresponding to a C=N bond (**Scheme S1b**).[19] These bond distances reveal that the bonds of **1a** that are the most susceptible of coordination are, in principle, N1-C2, C2-C3, C4-C5,C5-C6, C6-N1. Among them, we have found that those bonds bearing substituents are not capable of interacting with copper, being steric hindrance the main reason, *i.e.* the presence of the CH_2 group and the CN moiety, impede the accommodation of (*R*)-Tol-BINAP **L11** which features a quite rigid and lengthy organic scaffold, hence preventing the formation of any potential

copper-substrate complex (**Scheme S1c**). Notice that against what could happen with a (*R*,*R*)-Ph-BPE ligand, here the naphthalene core imposes a significant ligand rigidity which difficult coordination (**Scheme S1c**). We could confirm experimentally, the predicted coordination of the ligand to CuBr and CuEt (**Scheme S1d**).

Having identified the most stable **L11CuEt-1a** complexes, we moved to study the step involving the addition of the *Et* group at the C4 position. We have found that the pair of resulting isomers are very close in energy.

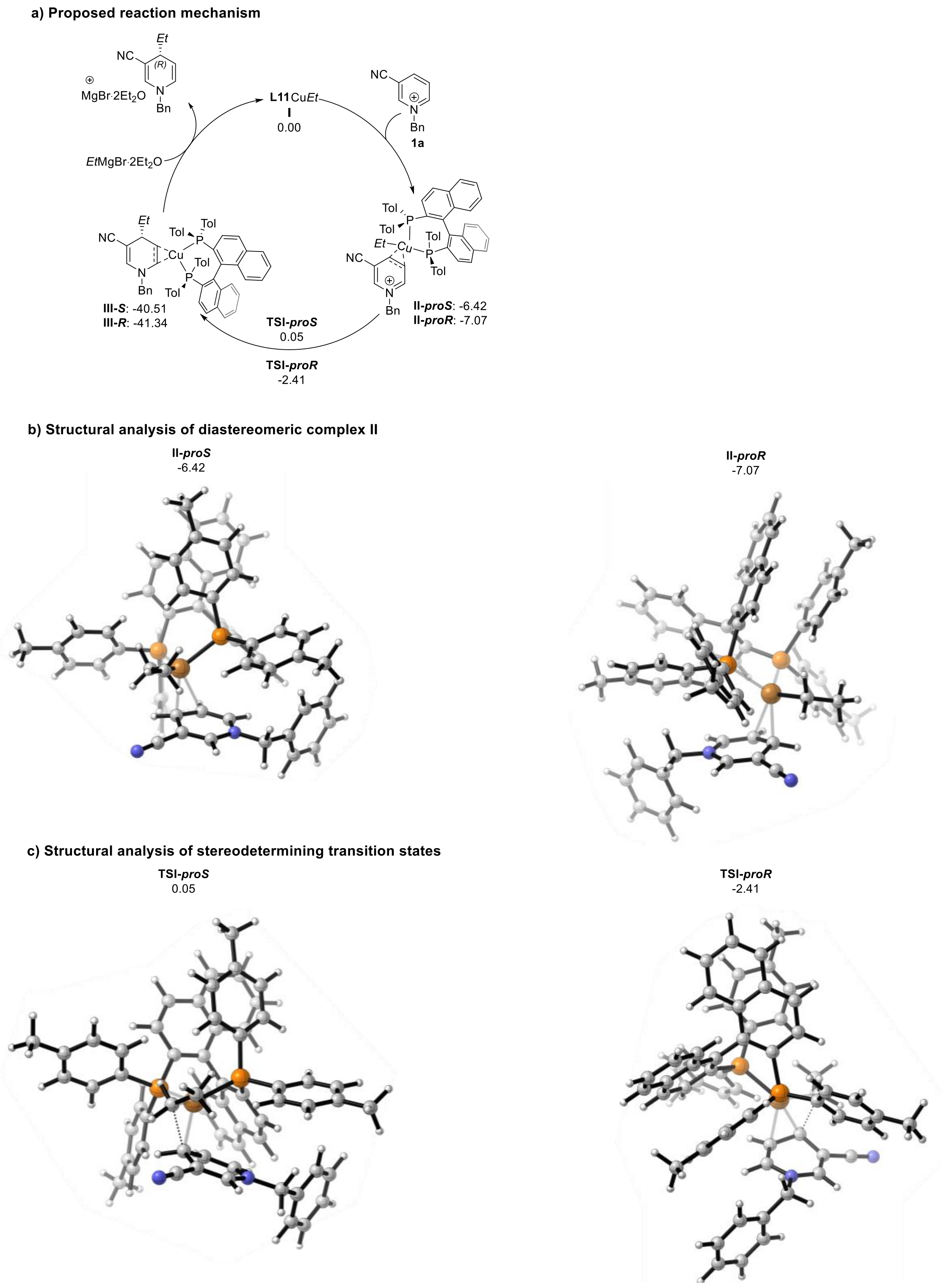

Scheme S2. a) Proposed catalytic cycle. 3D images of b) the diastereomeric **II** complexes and c) the diastereomeric **TSI** transition states.

Table S4. Summary of the energies of the structures presented in Scheme S1 and S2. [a]

ID	ImFreqs	Stable	SCF[b]	SCF+ZPVE[b]	H[c]	G[d]
**II-56**	-	Yes	-4868.272609	-4863.91666	-4863.852193	-4864.023277
**L11CuEt**	-	Yes	-4256.67985	-4253.221193	-4253.16967	-4253.312827
**1a**	-	Yes	-611.5691283	-610.664961	-610.652083	-610.705053
**II-*proS***	-	Yes	-4868.280978	-4863.925344	-4863.861355	-4864.028116
**II-*proR***	-	Yes	-4868.281326	-4863.92601	-4863.862117	-4864.029148
**TSI-*proS***	-260.2	Yes	-4868.269146	-4863.91488	-4863.851382	-4864.0178
**TSI-*proR***	-321.9	Yes	-4868.274503	-4863.920788	-4863.85831	-4864.021715
**EtMgBr-2Et_2O**	-	Yes	-3320.176507	-3319.835029	-3319.812718	-3319.886526
**III-*S***	-	Yes	-4868.33623	-4863.981022	-4863.917587	-4864.082437
**III-*R***	-	Yes	-4868.340453	-4863.984721	-4863.922269	-4864.083762

[a] Energies are expressed in a.u. and the imaginary frequencies in cm^{-1}. [b] SCF denotes electronic energies, [c] H denotes enthalpies and [d] G Gibbs free energies.

6.2 Nonlinear relationship study

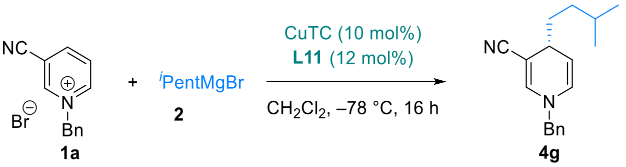

Entry	*ee* of L11 (%)	Yield of 4g (%) [b]	*ee* of 4g (%) [c]
1	100	94	94
2	80	93	68
3	60	96	49
4	40	96	32
5	20	91	16
6	0	92	0

[a] **Reaction conditions:** 3-cyanopyridinium salt (0.2 mmol, 1.0 equiv.), Cu salt (10 mol%), Tol-BINAP ligand **L11** (12 mol%), ^{i}PentMgBr in Et_2O (2 M; 0.24 mmol, 1.2 equiv.) in CH_2Cl_2 (2.0 mL) for 16 h. [b] The yields of **4g** were determined by analysis of ^{1}H NMR spectra of the reaction crude using 1,3,5-trimethoxybenzene as an internal standard. [c] Enantiomeric excess (*ee*) was determined by SFC on a chiral stationary phase.

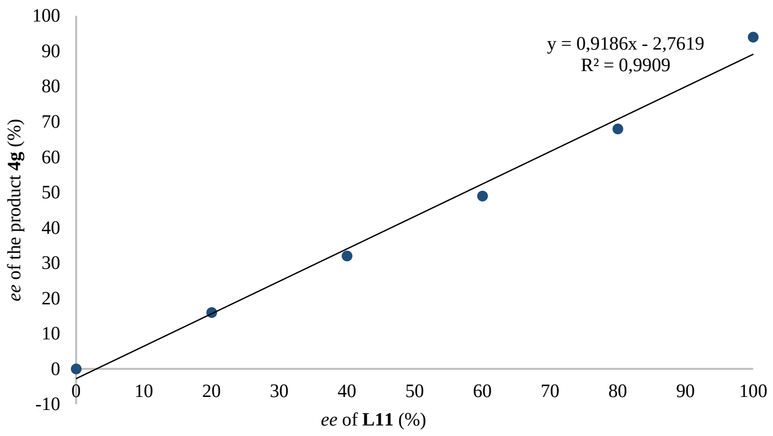

Figure S1. Non-linear effect study on the asymmetric Cu-catalysed dearomatization of pyridinium salt **1a** with ^{i}PentMgBr using Tol-BINAP (**L11**) with different *ee* values

A linear correlation between the *ee* of Tol-BINAP and the product **4g** was observed in this nucleophilic addition dearomatization of pyridinium salt, suggesting the involvement of a 1 : 1 ratio of the chiral ligand and the copper in the copper complexes in the enantiodetermining transition state and the bidentate coordination mode of P,P-ligand to a monomeric copper species in this process.

6.3 Racemization study

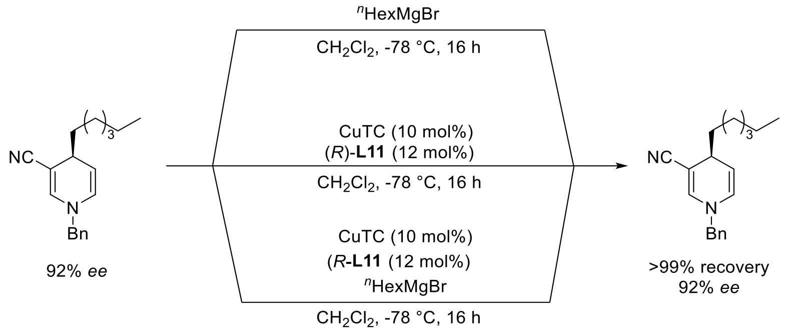

To probe that the reaction product does not racemize or experience any loss of enantioselectivity during the reaction, the product **4c** was subjected to standard conditions with and without ^{n}HexMgBr as well as only Grignard reagent. Racemization of the product was not observed. In addition, purification of product via silica column chromatography does not affect chiral centre of the product.

6.4 Corroboration of the mechanistic proposal via quantitative KIE studies

With a general mechanistic picture of this dearomative protocol, we took a step further and attempted to confirm our proposal by studying the KIE. The accurate determination of KIEs can provide powerful information on the bonding changes occurring at those atoms actively participating in the rate-determining step of a reaction. As a result, it has constituted a key tool in physical-organic chemistry for the past several decades. In 1995, Singleton introduced quantitative ^{13}C NMR methodology for the determination of ^{13}C KIEs at natural abundance with high precision, in a strategy that avoids the necessity of enriching substrates with a specific isotope. In this regard, due to the intrinsic difficulties of labelling the pyridine scaffold or even the Grignard reagents we decided to take advantage of the natural abundance of the ^{13}C isotope in our substrates and study the quantitative kinetic isotope effects (QKIE).

The ^{13}C NMR KIEs study of Cu(I)-catalysed dearomatization reaction between 3-cyanopyridinium salt and isopropylmagnesium bromide (product analysis)

Low conversion reaction

The reaction of **1a** with isobutylmagnesium bromide (^{i}BuMgBr) catalysed by Cu(I)/**L1** catalyst was chosen as a model reaction for the product analysis of experimental ^{13}C KIEs at natural abundance. The study of ^{13}C NMR KIEs at natural abundance, the reactions have to be carried to low conversion for product analysis. Due to kinetic resolution, the product is enriched in the faster reacting isotope (^{12}C) at low conversion, whereas the remaining starting material becomes enriched in the slower reacting isotope (^{13}C) at high conversions. Three identical reactions were taken to 12, 15, and 7% yield of the product **4h** (each of these three KIE measurements require 8 mmol of **1a** and ^{i}BuMgBr), and the dihydropyridine product **4h** was isolated. The desired product **4h** was analyzed by quantitative ^{13}C NMR composition with a standard sample from the same commercial lot of starting materials. The relative changes in ^{13}C composition from **1a** and Grignard reagent were calculated using C-2 and C-3' as internal standards, respectively with the assumption that its isotopic composition does not change.

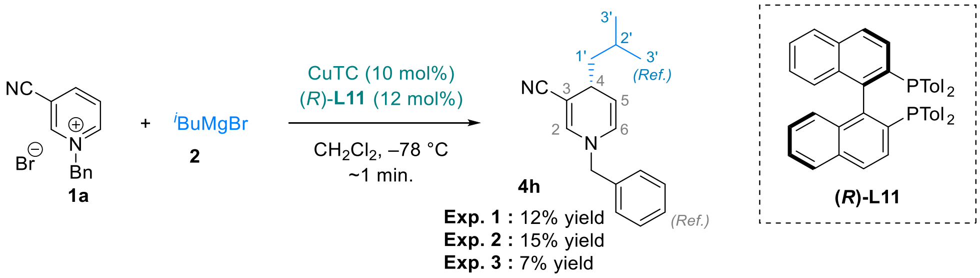

Quantitative ^{13}C NMR of product

1a and ^{i}BuMgBr were chosen as an initial starting material to study ^{13}C NMR KIEs. The quantitative ^{13}C NMR spectra were taken at 151 MHz on a Bruker Avance 600 MHz NMR spectrometer with inverse-gated decoupling. The chosen acquisition parameters were: acquisition time 6.0 s; size of fid 300k; recovery delays 60 s; transmitter frequency offset 100 ppm; number of scans 256. ^{13}C NMR measurements were carried out for the KIE values of product **4h**. ^{13}C NMR data were processed using 1 Hz exponential multiplication. For the KIE determination, the integration of methyl group (from Grignard reagent; ^{i}BuMgBr) and CH of benzene ring (from *N*-benzyl-3-

cyanopyridinium bromide **1a**) were set to 100 which assumed as an internal standard. The average integration values for the other carbons were used to calculate the KIE values following:

$$KIE_{calc} = \frac{ln(1\text{-}F)}{ln[1\text{-}\left(F\frac{R_P}{R_0}\right)]}$$

where F is the fraction of reaction and R_P and R_0 are the isotope ratio of product and initial starting substrate, respectively.

KIEs calculation from 1a (dihydropyridine moiety from the product 4h)

Table S5.1: ^{13}C Integrations of initial **1a** (standard)

C	ppm	#1	#2	#3	#4	#5	average	SD
**2**	149.8	99.34	100.98	99.41	99.77	99.57	99.814	0.672480
**3**	114.2	99.15	99.43	99.01	99.55	99.57	99.342	0.250040
**4**	150.2	104.58	104.32	103.66	104.06	103.38	104.000	0.485386
**5**	130.3	96.79	96.97	96.66	97.06	97.05	96.906	0.175014
**6**	149.2	99.73	99.88	99.77	100.09	99.85	99.864	0.139929
**CN**	115.7	97.49	97.45	97.12	97.22	97.62	97.380	0.204817
**CH (Bn)**	133.5	100	100	100	100	100	100.000	0
**C (Bn)**	131.3	99.69	100.15	99.76	100.02	99.9	99.904	0.187430

Table S5.2: ^{13}C Integrations of dihydropyridine ring from **1a** (12% yield of **4h**)

C	ppm	#1	#2	#3	#4	#5	average	SD
**2**	**142.8**	99.79	99.96	99.77	99.66	99.72	99.7800	0.112472219
**3**	**83.7**	99.20	99.25	99.28	99.12	99.19	99.2080	0.061400326
**4**	**31.0**	101.73	101.64	101.64	101.51	101.69	101.6420	0.082885463
**5**	**106.5**	96.45	96.62	96.52	96.31	96.51	96.4820	0.113885908
**6**	**127.7**	99.72	99.67	99.85	99.76	99.71	99.7420	0.068337398
**CN**	**121.7**	97.46	97.35	97.37	97.42	97.46	97.4120	0.050695167
**C**	**136.6**	99.96	99.87	99.99	100.07	99.86	99.9500	0.087464278
**CH**	**128.1**	100	100	100	100	100	100.0000	0

Table S5.3: Calculated ^{13}C KIEs of dihydropyridine ring from **1a** (12% yield of **4h**)

C	ppm	#1	#2	#3	#4	#5	average	SD
**2**	**142.8**	1.000256554	0.998441948	1.000470443	1.001648364	1.00100554	1.00036457	0.001201907
**3**	**83.7**	1.001526969	1.000988806	1.000666168	1.002389157	1.001634666	1.001441153	0.0006612
**4**	**31.0**	1.023802216	1.024767797	1.024767797	1.026165544	1.024231153	1.024746902	0.000890484
**5**	**106.5**	1.00504329	1.003157559	1.004266009	1.006601237	1.004376981	1.004689015	0.001265283
**6**	**127.7**	1.0015404	1.002076301	1.000149566	1.001112066	1.001647538	1.001305174	0.00073142
**CN**	**121.7**	0.999124374	1.00032873	1.000109554	0.999562008	0.999124374	0.999649808	0.000555016
**C**	**136.6**	0.999402392	1.00036316	0.99908252	0.998230463	1.000470019	0.999509711	0.000932879
**CH**	**128.1**	1	1	1	1	1	1	0

Table S5.4: ^{13}C Integrations of dihydropyridine ring from **1a** (15% yield of **4h**)

C	ppm	#1	#2	#3	#4	#5	average	SD
**2**	**142.8**	99.92	99.79	99.82	99.81	99.86	99.8400	0.051478151
**3**	**83.7**	99.04	99.03	99.16	99.28	99.22	99.1460	0.109909053
**4**	**31.0**	101.93	101.73	101.64	101.90	101.63	101.7660	0.141880231
**5**	**106.5**	96.40	96.47	96.54	96.50	96.52	96.4860	0.054589376
**6**	**127.7**	100.01	99.72	99.86	99.88	99.72	99.8380	0.122147452
**CN**	**121.7**	97.36	97.46	97.33	97.51	97.39	97.4100	0.073824115
**C**	**136.6**	99.82	99.96	99.93	99.77	99.95	99.8860	0.085615419
**CH**	**128.1**	100	100	100	100	100	100.0000	0

Table S5.5: Calculated ^{13}C KIEs of dihydropyridine ring from **1a** (15% yield of **4h**)

C	ppm	#1	#2	#3	#4	#5	average	SD
**2**	**142.8**	0.998848078	1.000261151	0.999934732	1.000043517	0.99949981	0.999717458	0.000559535
**3**	**83.7**	1.003311018	1.003420999	1.001992973	1.000678106	1.001335143	1.002147648	0.001206078
**4**	**31.0**	1.02205038	1.02422828	1.025211125	1.022376522	1.025320437	1.023837349	0.001546521
**5**	**106.5**	1.005699496	1.004907473	1.004116596	1.004568386	1.004342444	1.004726879	0.000617243
**6**	**127.7**	0.998414817	1.001568004	1.000043495	0.999826056	1.001568004	1.000284075	0.001328414
**CN**	**121.7**	1.000223058	0.999108682	1.000557816	0.998552349	0.999888505	0.999666082	0.000822493
**C**	**136.6**	1.000913754	0.999391682	0.999717482	1.001458384	0.99950026	1.000196312	0.000931228
**CH**	**128.1**	1	1	1	1	1	1	0

Table S5.6: ^{13}C Integrations of dihydropyridine ring from **1a** (7% yield of **4h**)

C	ppm	#1	#2	#3	#4	#5	average	SD
**2**	**142.8**	99.89	99.79	99.93	99.87	99.77	99.8500	0.0678233
**3**	**83.7**	98.98	99.09	99.18	99.18	99.13	99.1120	0.082885463
**4**	**31.0**	101.53	101.68	101.63	101.52	101.70	101.6120	0.083486526
**5**	**106.5**	96.37	96.33	96.38	96.45	96.30	96.3660	0.056833089
**6**	**127.7**	99.90	99.75	99.86	99.63	100.00	99.8280	0.14237275
**CN**	**121.7**	97.43	97.41	97.29	97.39	97.43	97.3900	0.058309519
**C**	**136.6**	99.84	99.95	99.94	99.89	99.99	99.9220	0.058051701
**CH**	**128.1**	100	100	100	100	100	100.0000	0

Table S5.7: Calculated ^{13}C KIEs of dihydropyridine ring from **1a** (7% yield of **4h**)

C	ppm	#1	#2	#3	#4	#5	average	SD
**2**	**142.8**	0.999210875	1.000249447	0.998796029	0.999418423	1.000457411	0.999626437	0.000704308
**3**	**83.7**	1.003793274	1.002637692	1.001694121	1.001694121	1.002218115	1.002407465	0.00086992
**4**	**31.0**	1.025232001	1.023664747	1.024186651	1.02533665	1.023456129	1.024375236	0.000872279
**5**	**106.5**	1.00576867	1.00620174	1.005660458	1.004903605	1.006526779	1.00581225	0.000614986
**6**	**127.7**	0.999626242	1.001185347	1.000041545	1.00243601	0.998589435	1.000375716	0.001480244
**CN**	**121.7**	0.999467731	0.999680573	1.000959462	0.999893502	0.999467731	0.9998938	0.000621324
**C**	**136.6**	1.000664858	0.999522659	0.999626391	1.000145365	0.999107936	0.999813442	0.00060257
**CH**	**128.1**	1	1	1	1	1	1	0

KIEs calculation from ^{i}BuMgBr (isobutyl moiety from the product 4h)

Table S6.1: ^{13}C Integrations of initial ^{i}BuMgBr (standard)

C	ppm	#1	#2	#3	#4	#5	average	SD
**3' & 3''**	**29.9**	200	200	200	200	200	200	0
**2'**	**29.0**	98.01	97.33	98.57	97.3	97.35	97.712	0.563844
**1'**	**21.8**	92.75	91.86	92.97	91.81	92.15	92.308	0.526137

Table S6.2: ^{13}C Integrations of isoprobutyl moiety from product **4h** (12% yield of **4h**)

C	ppm	#1	#2	#3	#4	#5	average	SD
**3''**	**22.1**	100.00	100.00	100.00	100.00	100.00	100.0000	0
**3'**	**23.5**	100.1	100.07	100.12	100.08	99.88	100.0500	0.096953597
**2'**	**23.6**	97.9	97.72	97.87	97.66	97.6	97.7500	0.130766968
**1'**	**48.9**	90.61	90.65	90.77	90.66	90.42	90.6220	0.12755391

Table S6.3: Calculated ^{13}C KIEs of isoprobutyl moiety from product **4h** (12% yield of **4h**)

C	ppm	#1	#2	#3	#4	#5	average	SD
**3''**	**22.1**	1	1	1	1	1	1	0
**3'**	**23.5**	0.998934335	0.999253811	0.998721457	0.999147298	1.001281611	0.999467702	0.001034413
**2'**	**23.6**	0.997951525	0.999912671	0.998277882	1.00056799	1.001224114	0.999586836	0.001426281
**1'**	**48.9**	1.019989609	1.019510111	1.018074148	1.019390302	1.022273011	1.019847436	0.001530599

Table S6.4: ^{13}C Integrations of isoprobutyl moiety from product **4h** (15% yield of **4h**)

C	ppm	#1	#2	#3	#4	#5	average	SD
**3''**	**22.1**	100.00	100.00	100.00	100.00	100.00	100.0000	0
**3'**	**23.5**	100,03	99,95	100,08	100,02	100	100,0160	0,047222876
**2'**	**23.6**	97,83	97,5	97,8	97,83	97,52	97,6960	0,170381924
**1'**	**48.9**	90,66	90,38	90,73	90,47	90,79	90,6060	0,174441967

Table S6.5: Calculated ^{13}C KIEs of isoprobutyl moiety from product **4h** (15% yield of **4h**)

C	ppm	#1	#2	#3	#4	#5	average	SD
**3''**	**22.1**	1	1	1	1	1	1	0
**3'**	**23.5**	0.999674344	1.000543194	0.999132016	0.999782874	1	0.999826485	0.00051262
**2'**	**23.6**	0.998690277	1.002361008	0.999022958	0.998690277	1.002137833	1.00018047	0.001895205
**1'**	**48.9**	1.019737465	1.023162287	1.018884555	1.022059145	1.018154534	1.020399597	0.002130765

Table S6.6: ^{13}C Integrations of isoprobutyl moiety from product **4h** (7% yield of **4h**)

C	ppm	#1	#2	#3	#4	#5	average	SD
**3''**	**22.1**	100.00	100.00	100.00	100.00	100.00	100.0000	0
**3'**	**23.5**	100.09	100.11	99.96	99.98	100.19	100.0660	0.095551033
**2'**	**23.6**	97.92	97.55	97.76	97.66	97.73	97.7240	0.136124943
**1'**	**48.9**	90.47	90.58	90.63	89.99	90.66	90.4660	0.27573538

Table S6.7: Calculated ^{13}C KIEs of isoprobutyl moiety from product **4h** (7% yield of **4h**)

C	ppm	#1	#2	#3	#4	#5	average	SD
**3''**	**22.1**	1	1	1	1	1	1	0
**3'**	**23.5**	0.999067378	0.998860356	1.000415038	1.000207477	0.998033095	0.999316669	0.000989682
**2'**	**23.6**	0.997796839	1.001722429	0.999490747	1.000552256	0.999808971	0.999874248	0.001444163
**1'**	**48.9**	1.021071275	1.019786161	1.01920305	1.026715791	1.018853491	1.021125954	0.003236741

Table S7: Experimental ^{13}C KIEs at natural abundance from Product analysis

	**Experimental** [a]		
	**Exp. 1**	**Exp. 2**	**Exp. 3**
CN	1.000 (1)	1.000 (1)	1.000 (1)
C2	1.000 (1)	1.000 (1)	1.000 (1)
C3	1.001 (1)	1.002 (1)	1.002 (1)
C4	***1.025 (1)***	***1.024 (2)***	***1.024 (1)***
C5	1.005 (1)	1.005 (2)	1.006 (1)
C6	1.001 (1)	1.000 (1)	1.000 (1)
C	1.000 (1)	1.000 (1)	1.000 (1)
CH		1.000 (Reference)	
C1'	***1.020 (2)***	***1.020 (2)***	***1.021 (3)***
C2'	1.000 (1)	1.000 (2)	1.000 (1)
C3'	0.999 (1)	1.000 (1)	0.999 (1)
C3''		1.000 (Reference)	

[a] Three experiments were carried to 12, 15, and 7% yield to determine the KIEs for incoming 1,4-dihydropyridine and isobutyl group. The numbers in parenthesis represent the standard deviation in the last digit as determined from five independent measurements.

7. Determination of Absolute Configuration

To determine the absolute configuration of the products, we run an ECD spectra of the enantiomeric form of the obtained product **4c** and we compared them with their predicted ECD spectra. Comparing the computational results with the experimental CD spectra, the computational spectra of (*R*)-**4c** was in good agreement with the experimental results. We conclude that the product obtained is the *R* enantiomer.

a) Experimental ECD spectrum of 4c.

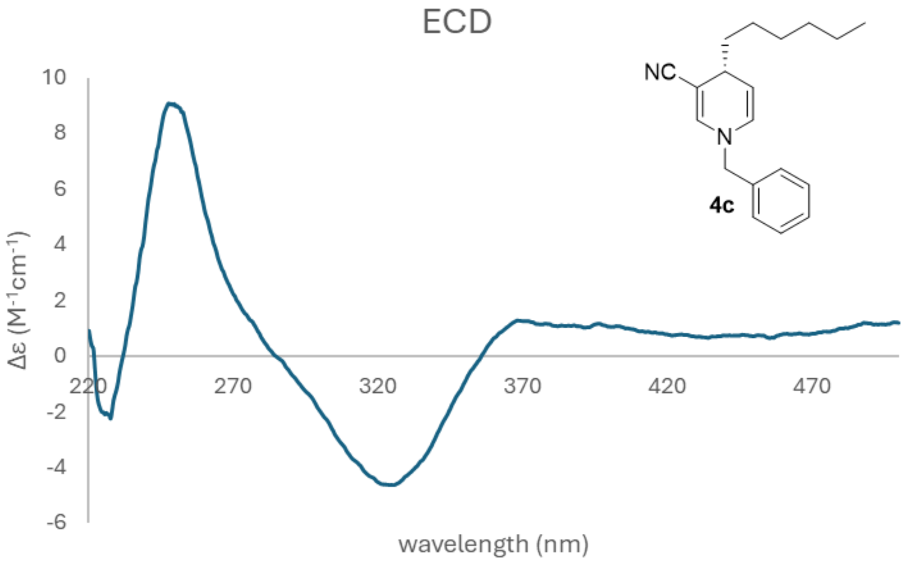

b) Simulated ECD spectrum of 4c.

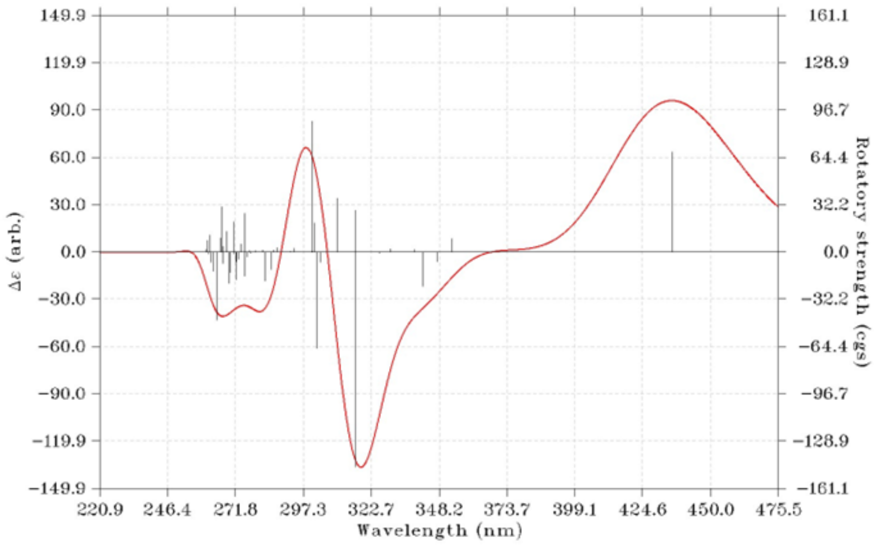

Figure S2: Determination of absolute configuration. a) Experimentally obtained ECD spectra of **4c**. b) Predicted ECD spectra of the most stable conformer of **4c**.

8. NMR Spectra

NMR spectra of *N*-benzyl-3-cyanopyridinium bromide (1a)

^{1}H NMR with DMSO-d$_6$, 400 MHz

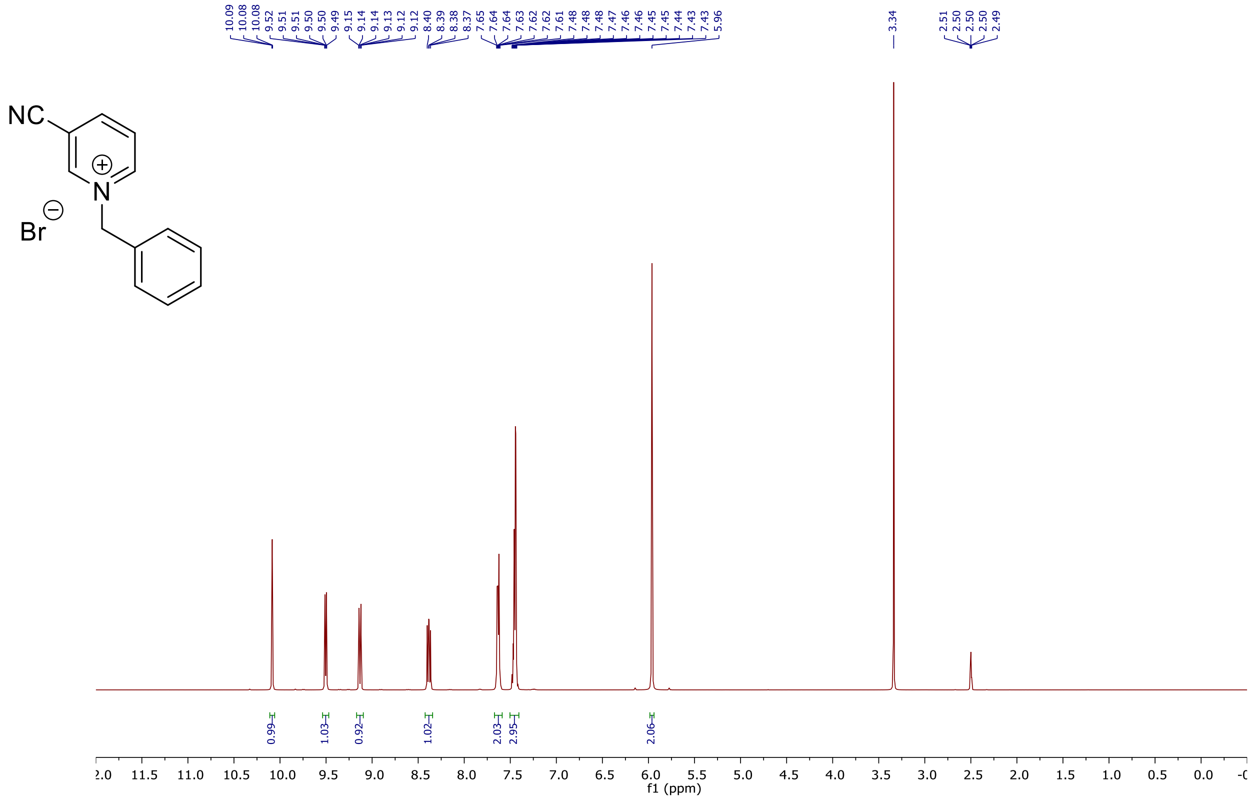

^{13}C NMR with DMSO-d$_6$, 101 MHz

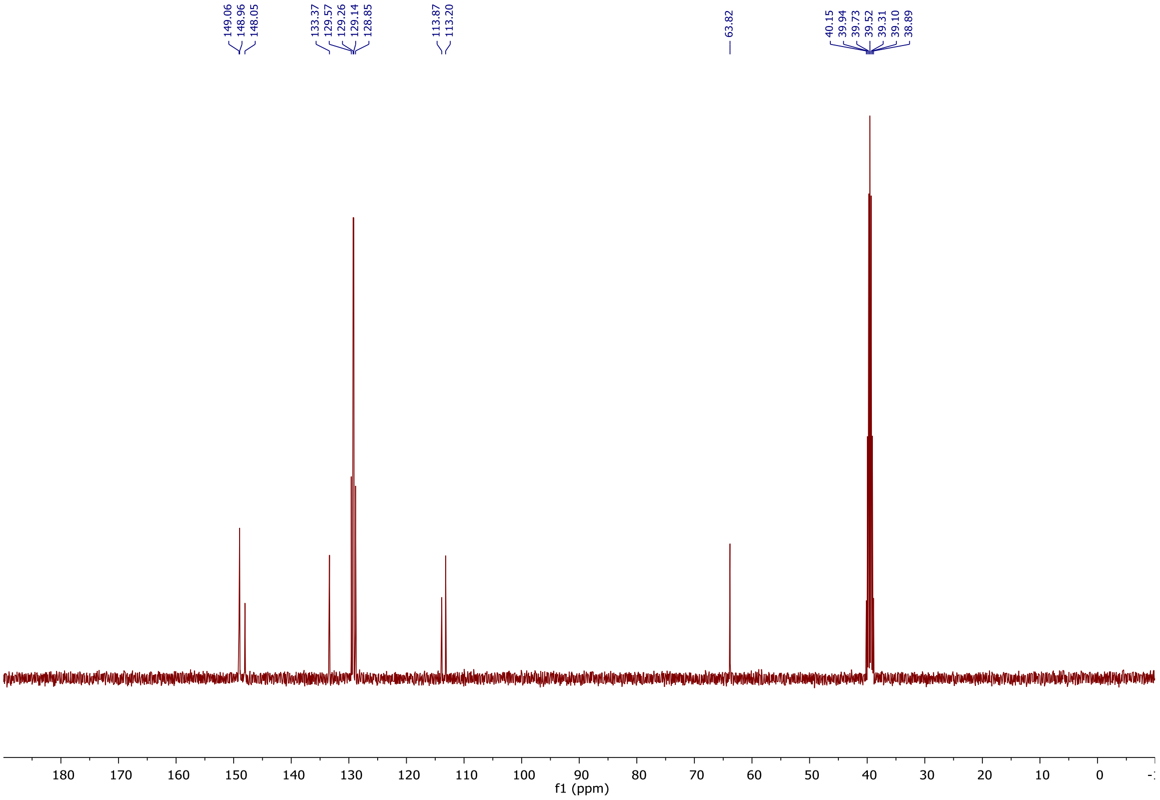

NMR spectra of *N*-(4-methylbenzyl)-3-cyanopyridinium bromide (1b)

^{1}H NMR with DMSO-d$_6$, 400 MHz

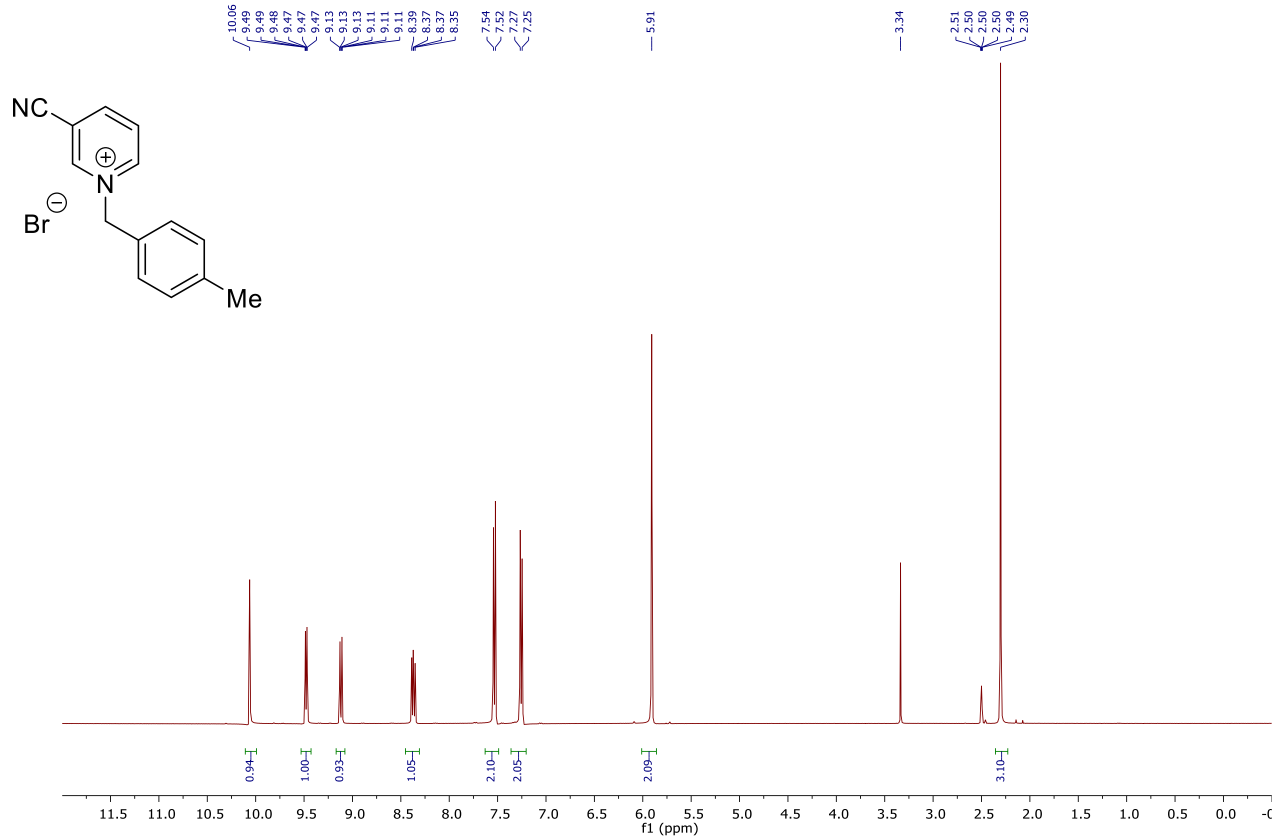

^{13}C NMR with DMSO-d$_6$, 101 MHz

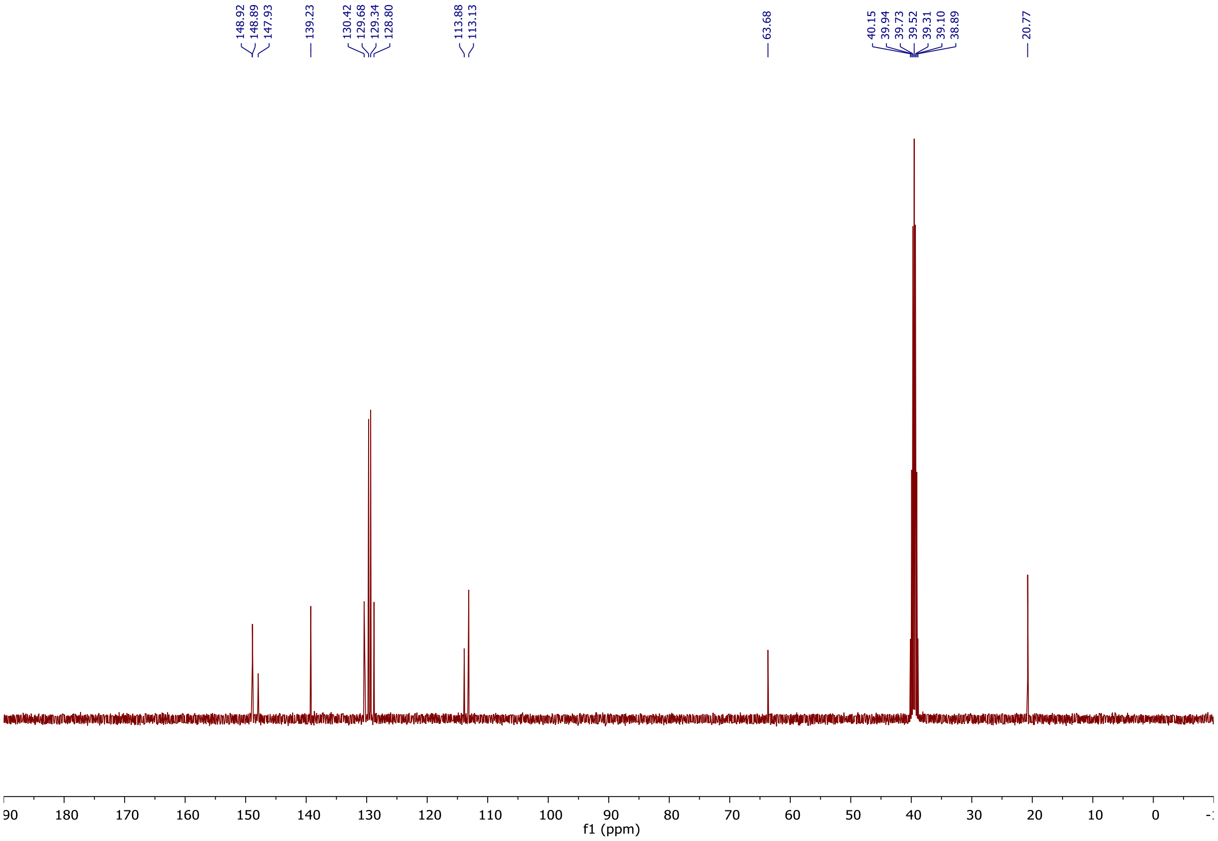

NMR spectra of *N*-(4-(*tert*-butyl)benzyl)-3-cyanopyridinium bromide (1c)

^{1}H NMR with DMSO-d_6, 400 MHz

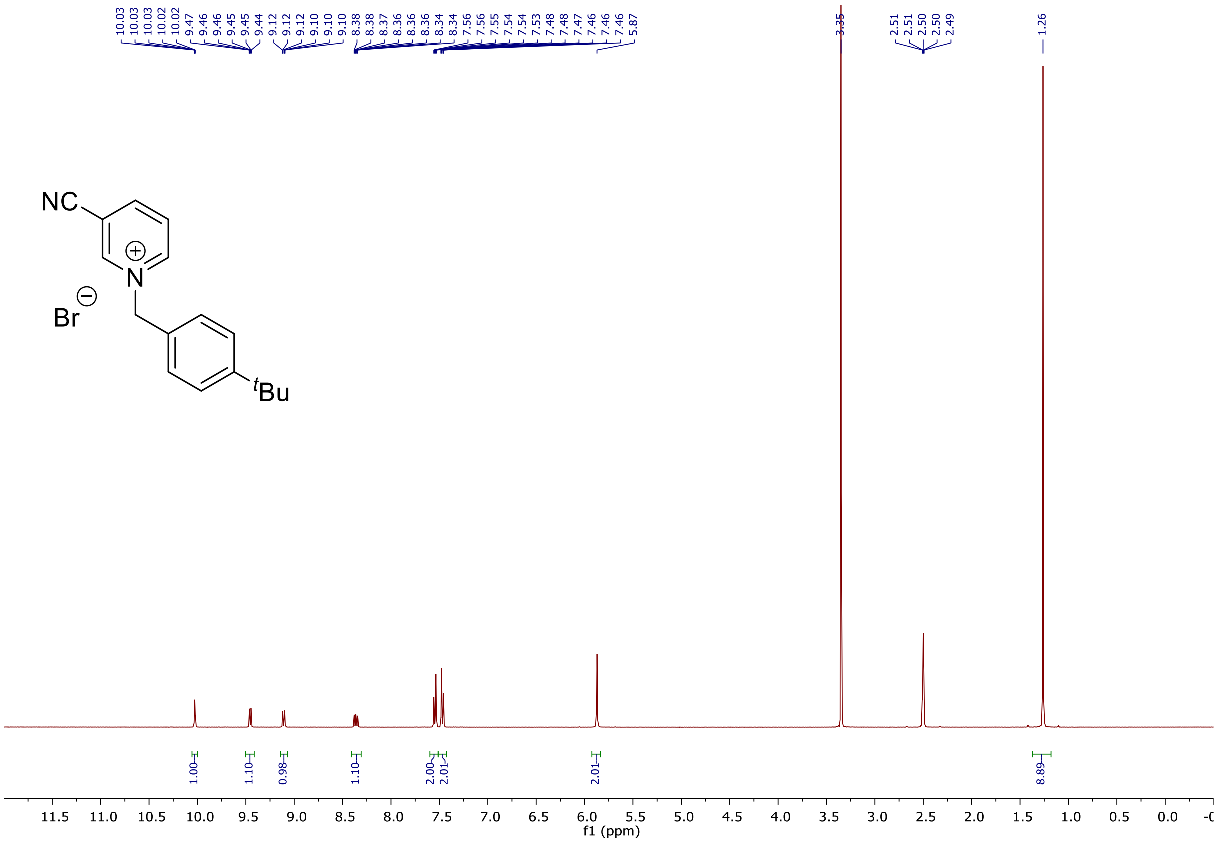

^{13}C NMR with DMSO-d_6, 101 MHz

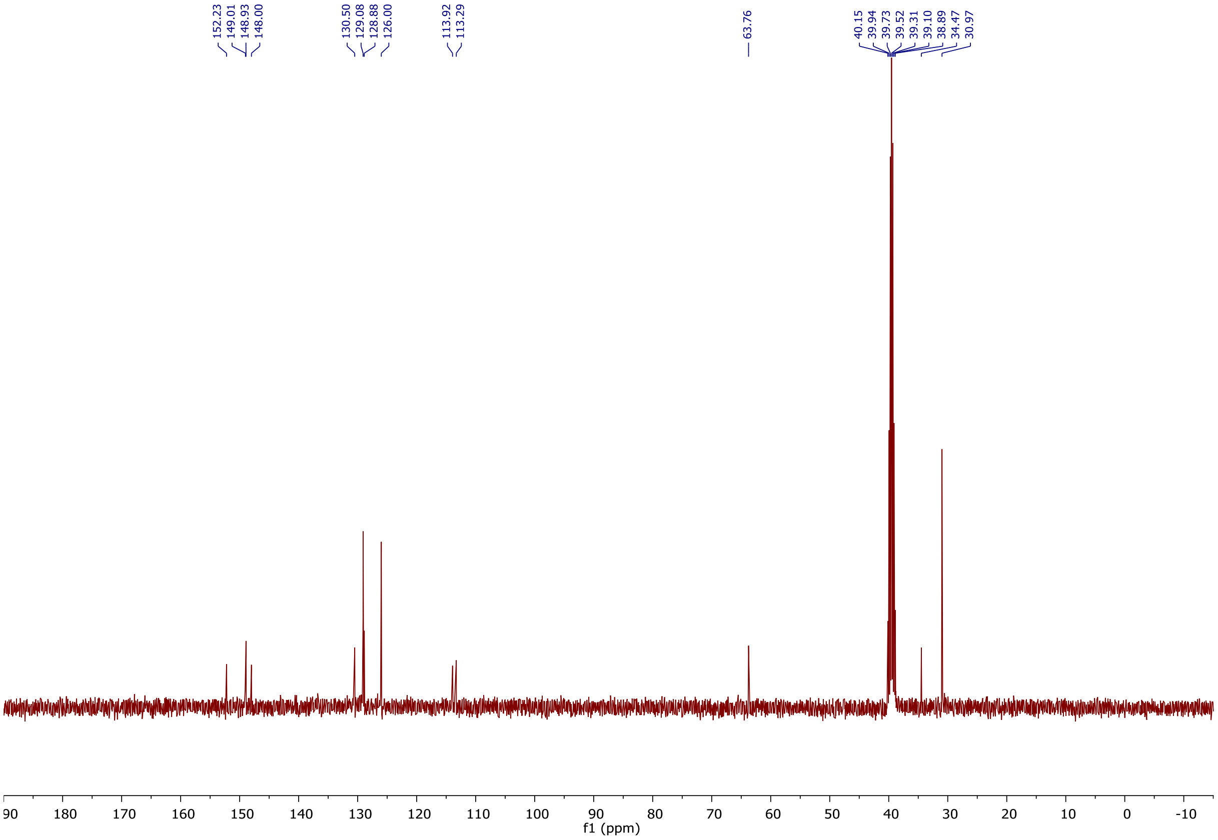

NMR spectra of *N*-(4-(trifluoromethyl)benzyl)-3-cyanopyridinium bromide (1d)

^{1}H NMR with DMSO-d$_6$, 400 MHz

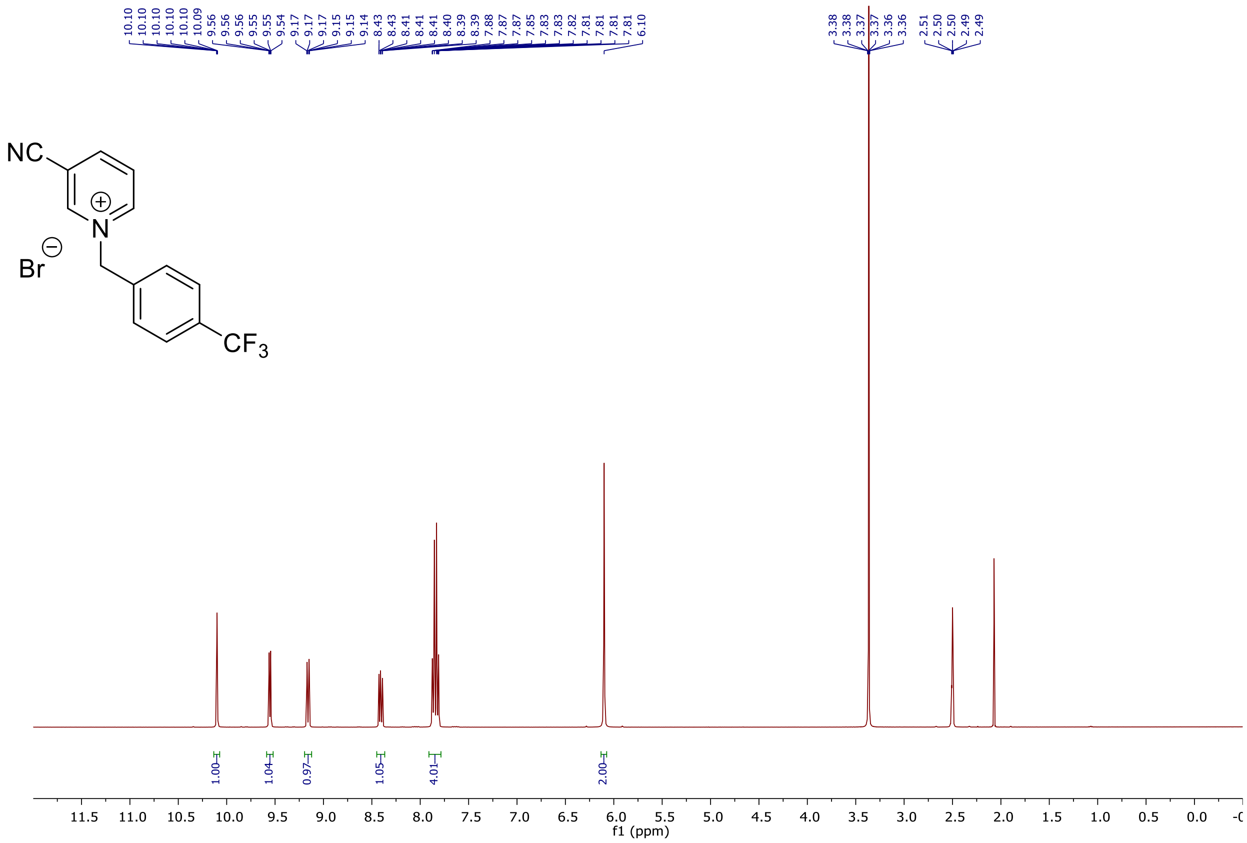

^{13}C NMR with DMSO-d$_6$, 101 MHz

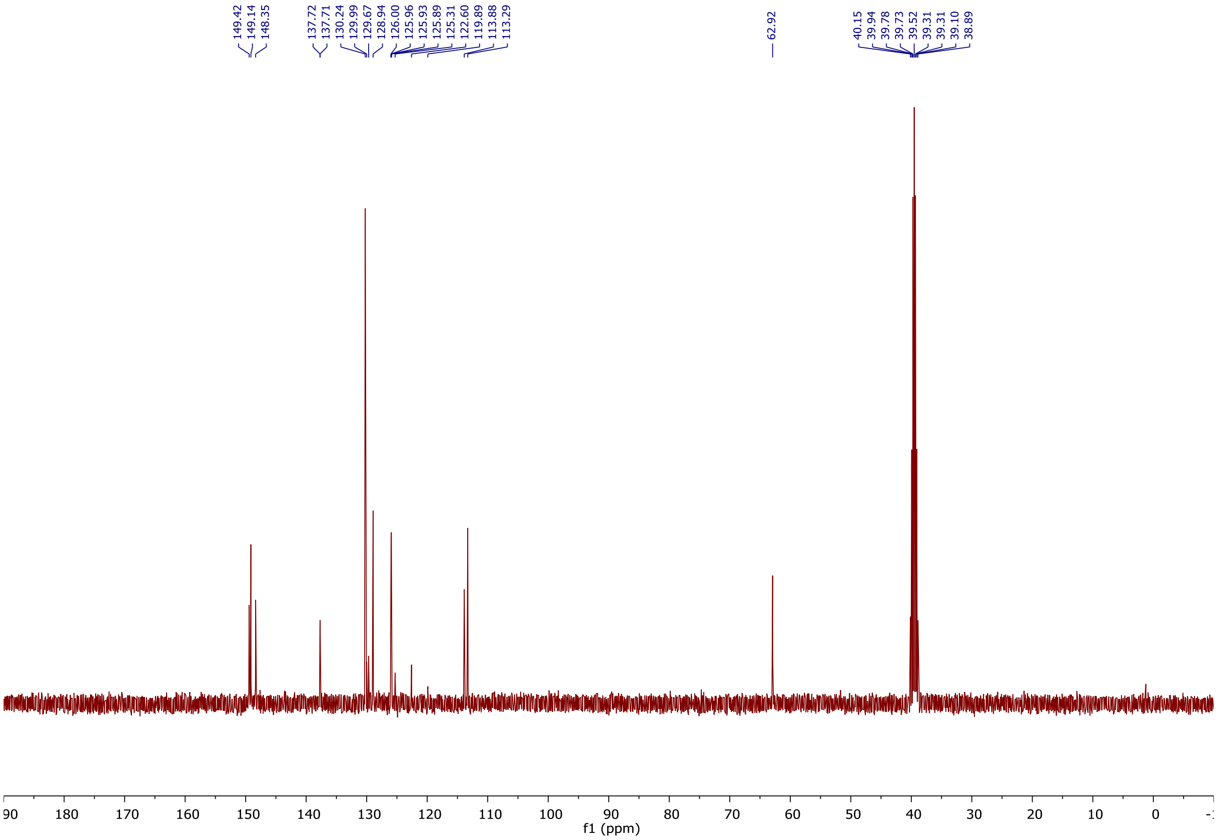

^{19}F NMR with DMSO-d_6, 376 MHz

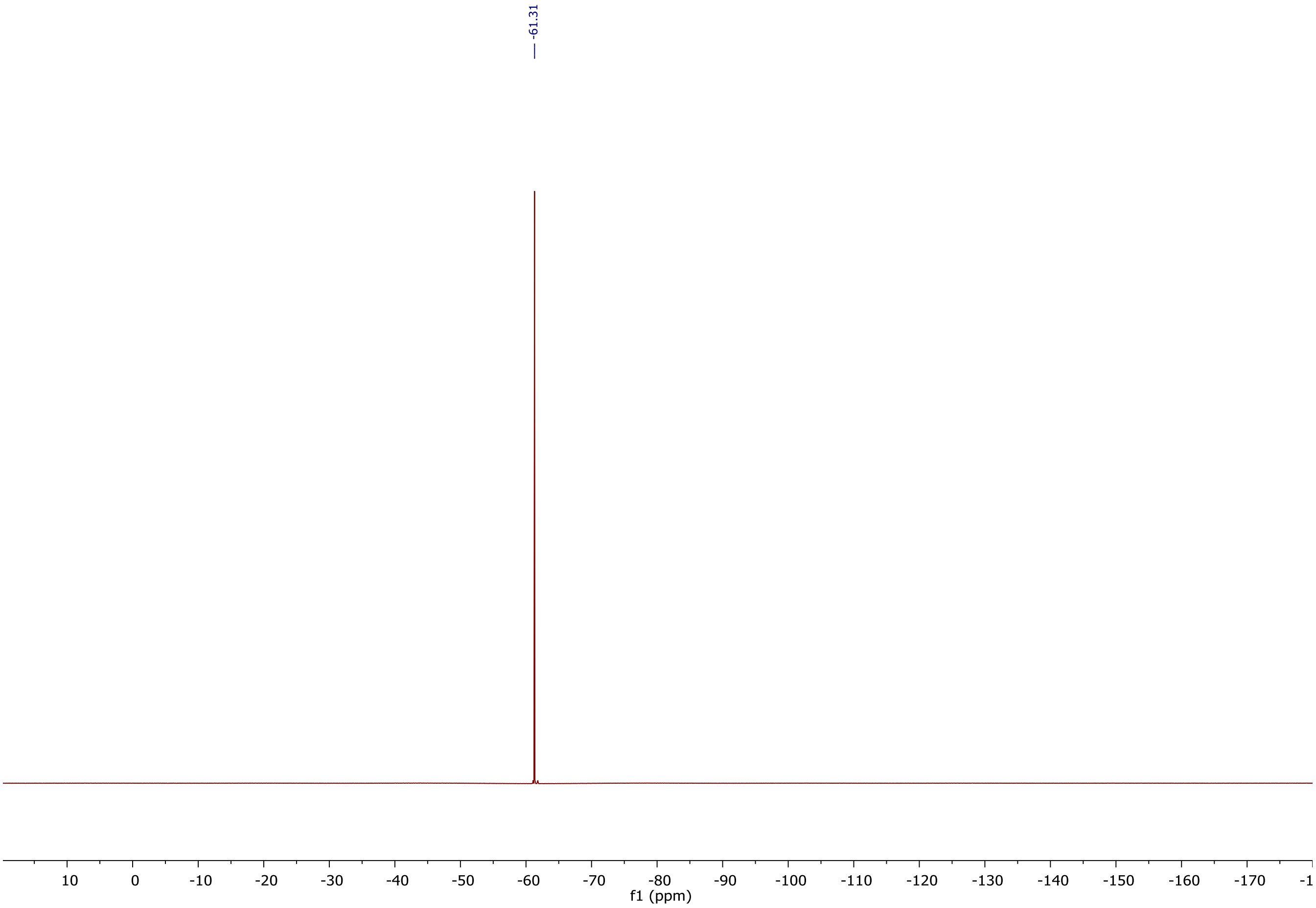

NMR spectra of *N*-(4-fluorobenzyl)-3-cyanopyridinium bromide (1e)

^{1}H NMR with DMSO-d$_6$, 400 MHz

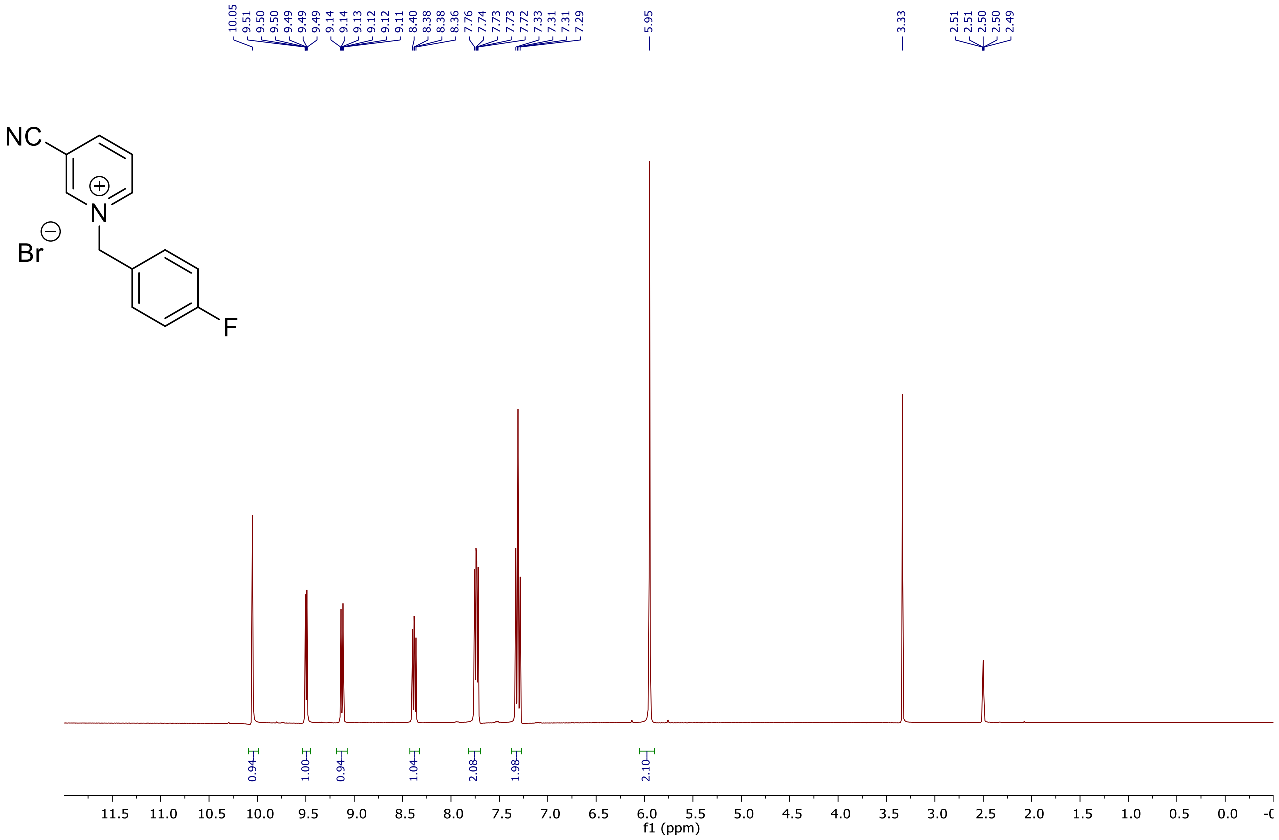

^{13}C NMR with DMSO-d$_6$, 101 MHz

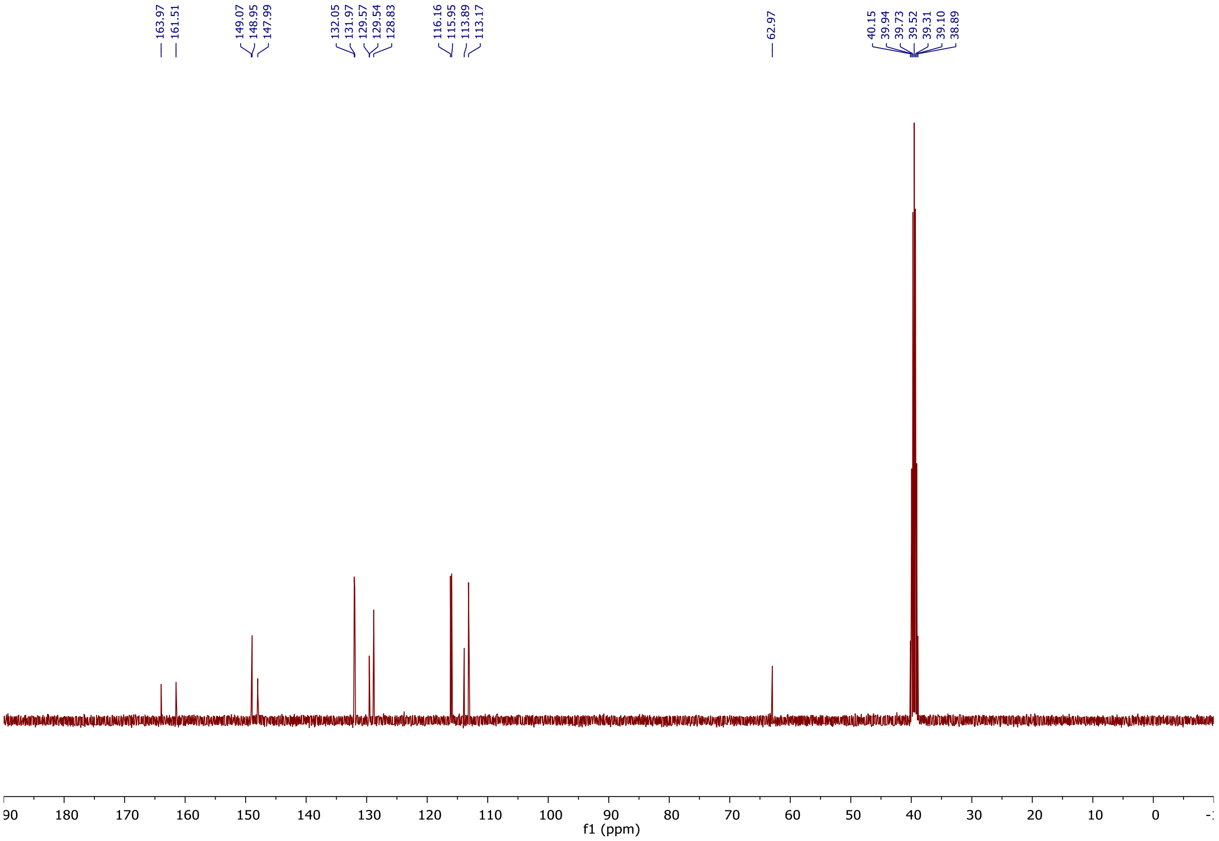

^{19}F NMR with DMSO-d$_6$, 376 MHz

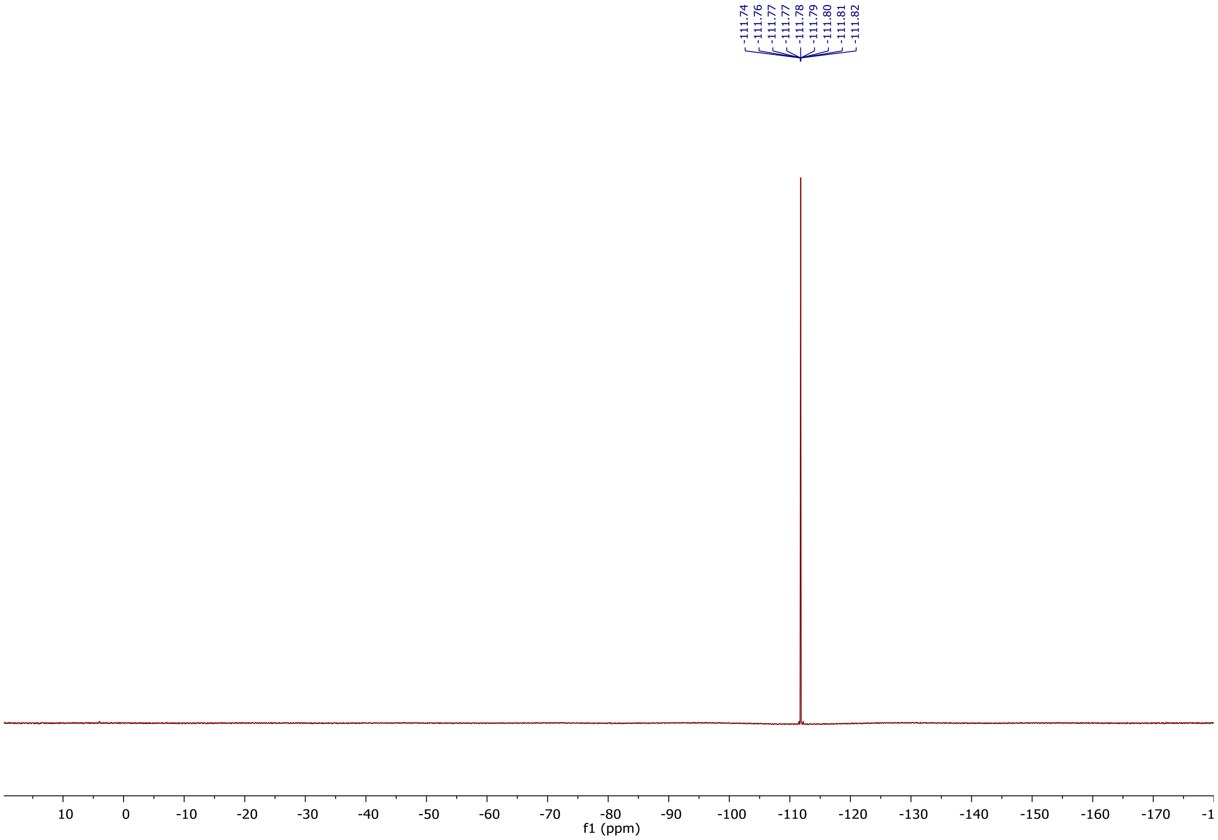

NMR spectra of *N*-(naphthalen-2-ylmethyl)-3-cyanopyridinium bromide (1f)

^{1}H NMR with DMSO-d$_6$, 400 MHz

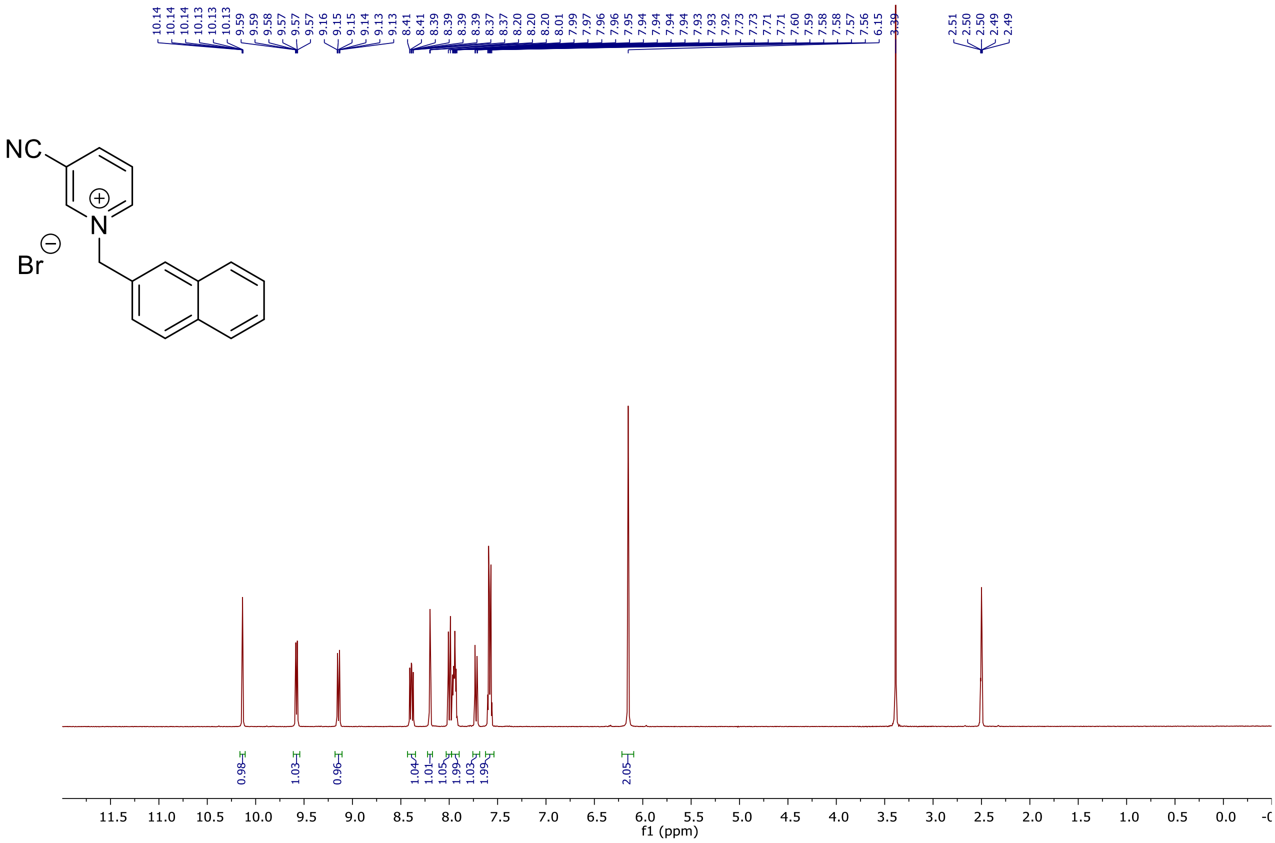

^{13}C NMR with DMSO-d$_6$, 101 MHz

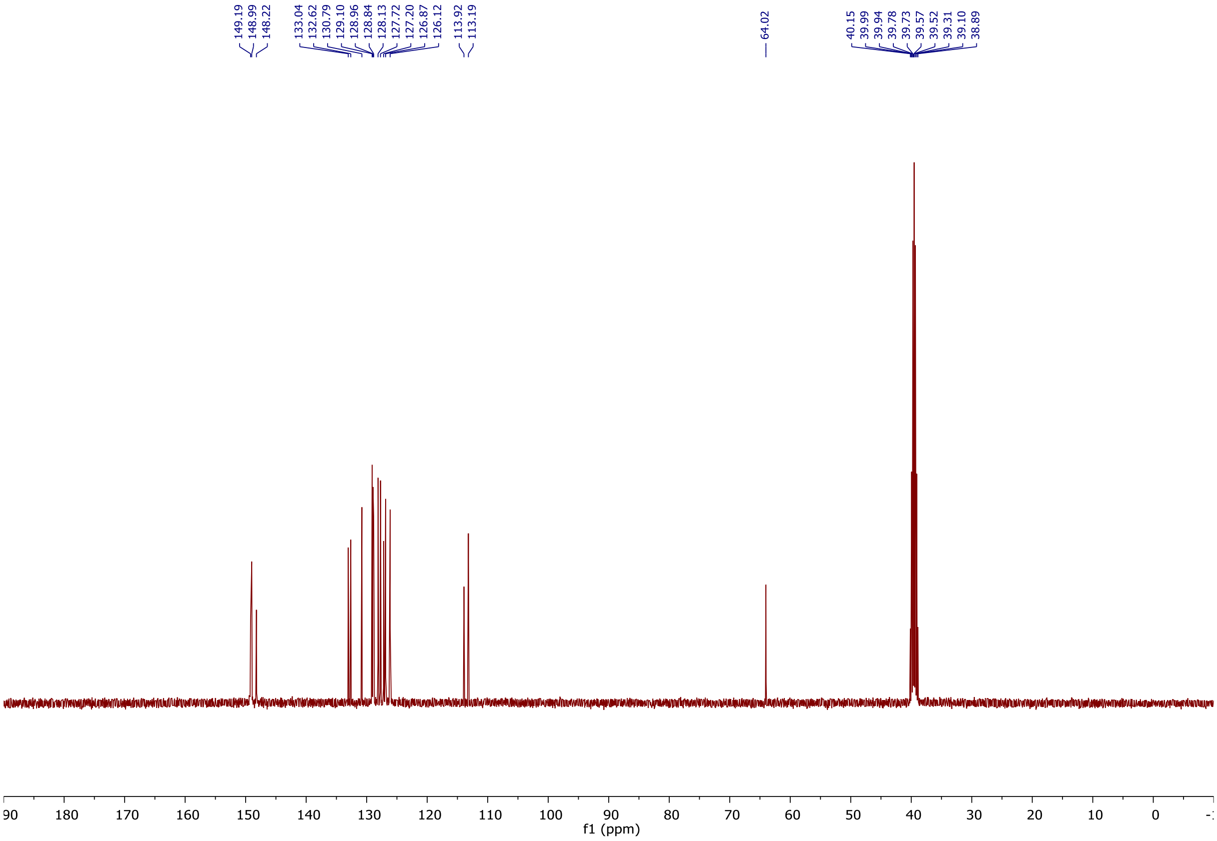

NMR spectra of *N*-(3-methylbenzyl)-3-cyanopyridinium bromide (1g)

^{1}H NMR with DMSO-d$_6$, 400

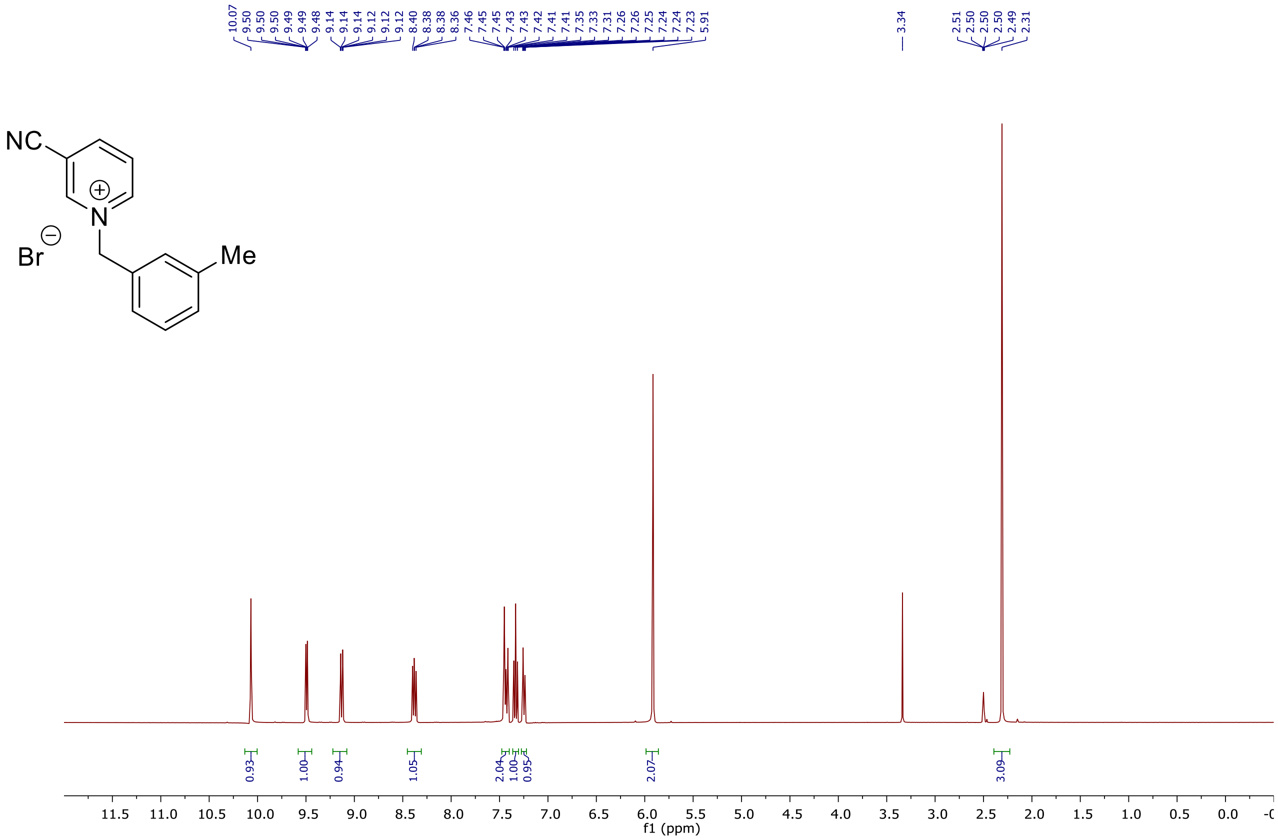

^{13}C NMR with DMSO-d$_6$, 101 MHz

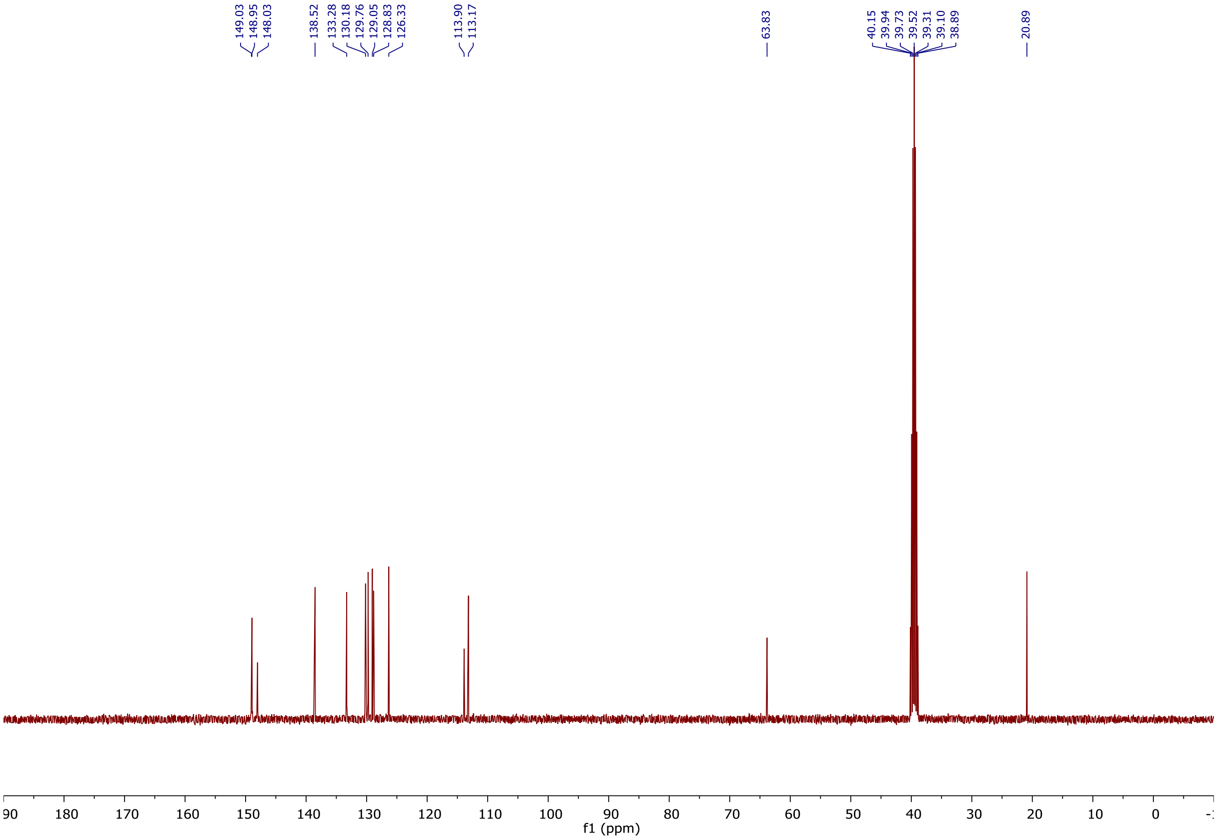

NMR spectra of *N*-(2-methylbenzyl)-3-cyanopyridinium bromide (1h)

^{1}H NMR with DMSO-d$_6$, 400 MHz

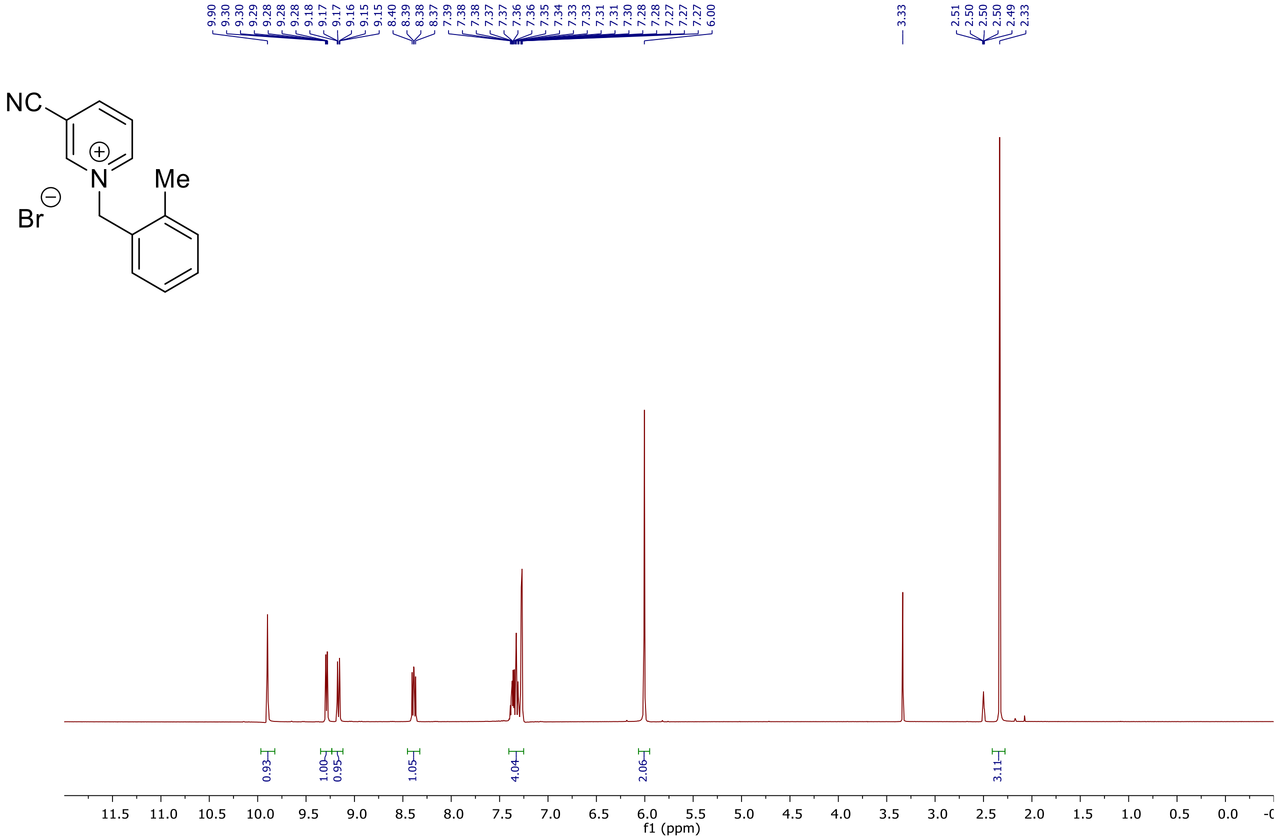

^{13}C NMR with DMSO-d$_6$, 101 MHz

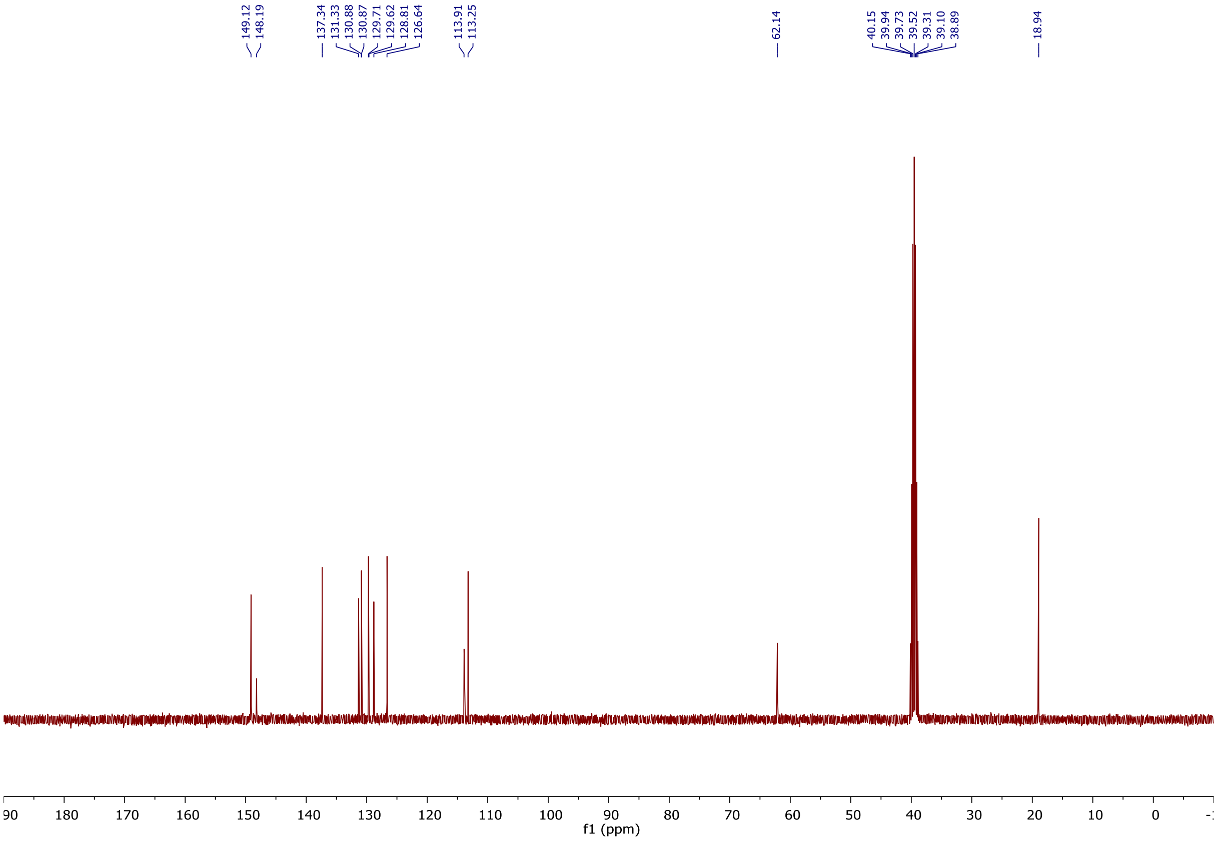

NMR spectra of *N*-(3,5-di-*tert*-butylbenzyl)-3-cyanopyridinium bromide (1i)

^{1}H NMR with DMSO-d$_6$, 400 MHz

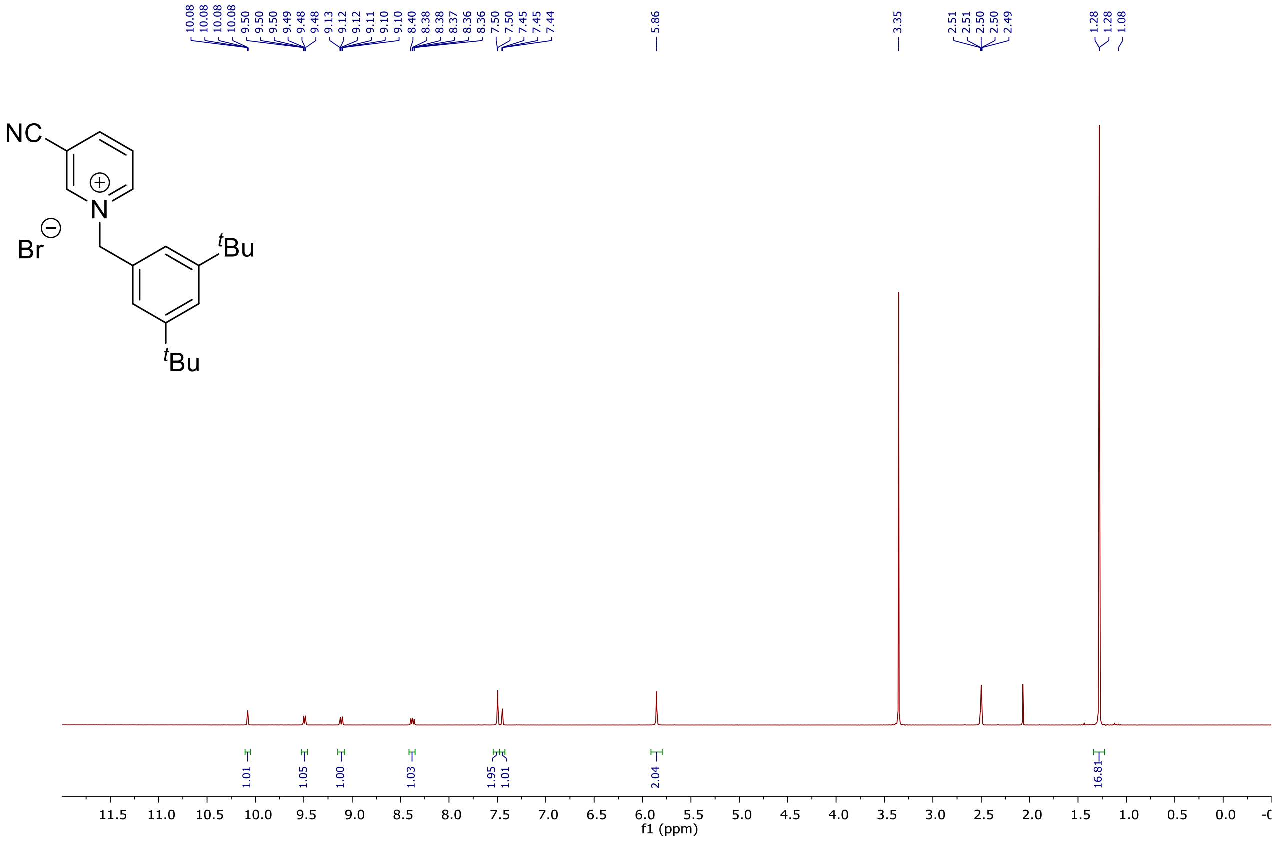

^{13}C NMR with DMSO-d$_6$, 101 MHz

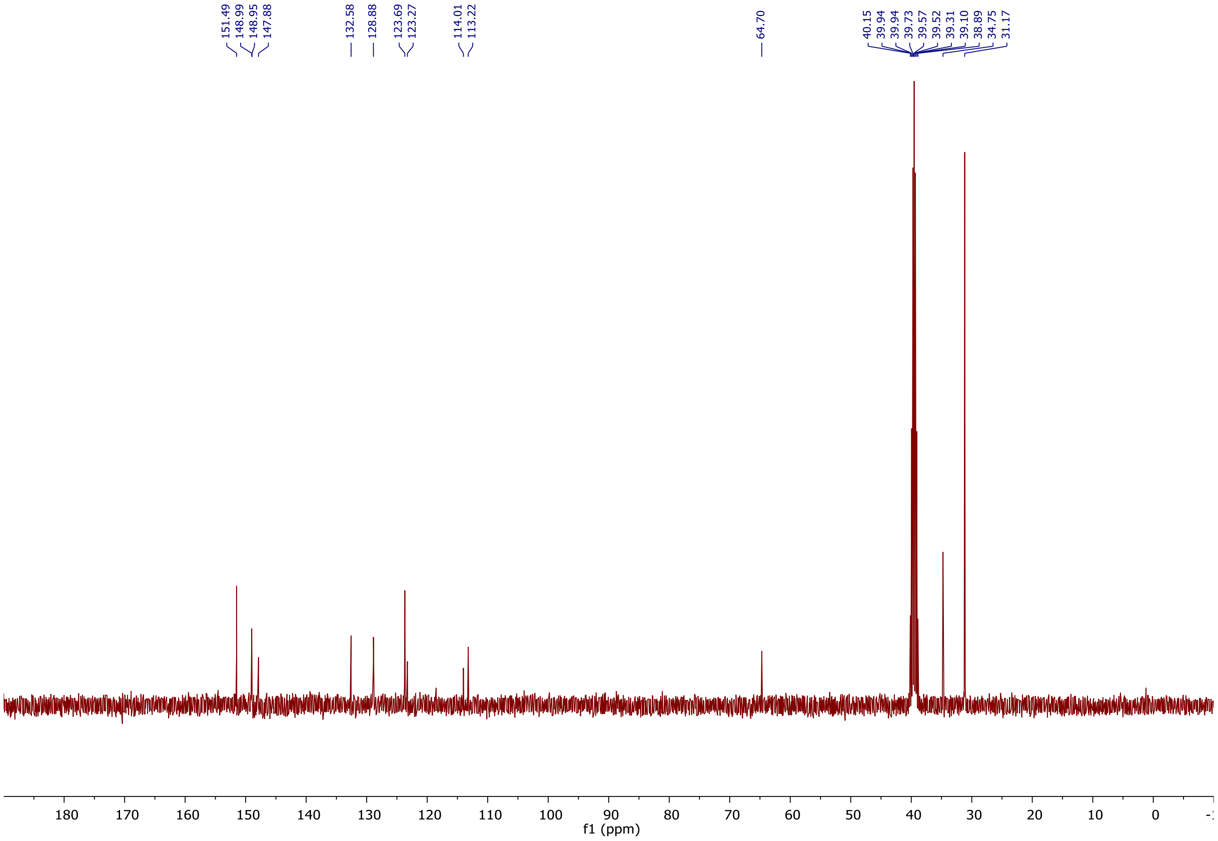

NMR spectra of *N*-(3,5-dimethoxybenzyl)-3-cyanopyridinium bromide (1j)

^{1}H NMR with DMSO-d6, 400 MHz

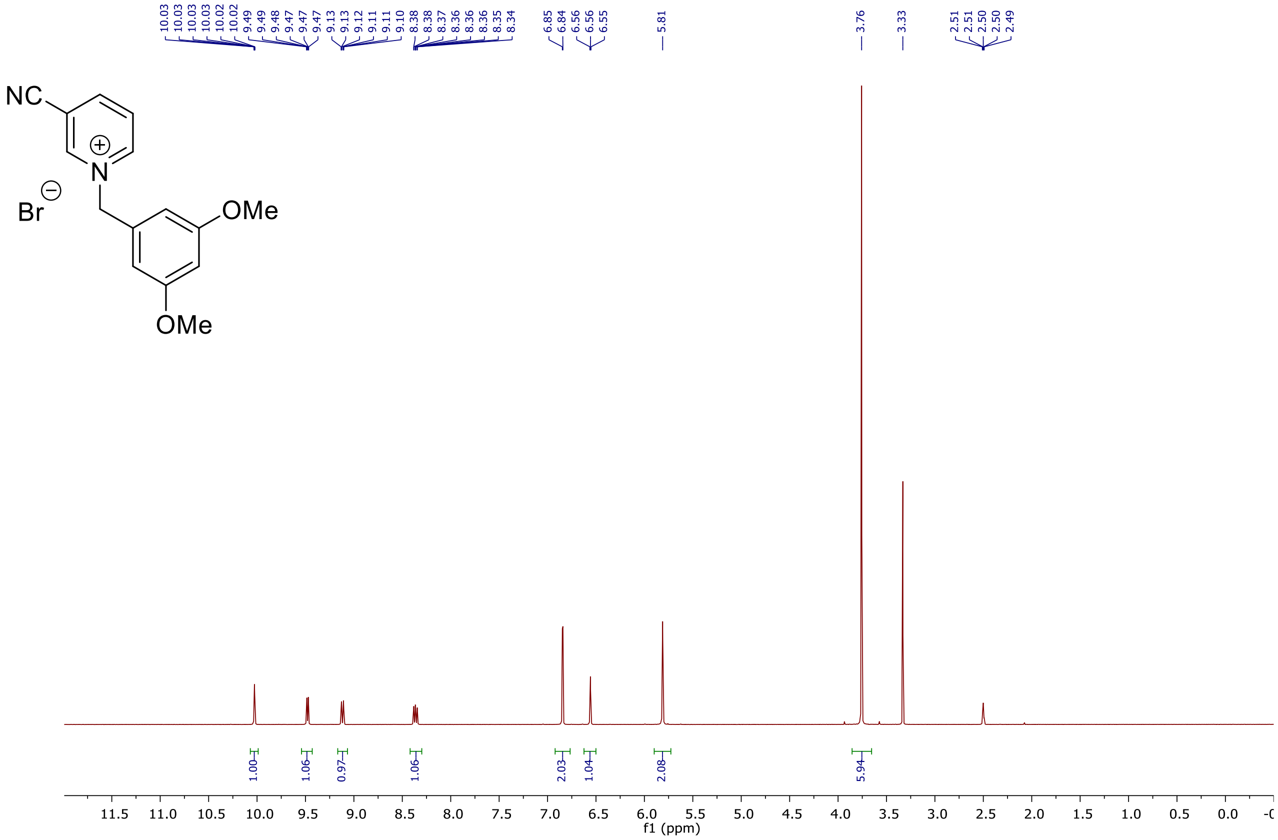

^{13}C NMR with DMSO-d6, 101 MHz

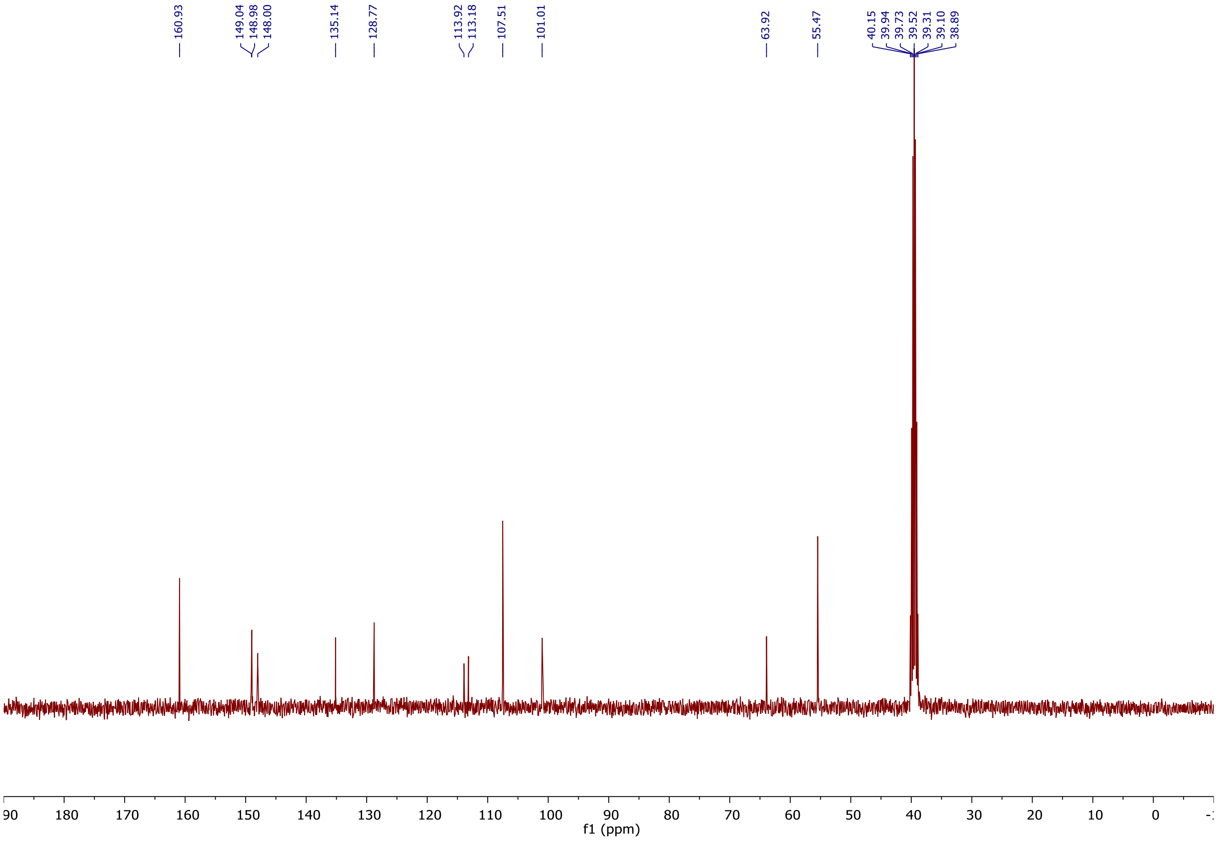

NMR spectra of *N*-(3,5-bis(trifluoromethyl)benzyl)-3-cyanopyridinium bromide (1k)

^{1}H NMR with DMSO-d$_6$, 400 MHz

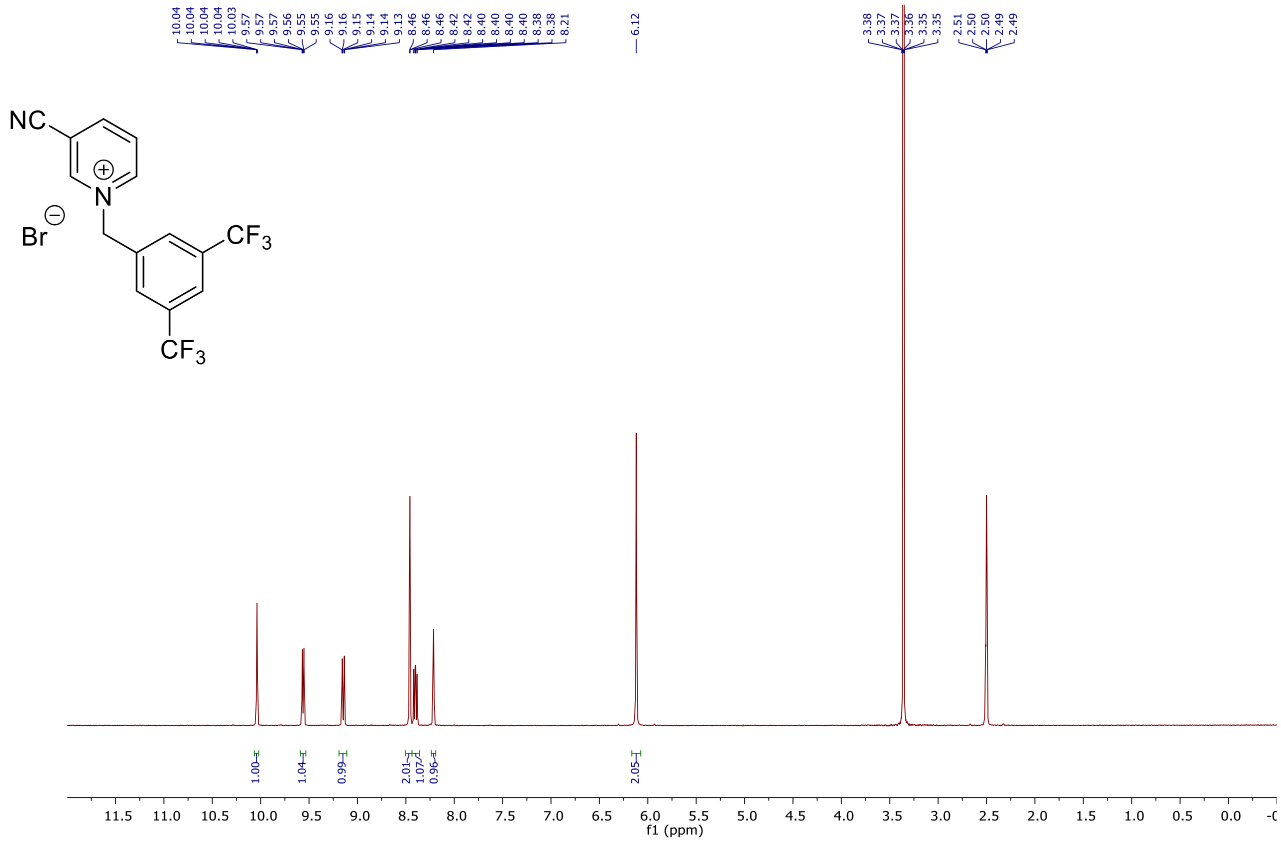

^{13}C NMR with DMSO-d$_6$, 101 MHz

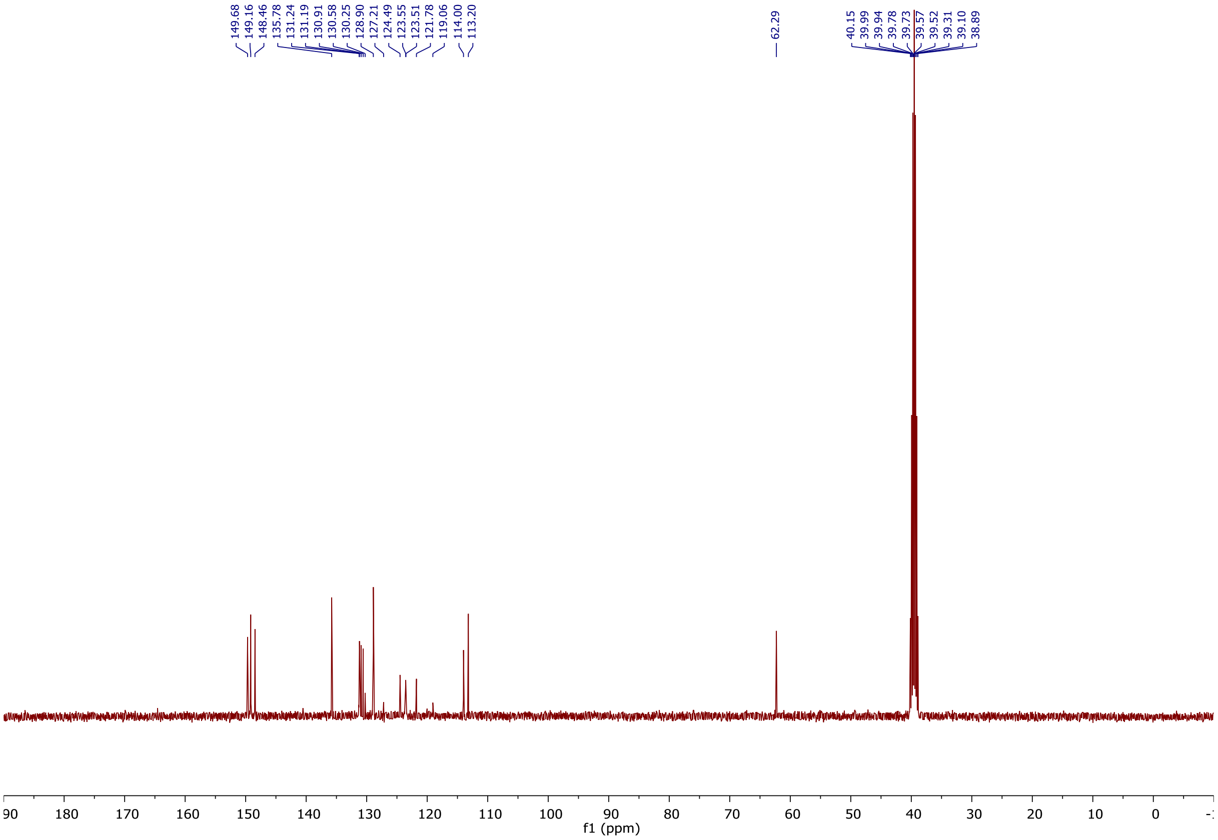

^{19}F NMR with DMSO-d_6, 376 MHz

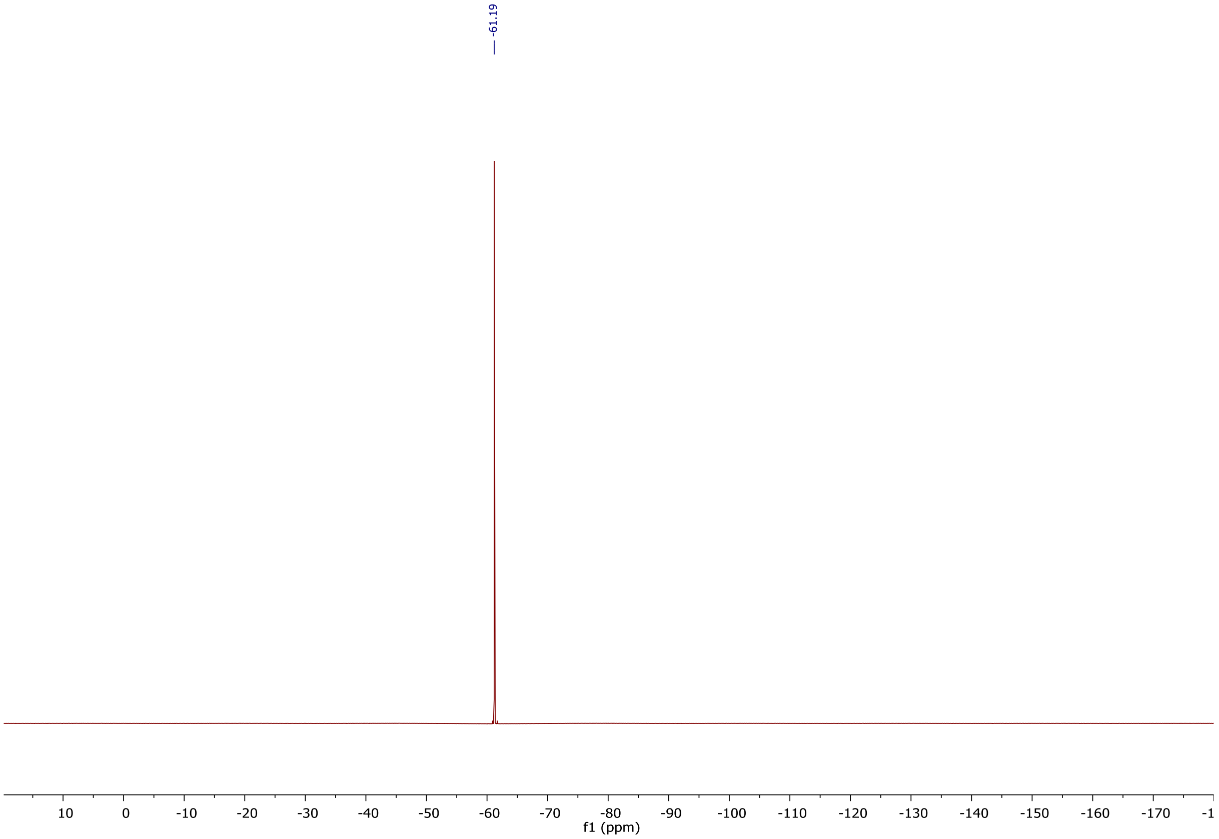

NMR spectra of *N*-methyl-3-cyanopyridinium iodide (1l)

^{1}H NMR with DMSO-d$_6$, 400 MHz

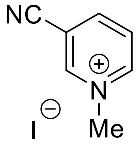

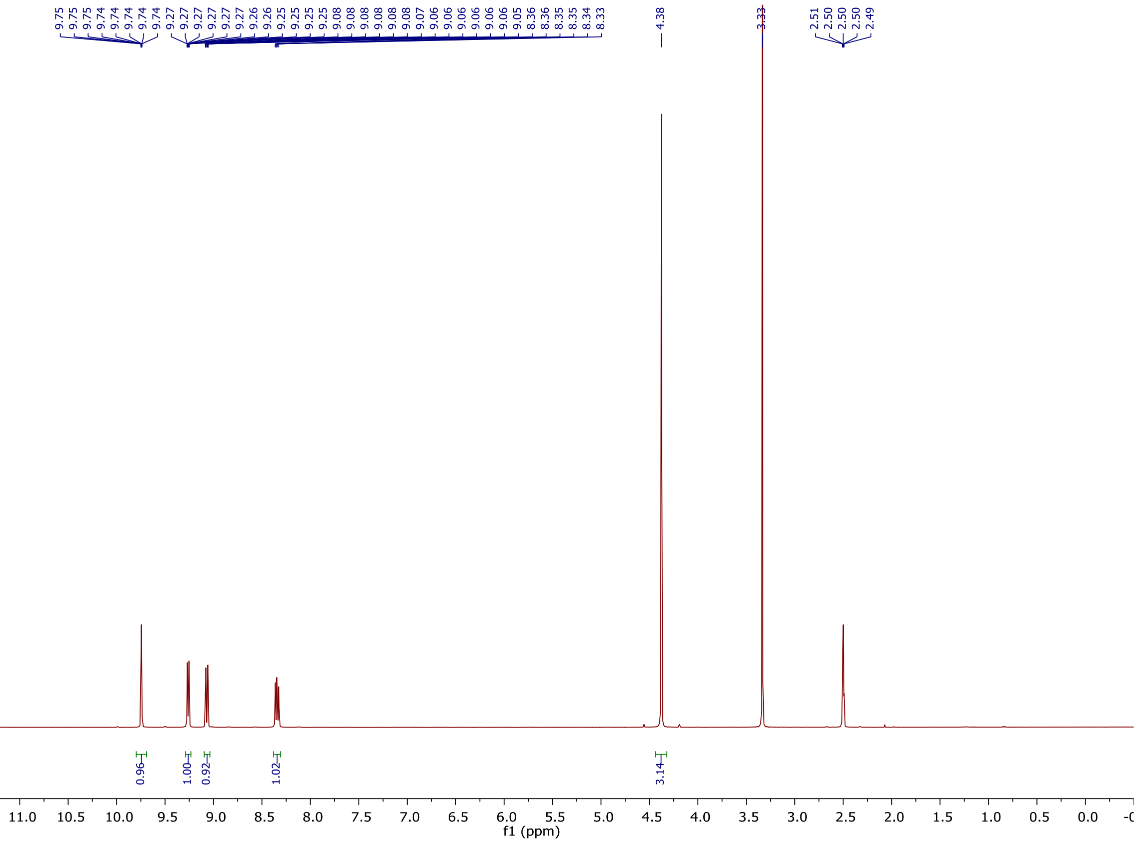

^{13}C NMR with DMSO-d$_6$, 101 MHz

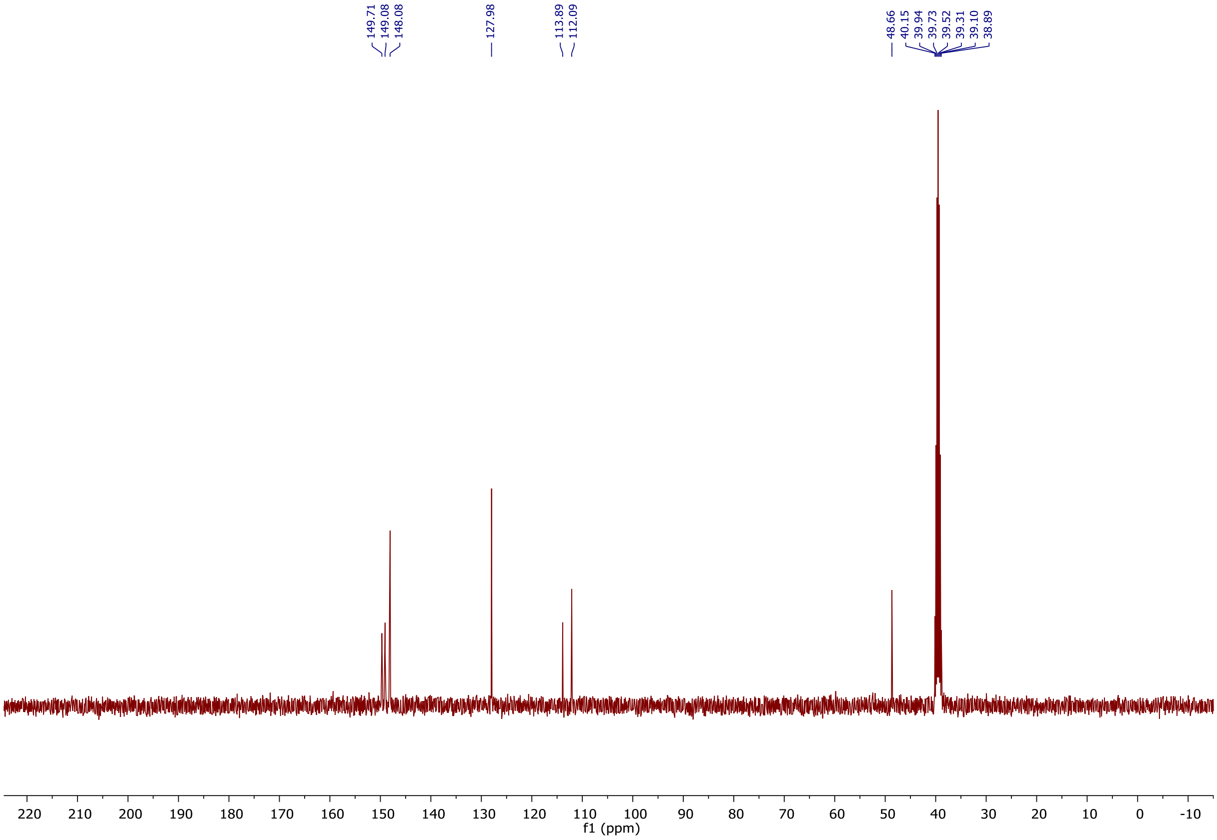

NMR spectra of *N*-propyl-3-cyanopyridinium bromide (1m)

^{1}H NMR with DMSO-d$_6$, 400 MHz

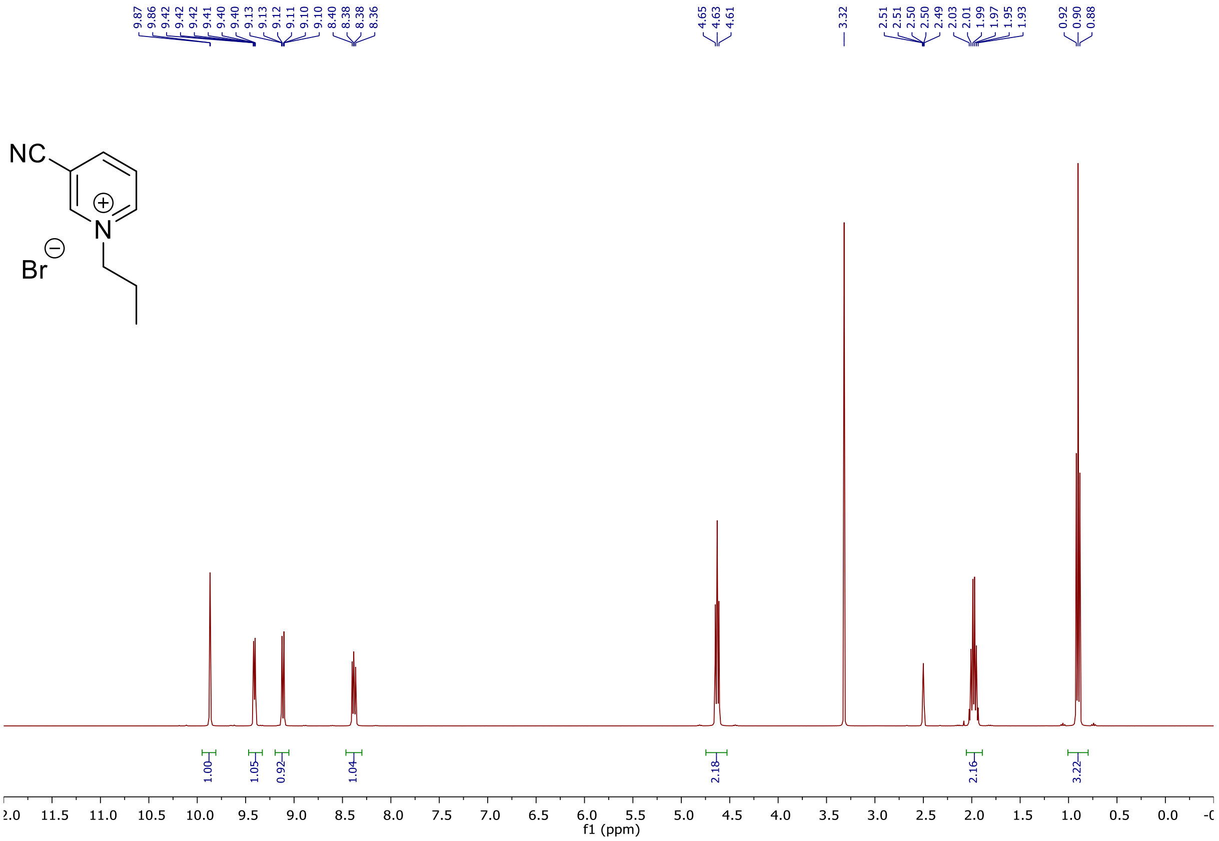

^{13}C NMR with DMSO-d$_6$, 101 MHz

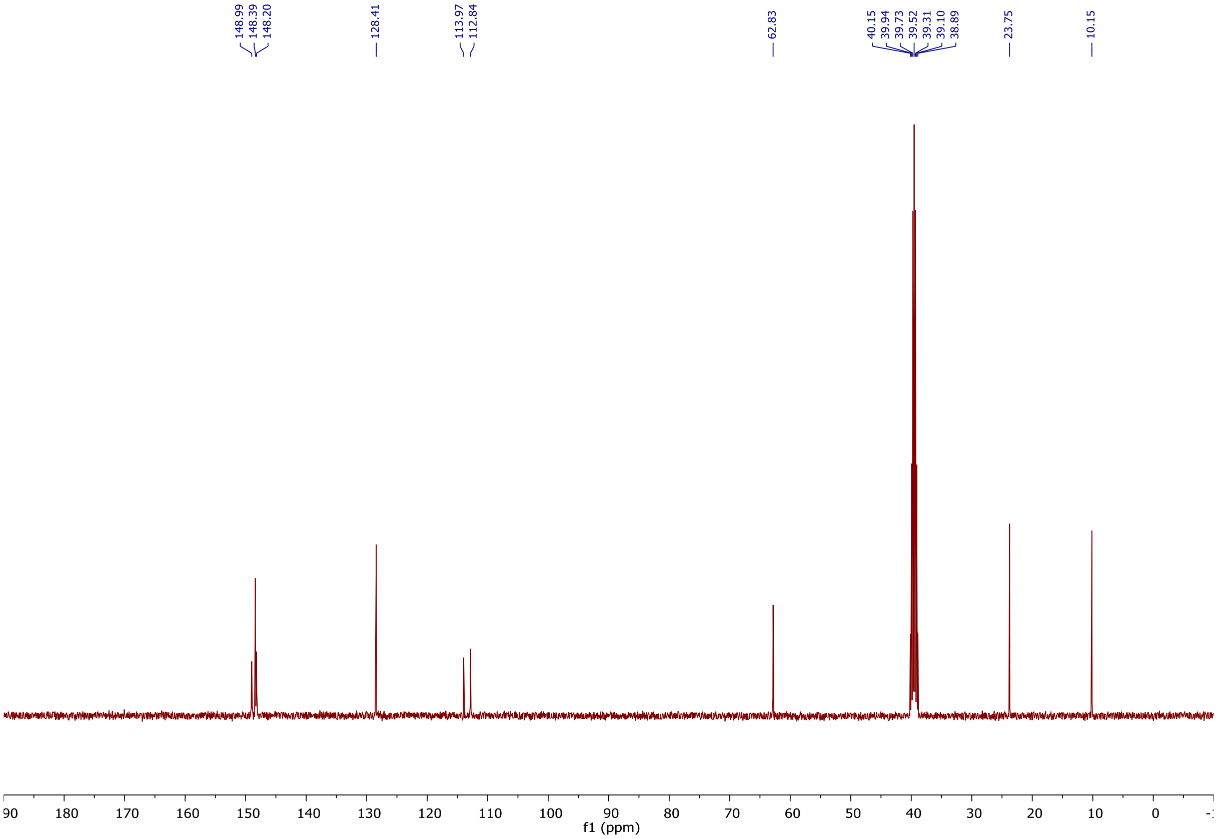

NMR spectra of *N*-(but-3-en-1-yl)-3-cyanopyridinium bromide (1n)

^{1}H NMR with DMSO-d$_6$, 400 MHz

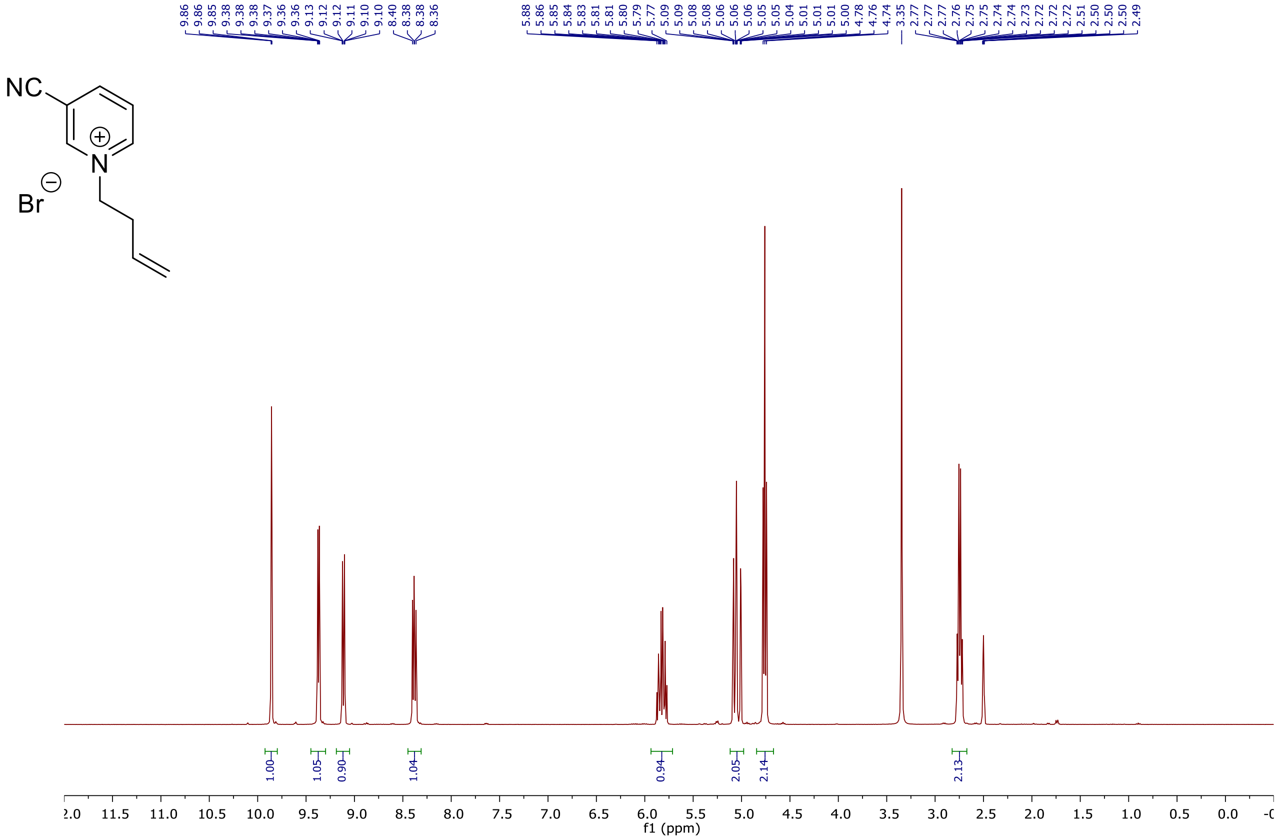

^{13}C NMR with DMSO-d$_6$, 101 MHz

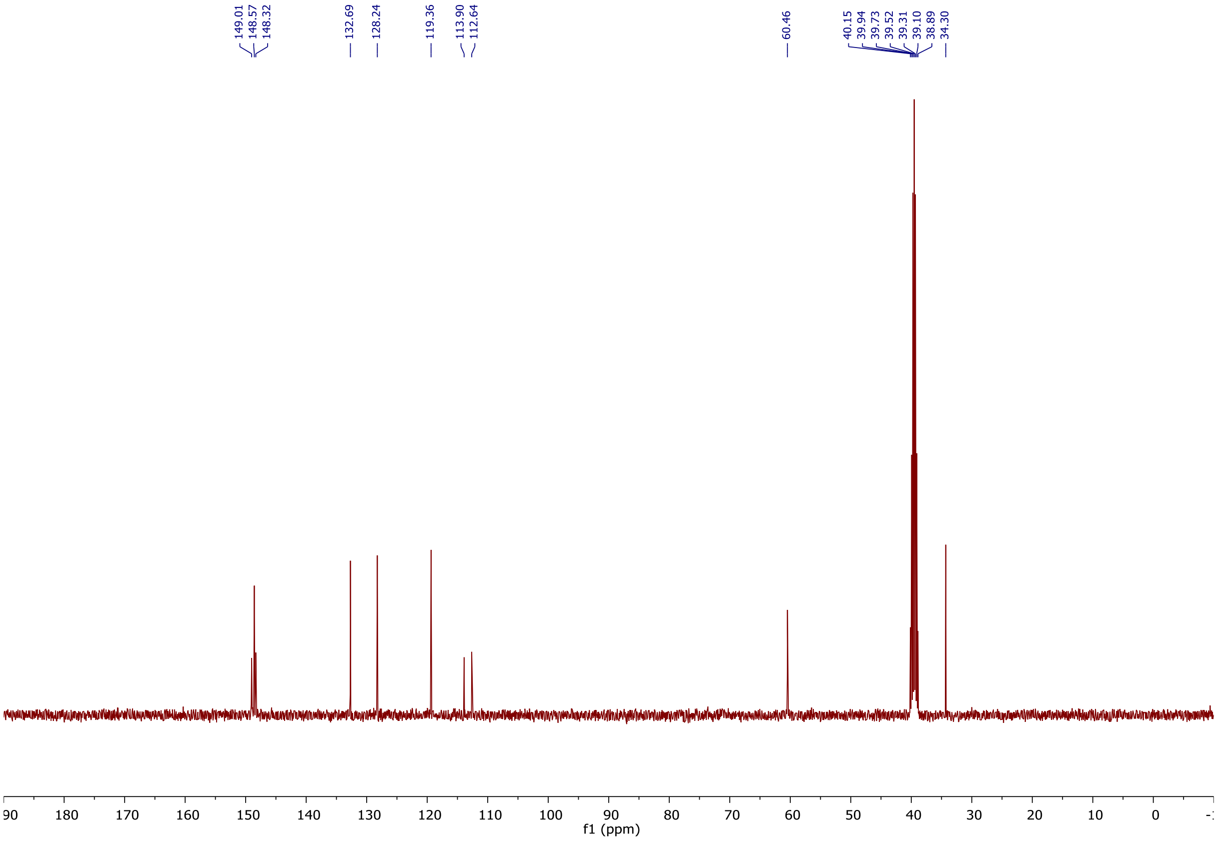

NMR spectra of *N*-benzyl-3-cyanopyridinium bromide (1o)

^{1}H NMR with DMSO-d$_6$, 400 MHz

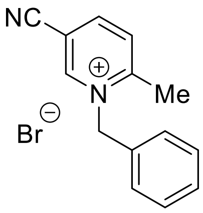

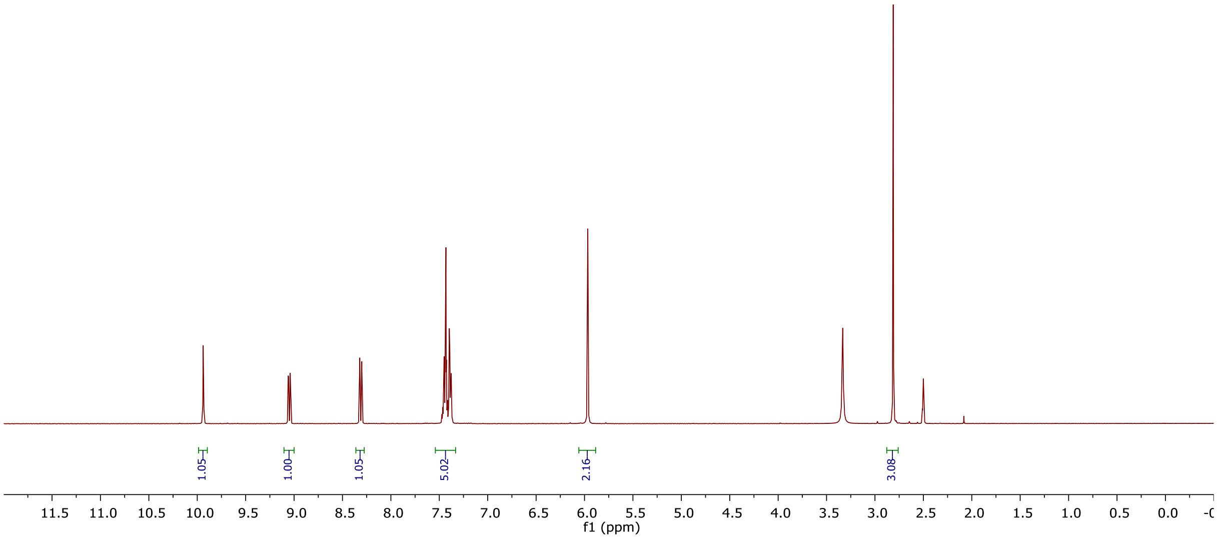

^{13}C NMR with DMSO-d$_6$, 101 MHz

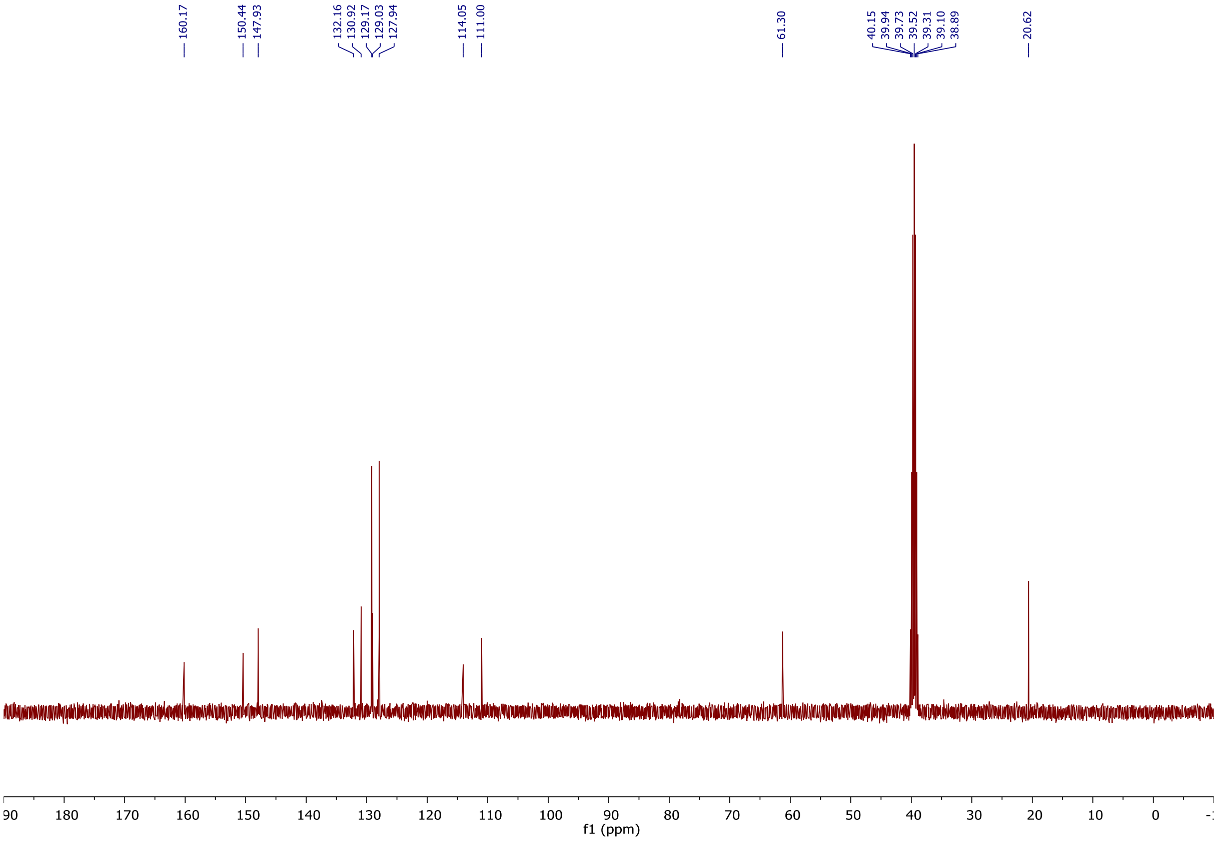

NMR spectra of *N*-benzyl-3-(methoxycarbonyl)pyridinium bromide (1p)

^{1}H NMR with DMSO-d_6, 400 MHz

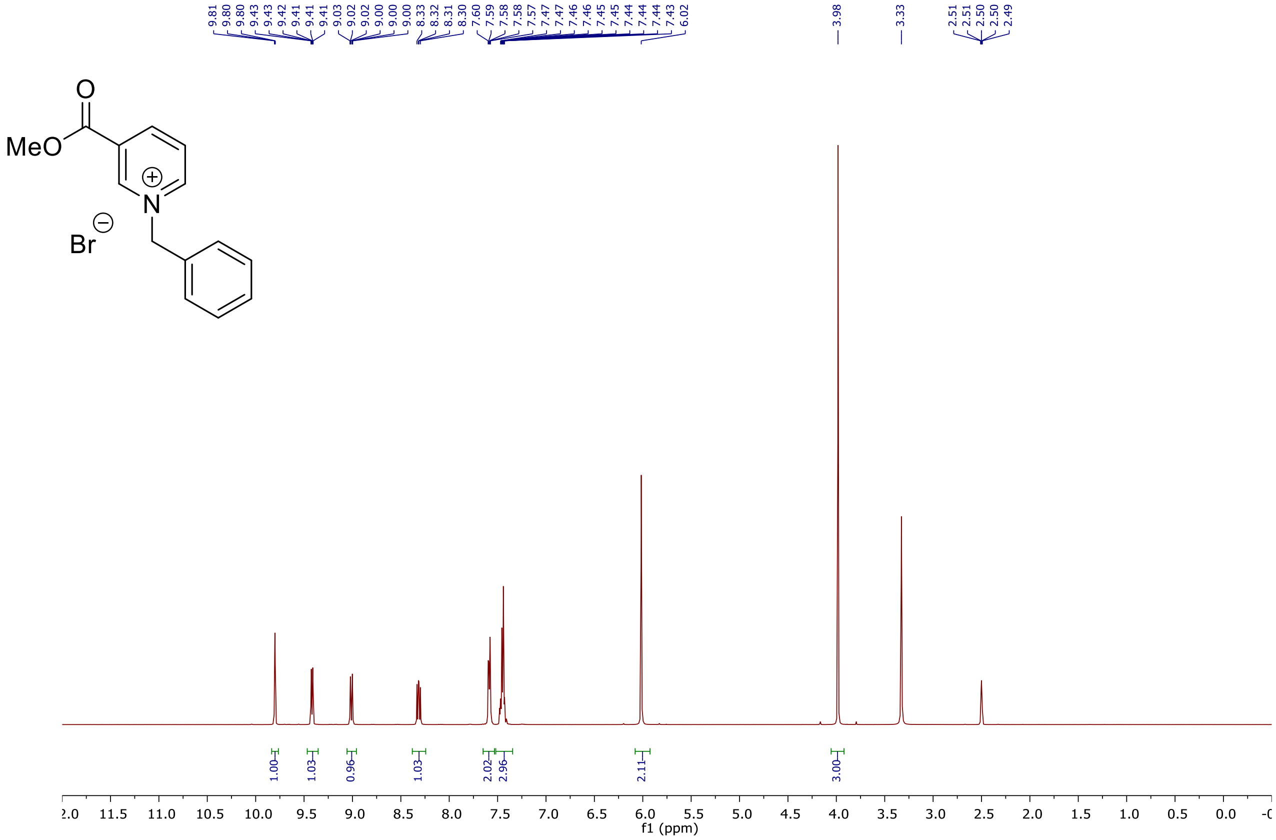

^{13}C NMR with DMSO-d_6, 101 MHz

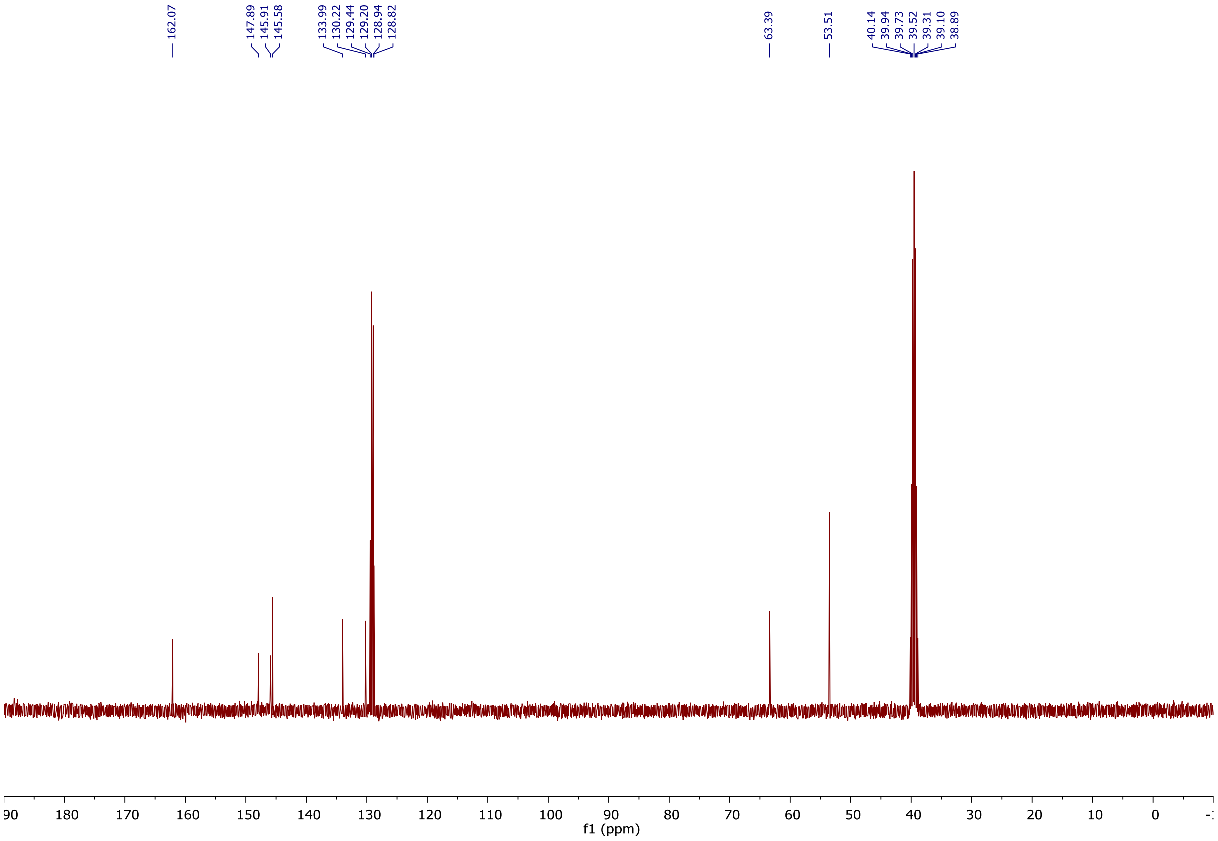

NMR spectra of *N*-benzyl-3-cyanoquinolinium bromide (1q)

^{1}H NMR with DMSO-d$_6$, 400 MHz

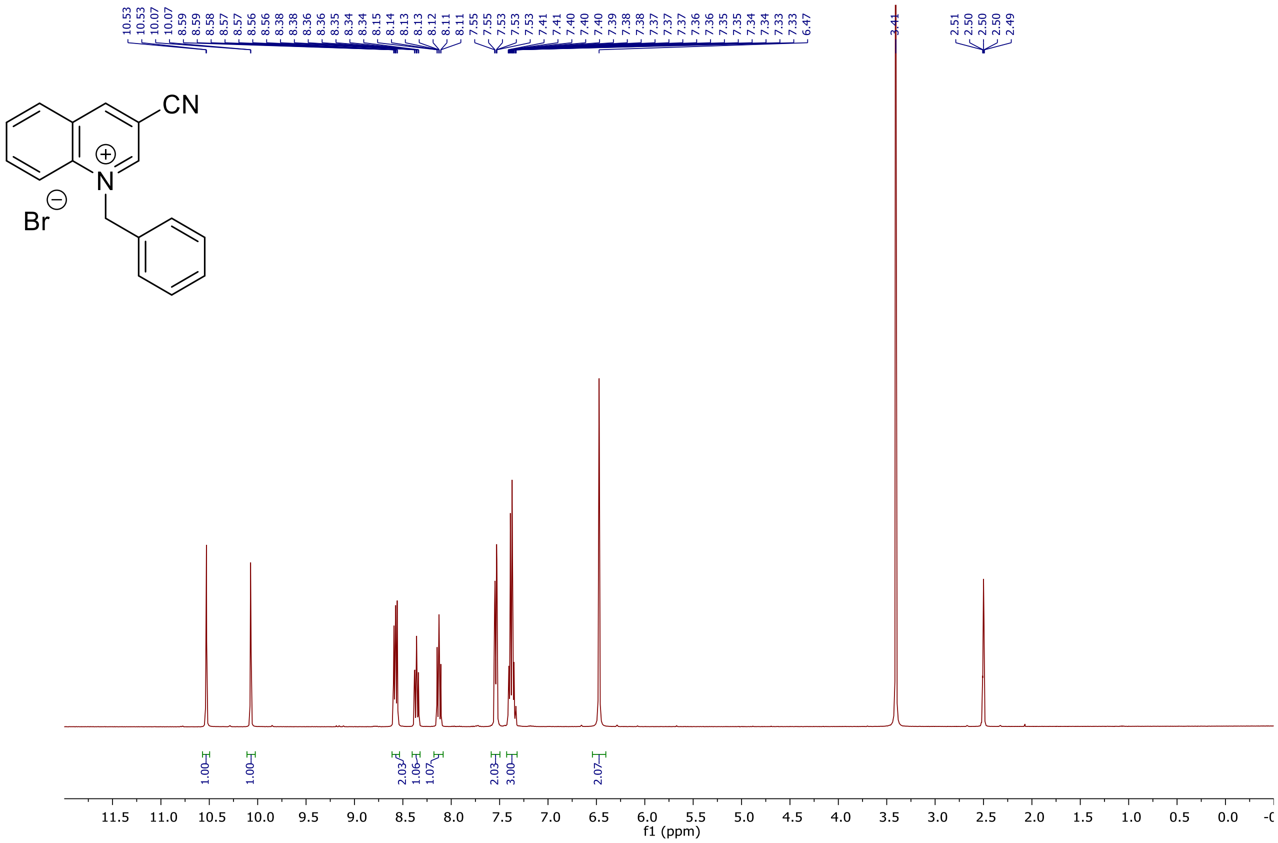

^{13}C NMR with DMSO-d$_6$, 101 MHz

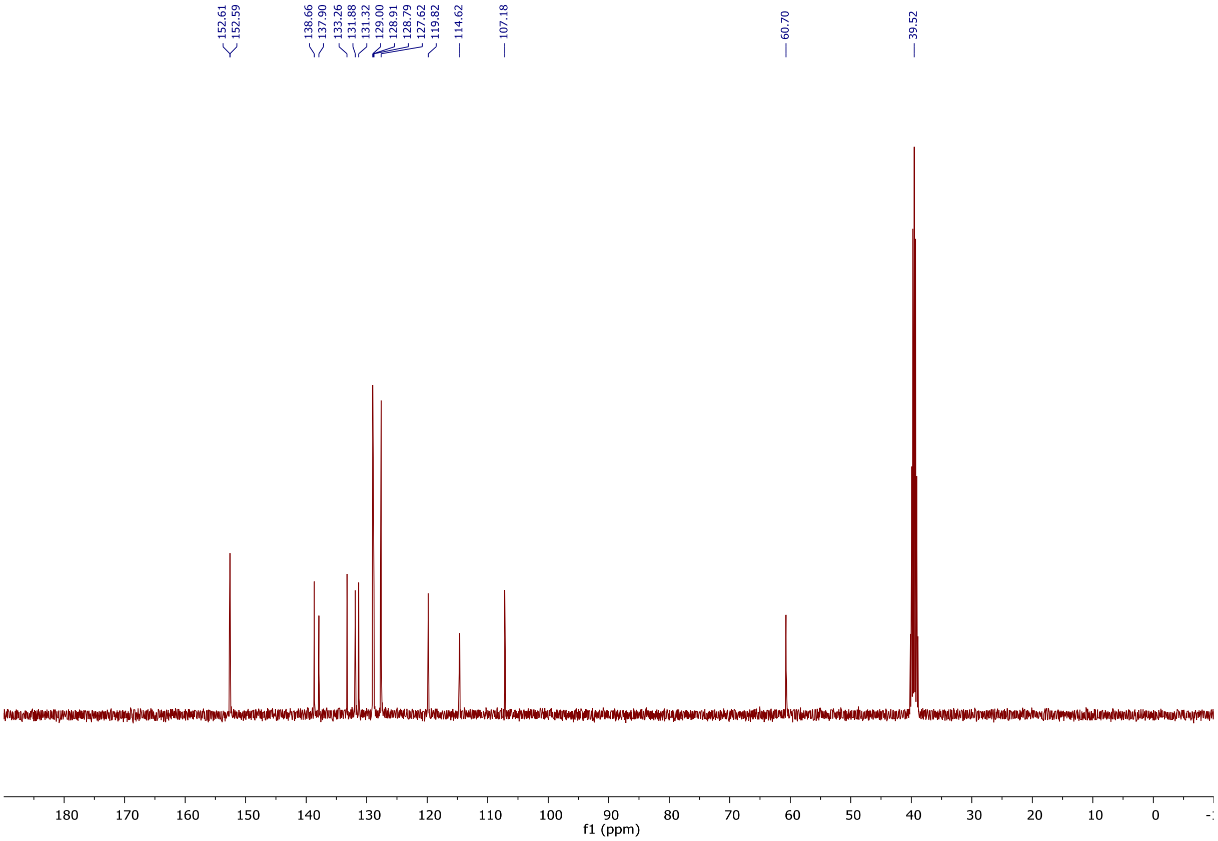

NMR spectra of *N*-(4-cyanobenzyl)-3-cyanopyridinium bromide (1r)

^{1}H NMR with DMSO-d$_6$, 400 MHz

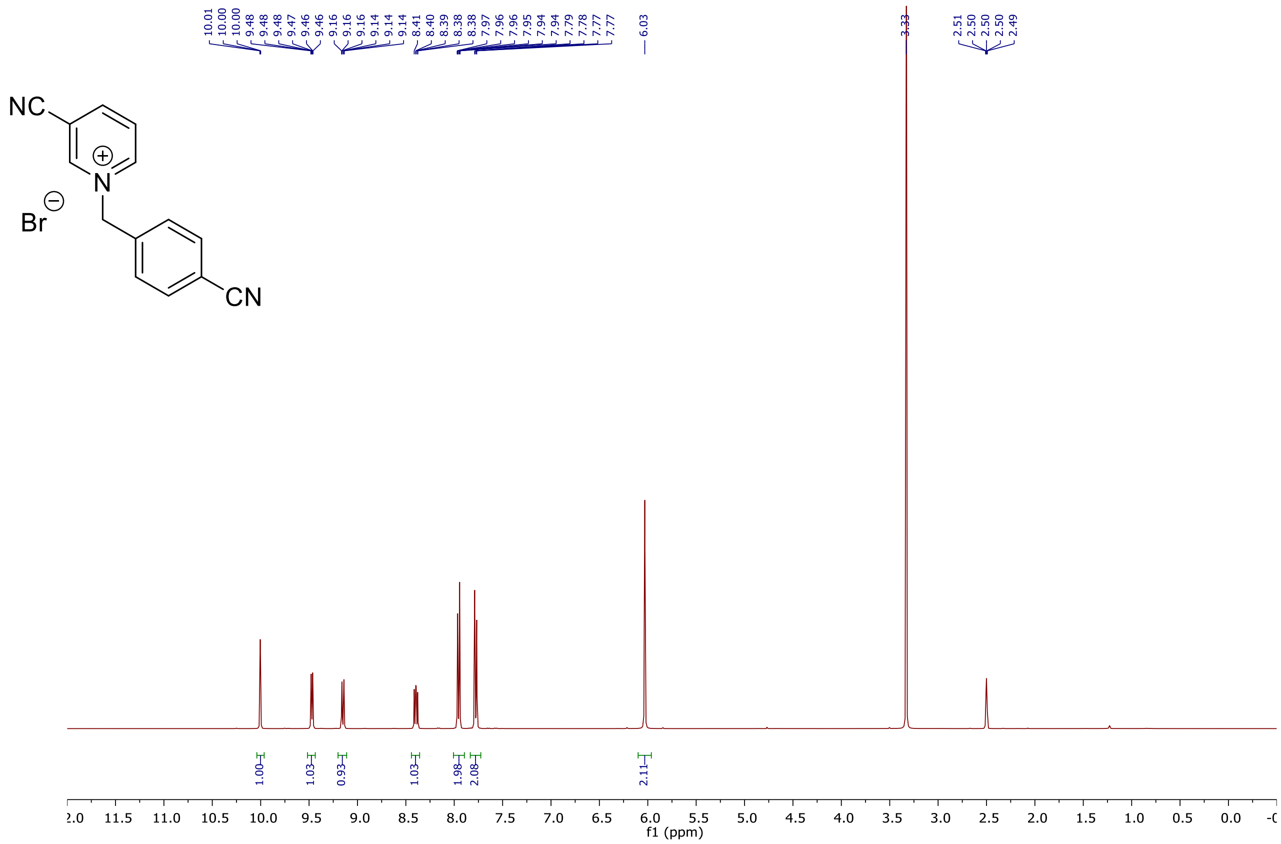

^{13}C NMR with DMSO-d$_6$, 101 MHz

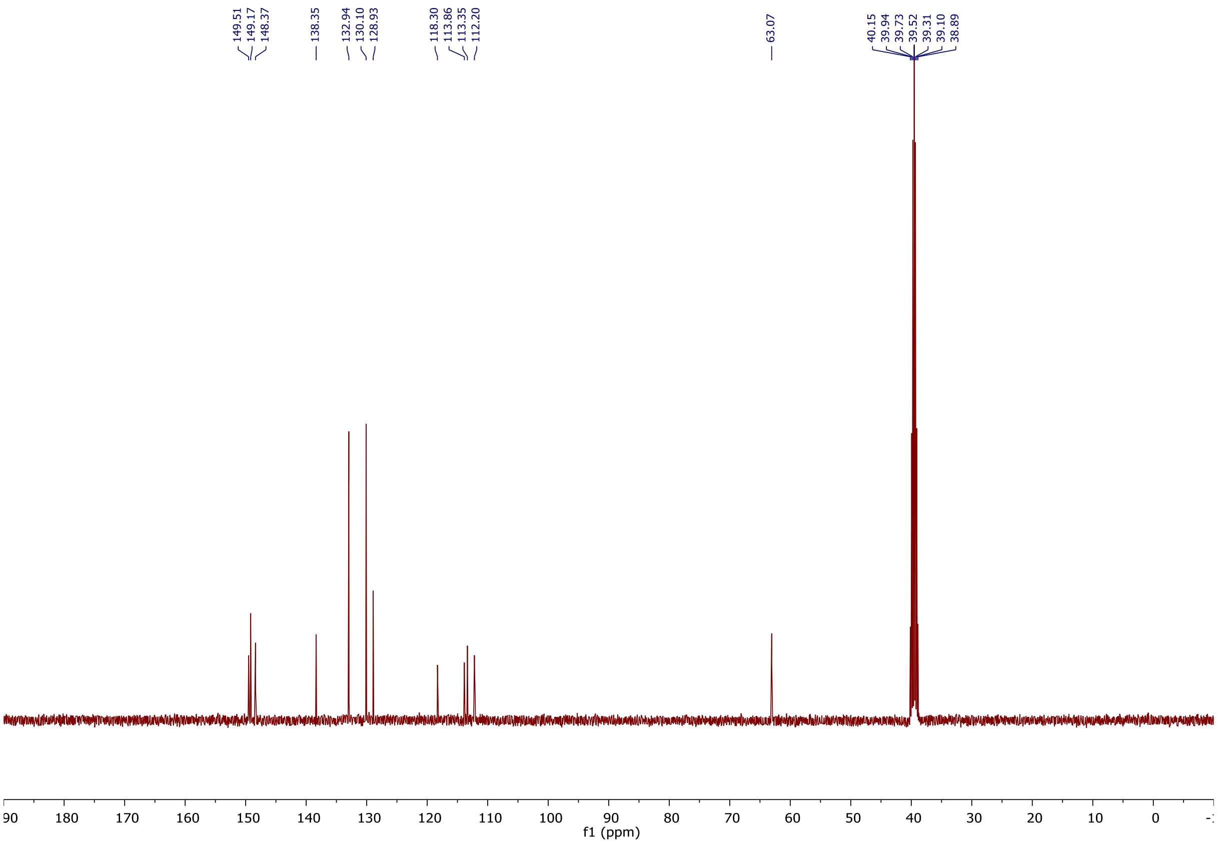

NMR spectra of *N*-(4-nitrobenzyl)-3-cyanopyridinium bromide (1s)

^{1}H NMR with DMSO-d$_6$, 400 MHz

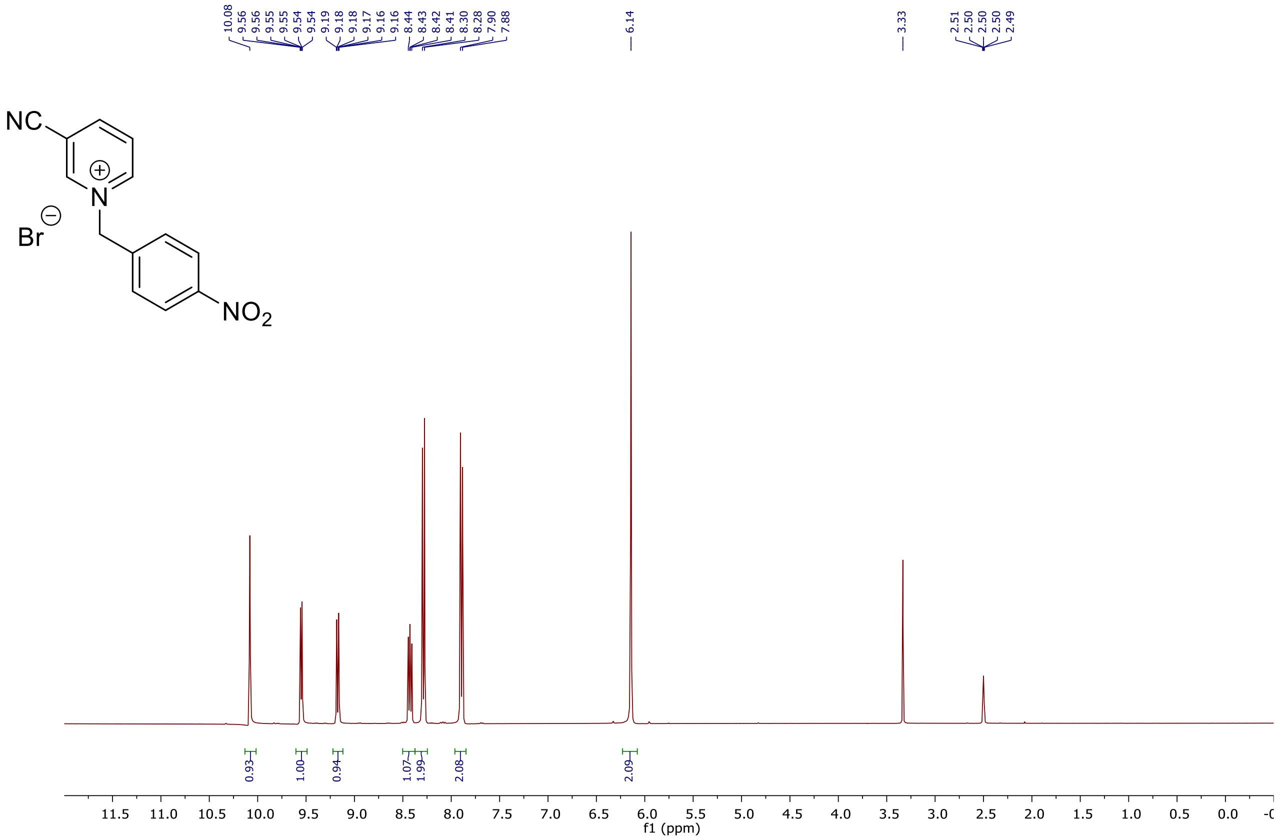

^{13}C NMR with DMSO-d$_6$, 101 MHz

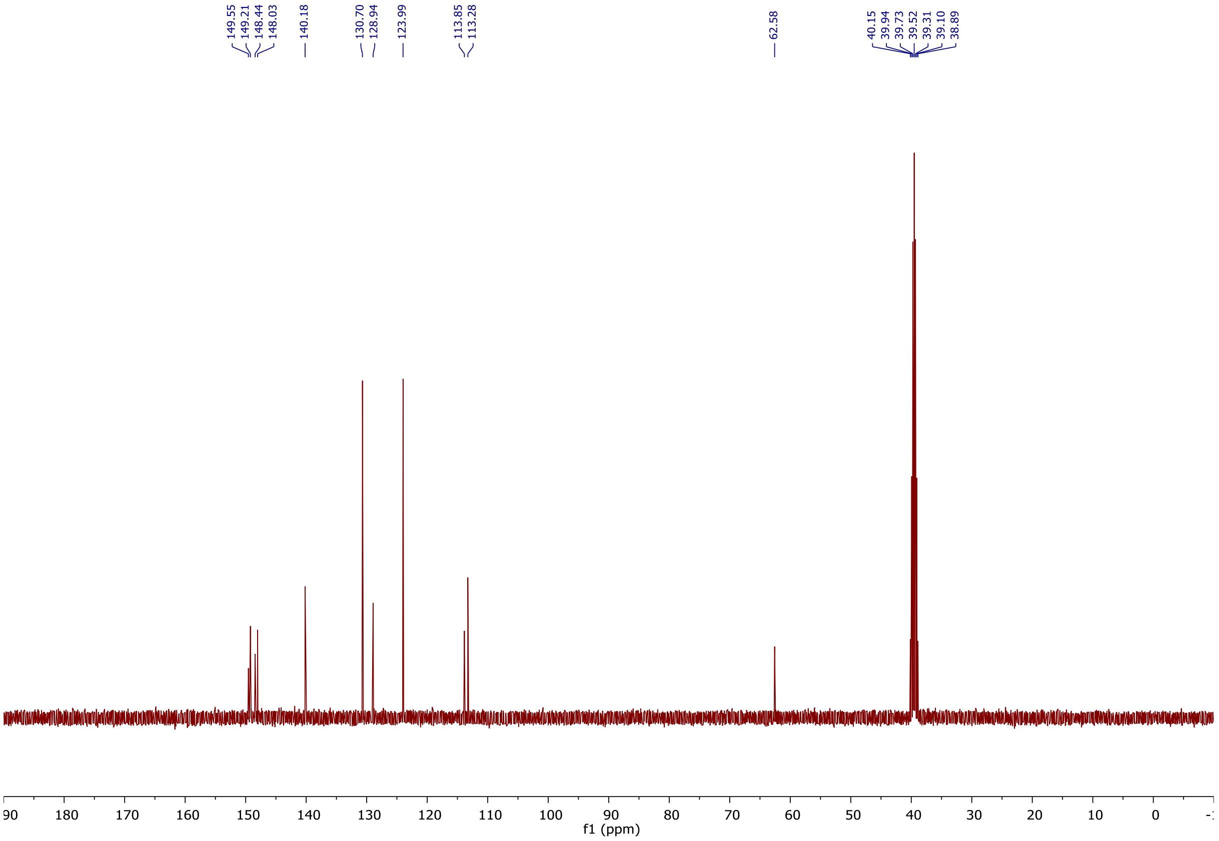

NMR spectra of *N*-allyl-3-cyanopyridinium bromide (1t)

^{1}H NMR with DMSO-d$_6$, 400 MHz

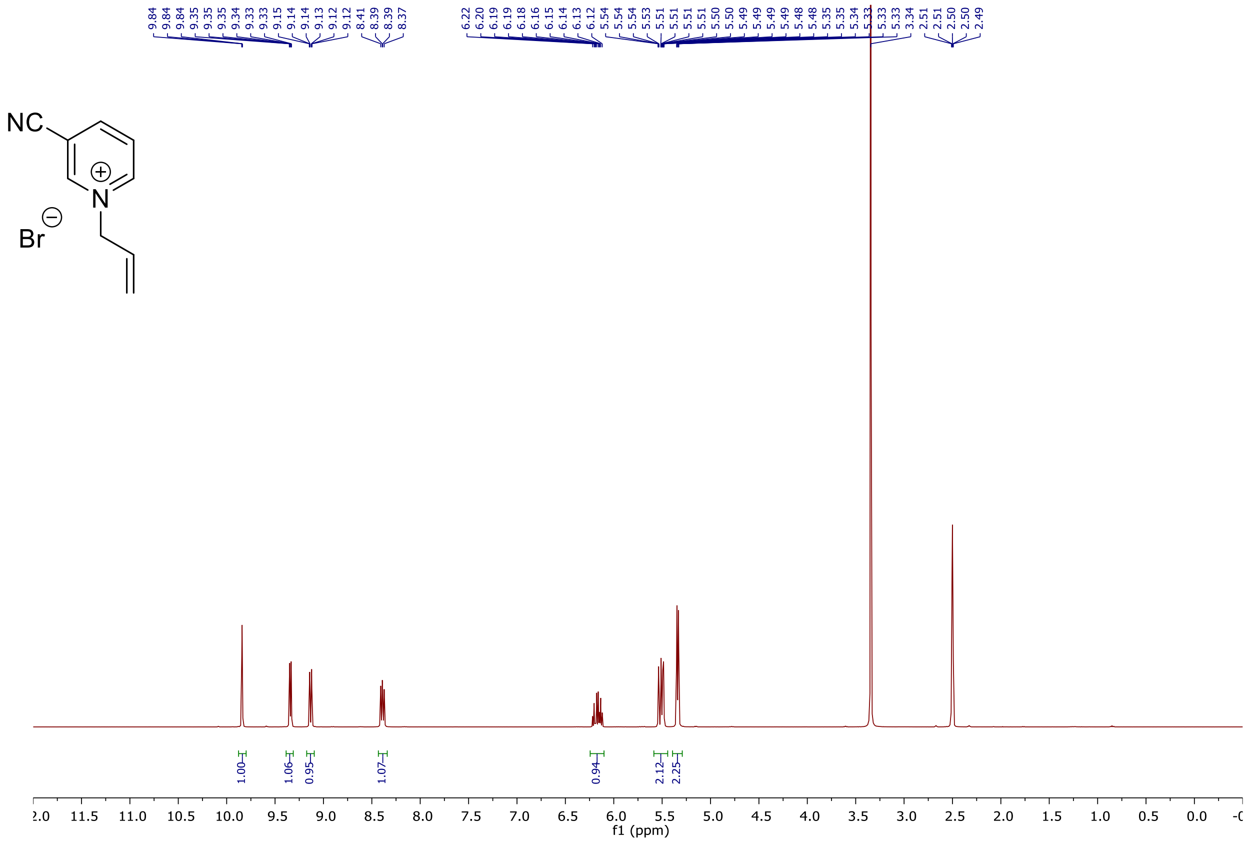

^{13}C NMR with DMSO-d$_6$, 101 MHz

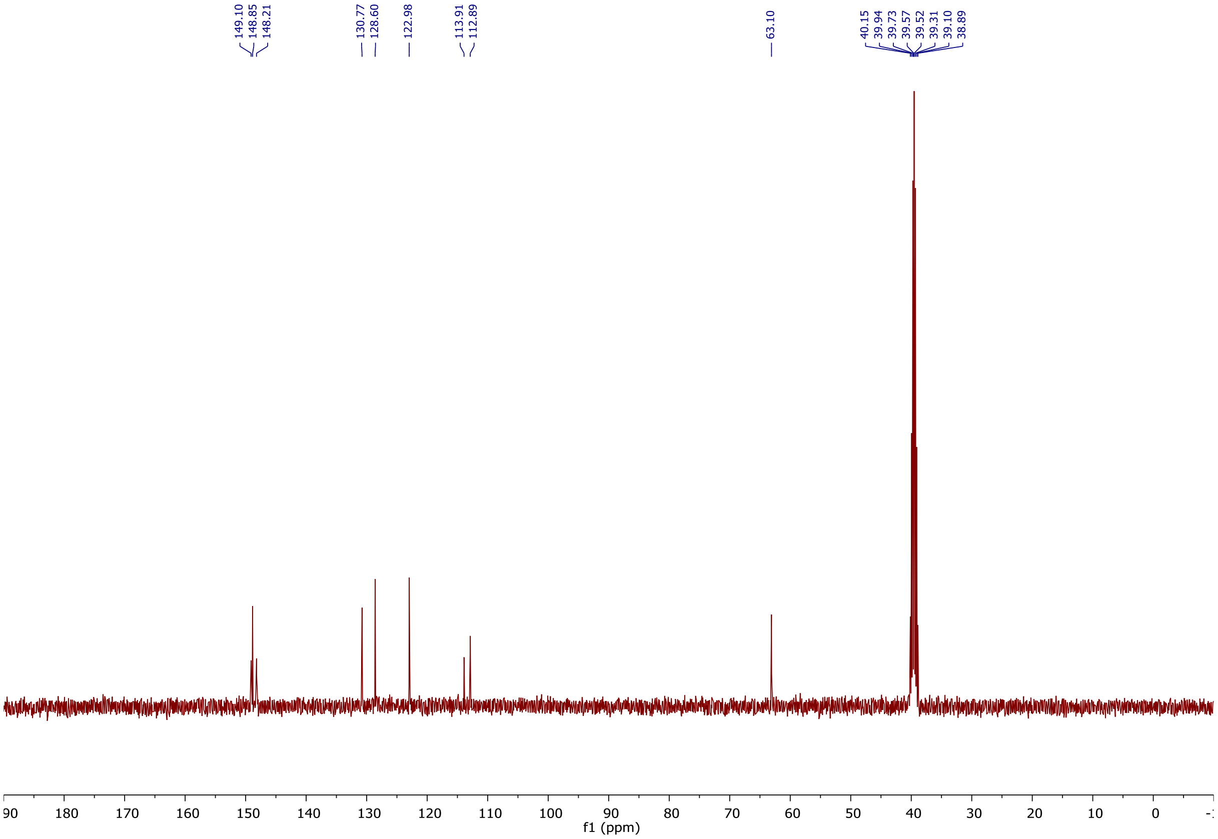

NMR spectra of *(R)*-1-benzyl-4-ethyl-1,4-dihydropyridine-3-carbonitrile (3a)

^{1}H NMR with $CDCl_3$, 400 MHz

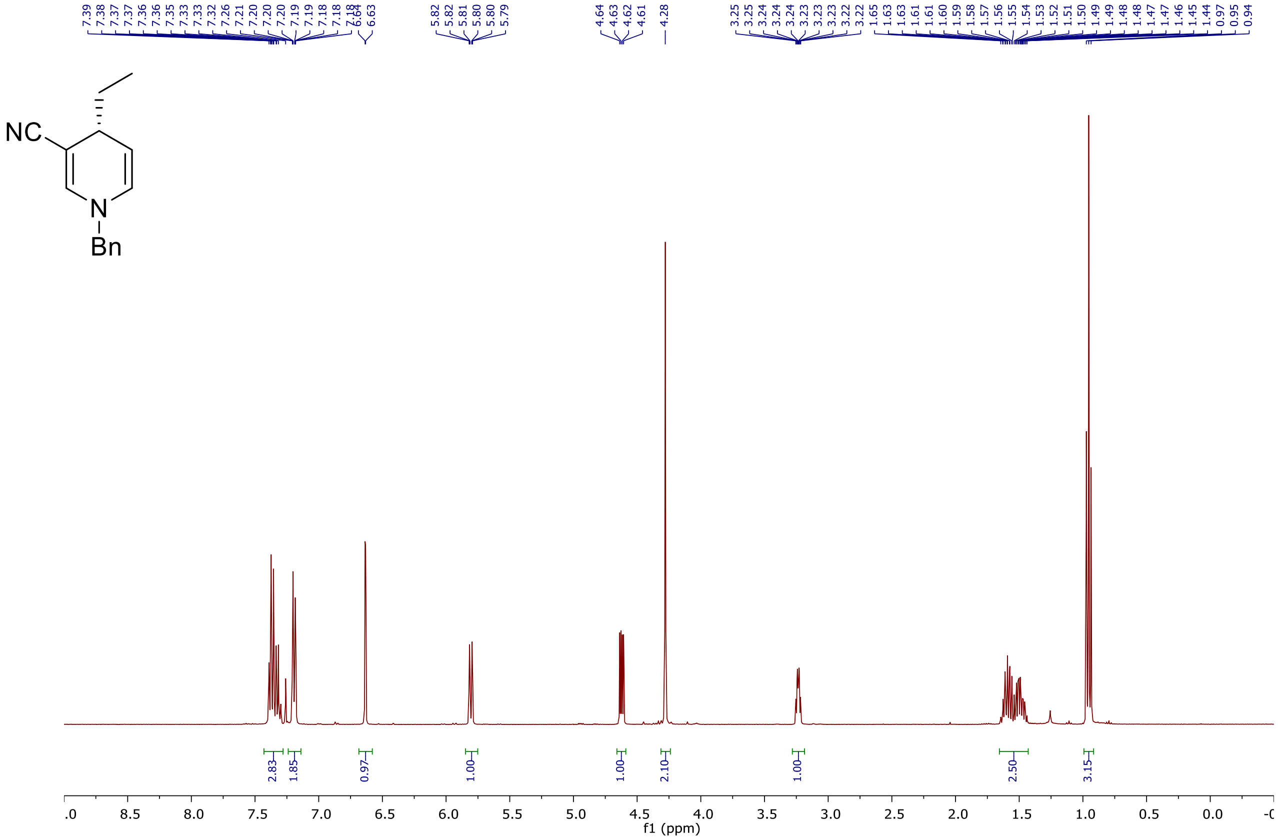

^{13}C NMR with $CDCl_3$, 101 MHz

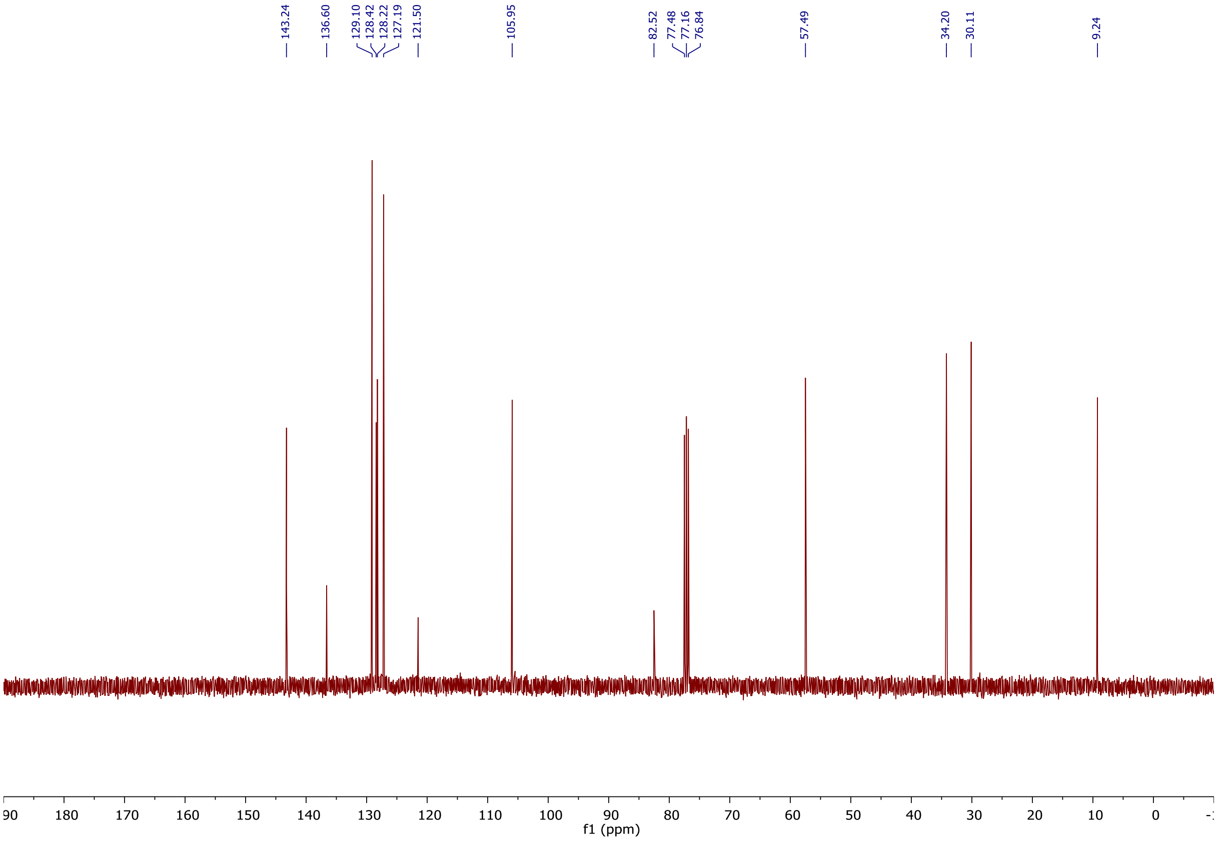

NMR spectra of (*S*)-*N*-benzyl-6-ethyl-1,6-dihydropyridine-3-carbonitrile (3a')

^{1}H NMR with $CDCl_3$, 400 MHz

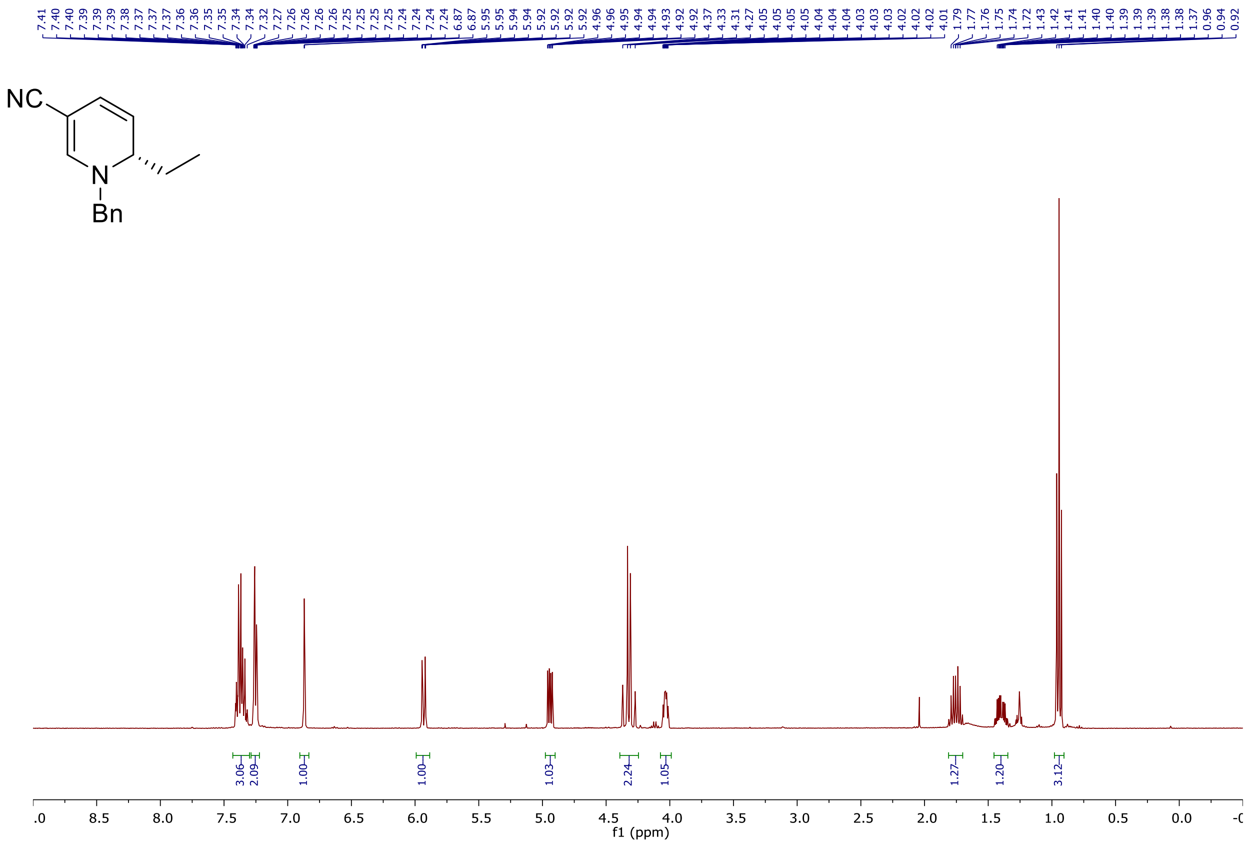

^{13}C NMR with $CDCl_3$, 101 MHz

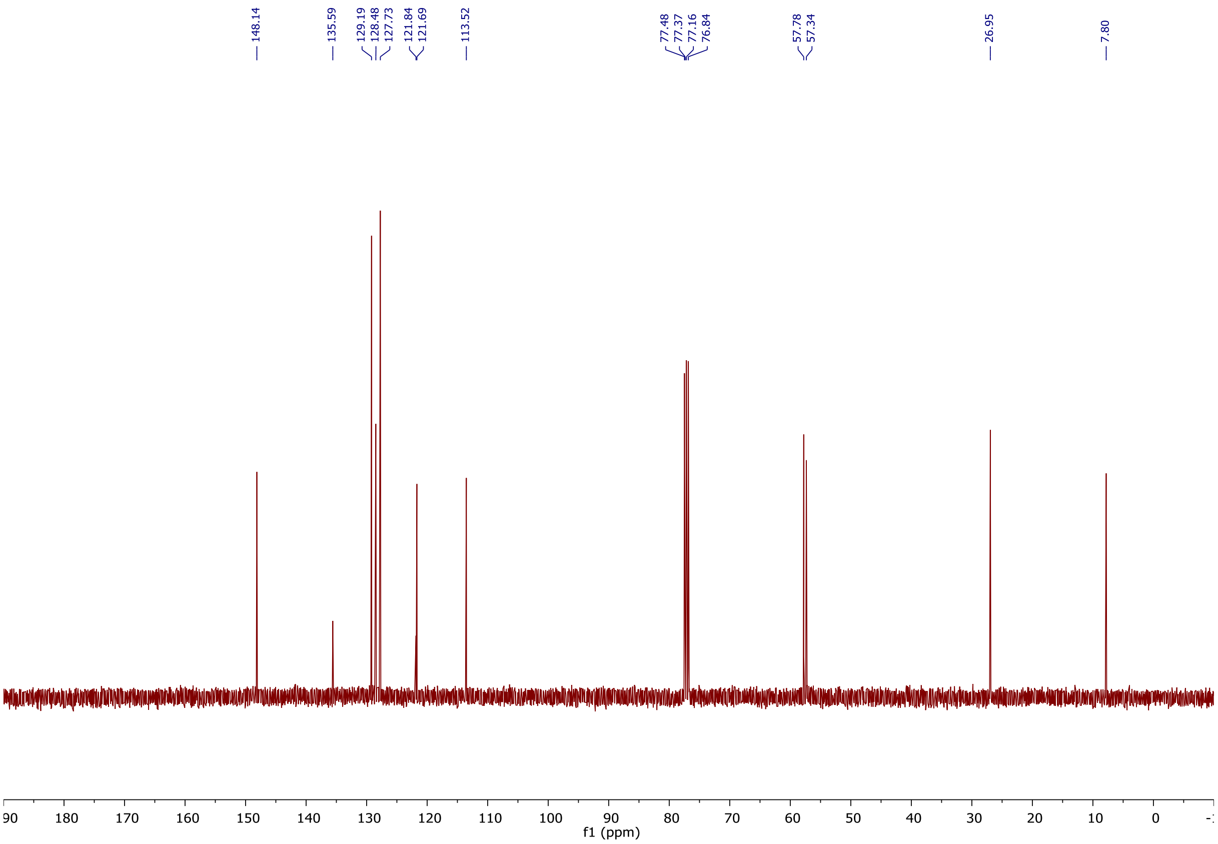

NMR spectra of (*R*)-4-ethyl-1-(4-methylbenzyl)-1,4-dihydropyridine-3-carbonitrile (3b)

^{1}H NMR with $CDCl_3$, 400 MHz

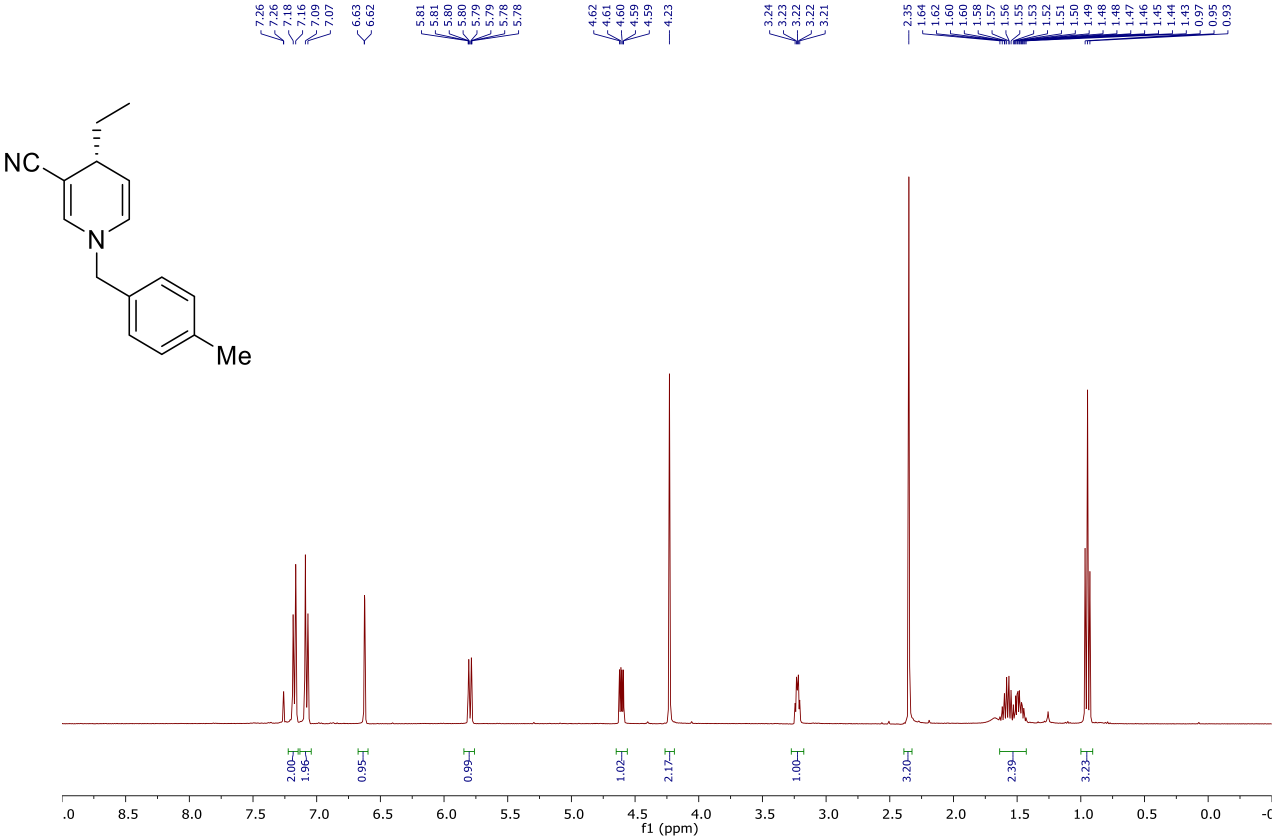

^{13}C NMR with $CDCl_3$, 101 MHz

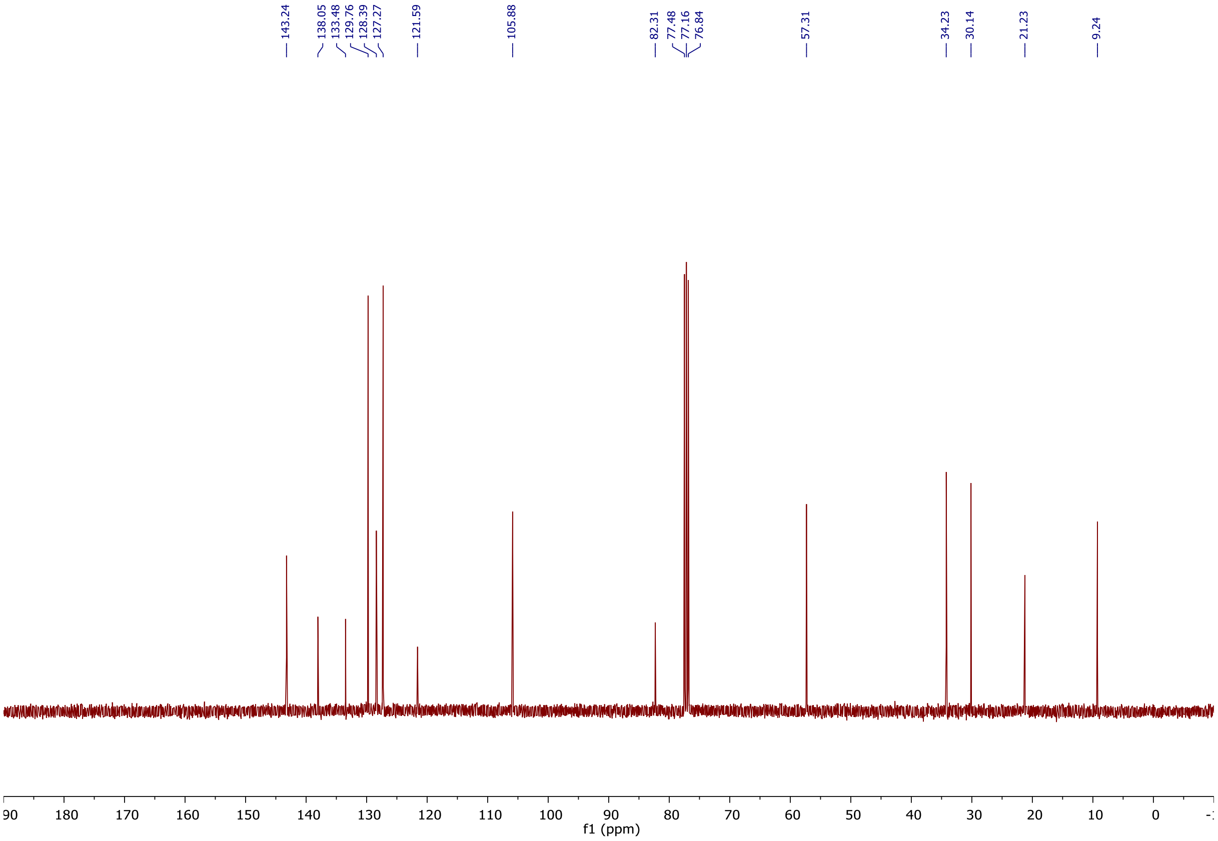

NMR spectra of (*R*)-1-(4-(*tert*-butyl)benzyl)-4-ethyl-1,4-dihydropyridine-3-carbonitrile (3c)

^{1}H NMR with $CDCl_3$, 400 MHz

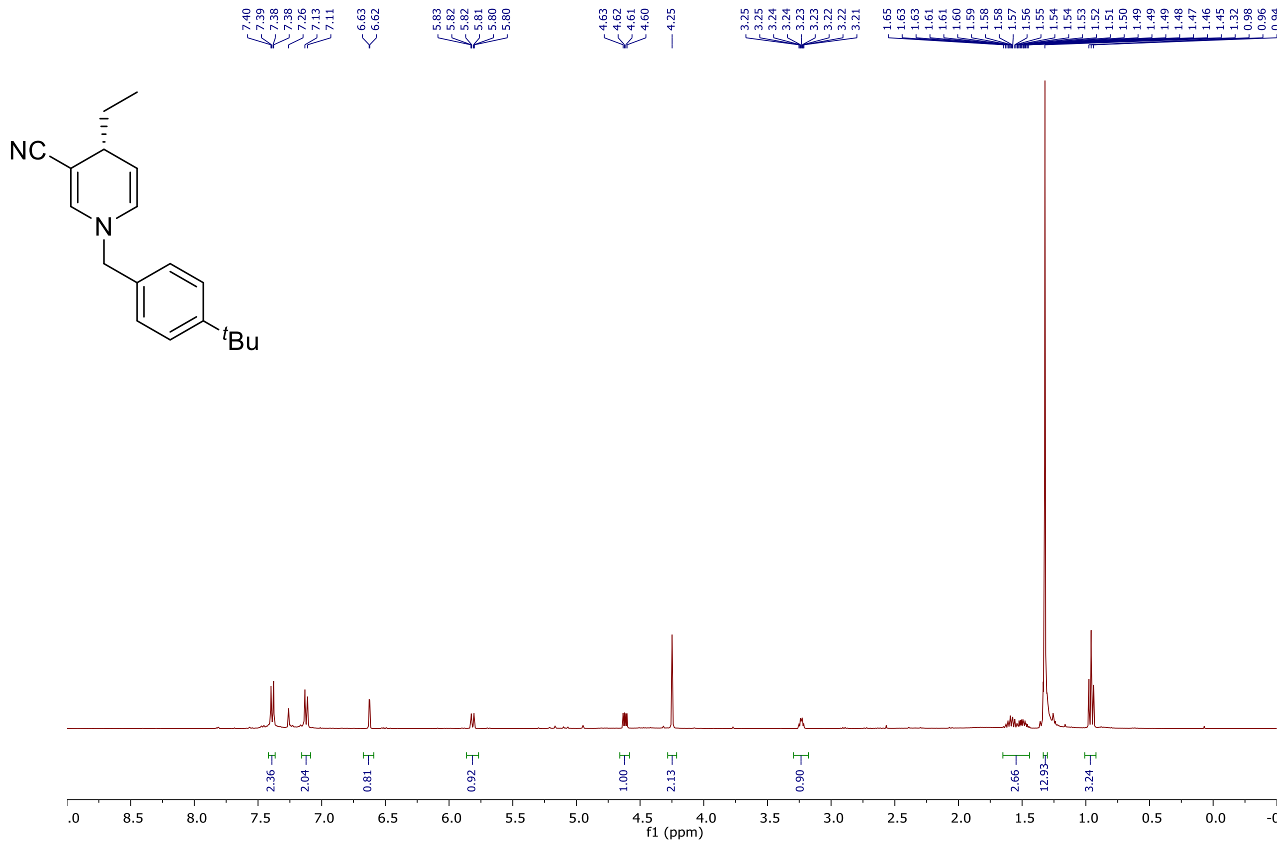

^{13}C NMR with $CDCl_3$, 101 MHz

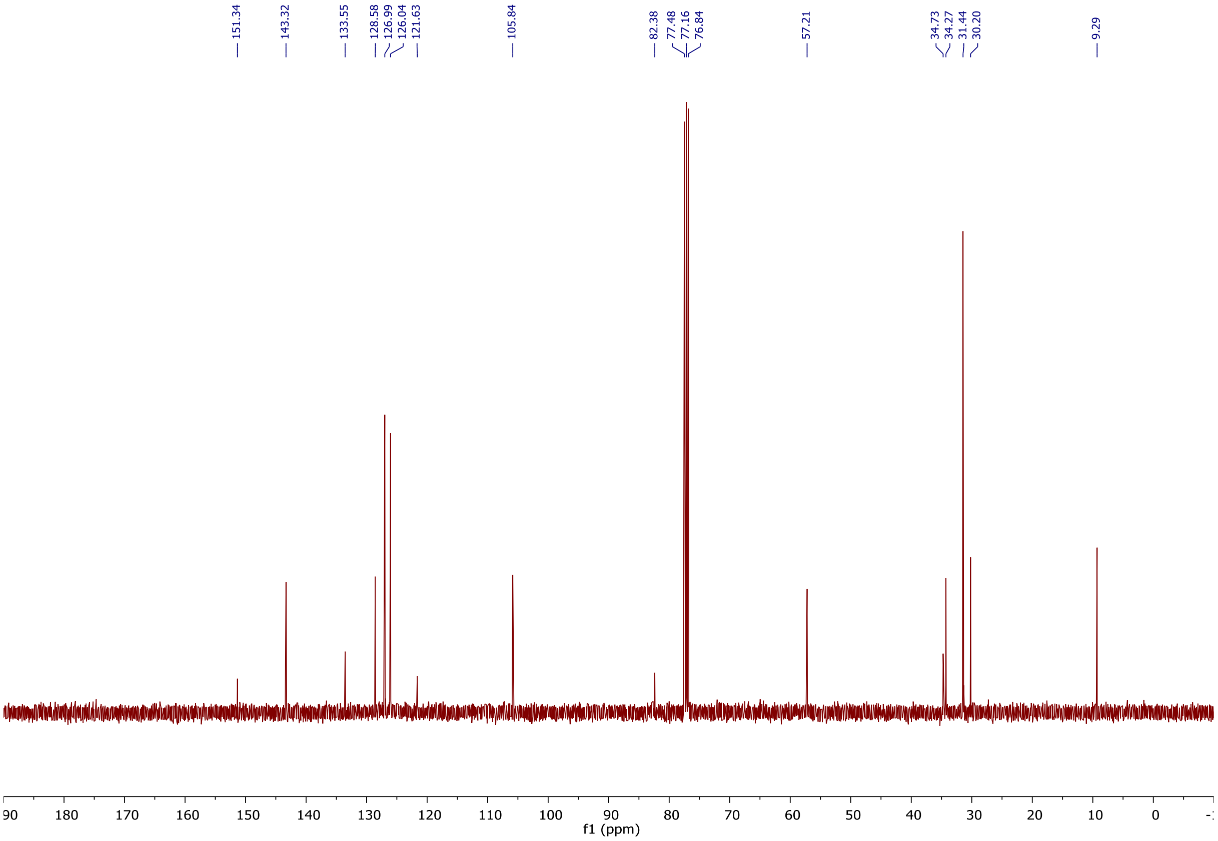

NMR spectra of (*R*)-4-ethyl-1-(4-(trifluoromethyl)benzyl)-1,4-dihydropyridine-3-carbonitrile (3d)

^{1}H NMR with $CDCl_3$, 400 MHz

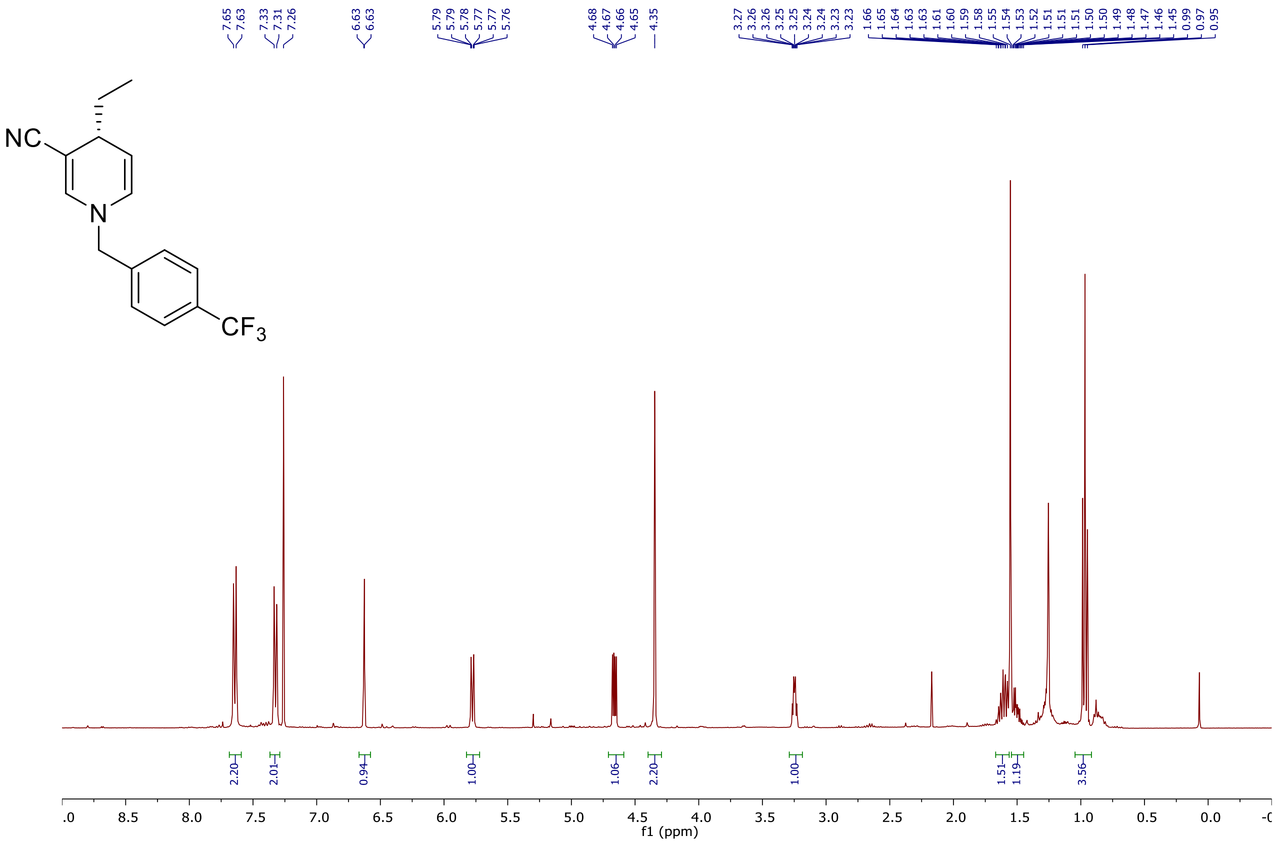

^{13}C NMR with $CDCl_3$, 101 MHz

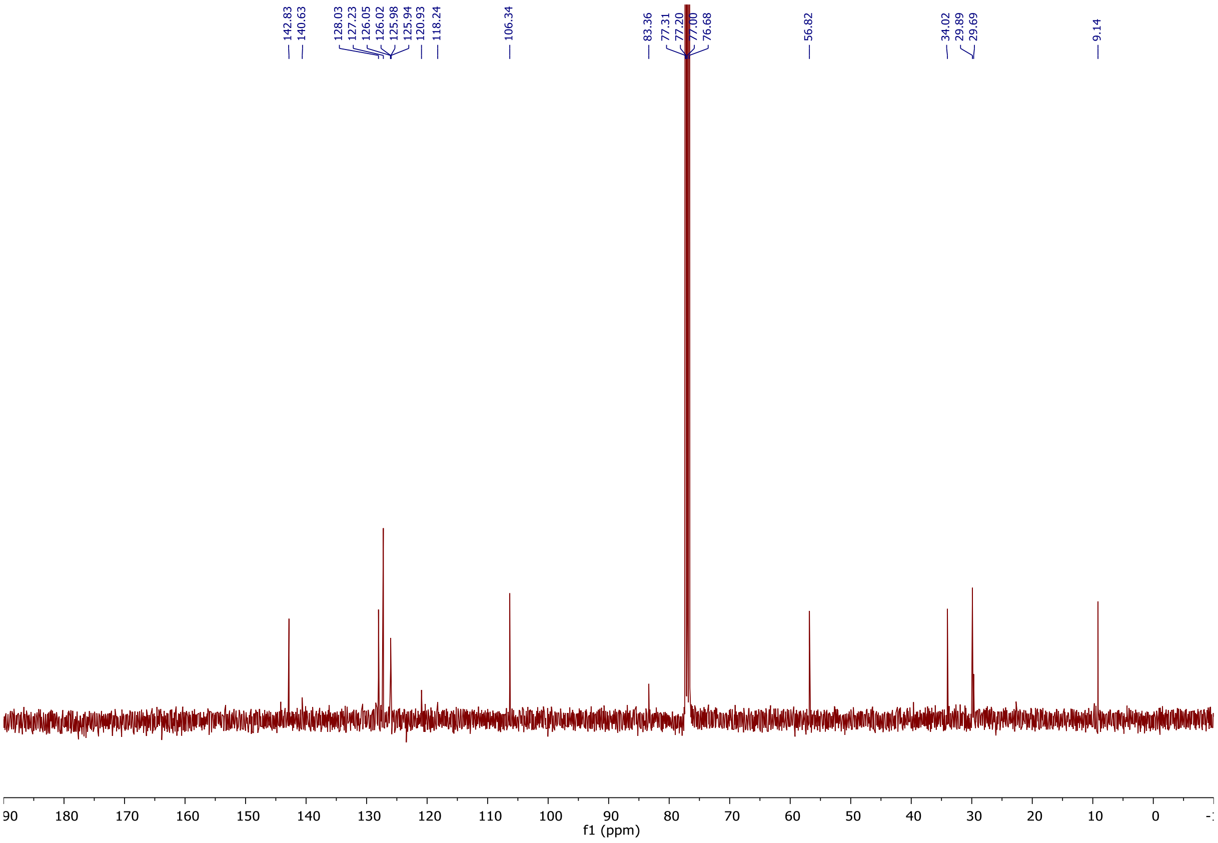

^{19}F NMR with $CDCl_3$, 376 MHz

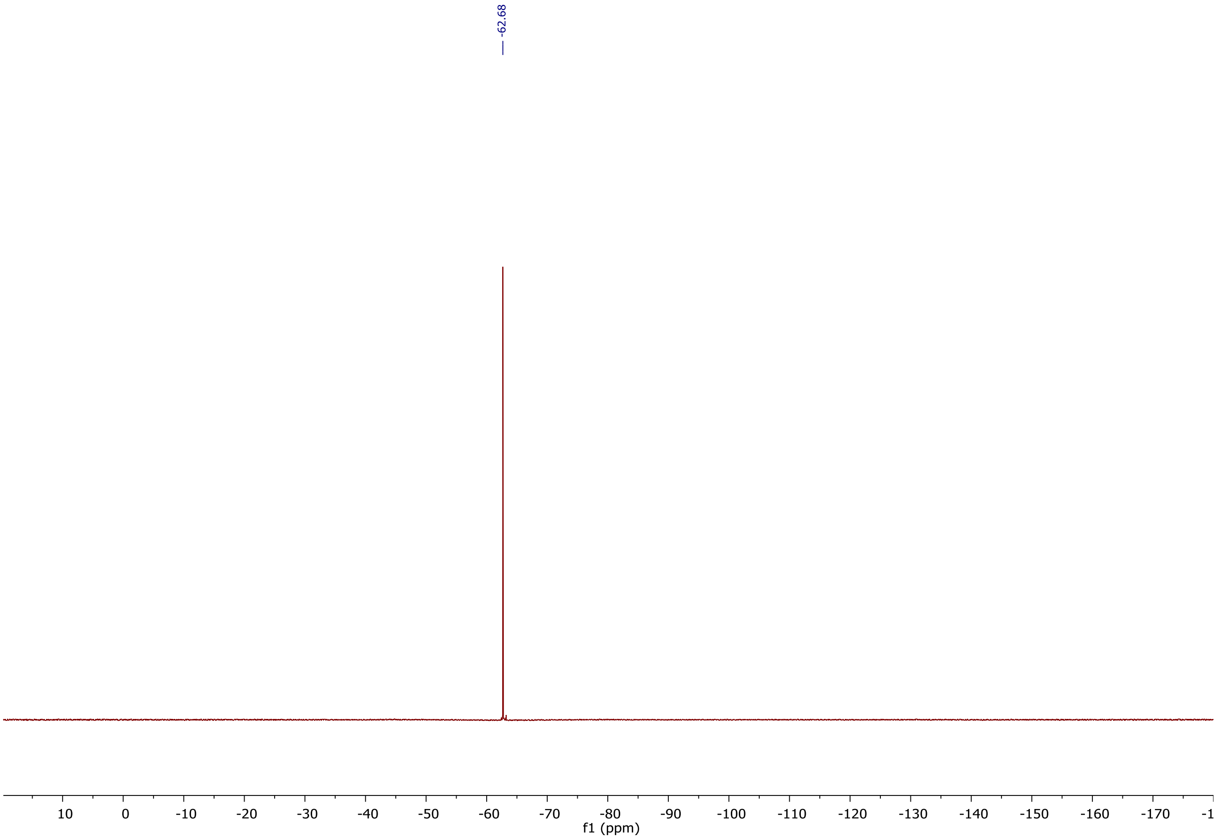

NMR spectra of (*R*)-4-ethyl-1-(4-fluorobenzyl)-1,4-dihydropyridine-3-carbonitrile (3e)

^{1}H NMR with $CDCl_3$, 400 MHz

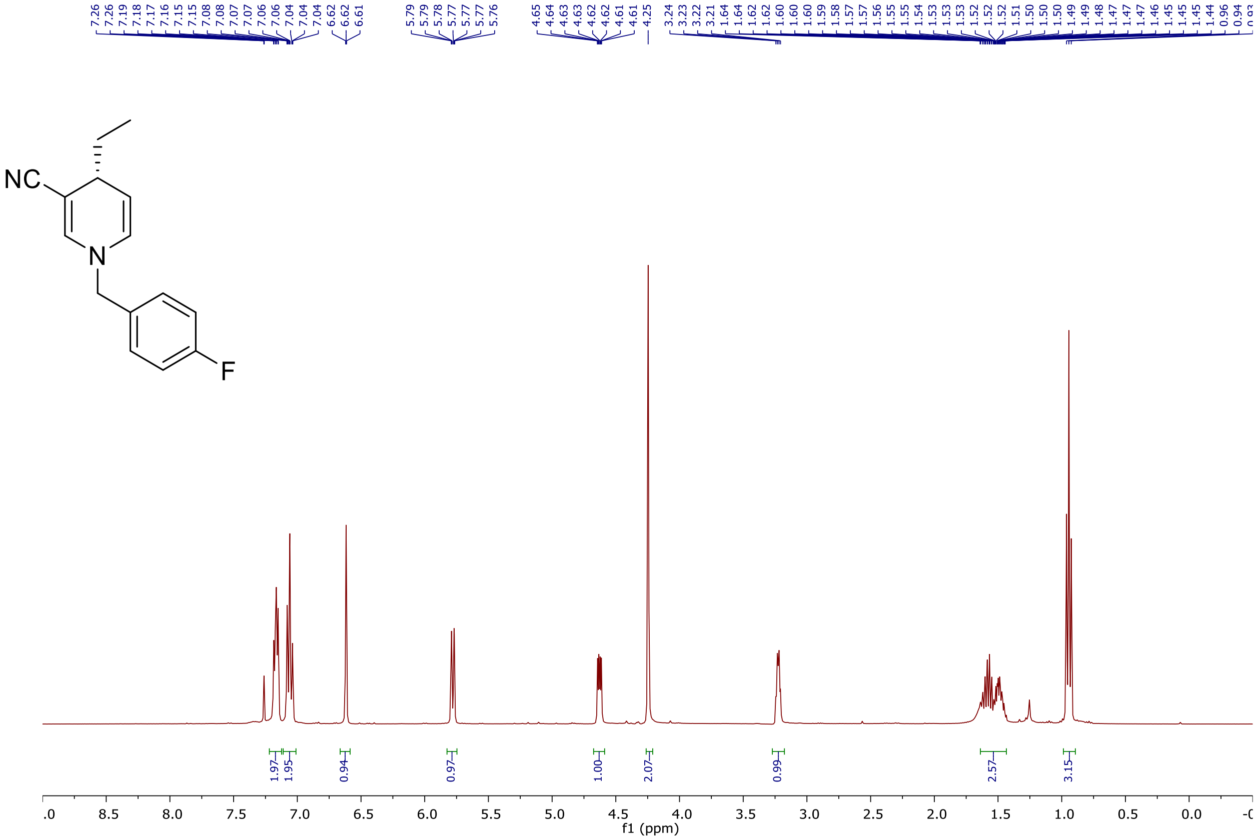

^{13}C NMR with $CDCl_3$, 101 MHz

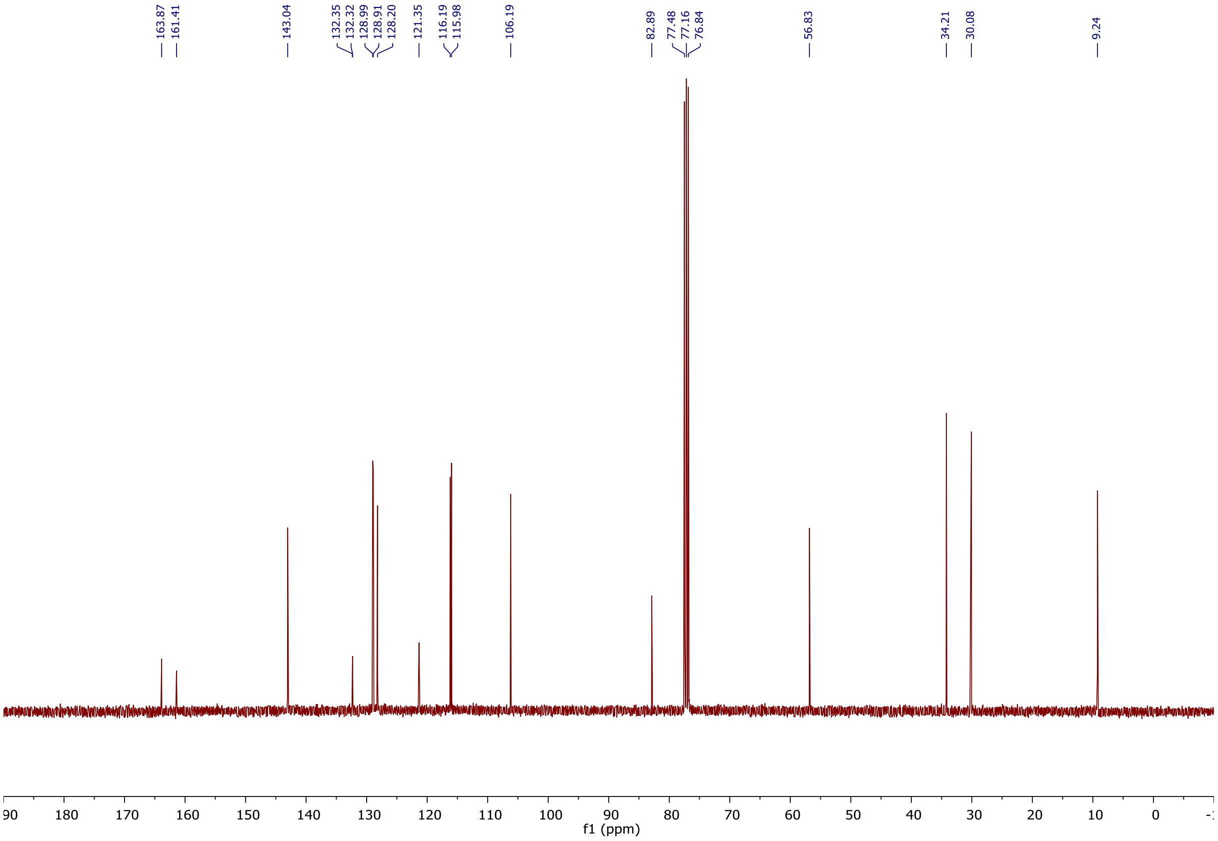

^{19}F NMR with $CDCl_3$, 376 MHz

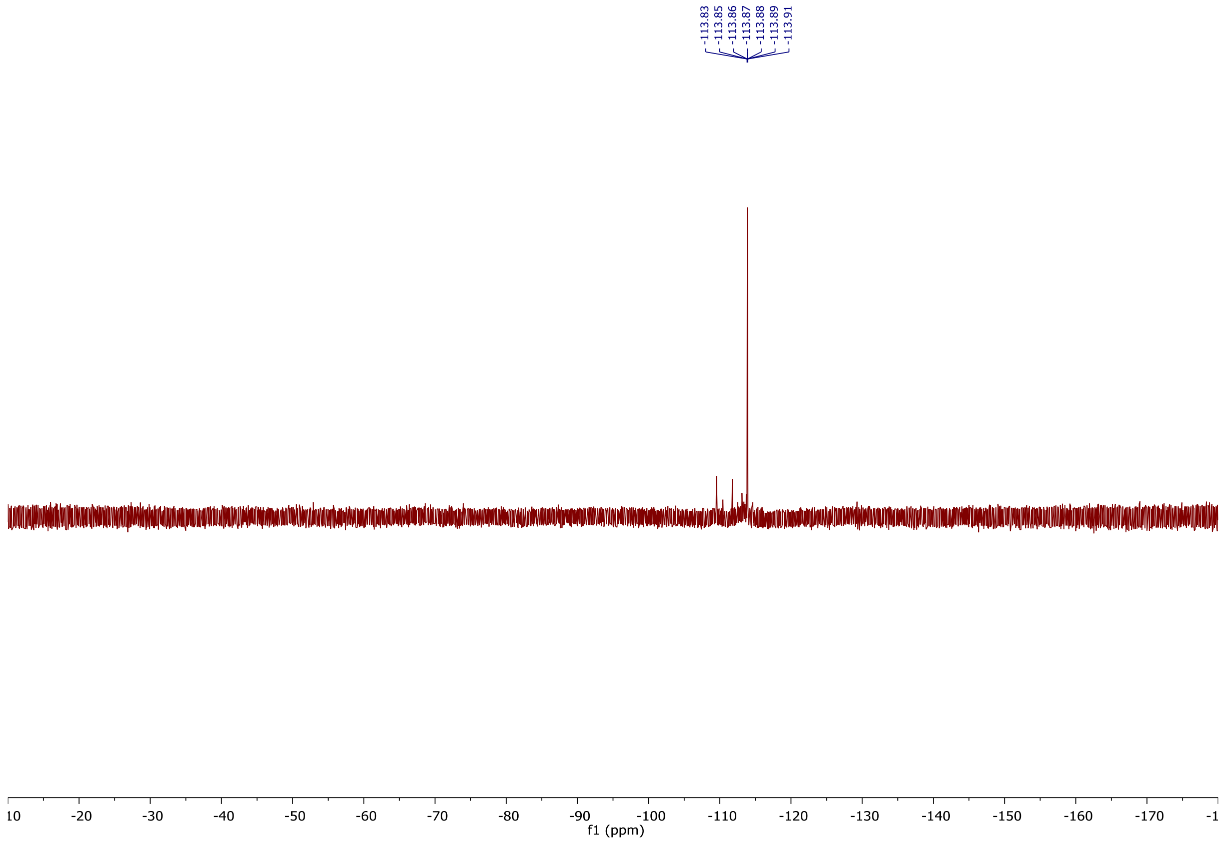

NMR spectra of (*R*)-4-ethyl-1-(naphthalen-2-ylmethyl)-1,4-dihydropyridine-3-carbonitrile (3f)

^{1}H NMR with $CDCl_3$, 400 MHz

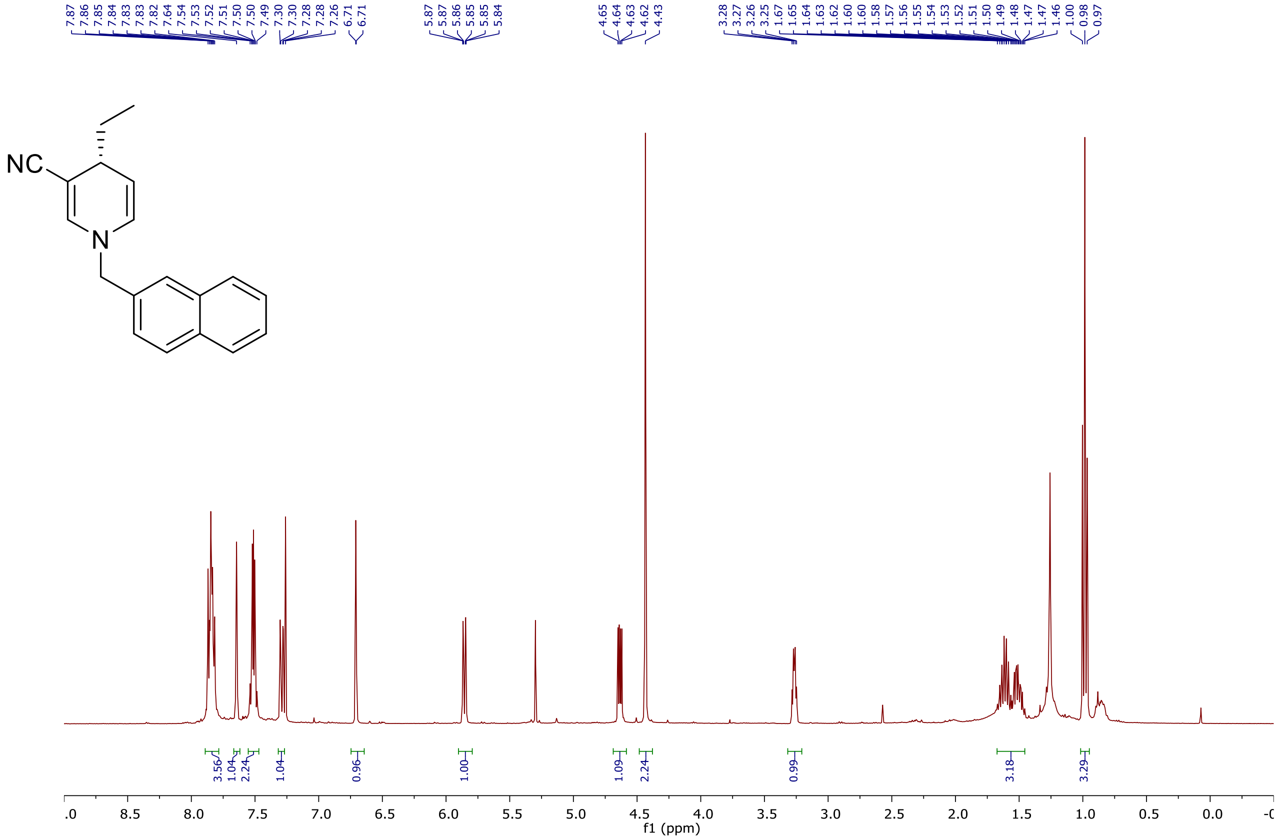

^{13}C NMR with $CDCl_3$, 101 MHz

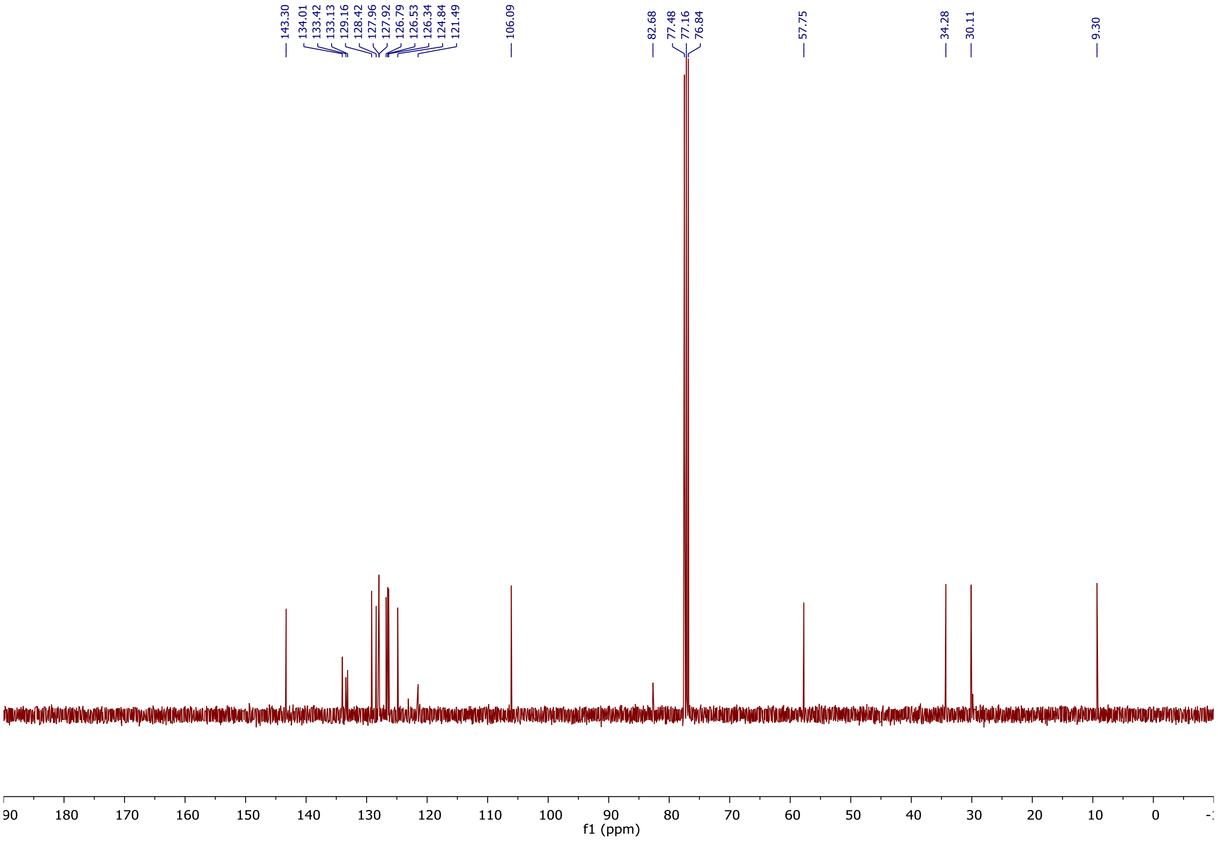

NMR spectra of (*R*)-4-ethyl-1-(3-methylbenzyl)-1,4-dihydropyridine-3-carbonitrile (3g)

^{1}H NMR with $CDCl_3$, 400 MHz

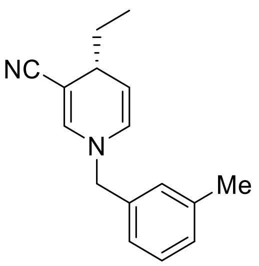

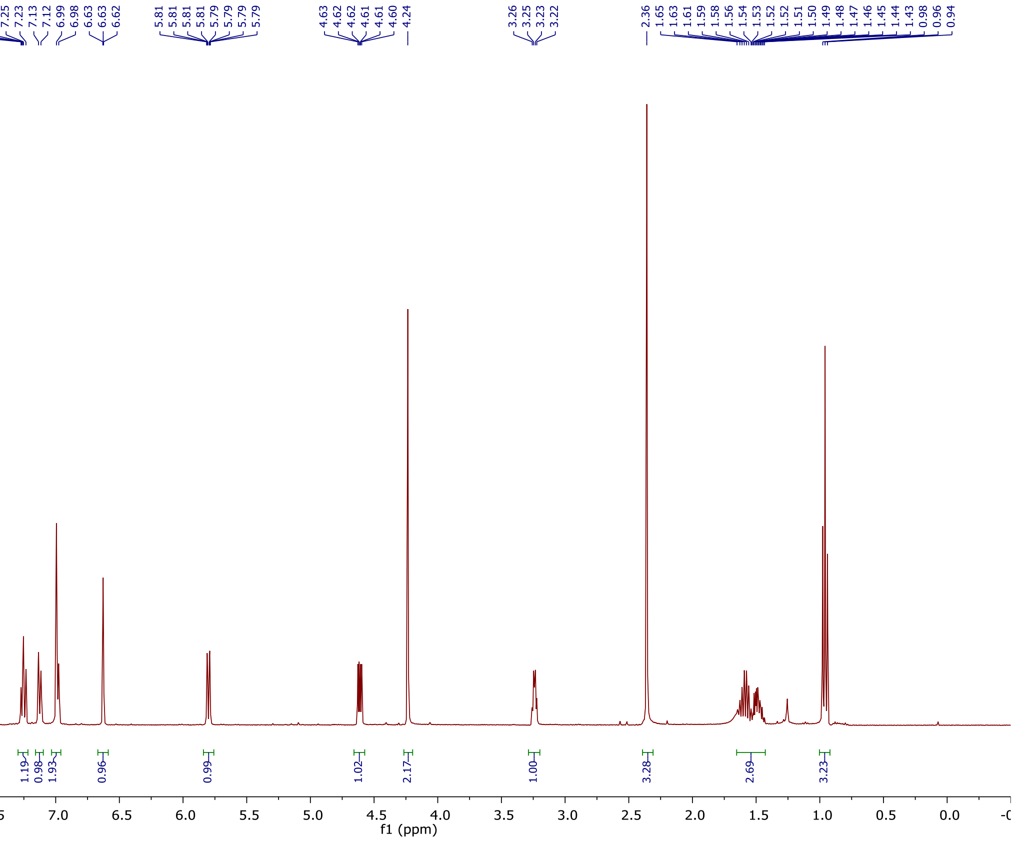

^{13}C NMR with $CDCl_3$, 101 MHz

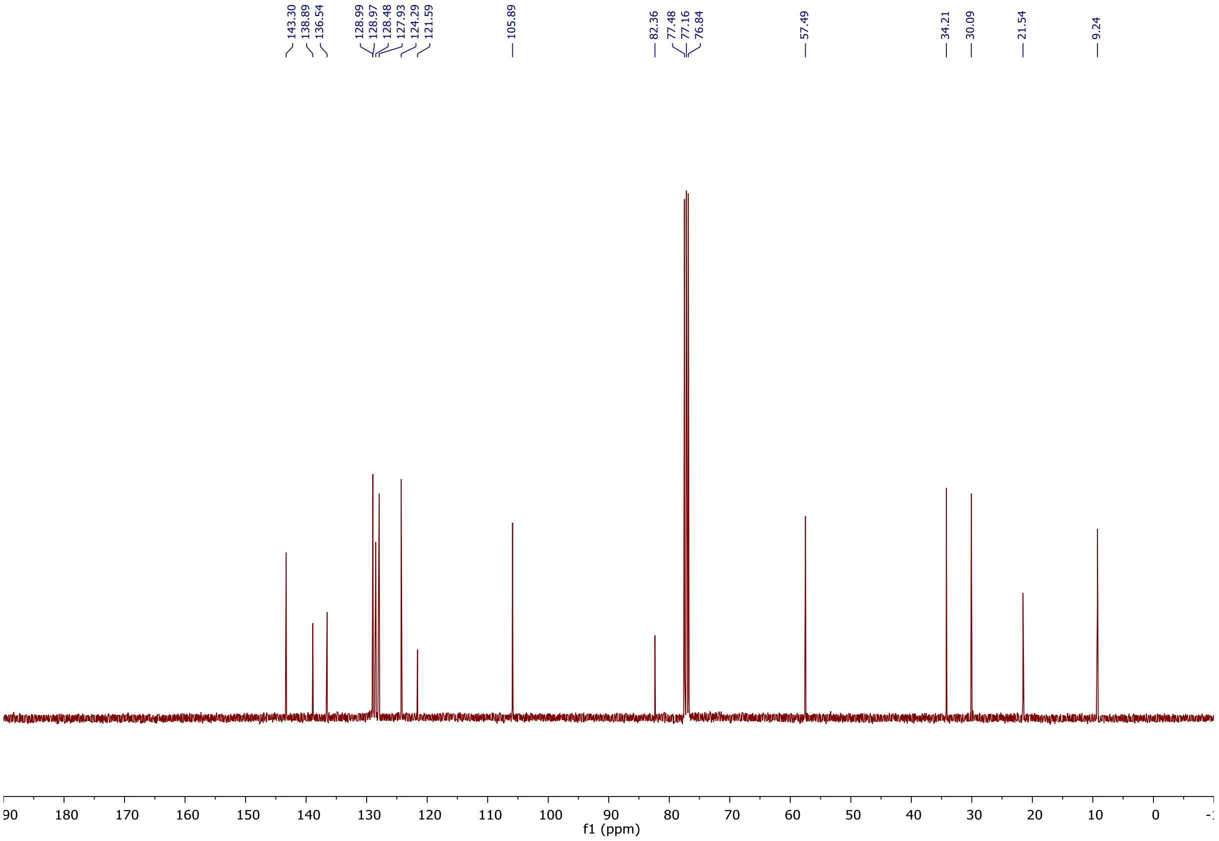

NMR spectra of (*R*)-4-ethyl-1-(2-methylbenzyl)-1,4-dihydropyridine-3-carbonitrile (3h)

^{1}H NMR with $CDCl_3$, 400 MHz

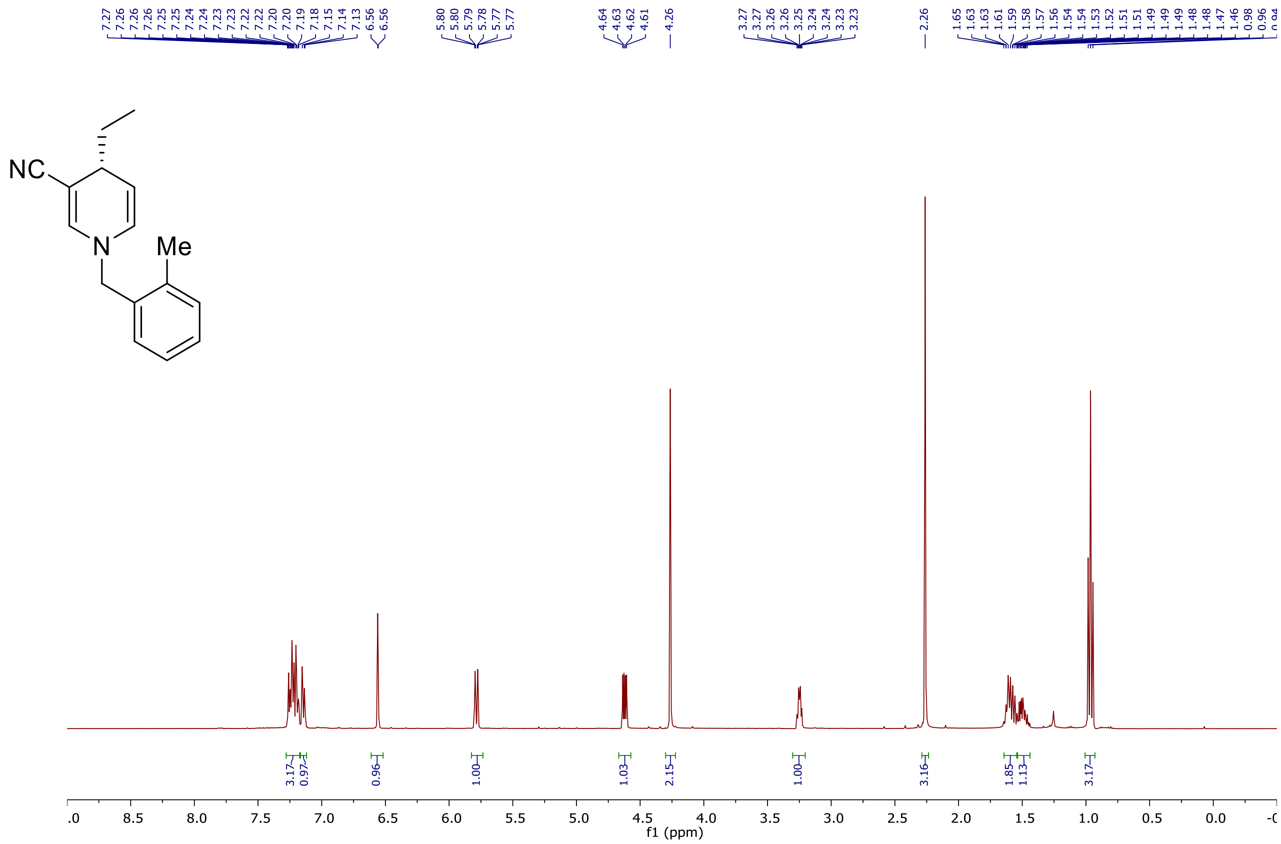

^{13}C NMR with $CDCl_3$, 101 MHz

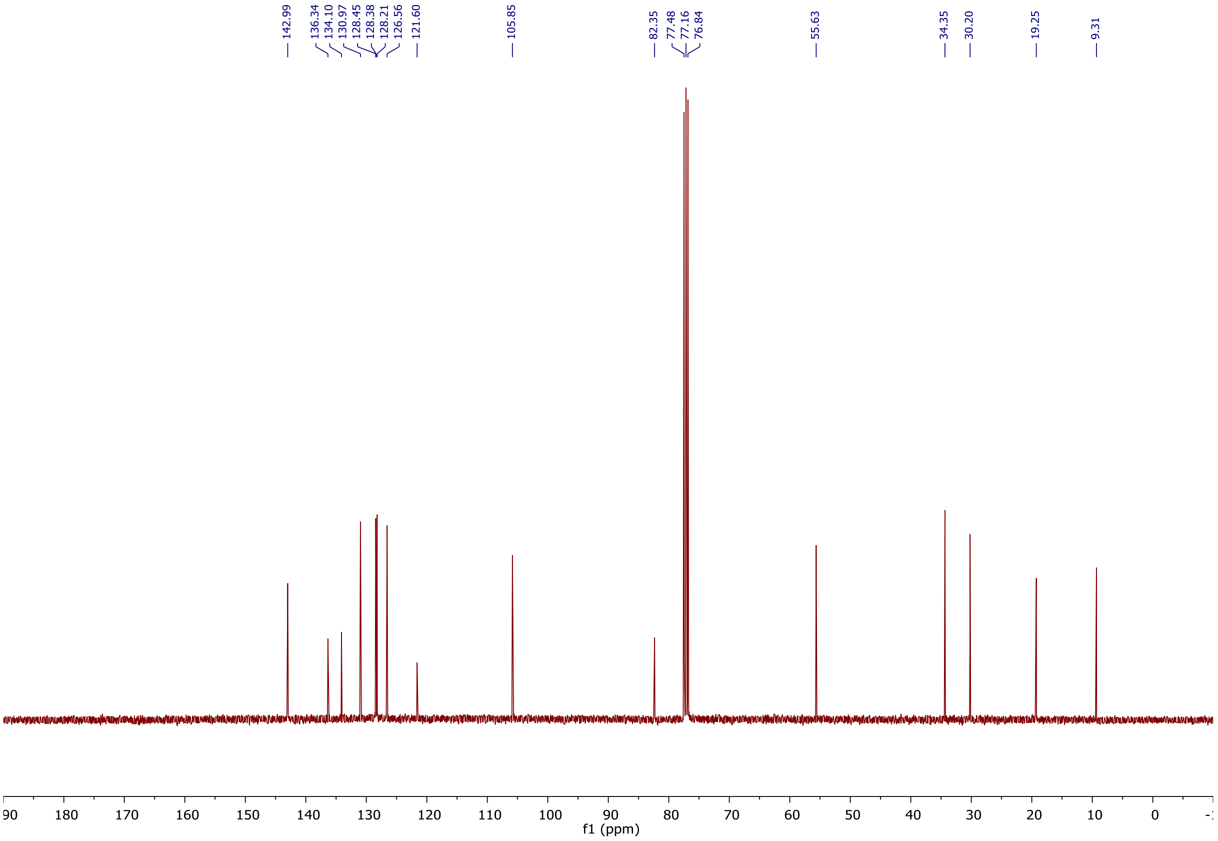

NMR spectra of (*R*)-1-(3,5-di-*tert*-butylbenzyl)-4-ethyl-1,4-dihydropyridine-3-carbonitrile (3i)

^{1}H NMR with $CDCl_3$, 400 MHz

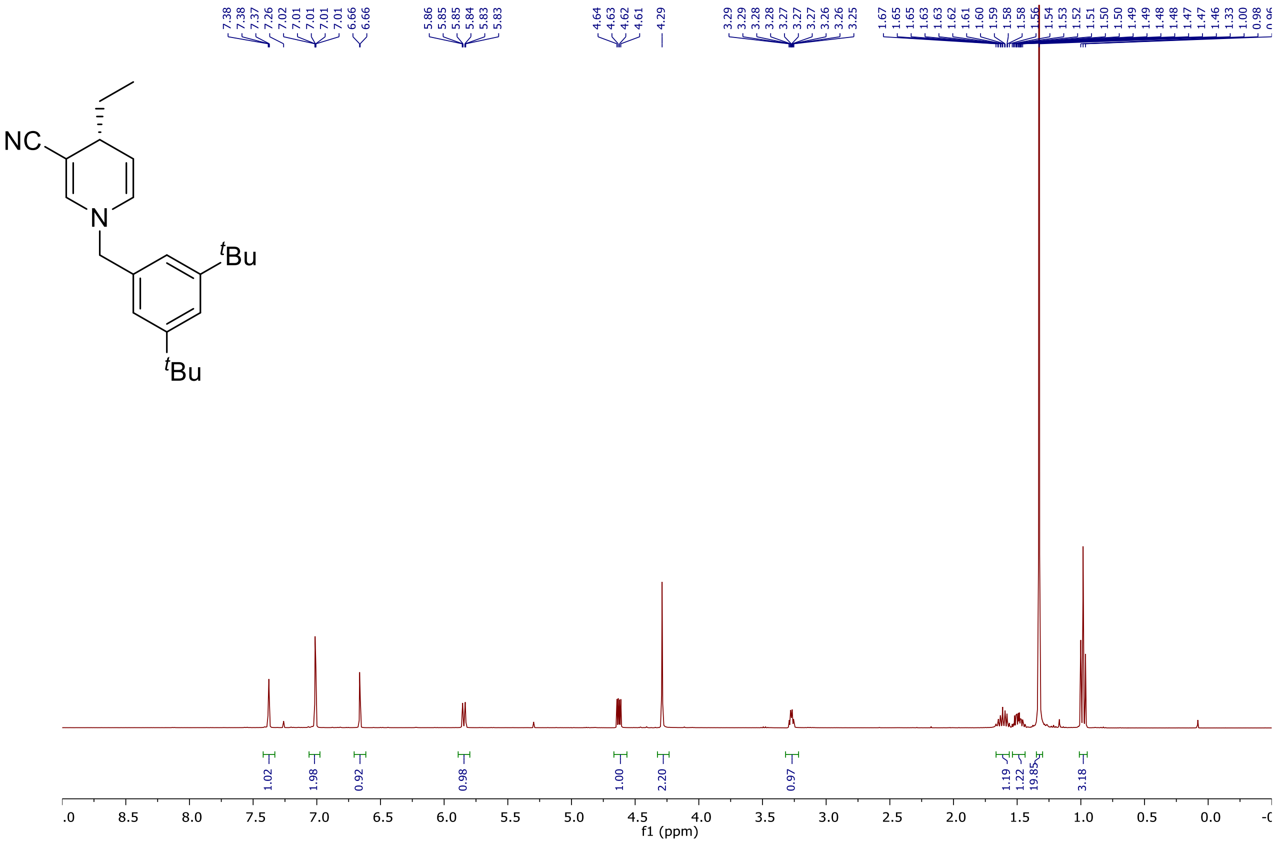

^{13}C NMR with $CDCl_3$, 101 MHz

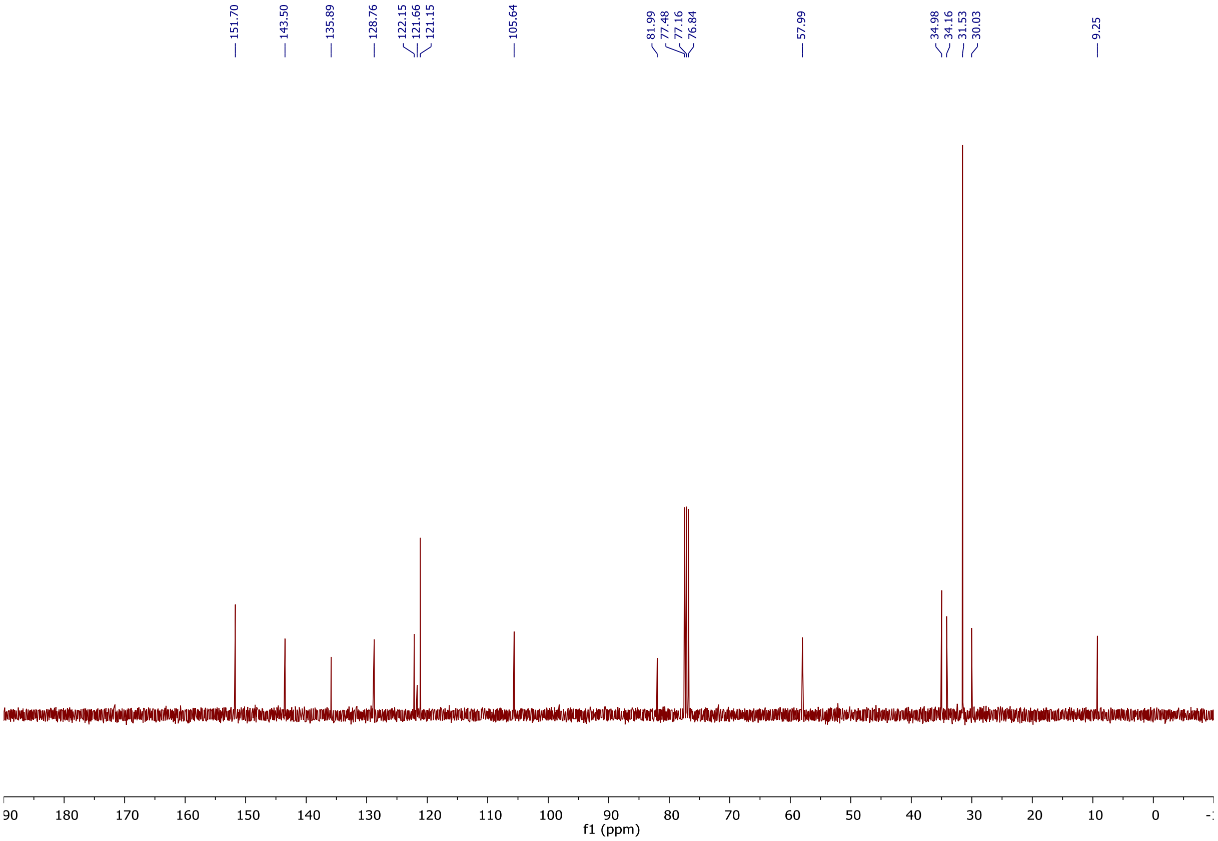

NMR spectra of (*R*)-1-(3,5-dimethoxybenzyl)-4-ethyl-1,4-dihydropyridine-3-carbonitrile (3j)

^{1}H NMR with $CDCl_3$, 400 MHz

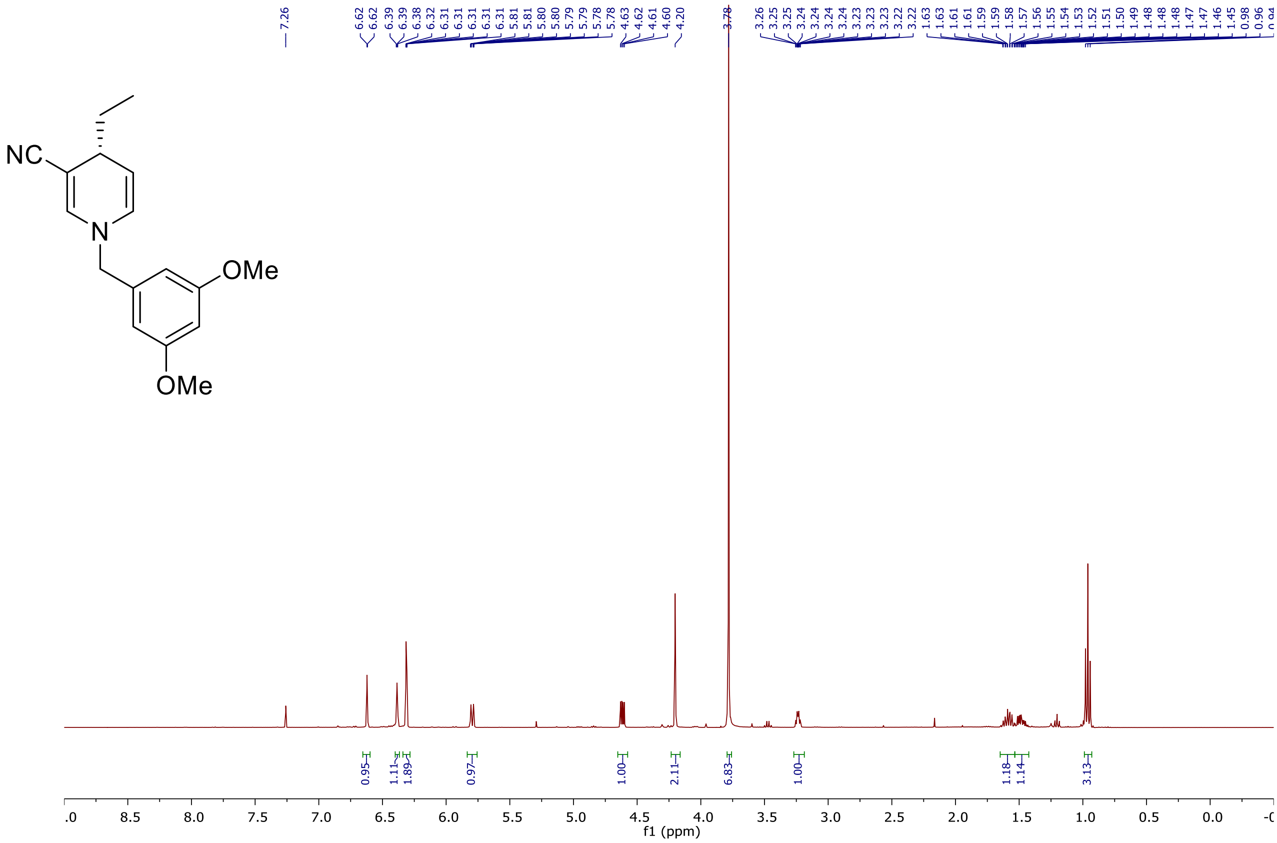

^{13}C NMR with $CDCl_3$, 101 MHz

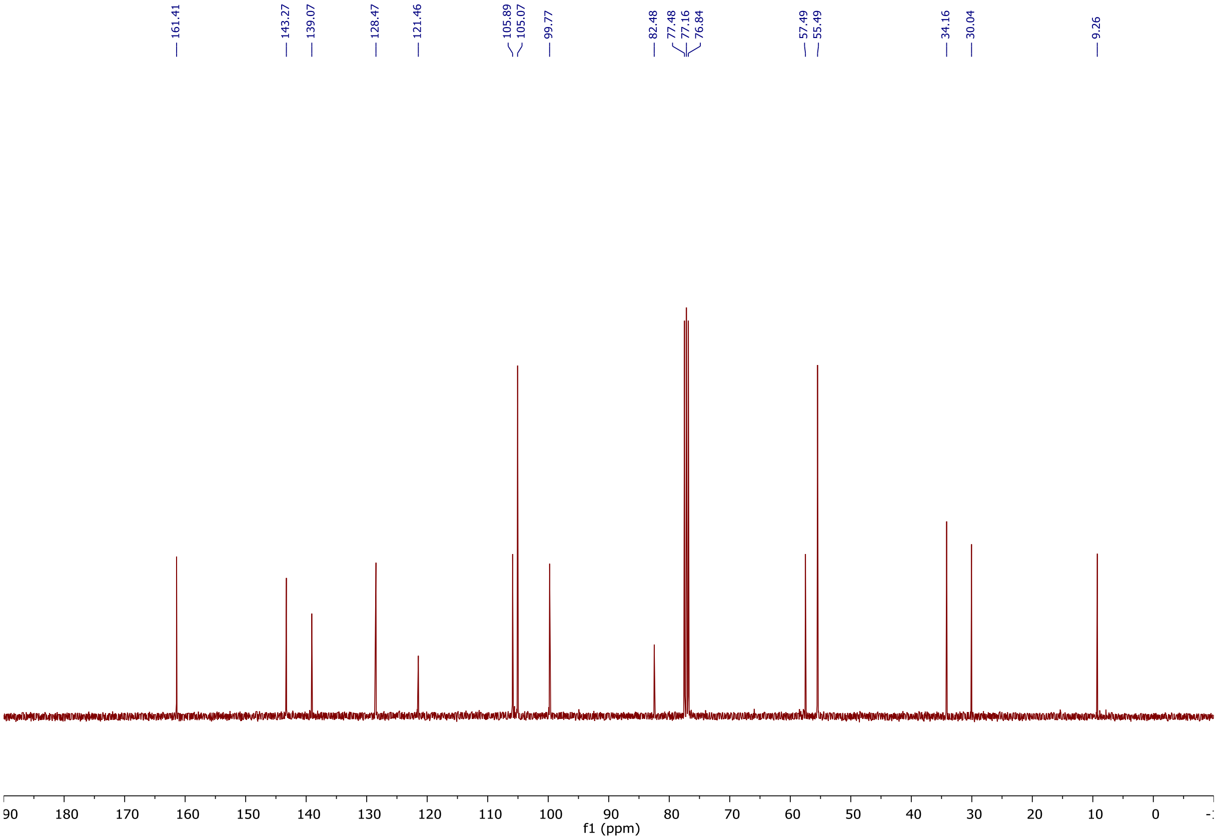

NMR spectra of (*R*)-1-(3,5-bis(trifluoromethyl)benzyl)-4-ethyl-1,4-dihydropyridine-3-carbonitrile (3k)

^{1}H NMR with $CDCl_3$, 400 MHz

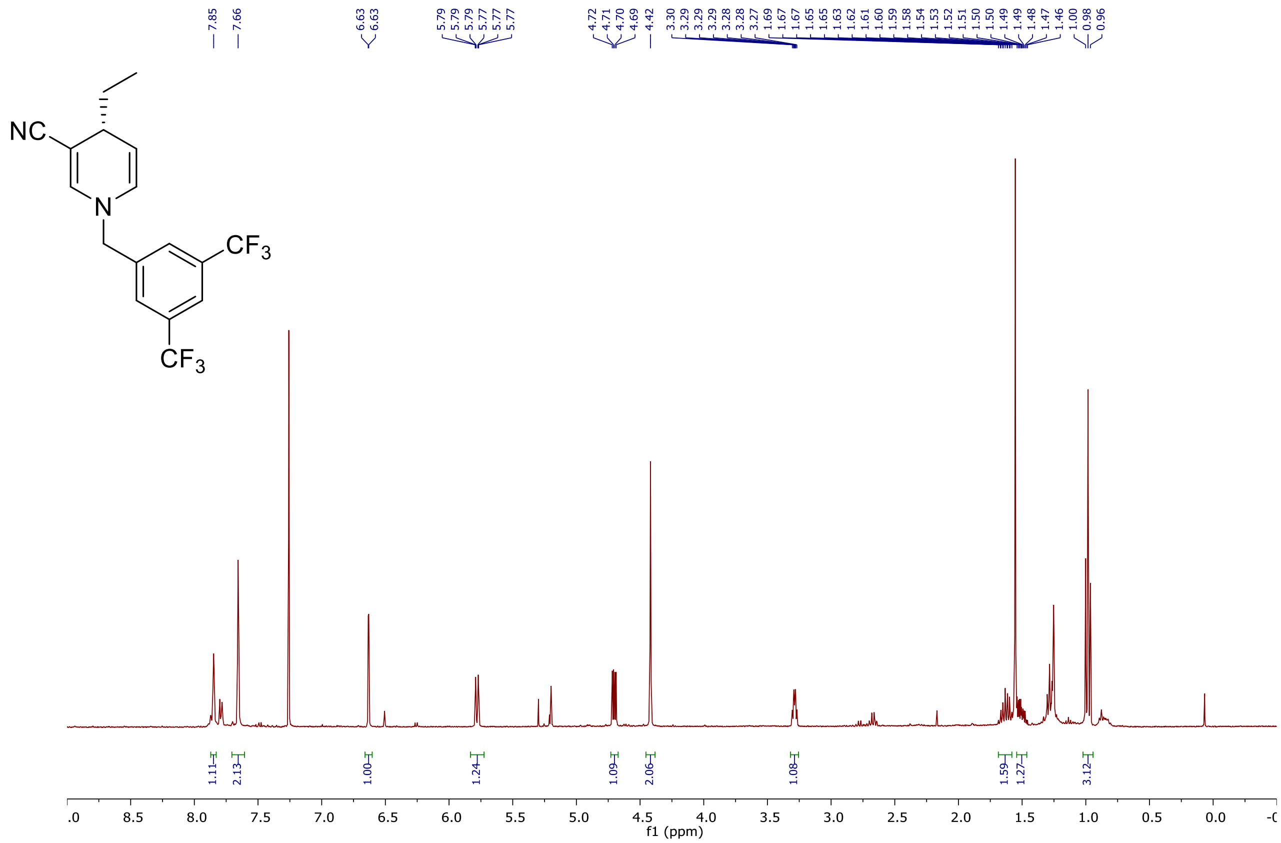

^{13}C NMR with $CDCl_3$, 101 MHz

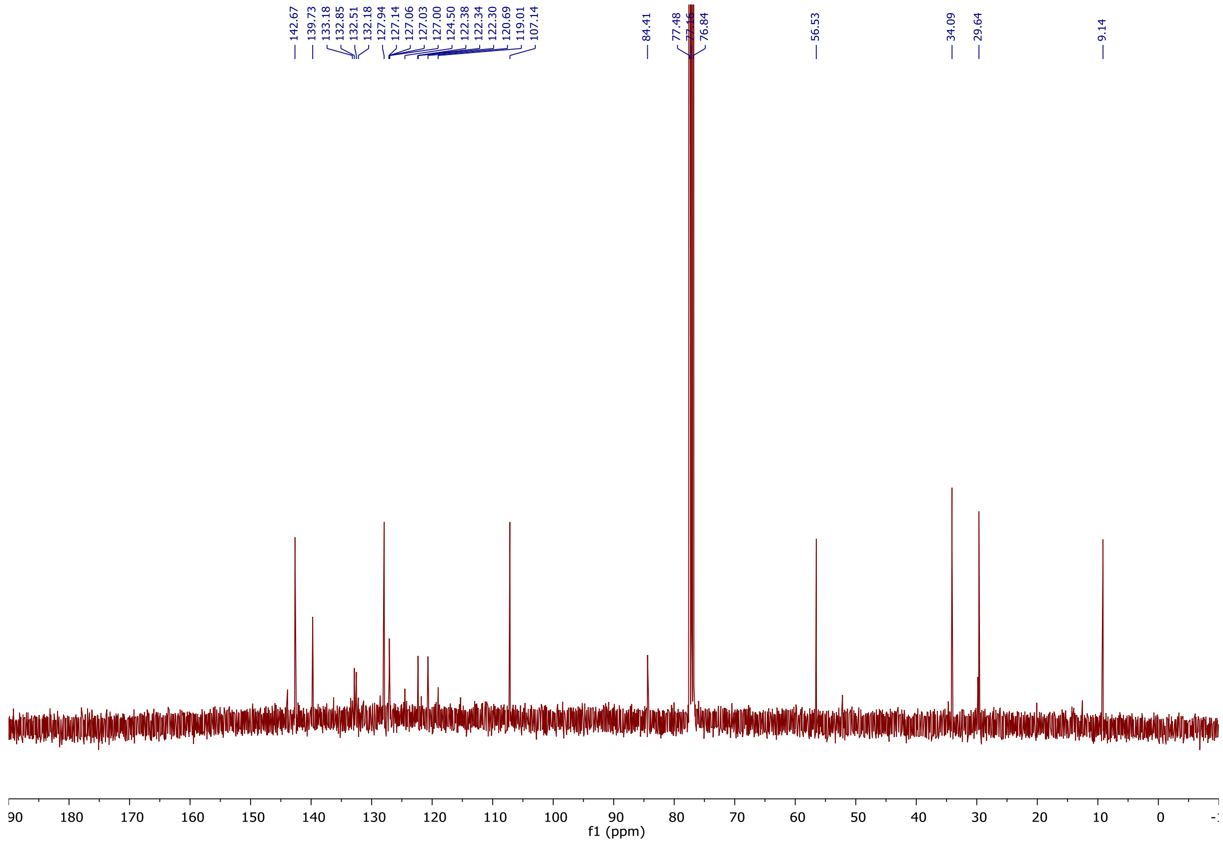

^{19}F NMR with $CDCl_3$, 376 MHz

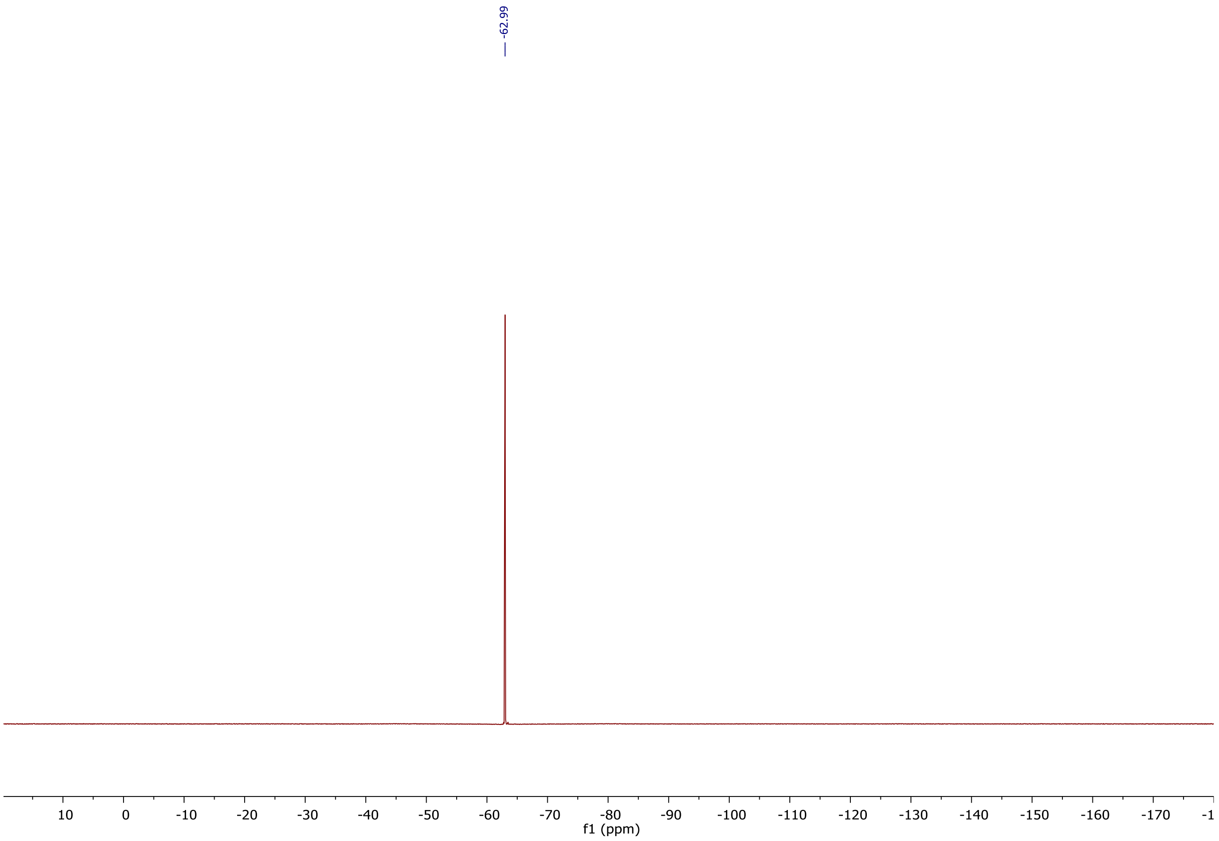

NMR spectra of (*R*)-4-ethyl-1-methyl-1,4-dihydropyridine-3-carbonitrile (3l)

^{1}H NMR with $CDCl_3$, 400 MHz

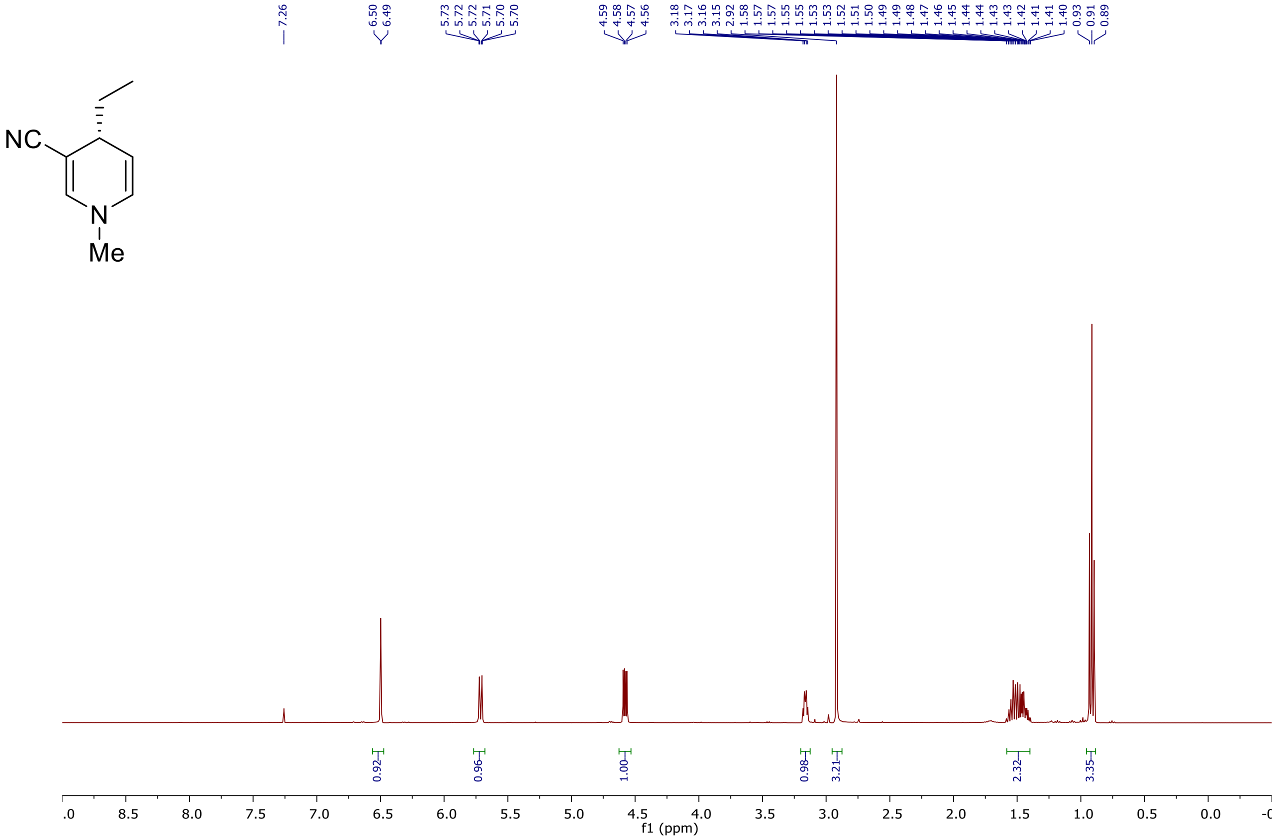

^{13}C NMR with $CDCl_3$, 101 MHz

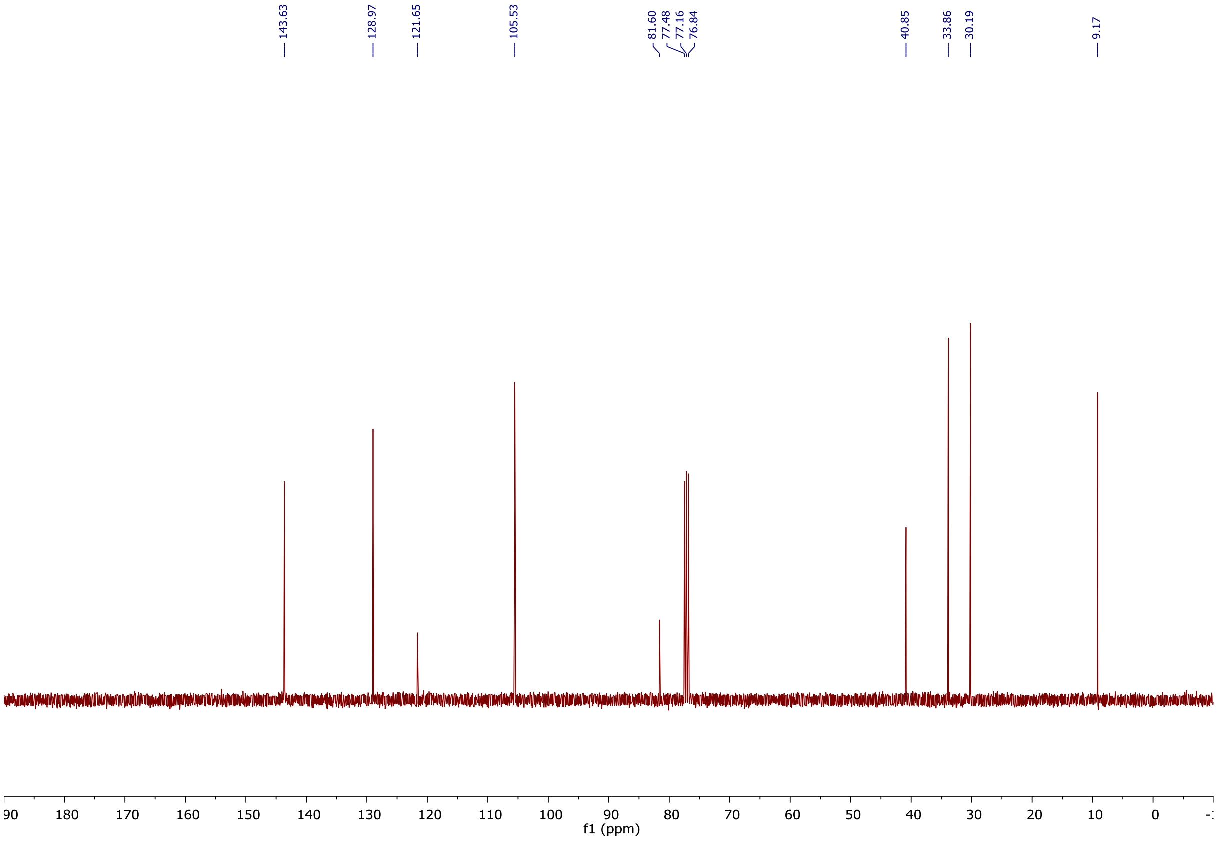

NMR spectra of (*R*)-4-ethyl-1-propyl-1,4-dihydropyridine-3-carbonitrile (3m)

^{1}H NMR with $CDCl_3$, 400 MHz

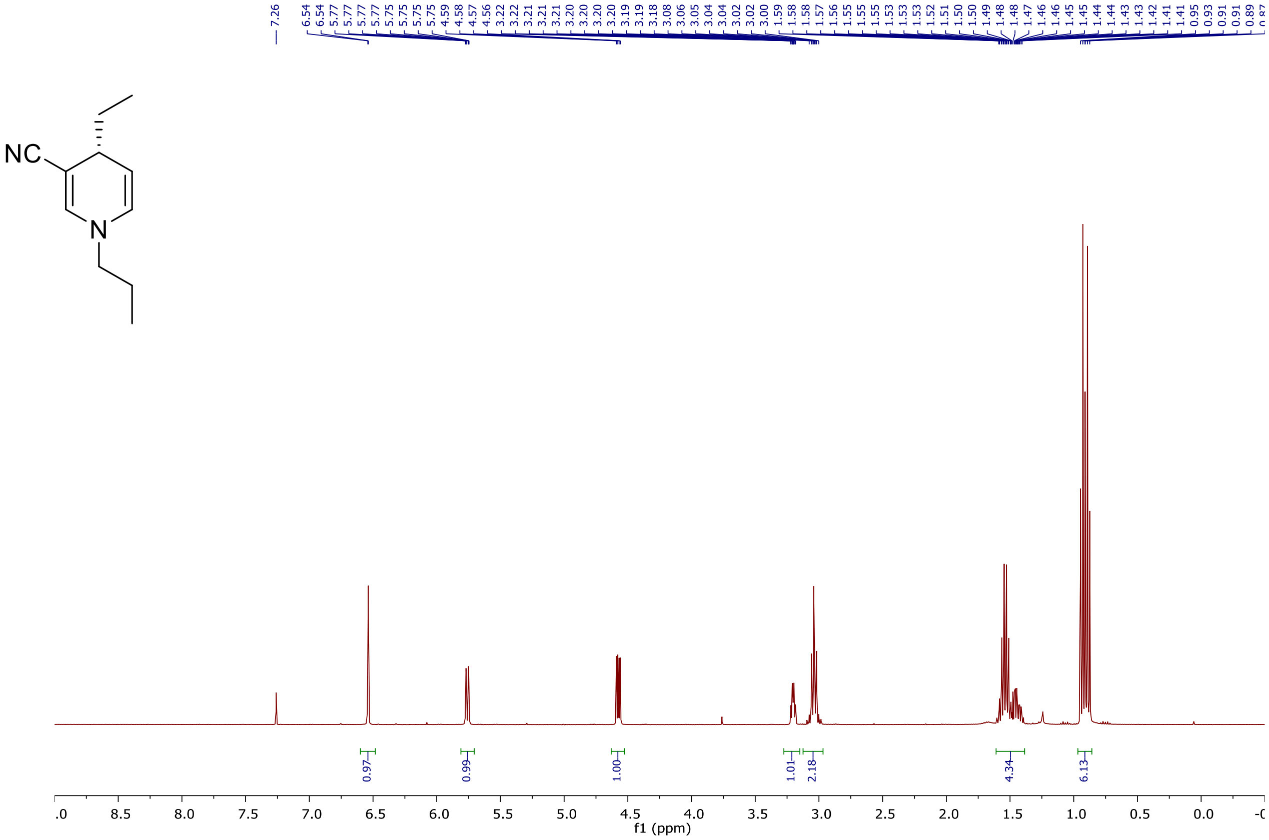

^{13}C NMR with $CDCl_3$, 101 MHz

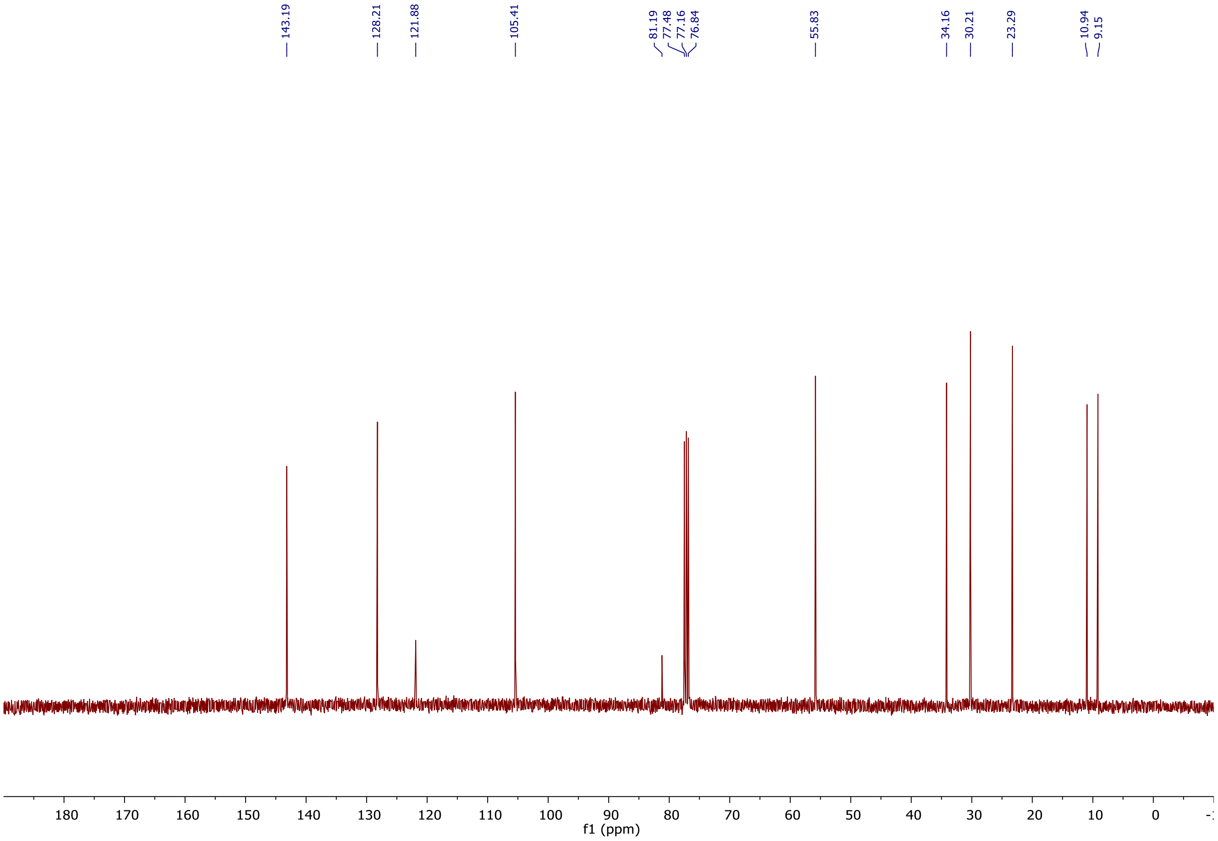

NMR spectra of (*R*)-1-(but-3-en-1-yl)-4-ethyl-1,4-dihydropyridine-3-carbonitrile (3n)

^{1}H NMR with $CDCl_3$, 400 MHz

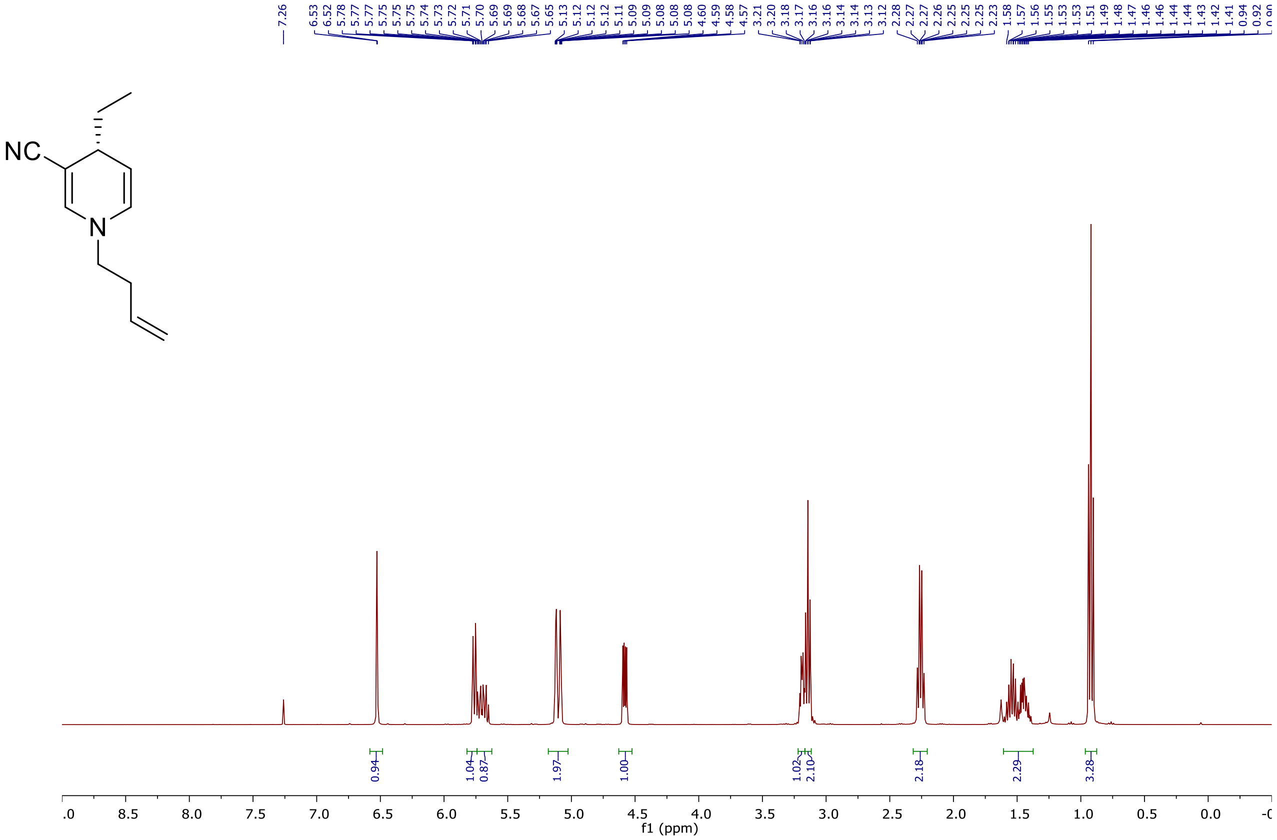

^{13}C NMR with $CDCl_3$, 101 MHz

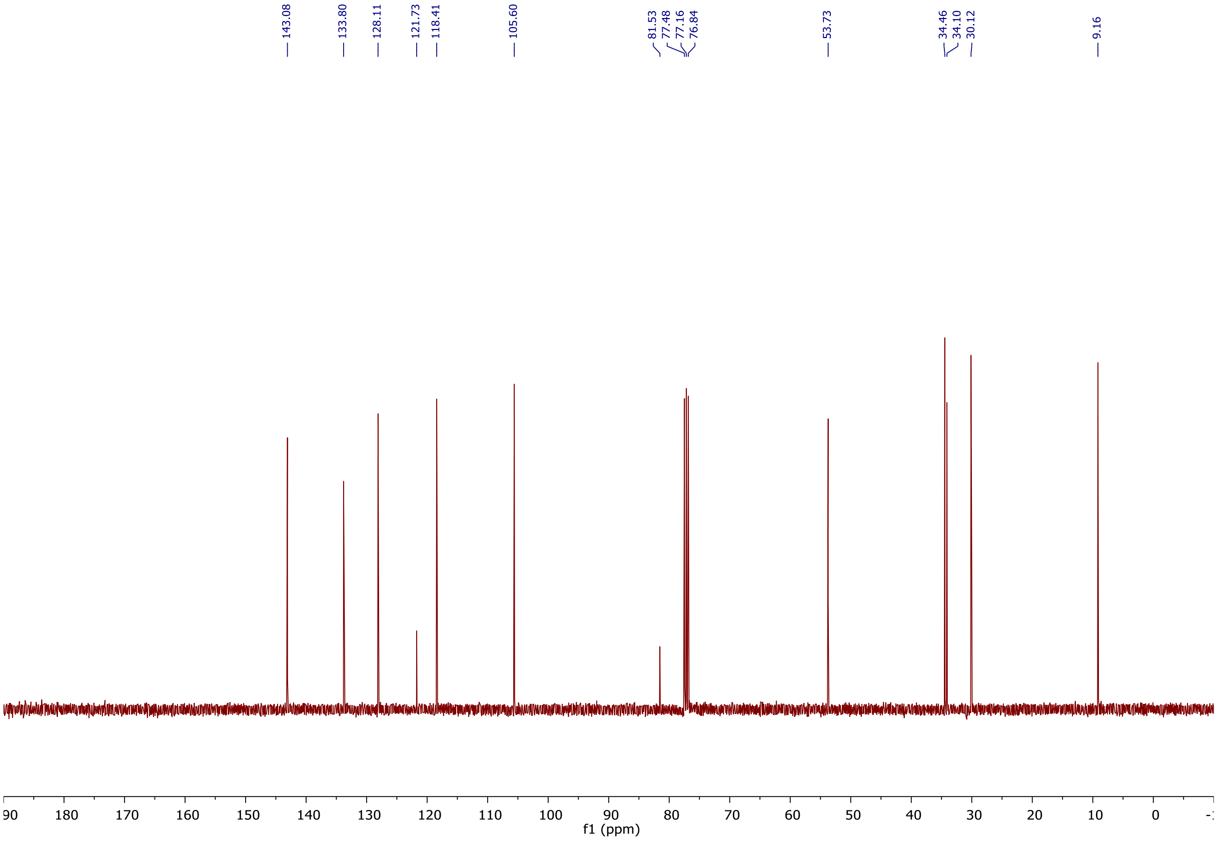

NMR spectra of (*R*)-1-benzyl-4-propyl-1,4-dihydropyridine-3-carbonitrile (4a)

^{1}H NMR with $CDCl_3$, 400 MHz

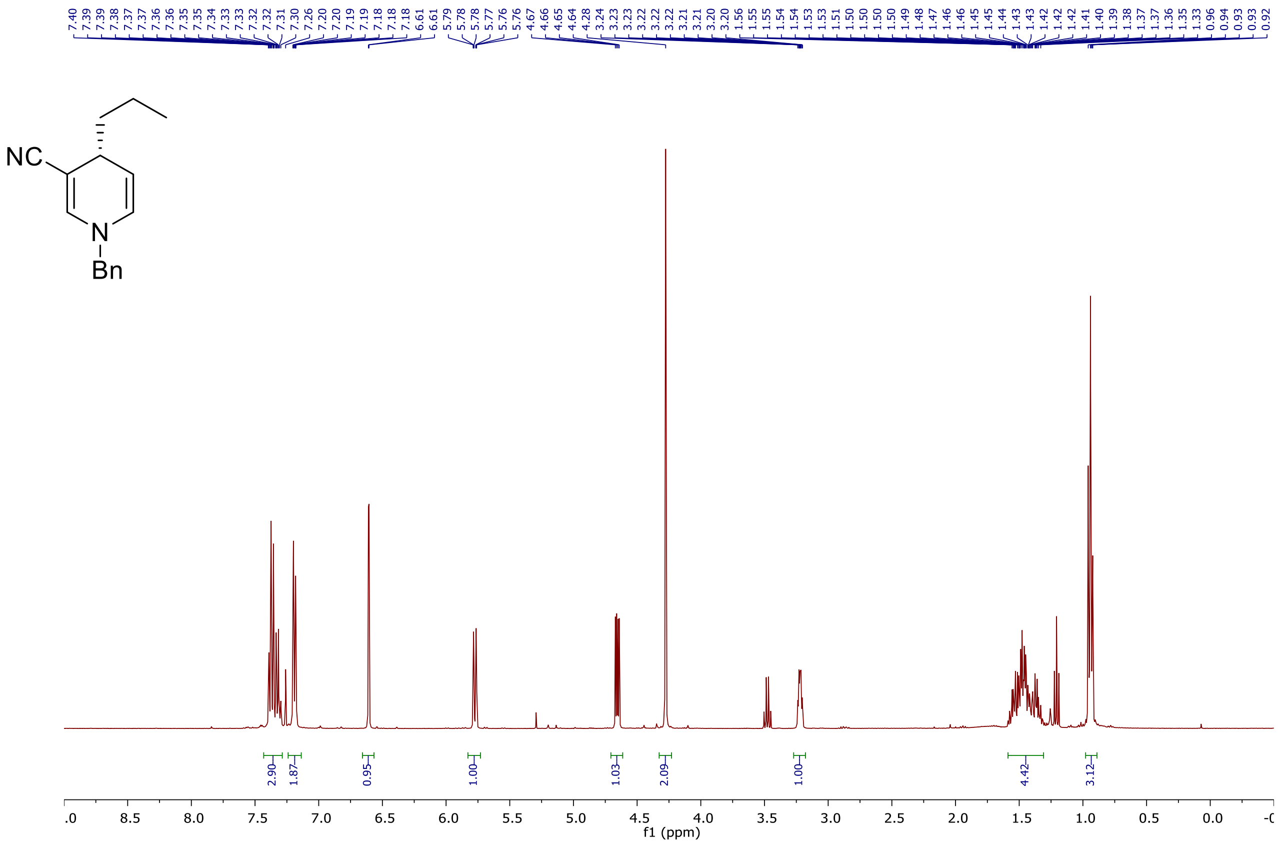

^{13}C NMR with $CDCl_3$, 101 MHz

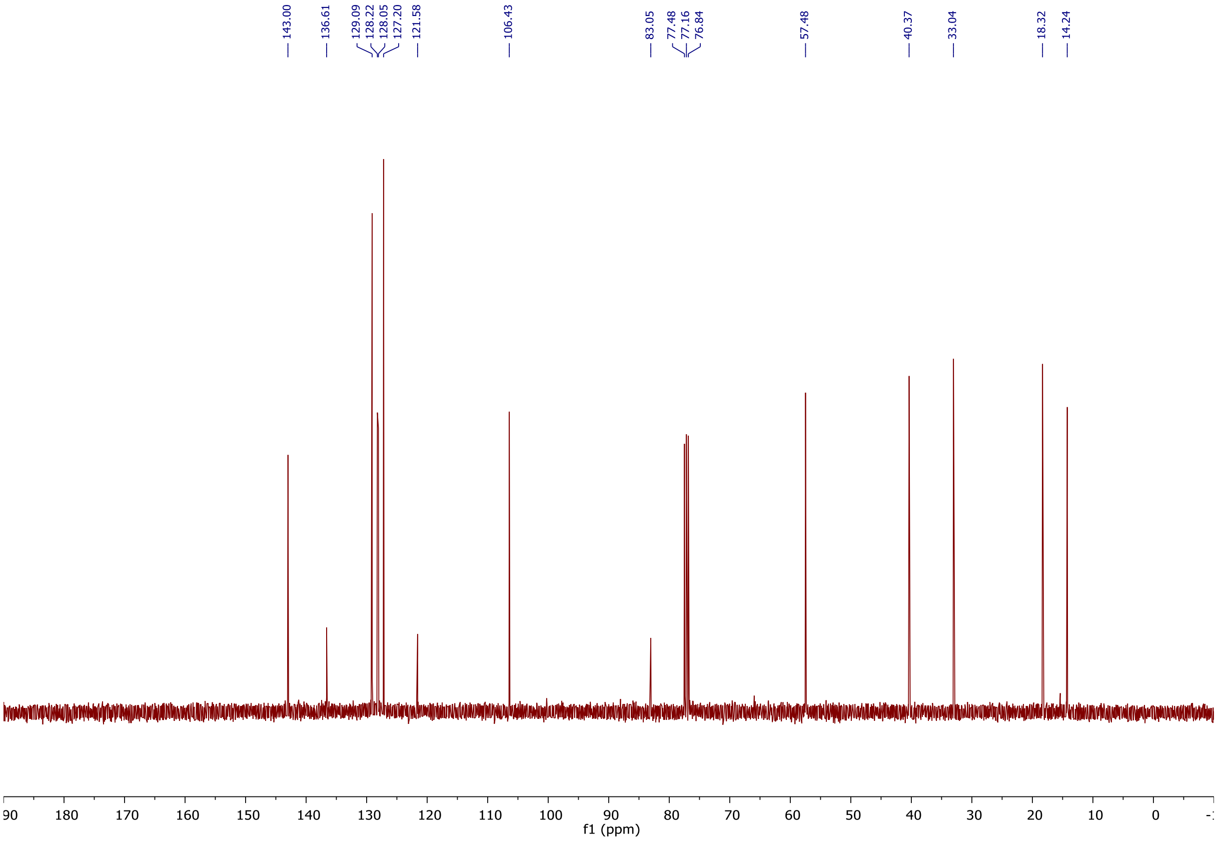

NMR spectra of (*R*)-1-benzyl-4-pentyl-1,4-dihydropyridine-3-carbonitrile (4b)

^{1}H NMR with $CDCl_3$, 400 MHz

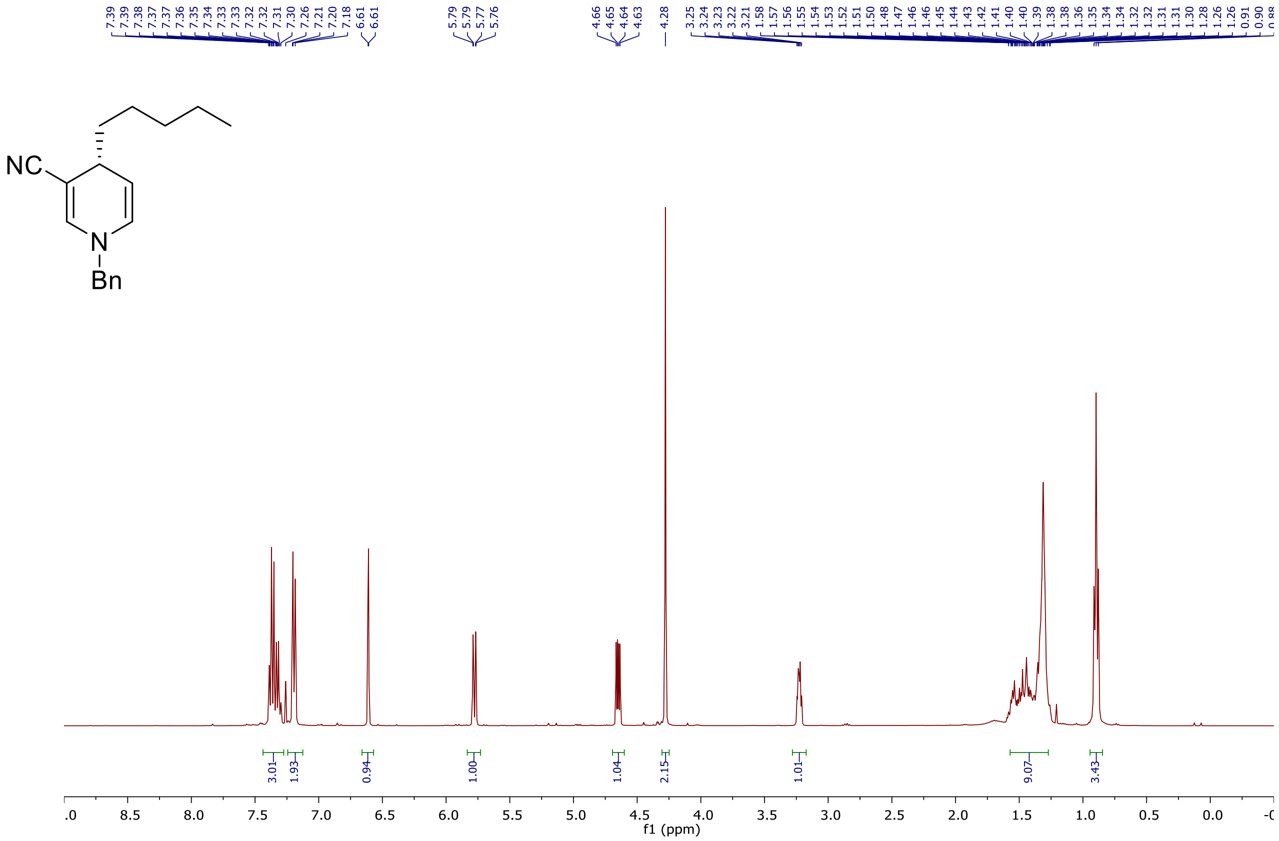

^{13}C NMR with $CDCl_3$, 101 MHz

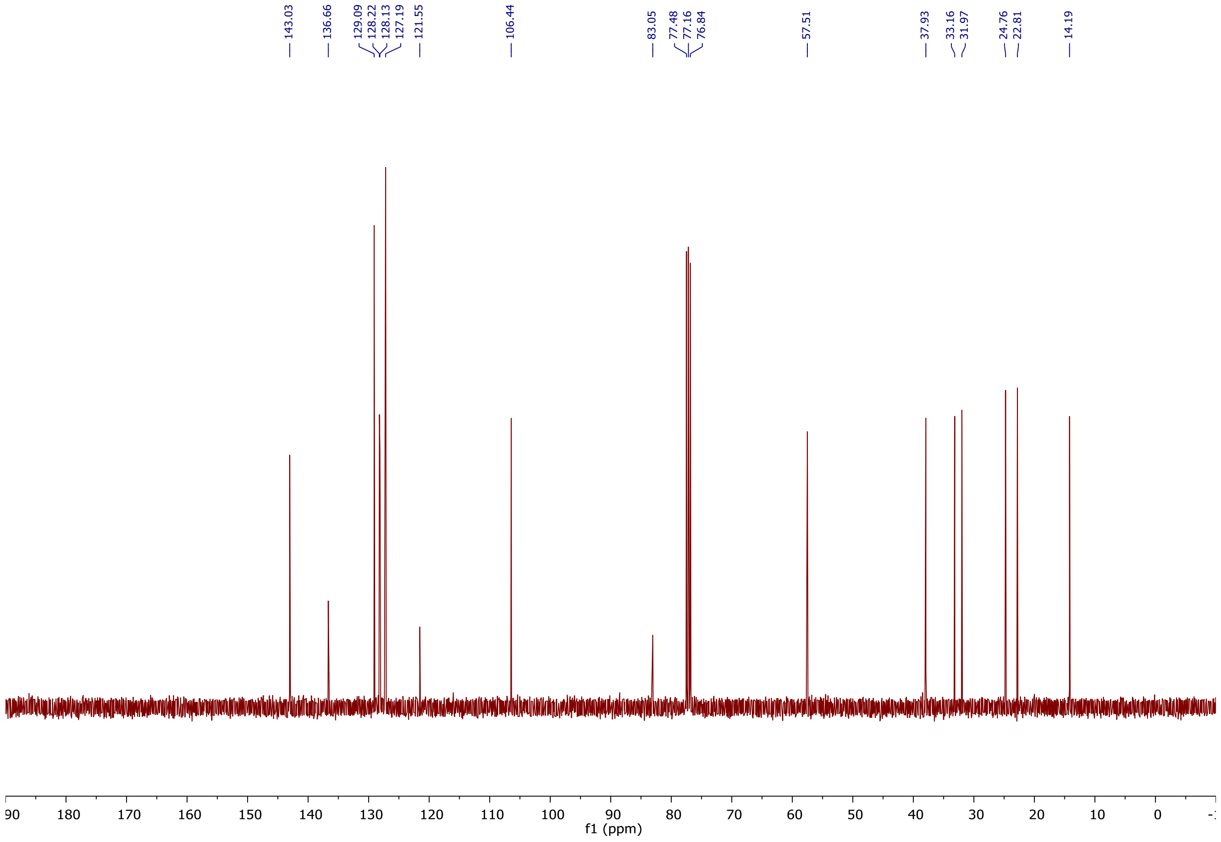

NMR spectra of (*R*)-1-benzyl-4-hexyl-1,4-dihydropyridine-3-carbonitrile (4c)

^{1}H NMR with $CDCl_3$, 400 MHz

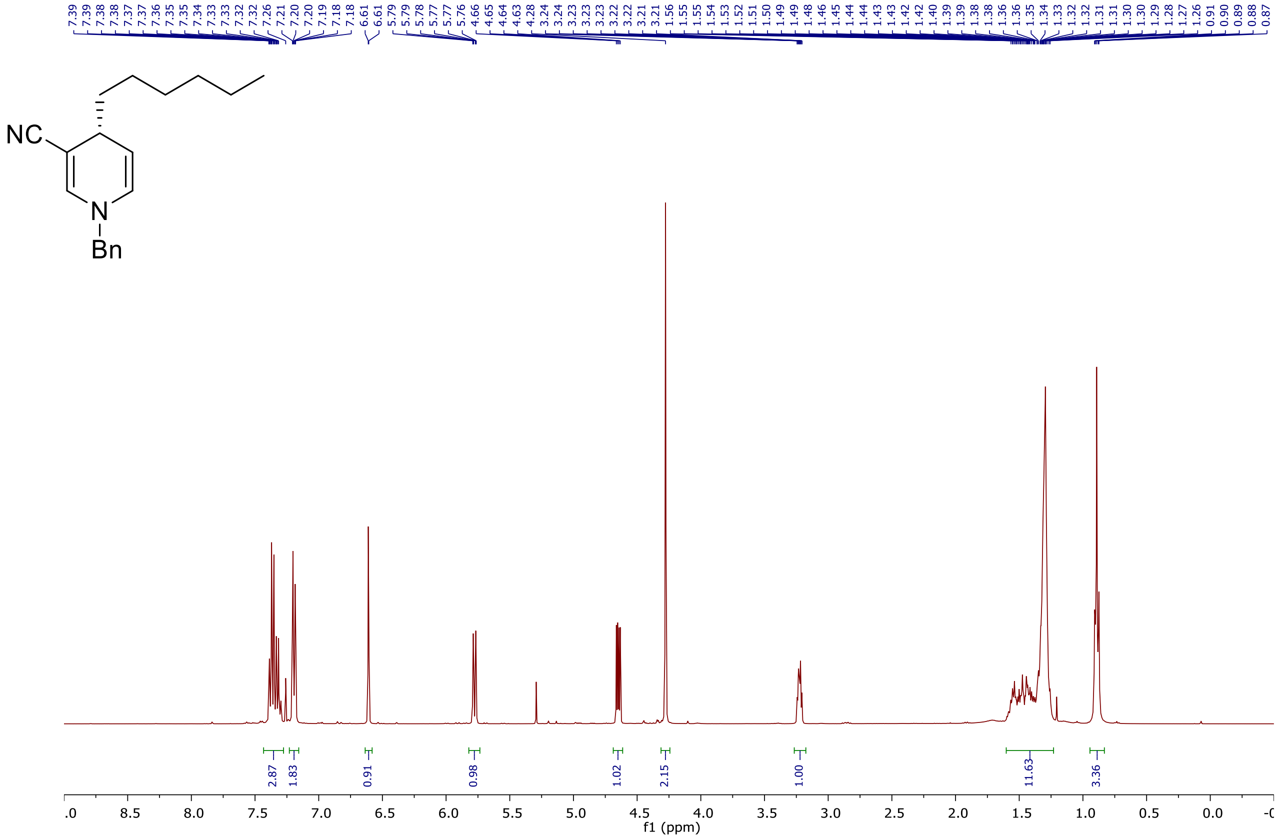

^{13}C NMR with $CDCl_3$, 101 MHz

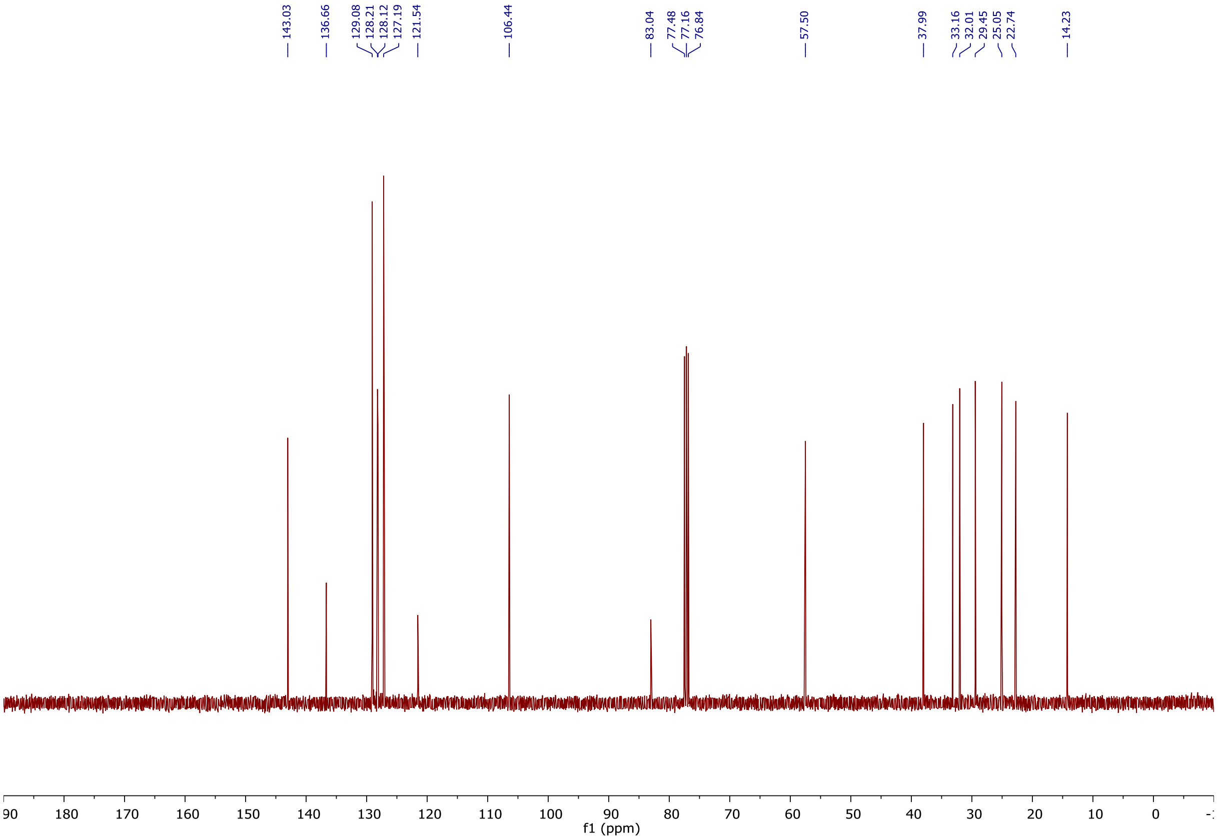

NMR spectra of (*R*)-1-benzyl-4-octyl-1,4-dihydropyridine-3-carbonitrile (4d)

^{1}H NMR with $CDCl_3$, 400 MHz

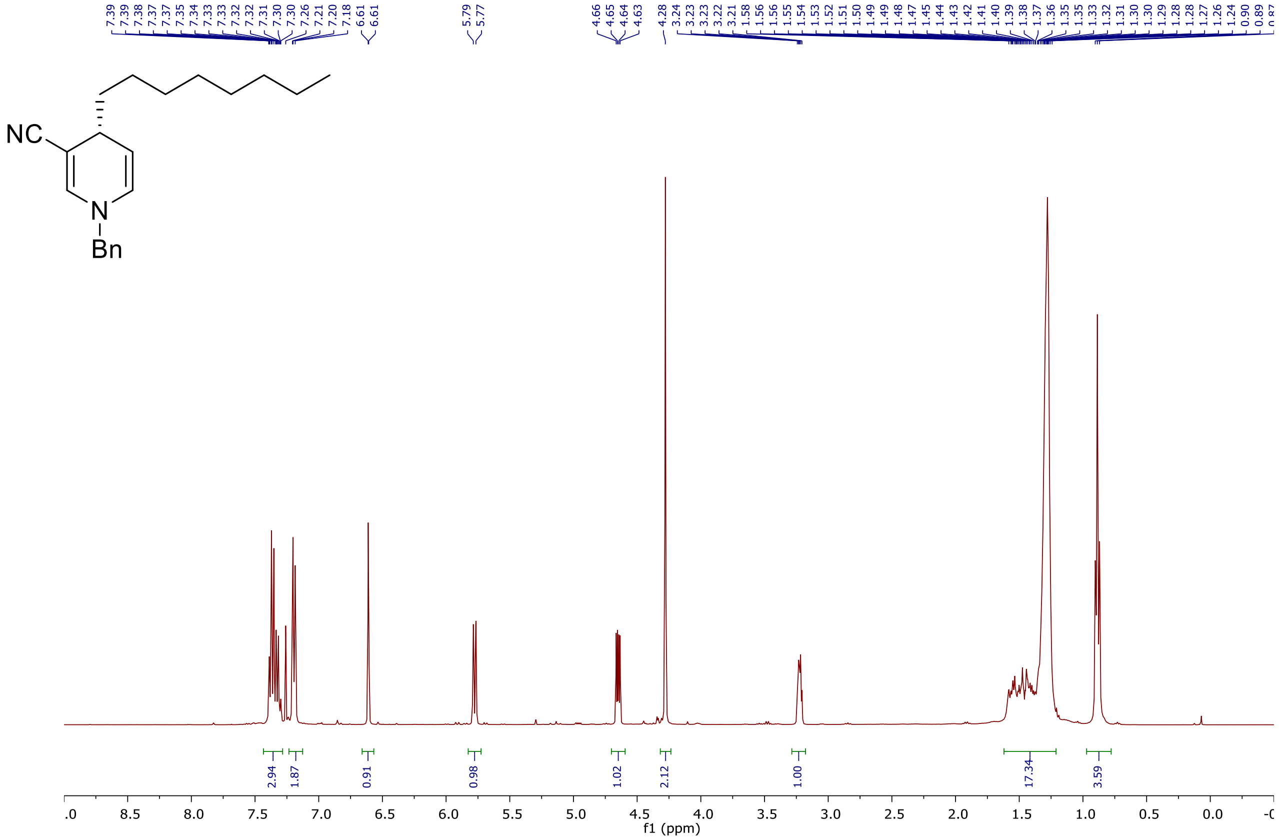

^{13}C NMR with $CDCl_3$, 101 MHz

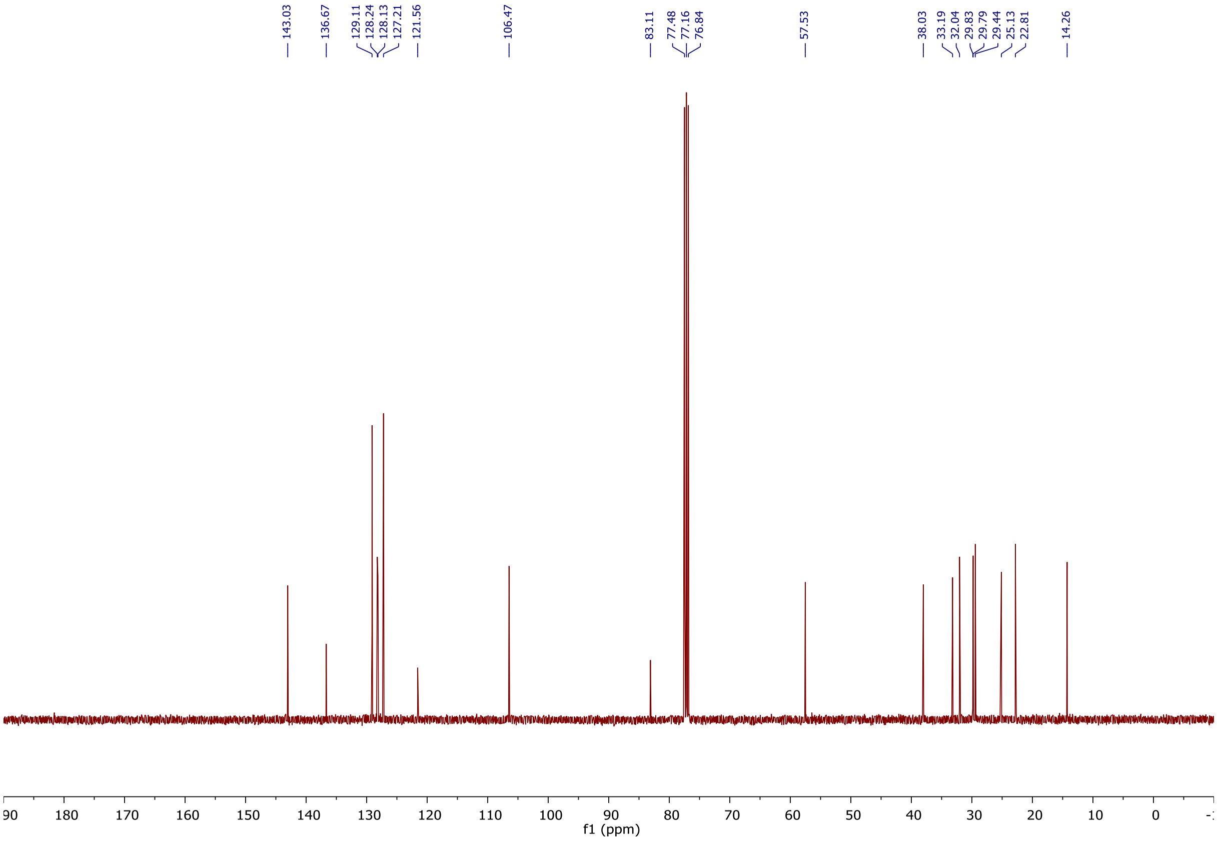

NMR spectra of (*R*)-1-benzyl-4-nonyl-1,4-dihydropyridine-3-carbonitrile (4e)

^{1}H NMR with $CDCl_3$, 400 MHz

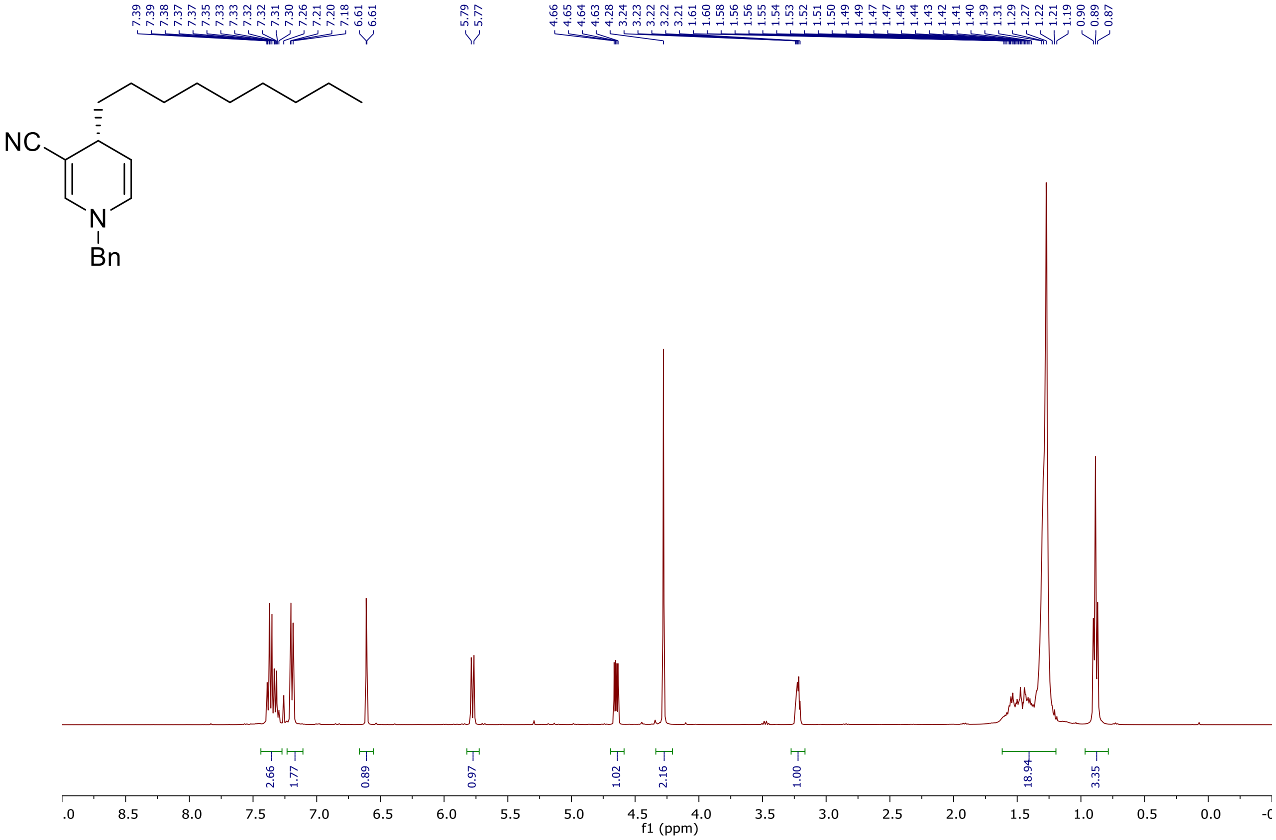

^{13}C NMR with $CDCl_3$, 101 MHz

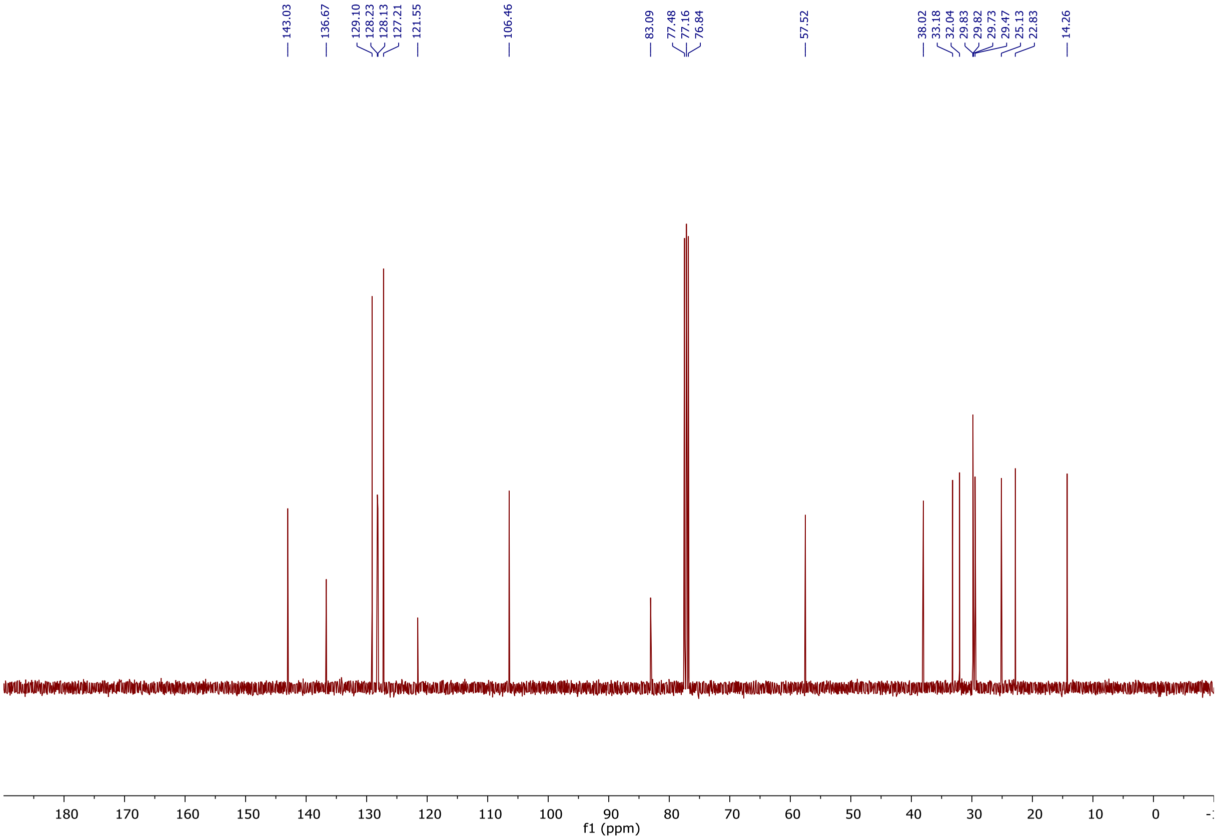

NMR spectra of (*R*)-1-benzyl-4-undecyl-1,4-dihydropyridine-3-carbonitrile (4f)

^{1}H NMR with $CDCl_3$, 400 MHz

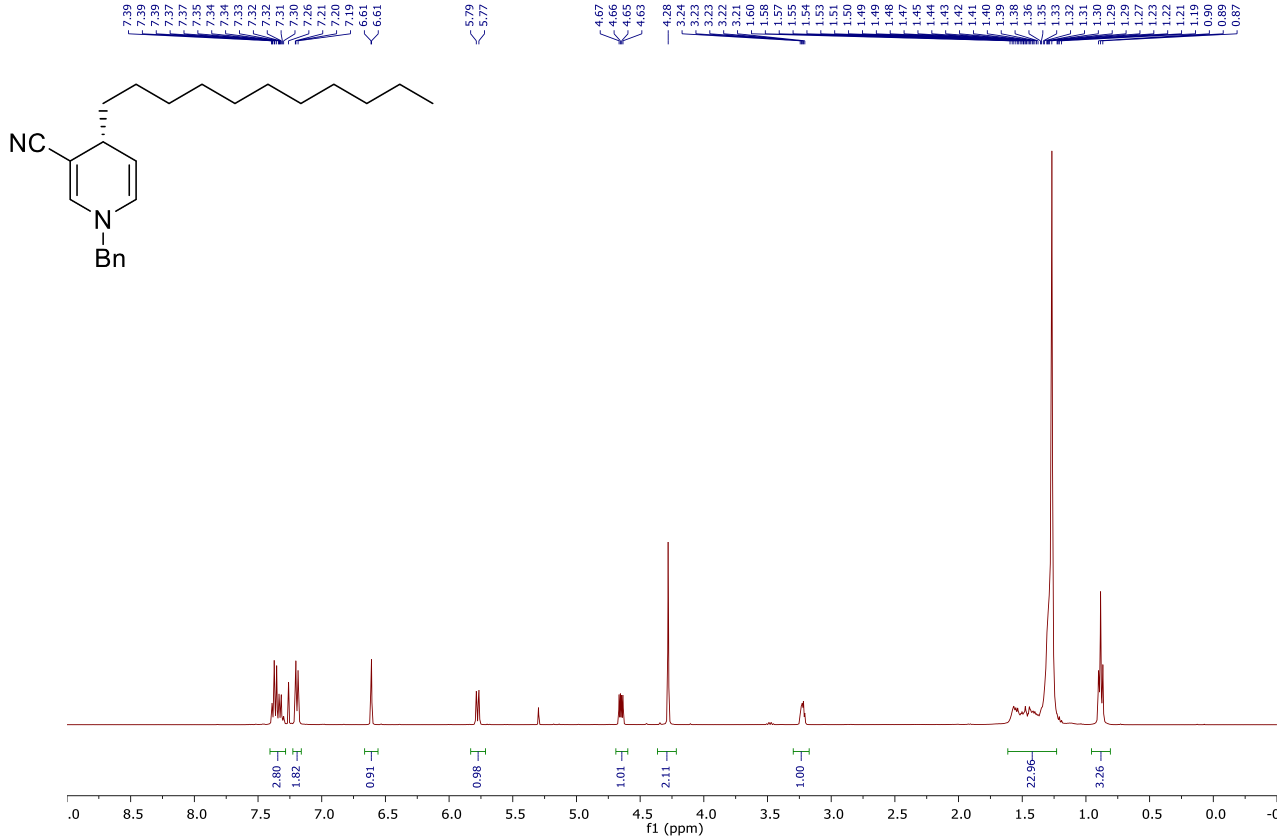

^{13}C NMR with $CDCl_3$, 101 MHz

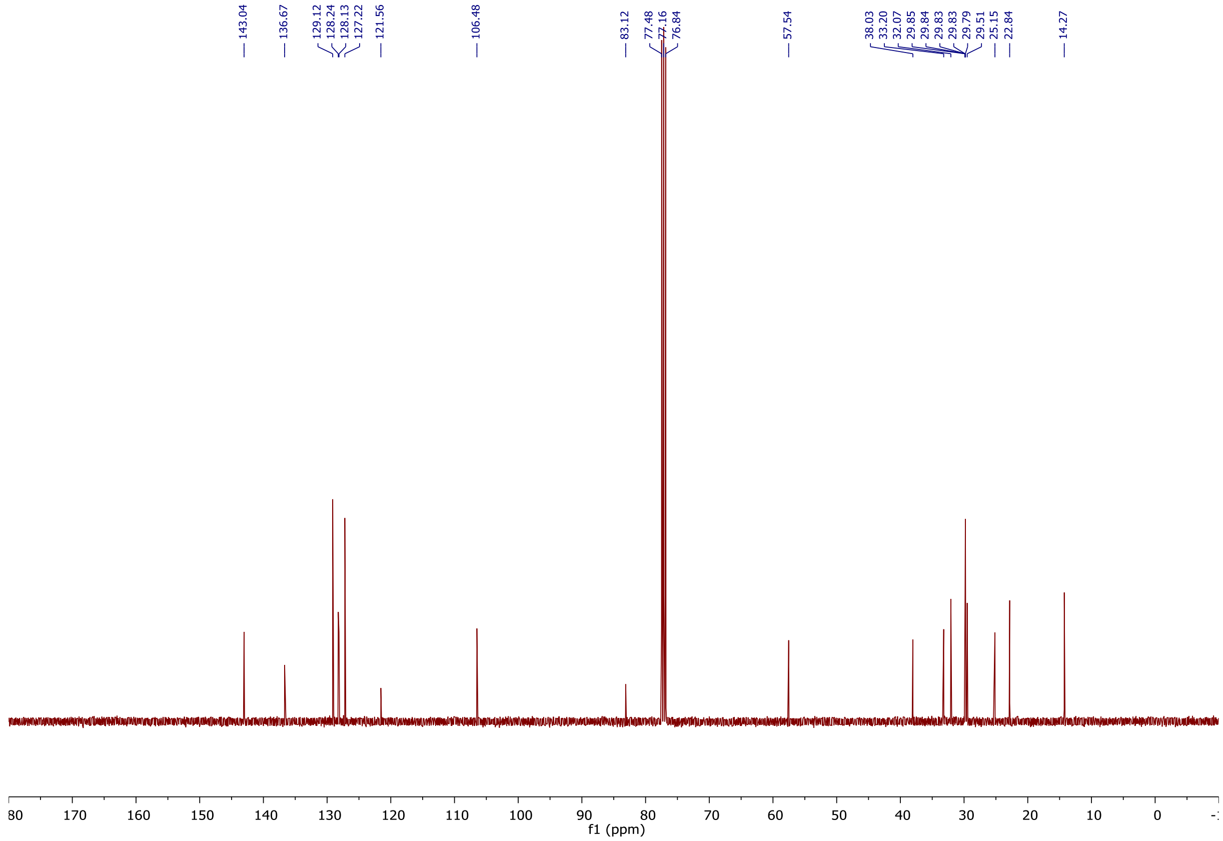

NMR spectra of (*R*)-1-benzyl-4-isopentyl-1,4-dihydropyridine-3-carbonitrile (4g)

^{1}H NMR with $CDCl_3$, 400 MHz

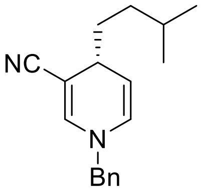

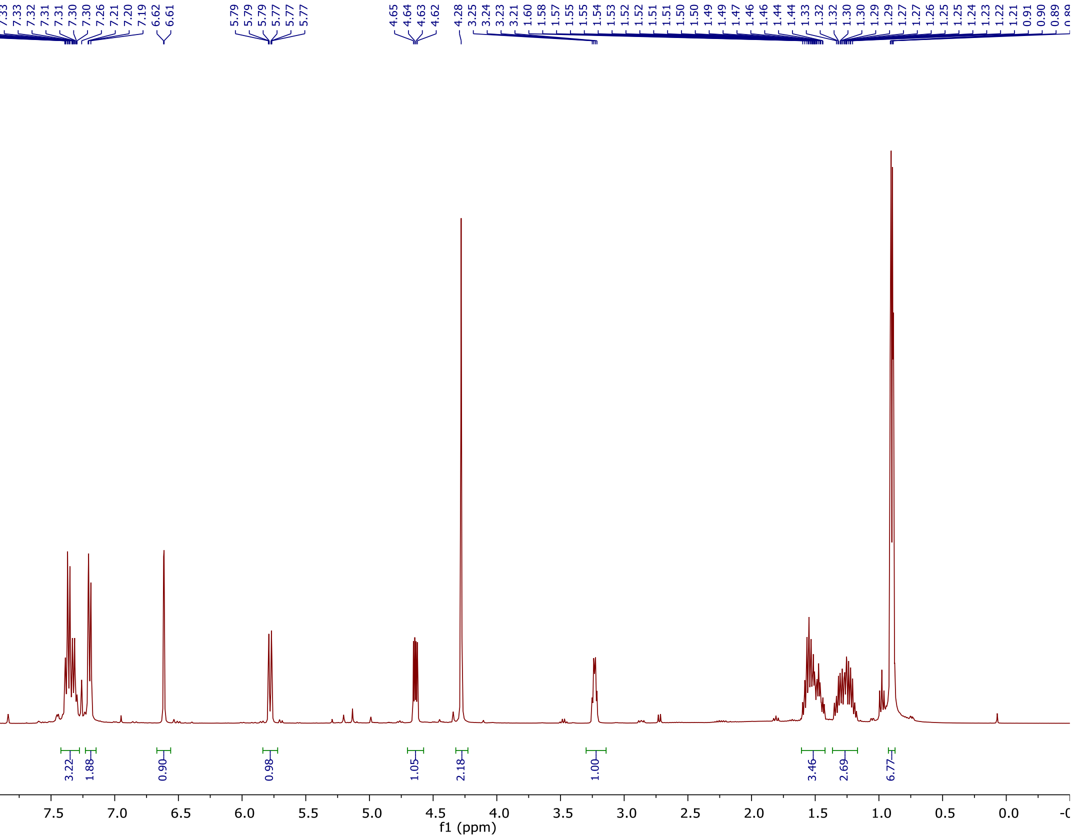

^{13}C NMR with $CDCl_3$, 101 MHz

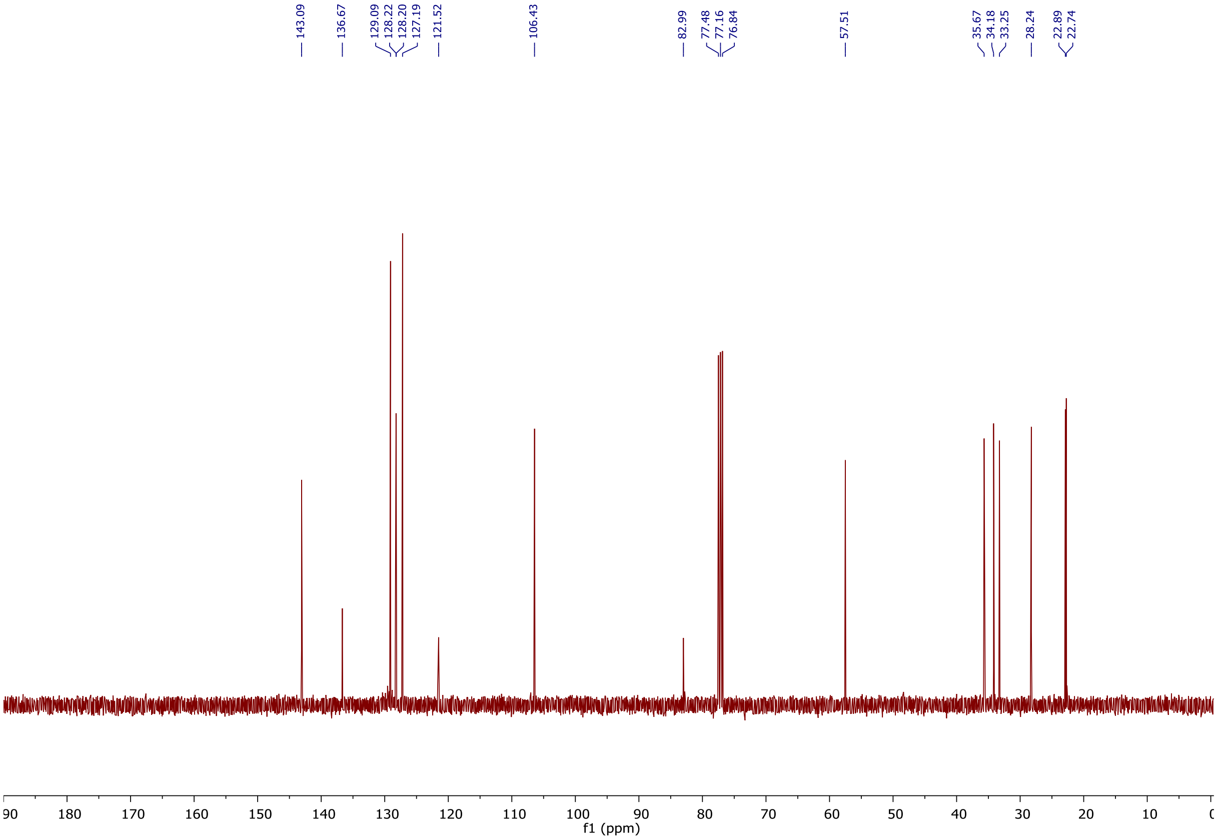

NMR spectra of (*R*)-1-benzyl-4-isobutyl-1,4-dihydropyridine-3-carbonitrile (4h)

^{1}H NMR with $CDCl_3$, 400 MHz

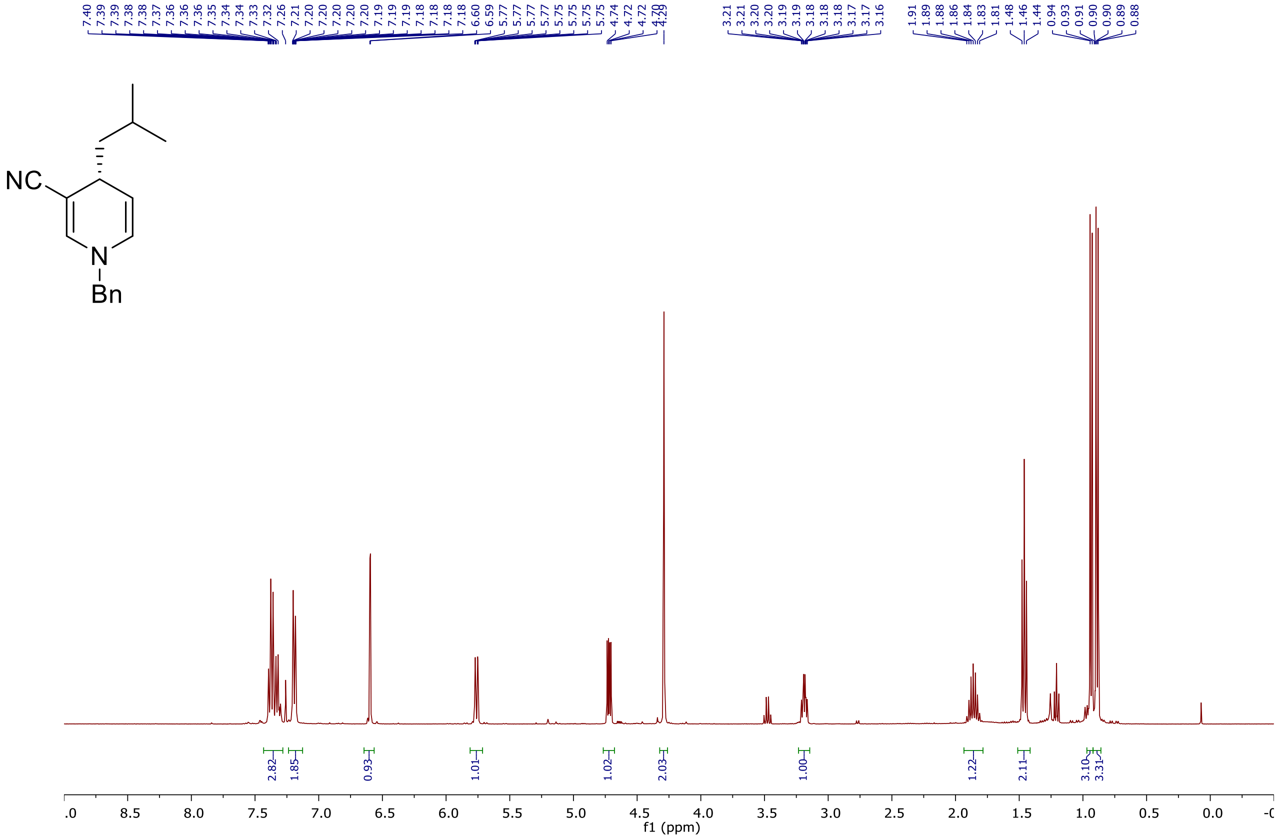

^{13}C NMR with $CDCl_3$, 101 MHz

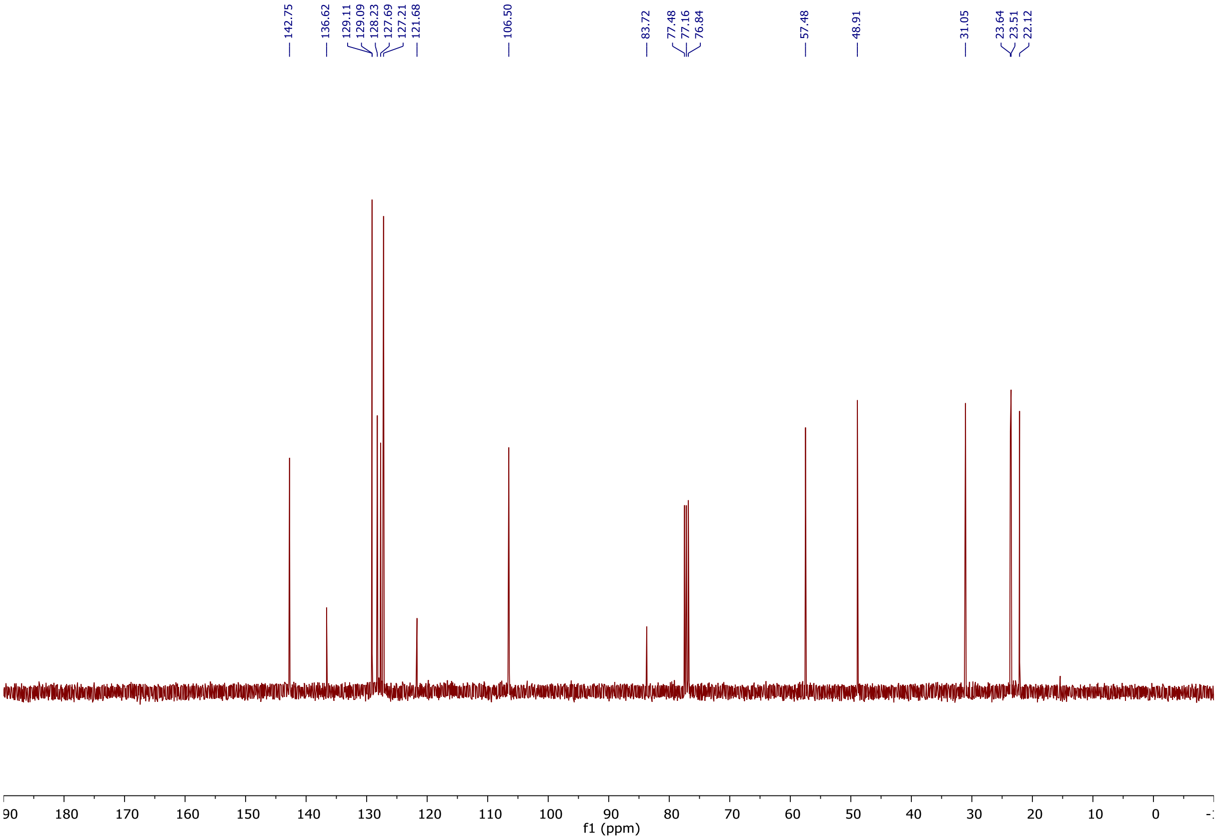

NMR spectra of (*R*)-1-benzyl-4-(cyclohexylmethyl)-1,4-dihydropyridine-3-carbonitrile (4i)

^{1}H NMR with $CDCl_3$, 400 MHz

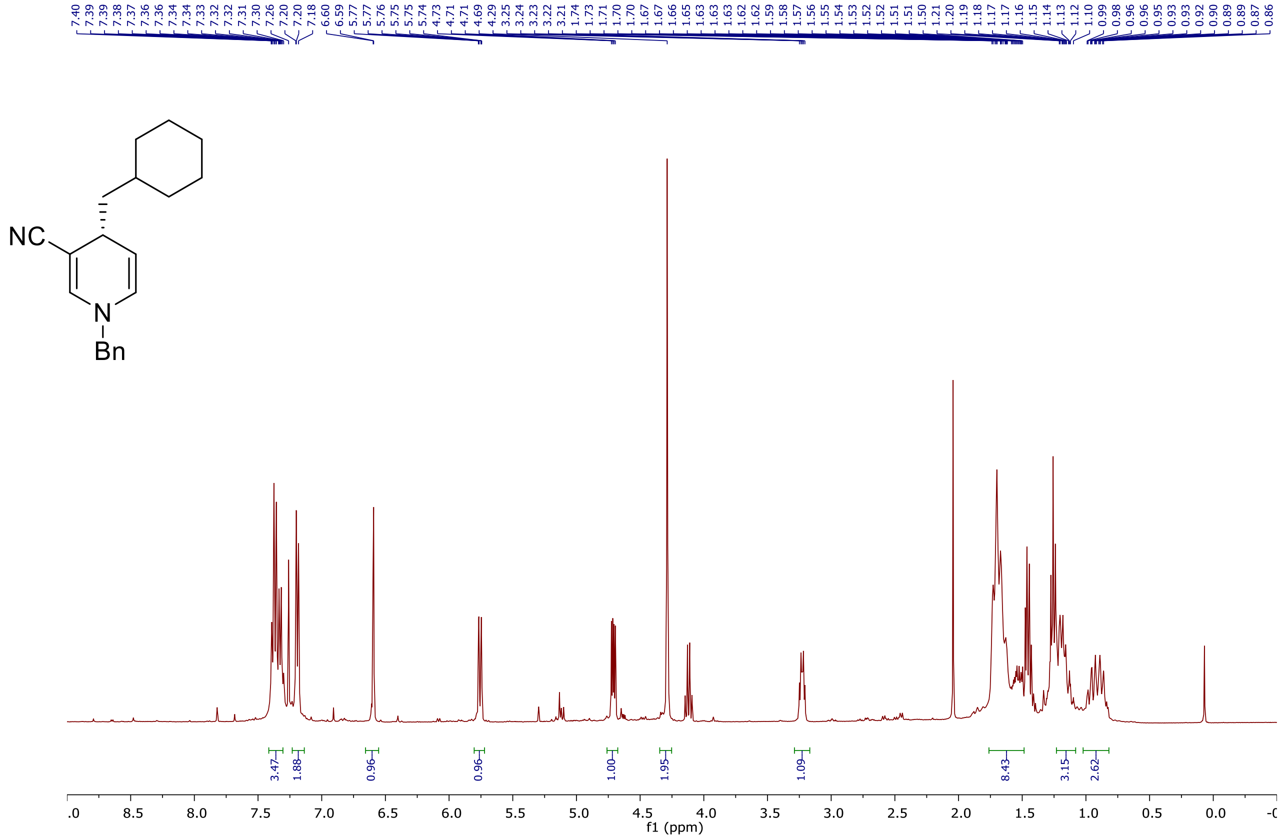

^{13}C NMR with $CDCl_3$, 101 MHz

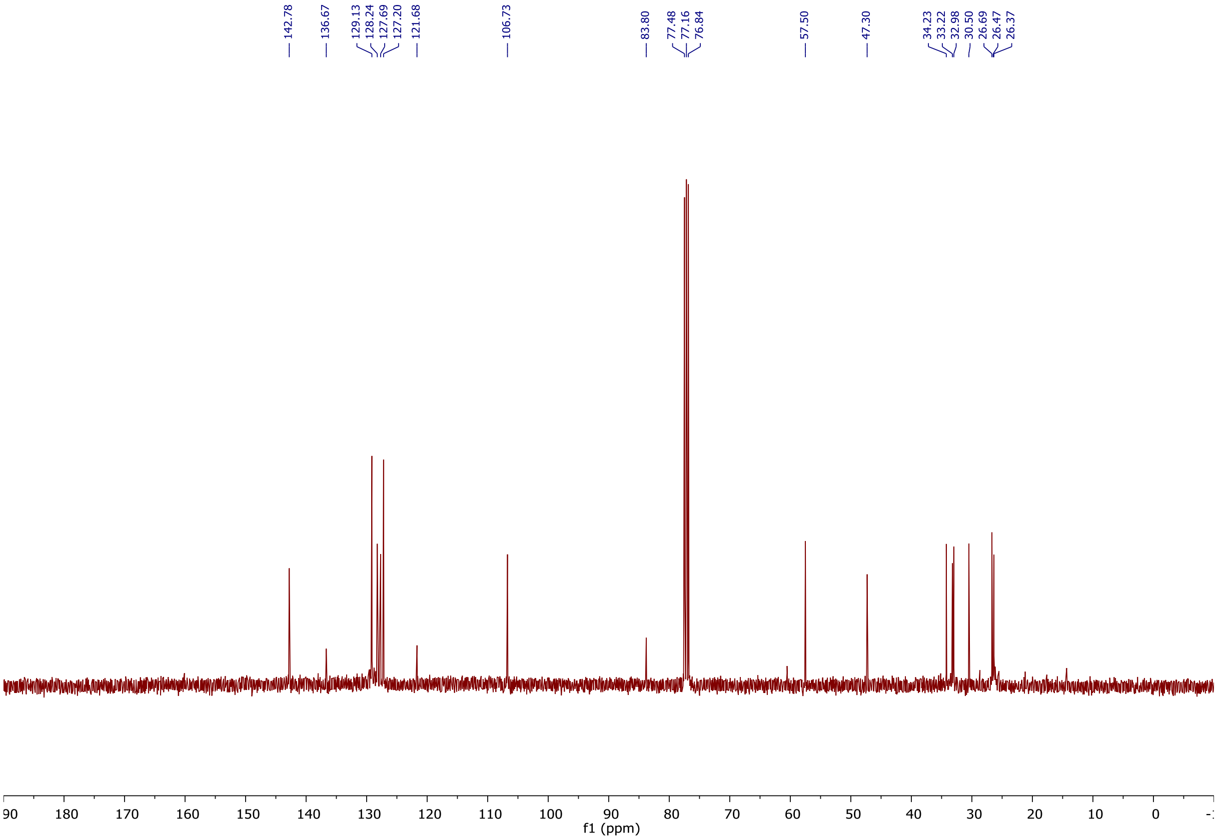

NMR spectra of (*R*)-1-benzyl-4-cyclopentyl-1,4-dihydropyridine-3-carbonitrile (4j)

^{1}H NMR with $CDCl_3$, 400 MHz

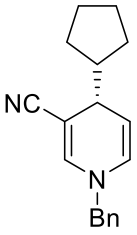

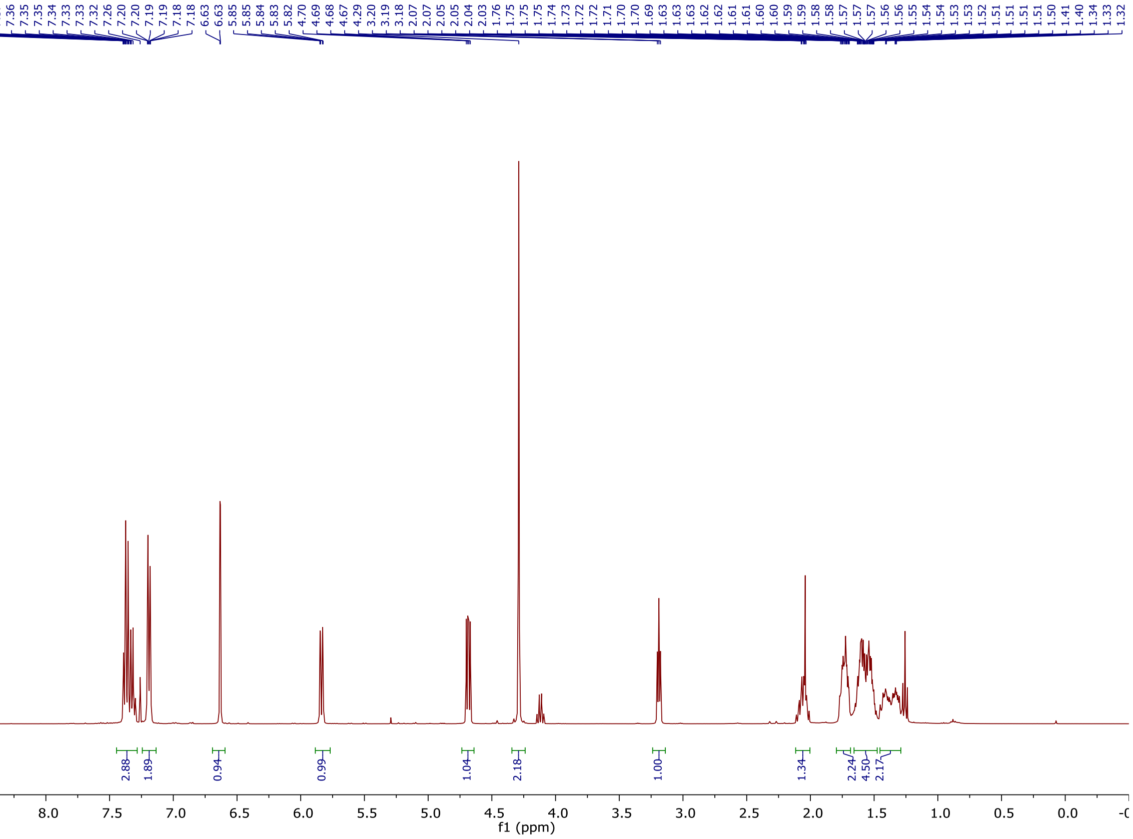

^{13}C NMR with $CDCl_3$, 101 MHz

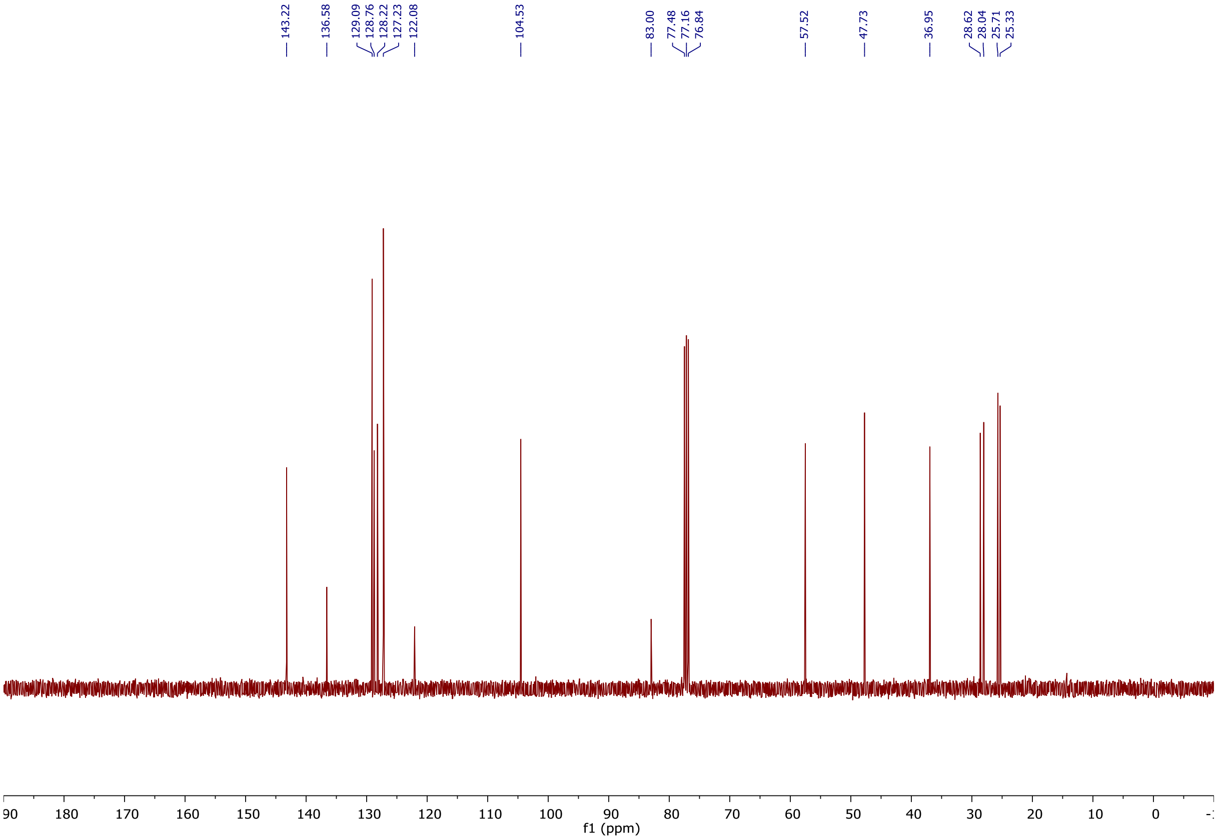

NMR spectra of (*R*)-1-benzyl-4-phenethyl-1,4-dihydropyridine-3-carbonitrile (4k)

^{1}H NMR with $CDCl_3$, 400 MHz

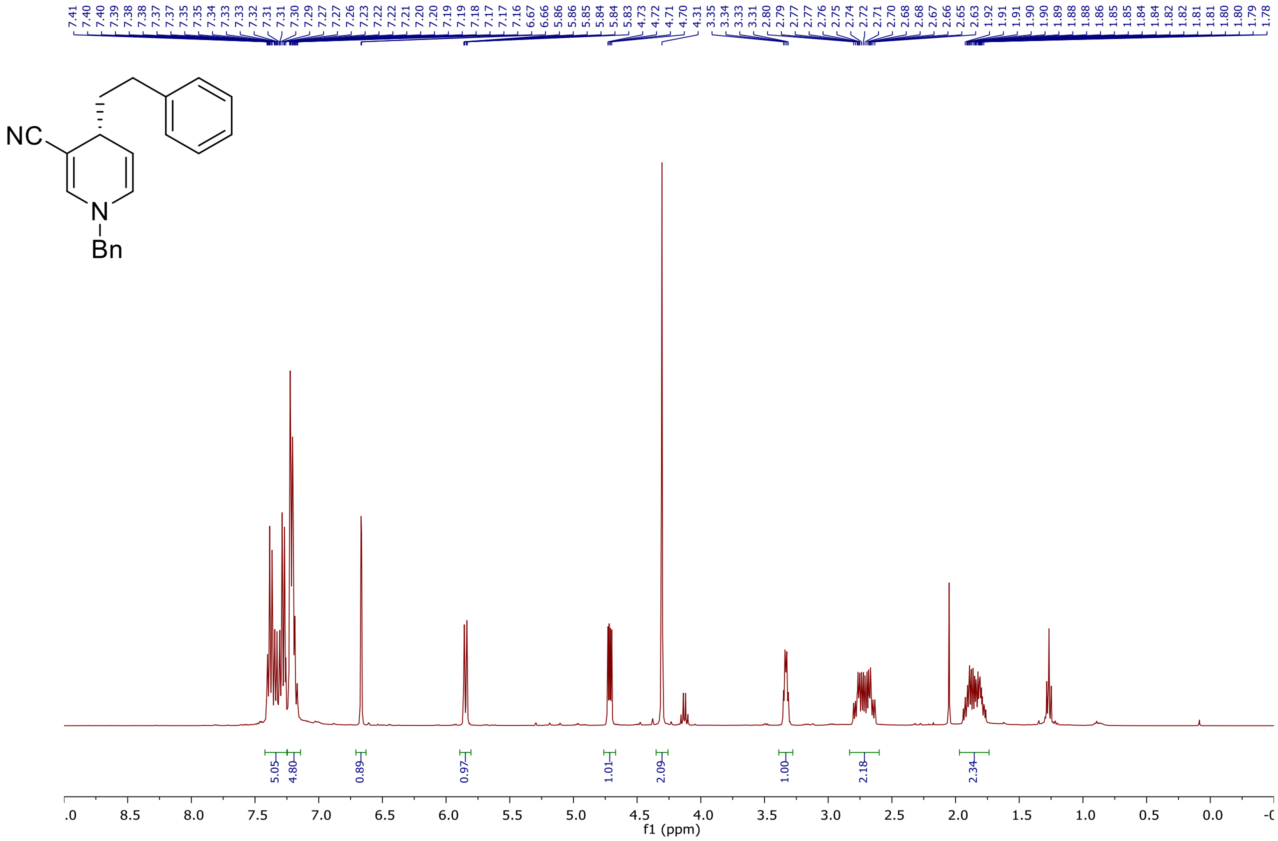

^{13}C NMR with $CDCl_3$, 101 MHz

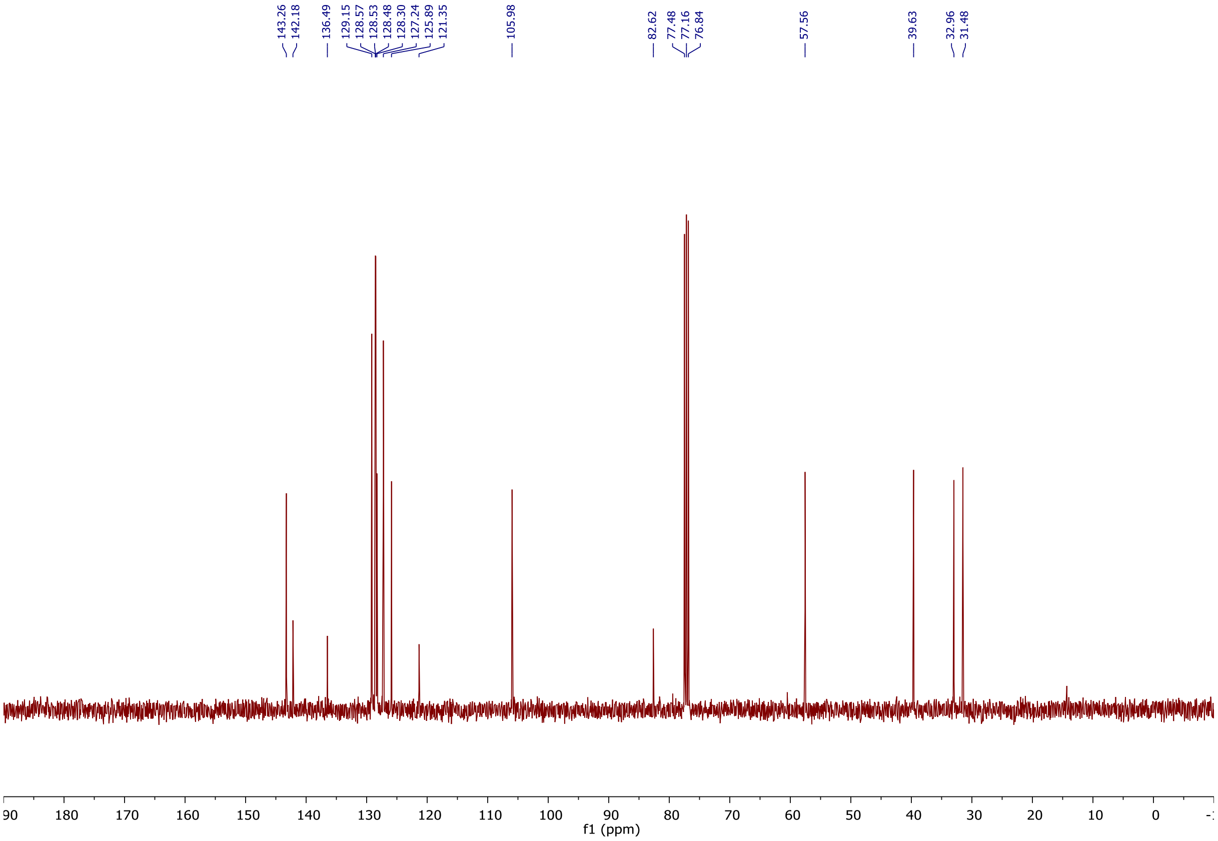

NMR spectra of (*R*)-1-benzyl-4-(3-phenylpropyl)-1,4-dihydropyridine-3-carbonitrile (4l)

^{1}H NMR with $CDCl_3$, 400 MHz

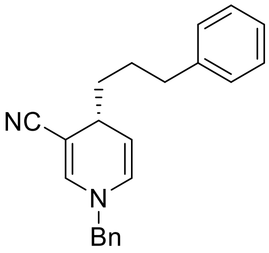

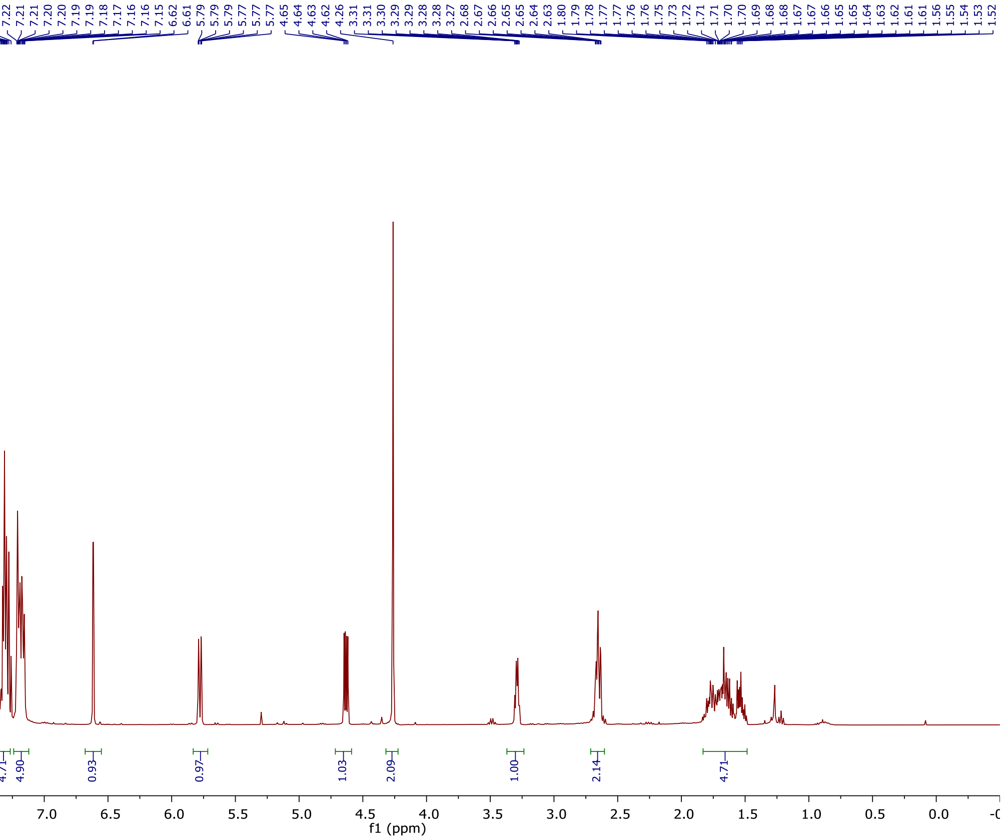

^{13}C NMR with $CDCl_3$, 101 MHz

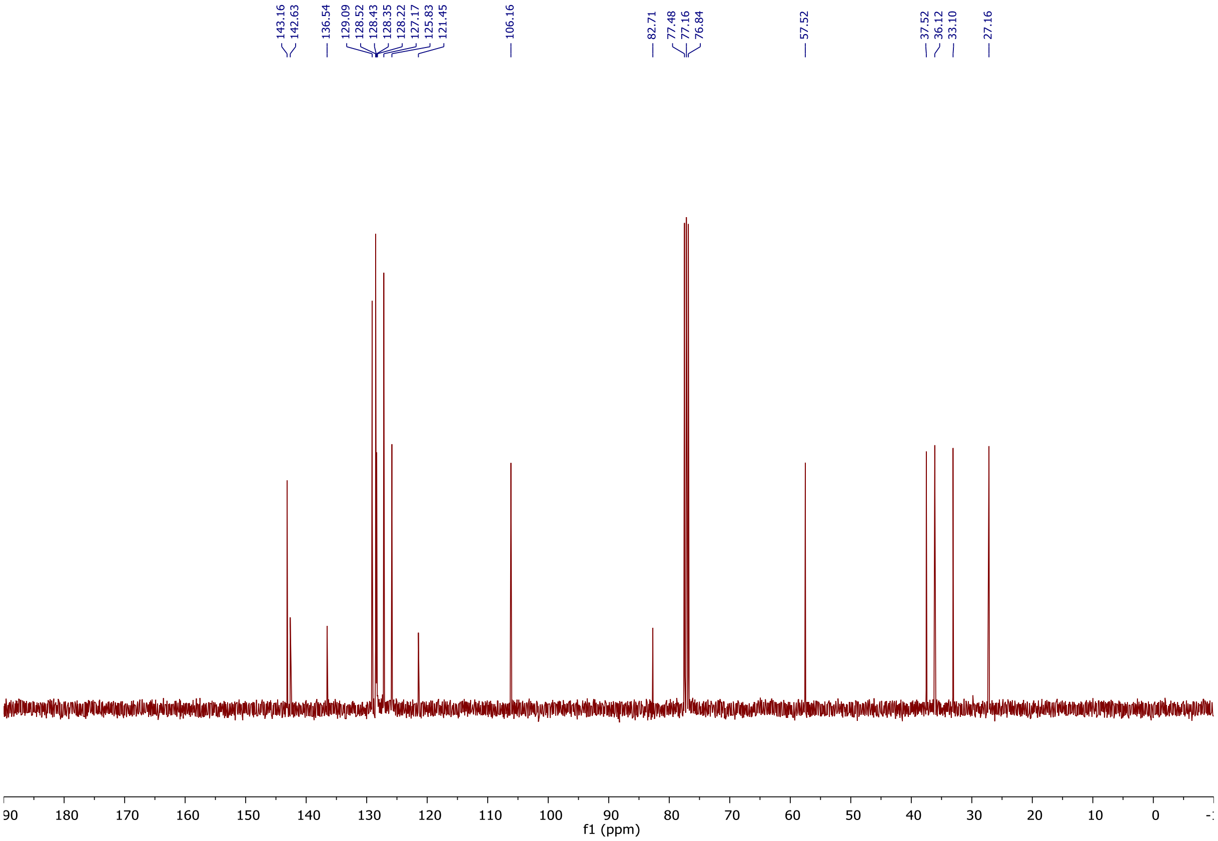

NMR spectra of (*R*)-1-benzyl-4-(but-3-en-1-yl)-1,4-dihydropyridine-3-carbonitrile (4m)

^{1}H NMR with $CDCl_3$, 400 MHz

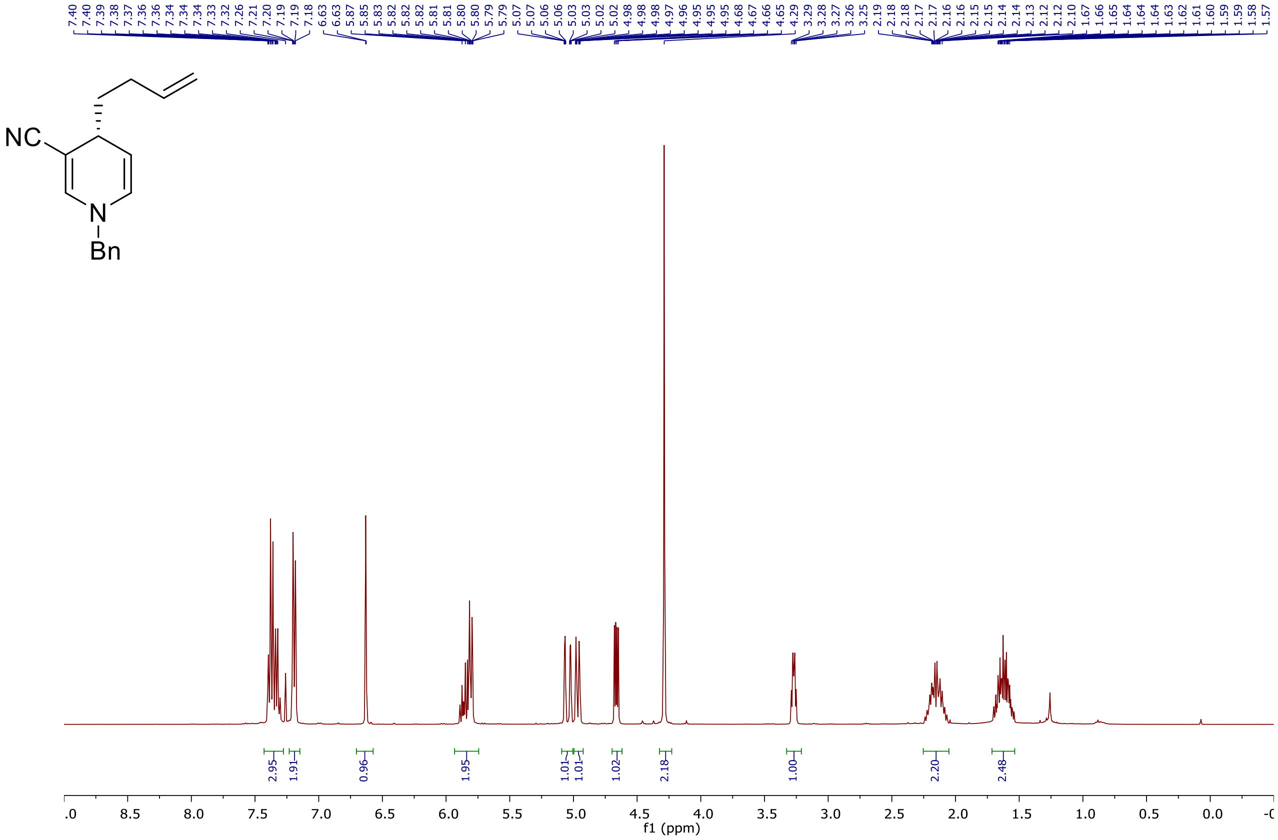

^{13}C NMR with $CDCl_3$, 101 MHz

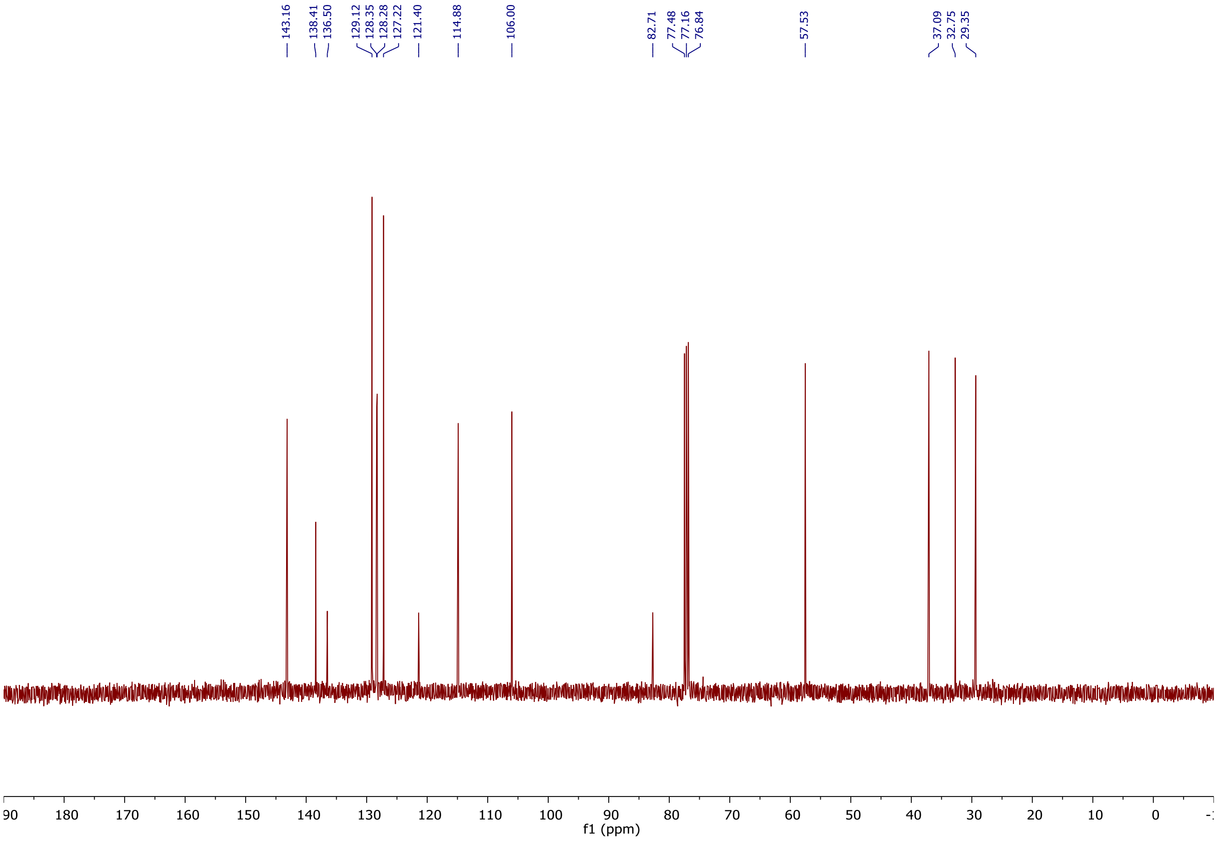

NMR spectra of (*R*)-1-benzyl-4-(hex-5-en-1-yl)-1,4-dihydropyridine-3-carbonitrile (4n)

^{1}H NMR with $CDCl_3$, 400 MHz

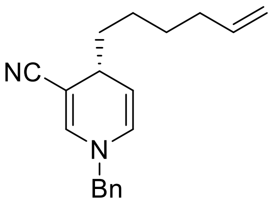

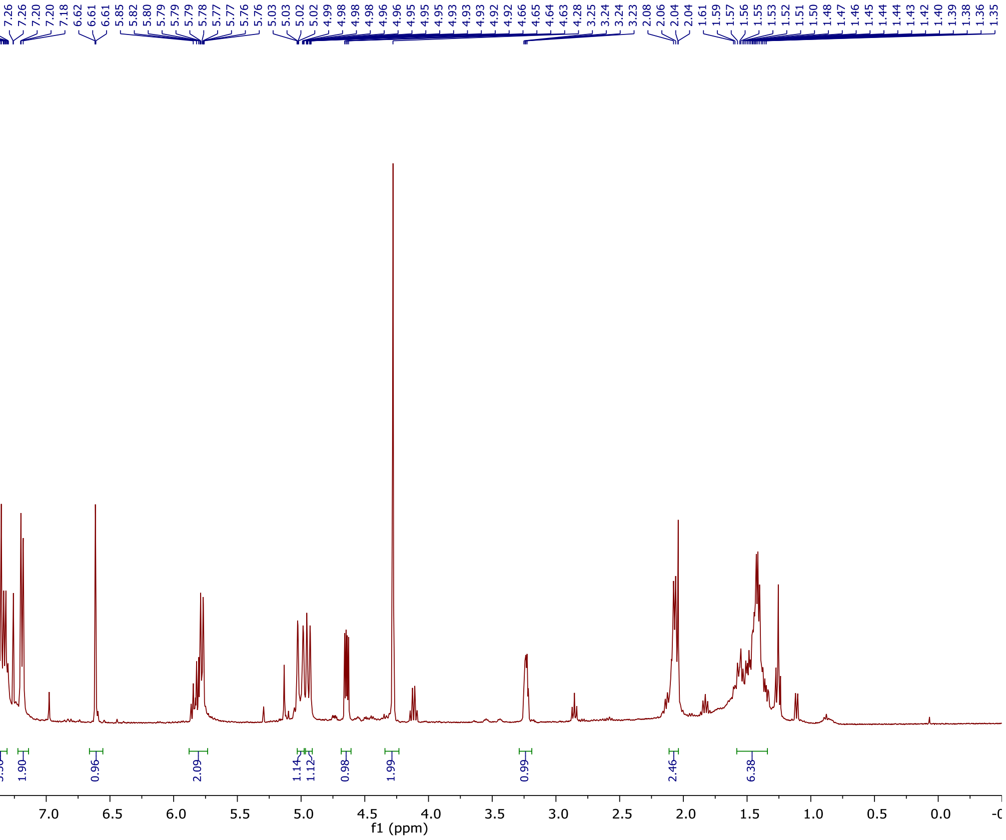

^{13}C NMR with $CDCl_3$, 101 MHz

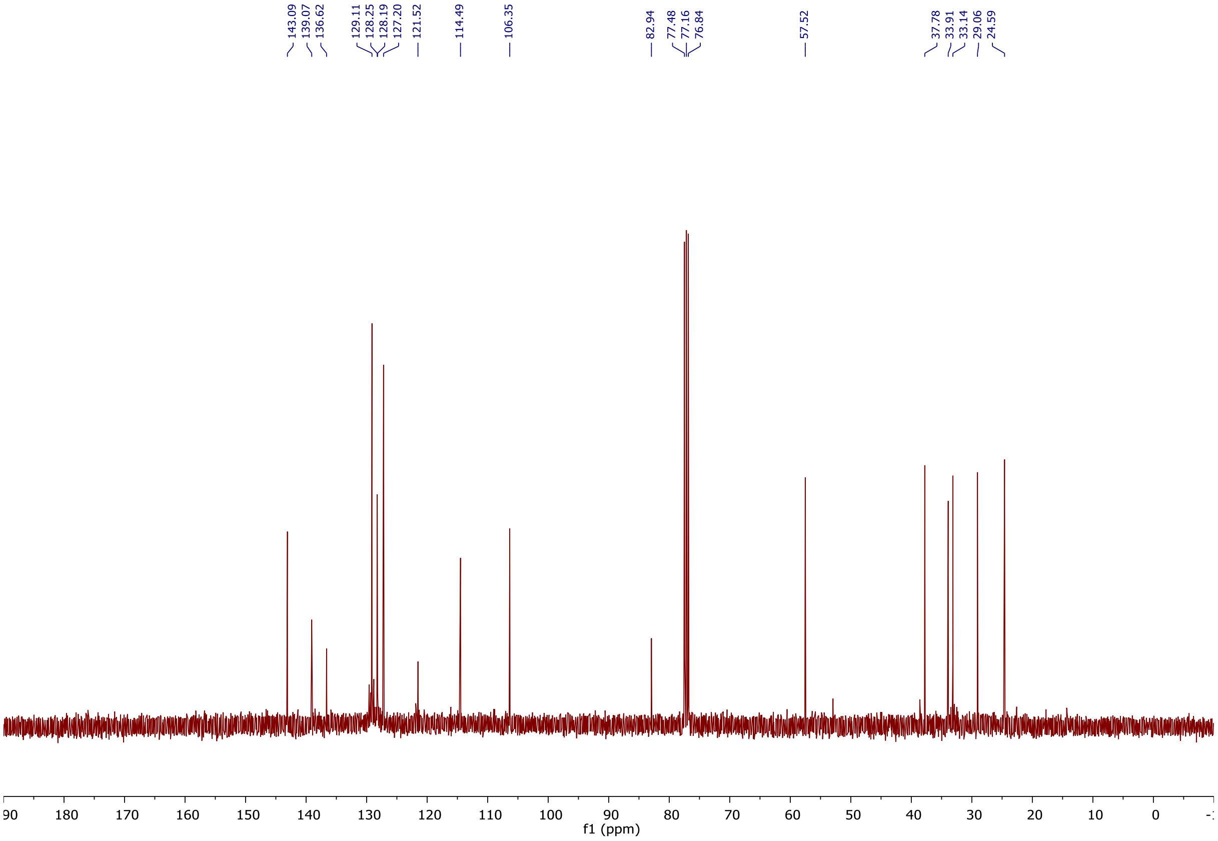

NMR spectra of (*R*)-1-benzyl-4-(4-chlorobutyl)-1,4-dihydropyridine-3-carbonitrile (4o)

^{1}H NMR with $CDCl_3$, 400 MHz

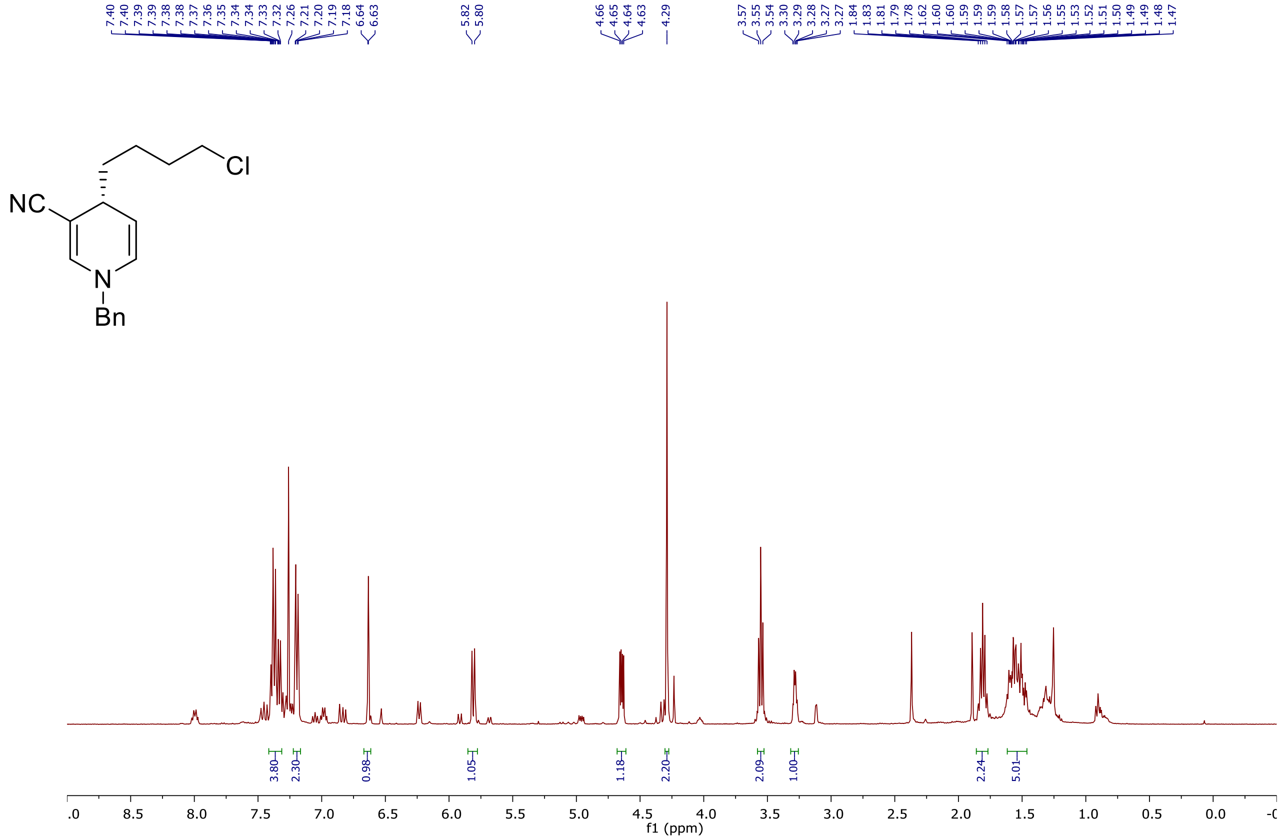

^{13}C NMR with $CDCl_3$, 101 MHz

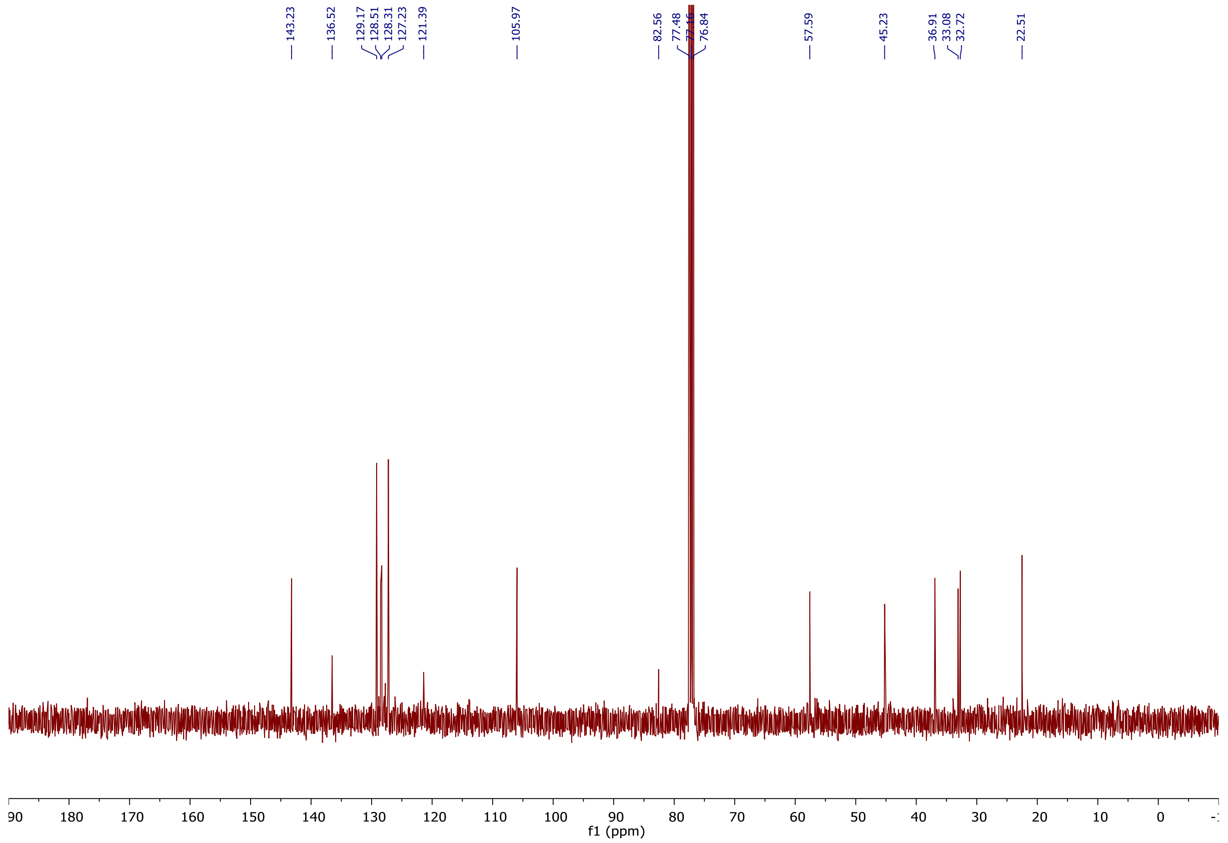

NMR spectra of (*R*)-1-benzyl-4-(6-chlorohexyl)-1,4-dihydropyridine-3-carbonitrile (4p)

^{1}H NMR with $CDCl_3$, 400 MHz

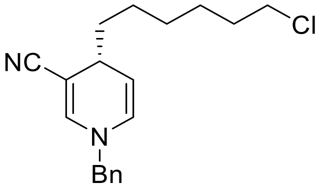

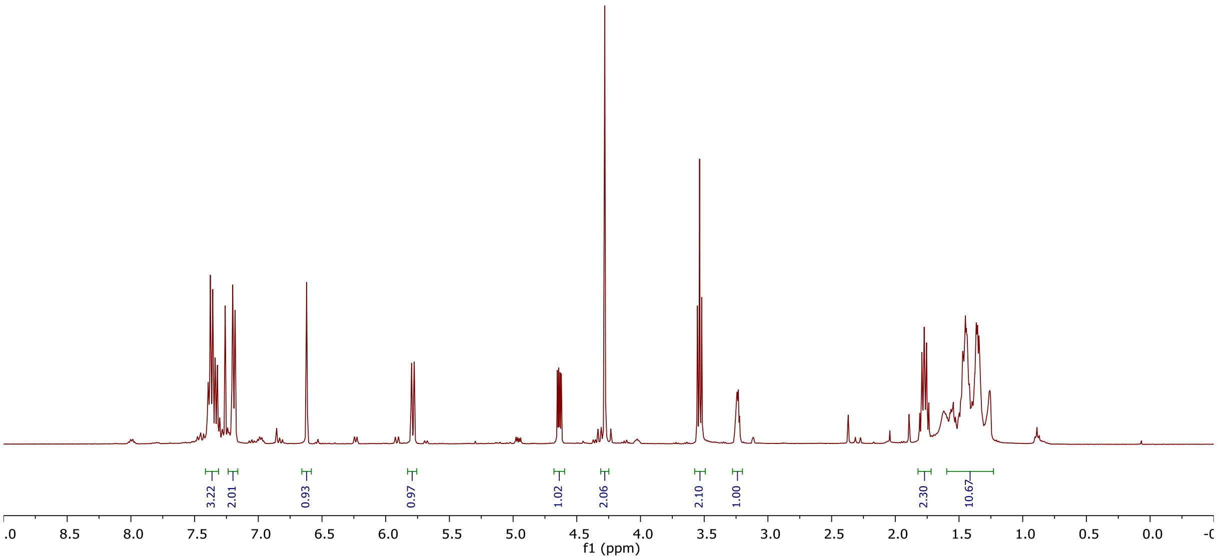

^{13}C NMR with $CDCl_3$, 101 MHz

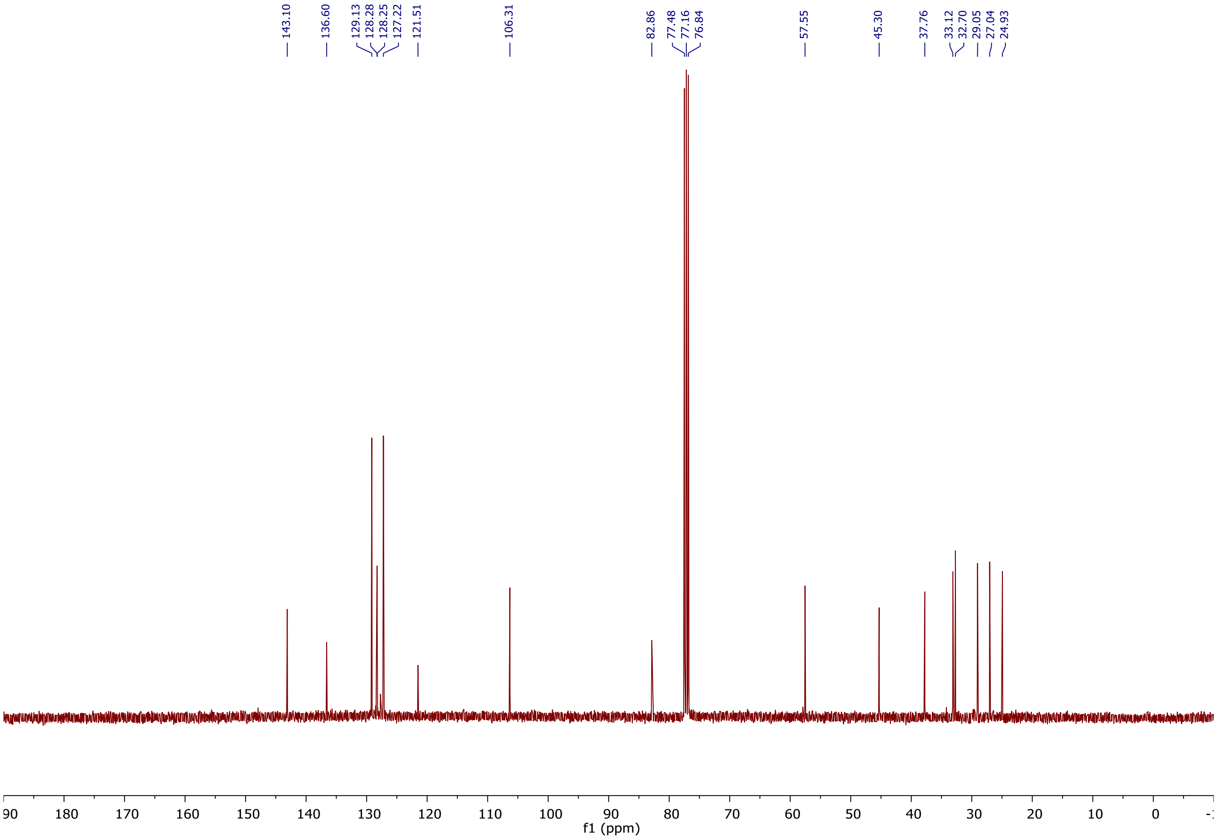

NMR spectra of (*rac*)-1-benzyl-4-isopropyl-1,4-dihydropyridine-3-carbonitrile (4q)

^{1}H NMR with $CDCl_3$, 400 MHz

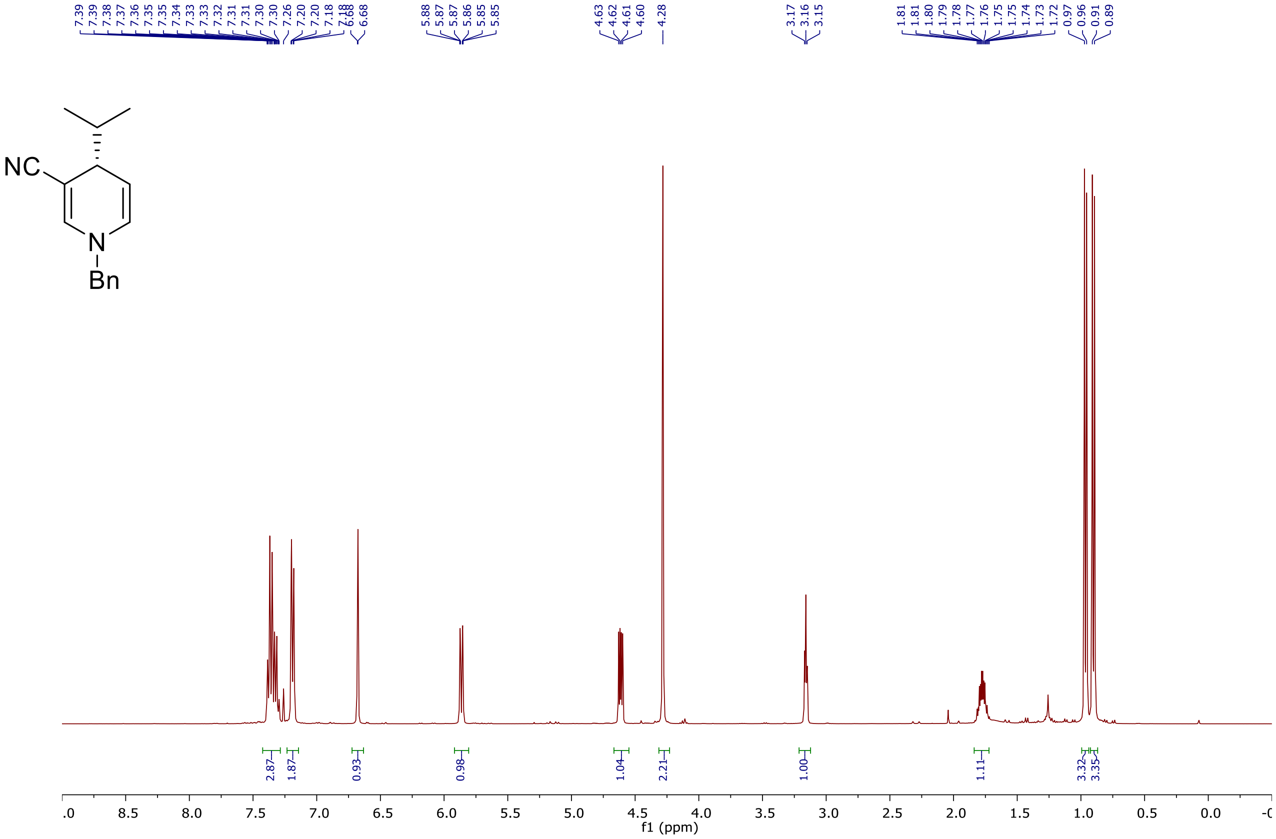

^{13}C NMR with $CDCl_3$, 101 MHz

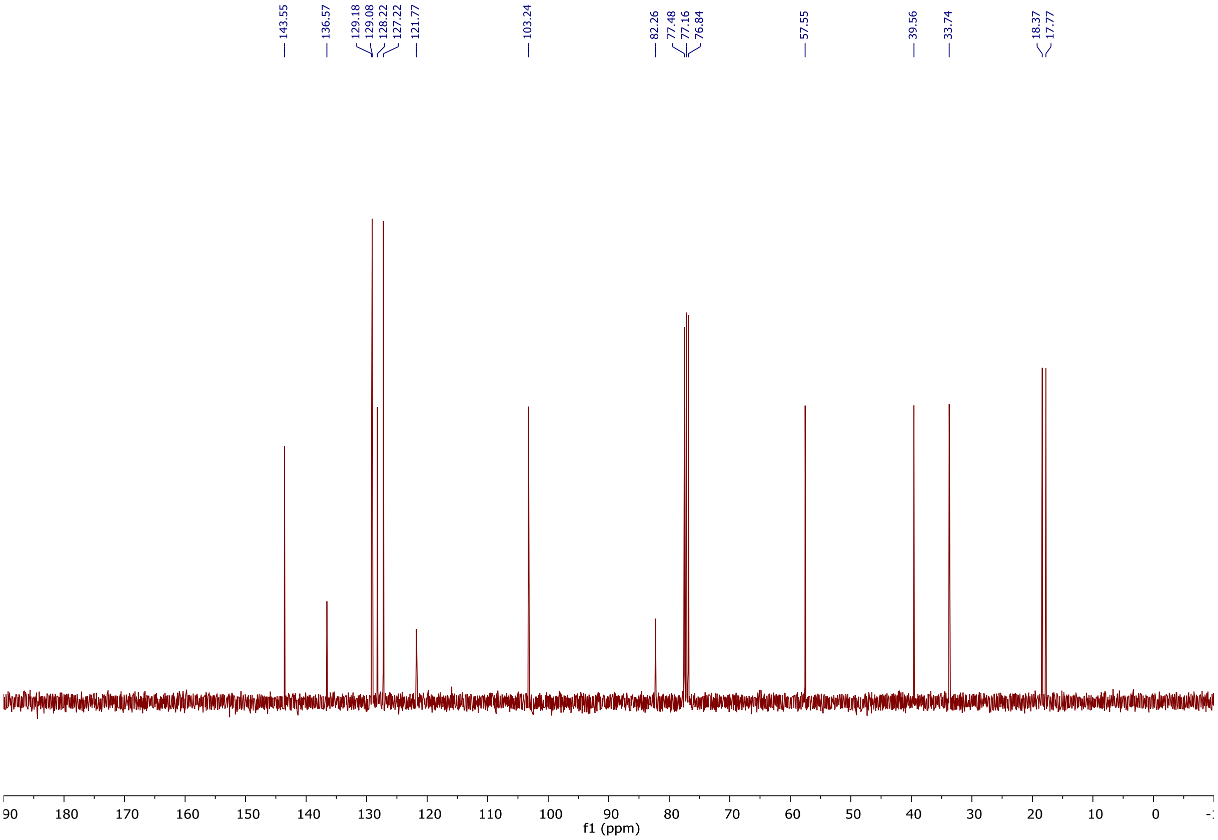

NMR spectra of (*rac*)-4-allyl-1-benzyl-1,4-dihydropyridine-3-carbonitrile (4r)

^{1}H NMR with $CDCl_3$, 400 MHz

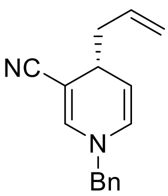

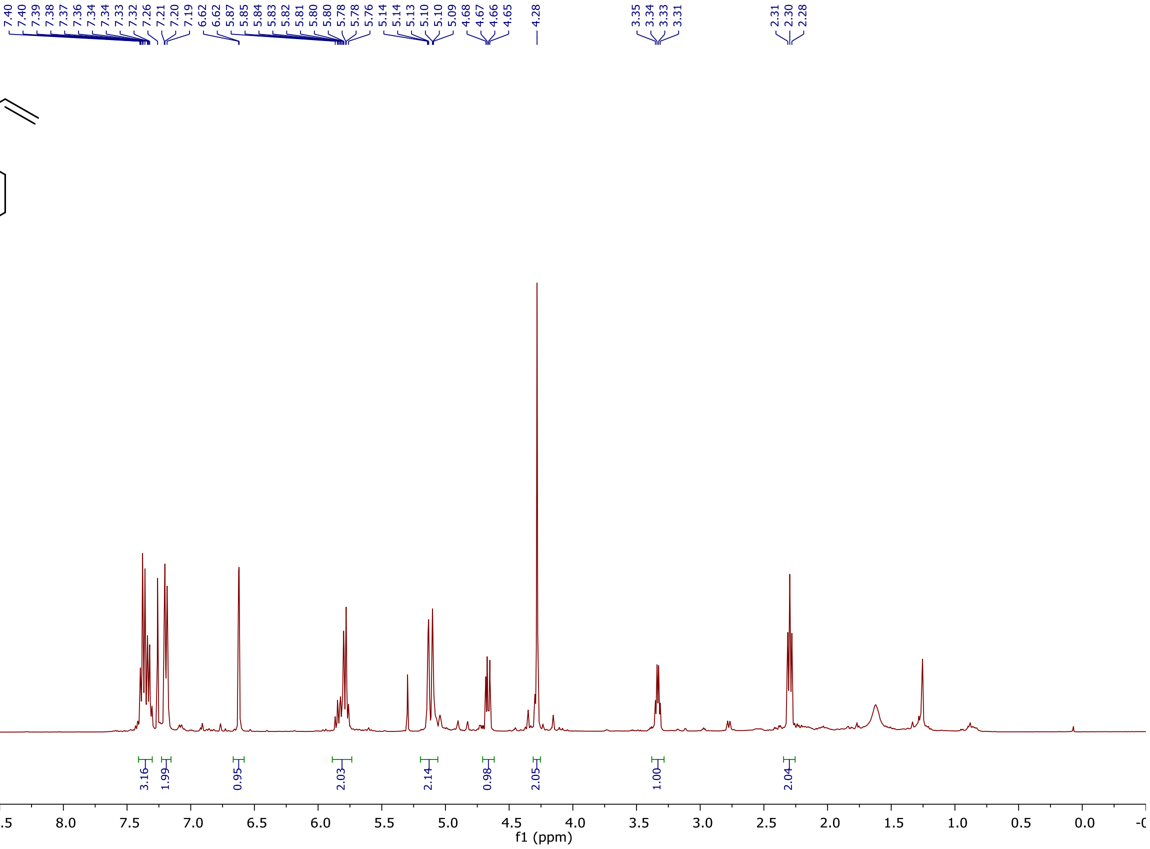

^{13}C NMR with $CDCl_3$, 101 MHz

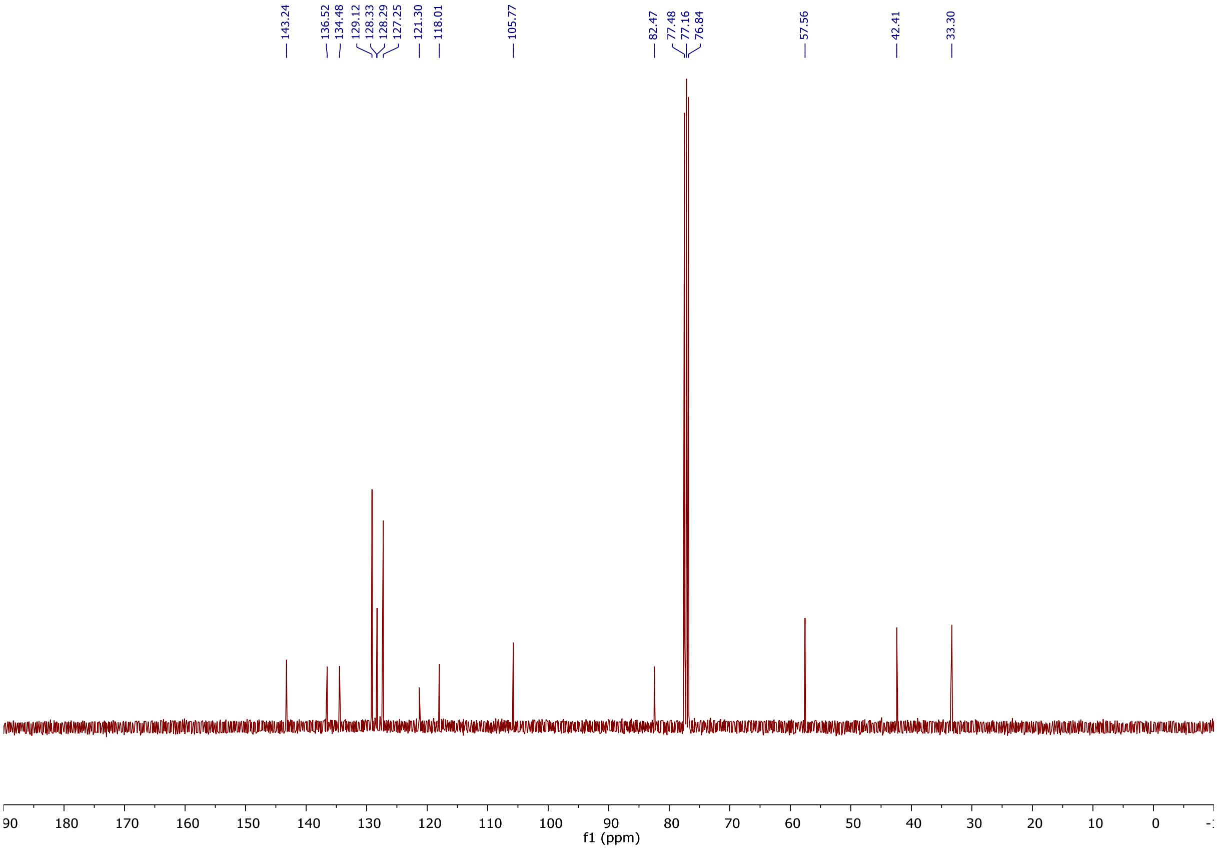

NMR spectra of (*rac*)-1-benzyl-4-phenyl-1,4-dihydropyridine-3-carbonitrile (4s)

^{1}H NMR with $CDCl_3$, 400 MHz

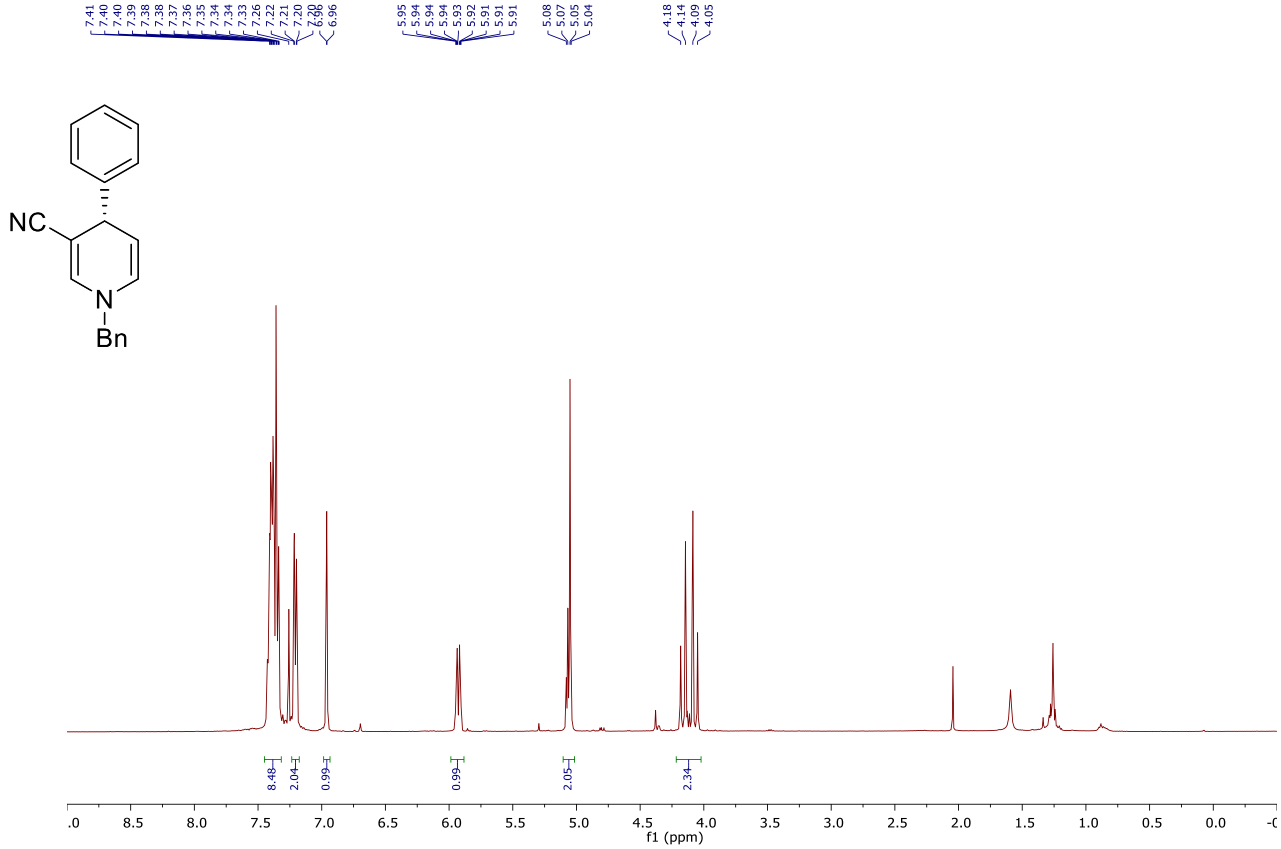

^{13}C NMR with $CDCl_3$, 101 MHz

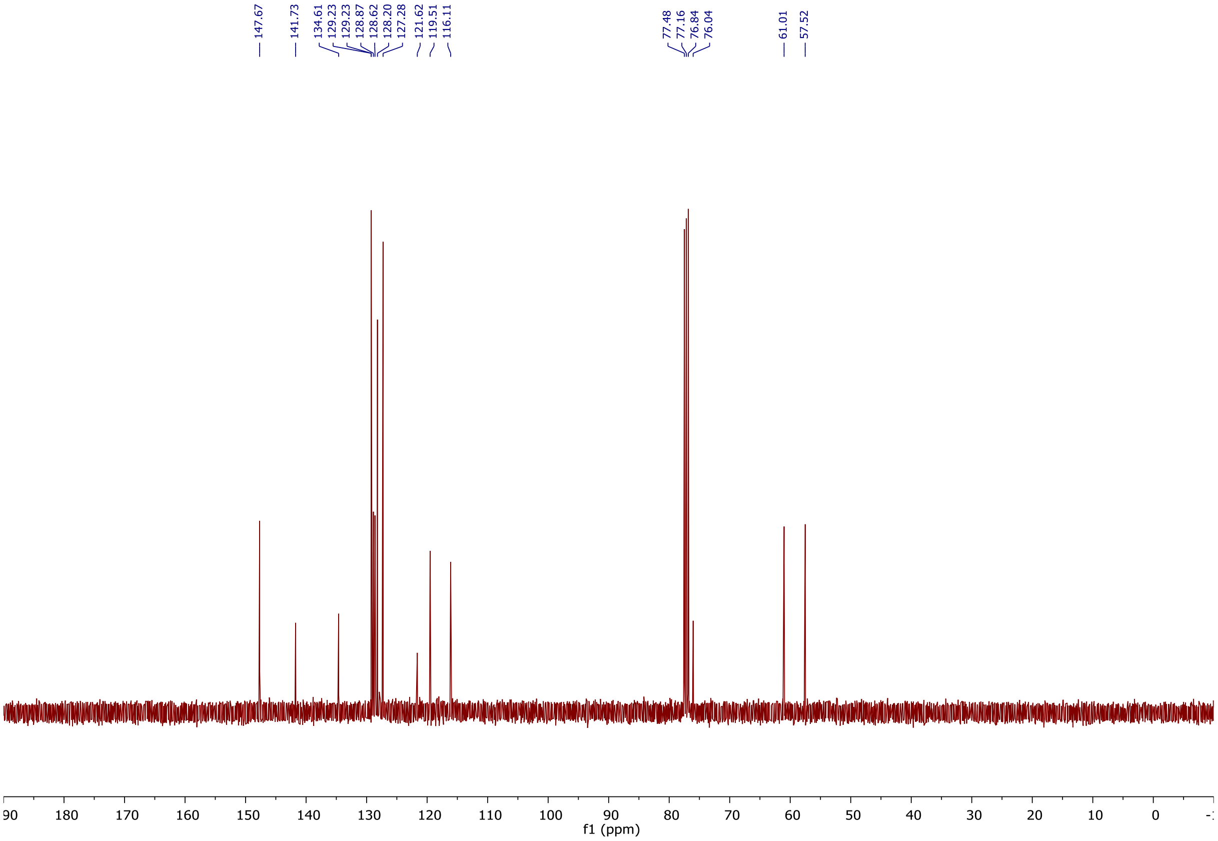

NMR spectra of (*rac*)-1,4-dibenzyl-1,4-dihydropyridine-3-carbonitrile (4t)

^{1}H NMR with $CDCl_3$, 400 MHz

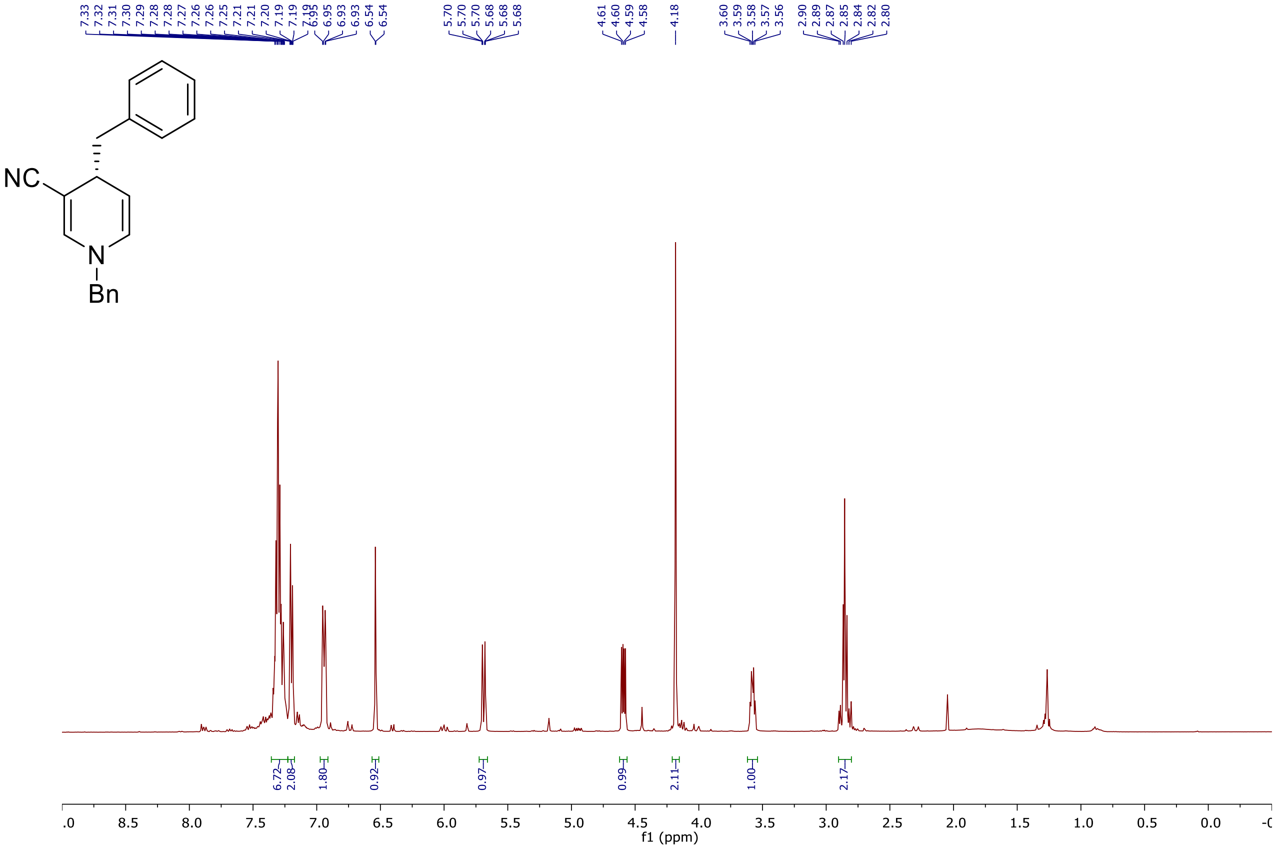

^{13}C NMR with $CDCl_3$, 101 MHz

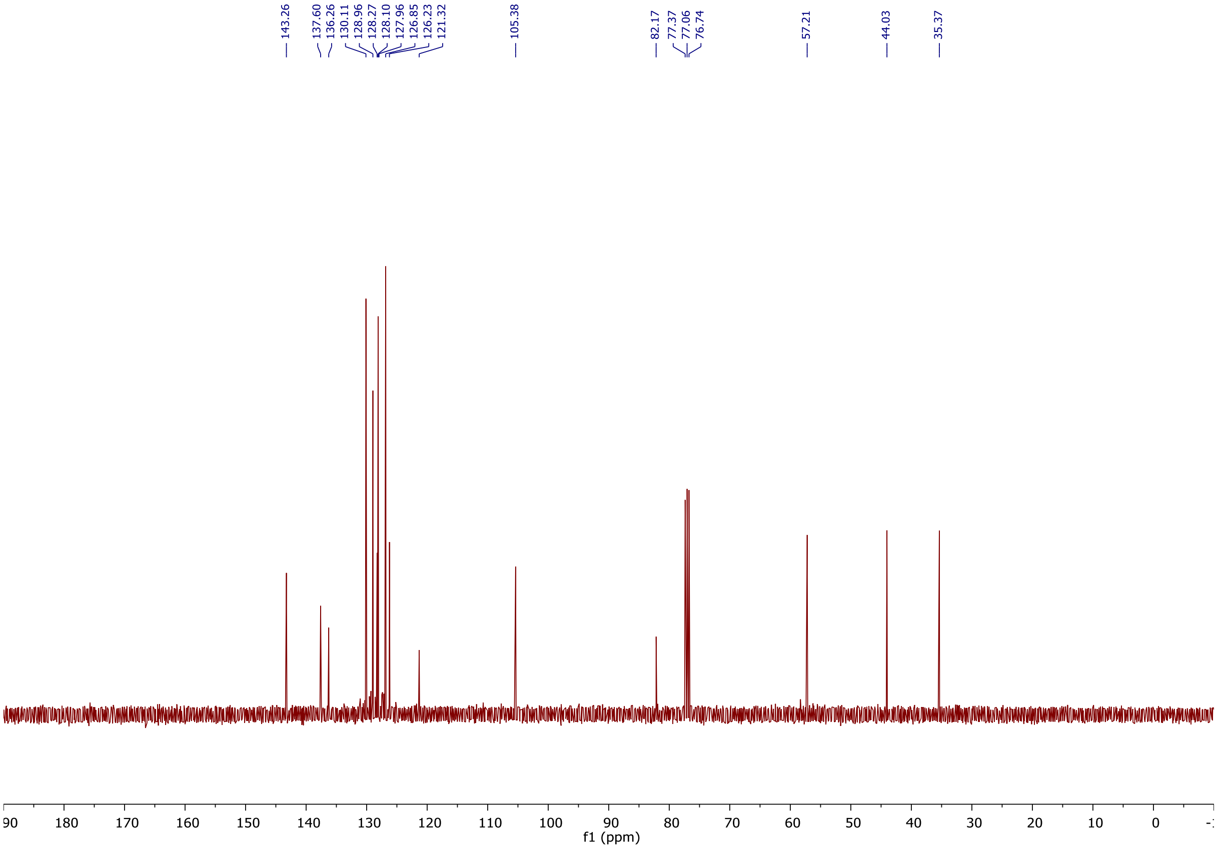

NMR spectra of (*R*)-1-benzyl-4-methyl-1,4-dihydropyridine-3-carbonitrile (4u)

^{1}H NMR with $CDCl_3$, 600 MHz

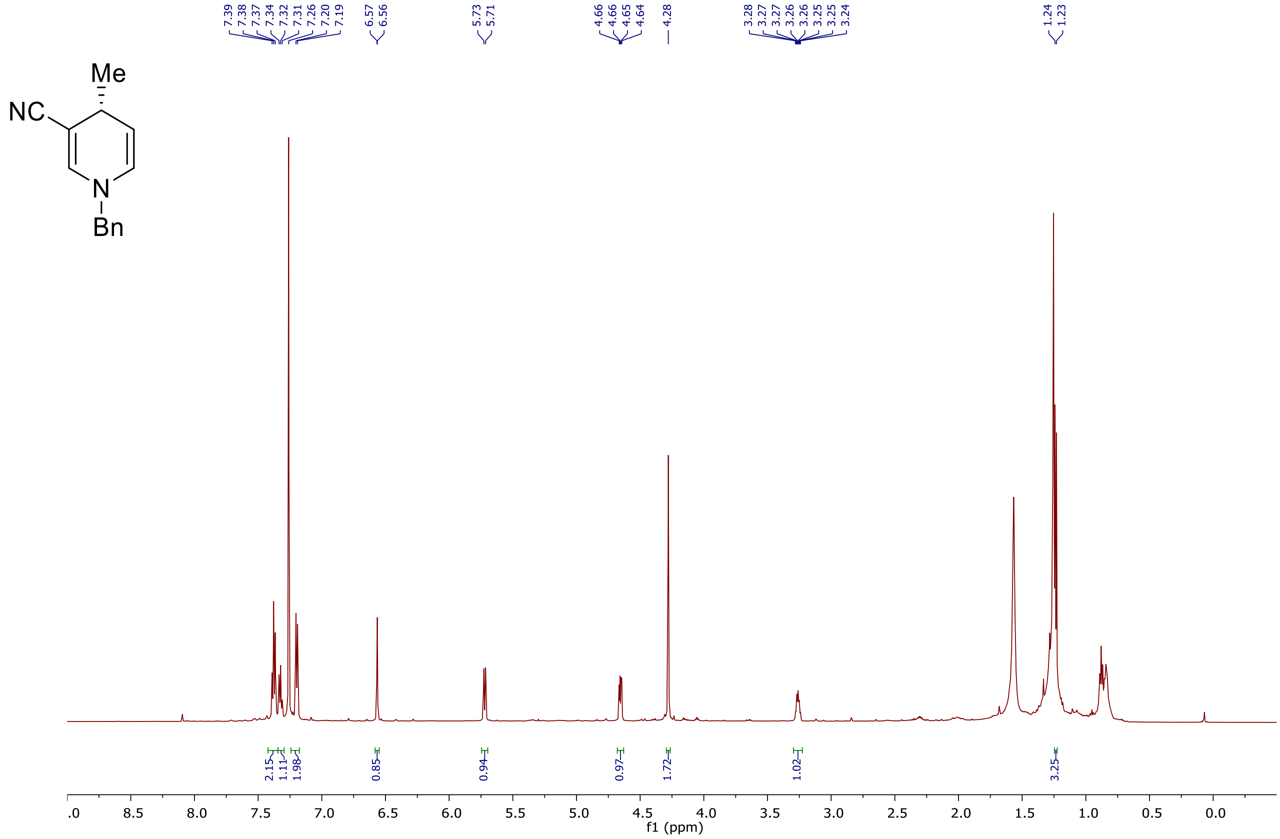

^{13}C NMR with $CDCl_3$, 151 MHz

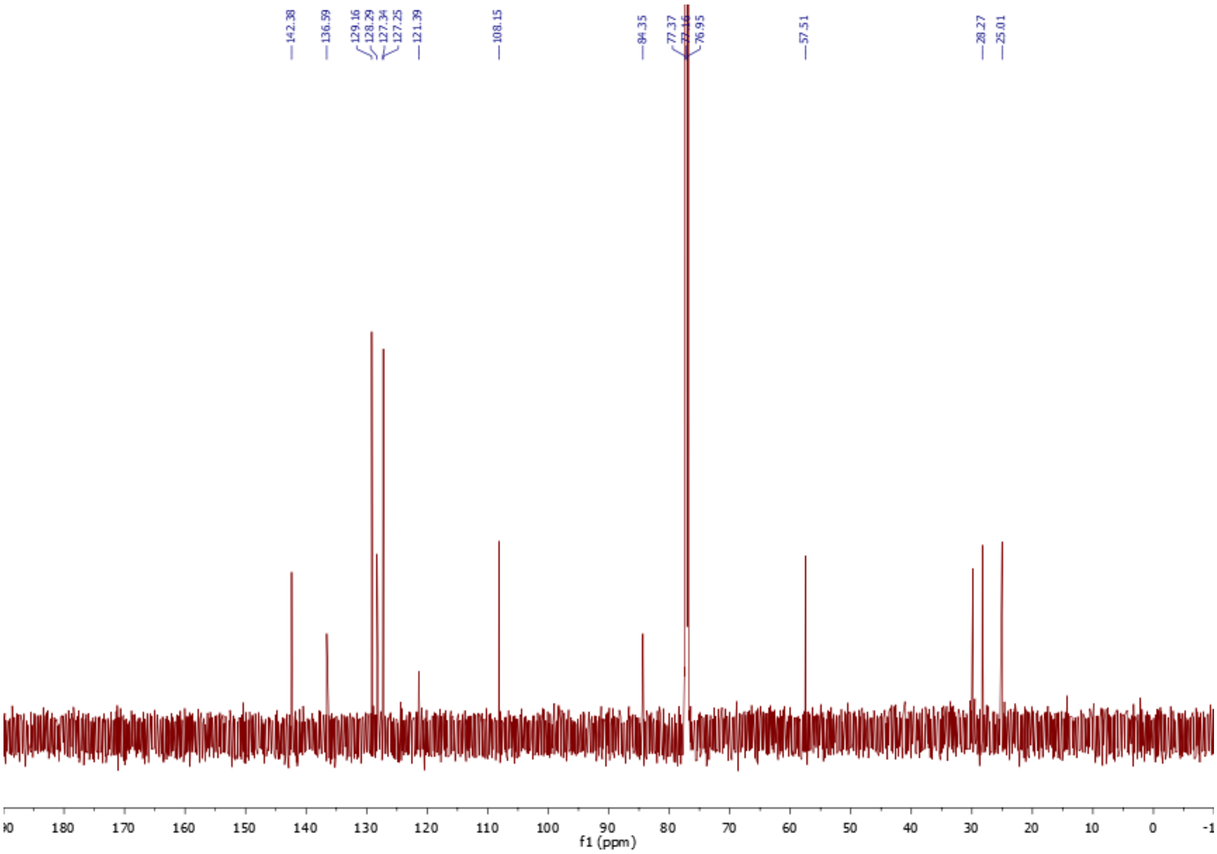

NMR spectra of (*R*)-1-benzyl-4-ethyl-6-methyl-1,4-dihydropyridine-3-carbonitrile (5a)

^{1}H NMR with $CDCl_3$, 400 MHz

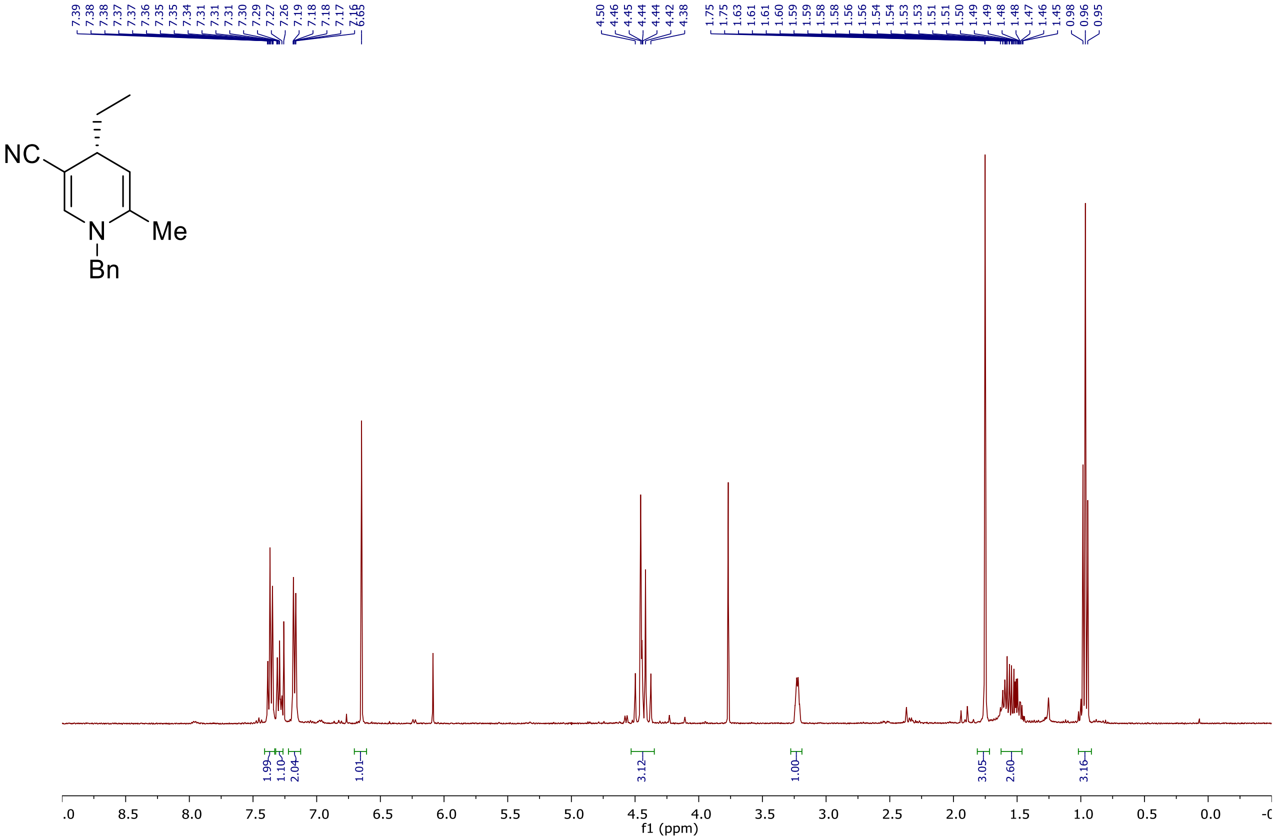

^{13}C NMR with $CDCl_3$, 101 MHz

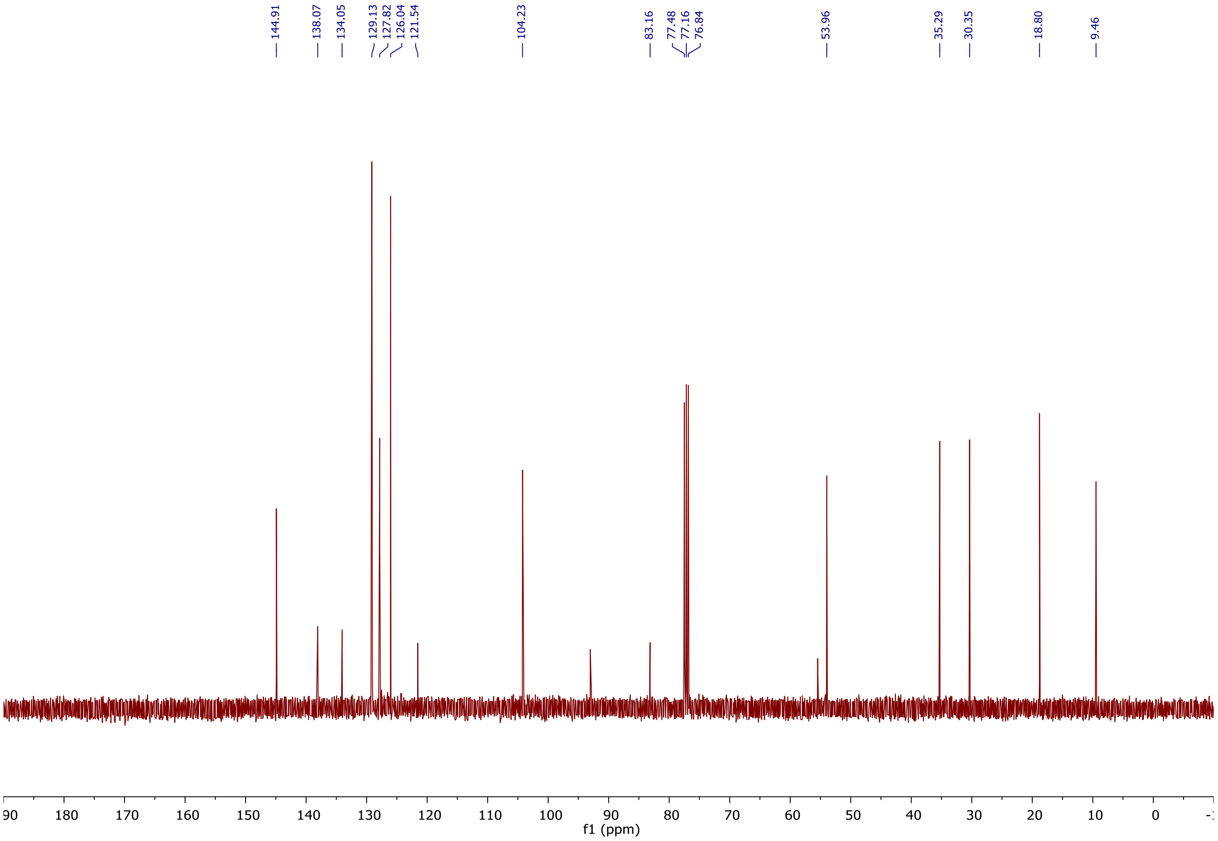

NMR spectra of (*R*)-1-benzyl-4-hexyl-6-methyl-1,4-dihydropyridine-3-carbonitrile (5b)

^{1}H NMR with $CDCl_3$, 400 MHz

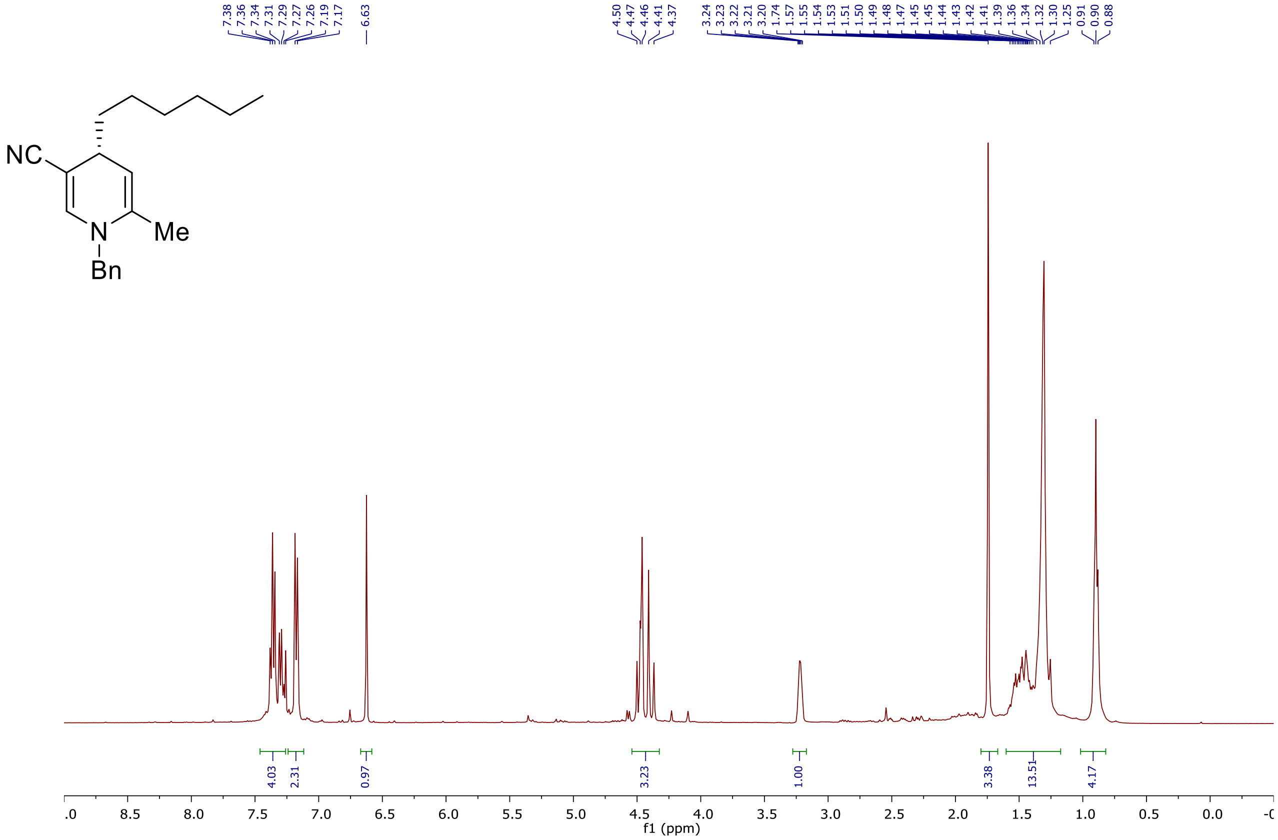

^{13}C NMR with $CDCl_3$, 101 MHz

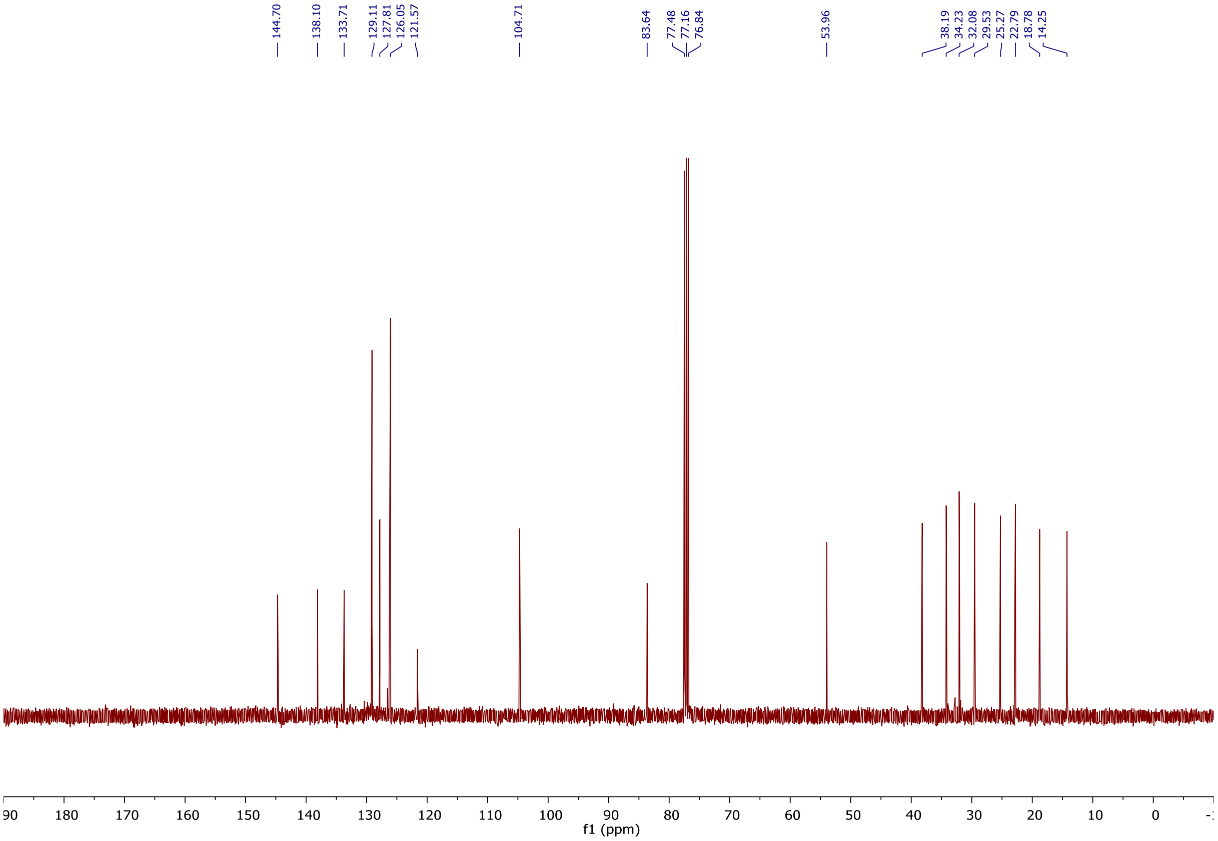

NMR spectra of methyl (*R*)-1-benzyl-4-hexyl-1,4-dihydropyridine-3-carboxylate (5c)

^{1}H NMR with $CDCl_3$, 400 MHz

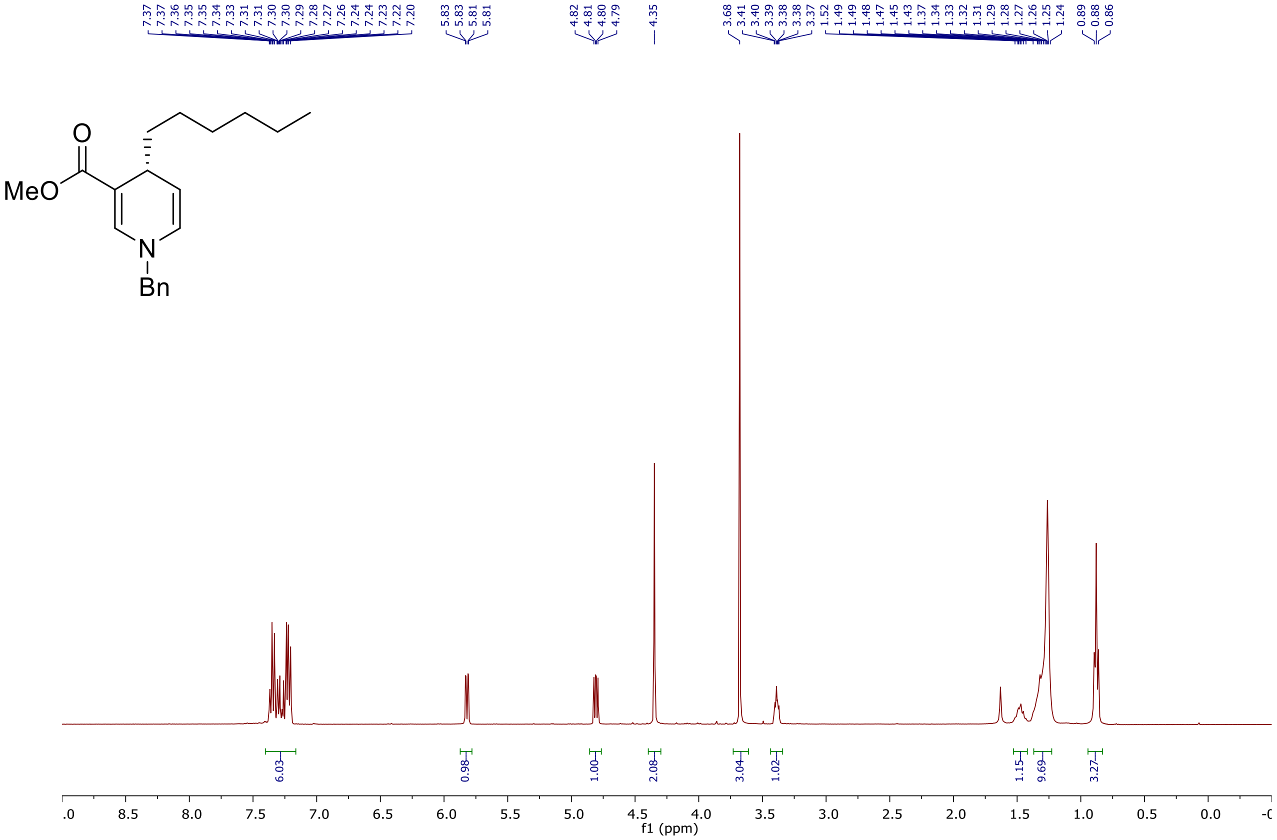

^{13}C NMR with $CDCl_3$, 101 MHz

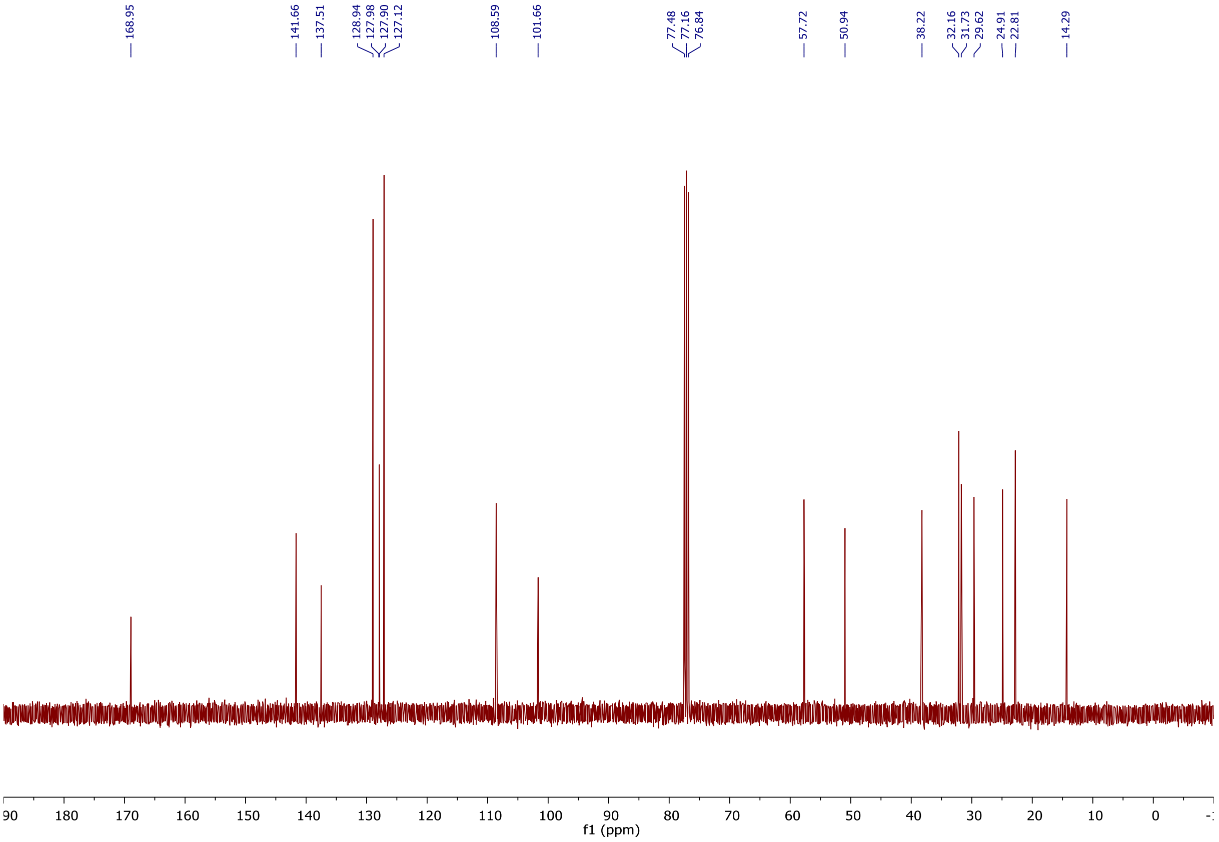

NMR spectra of (*R*)-1-benzyl-4-hexyl-1,4-dihydroquinoline-3-carbonitrile (5d)

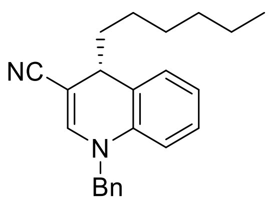

^{1}H NMR with $CDCl_3$, 400 MHz

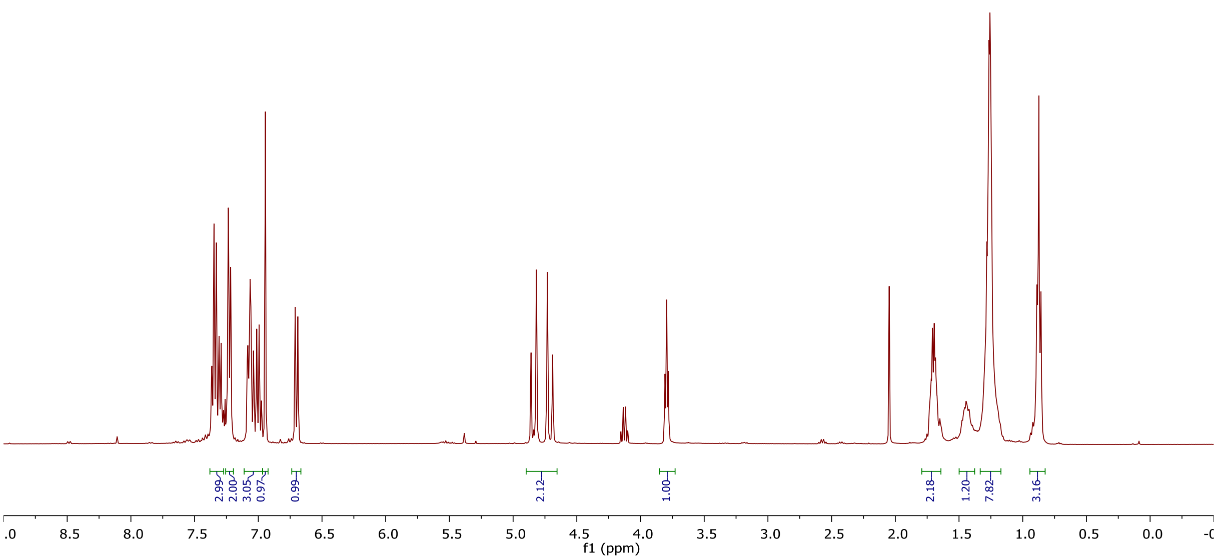

^{13}C NMR with $CDCl_3$, 101 MHz

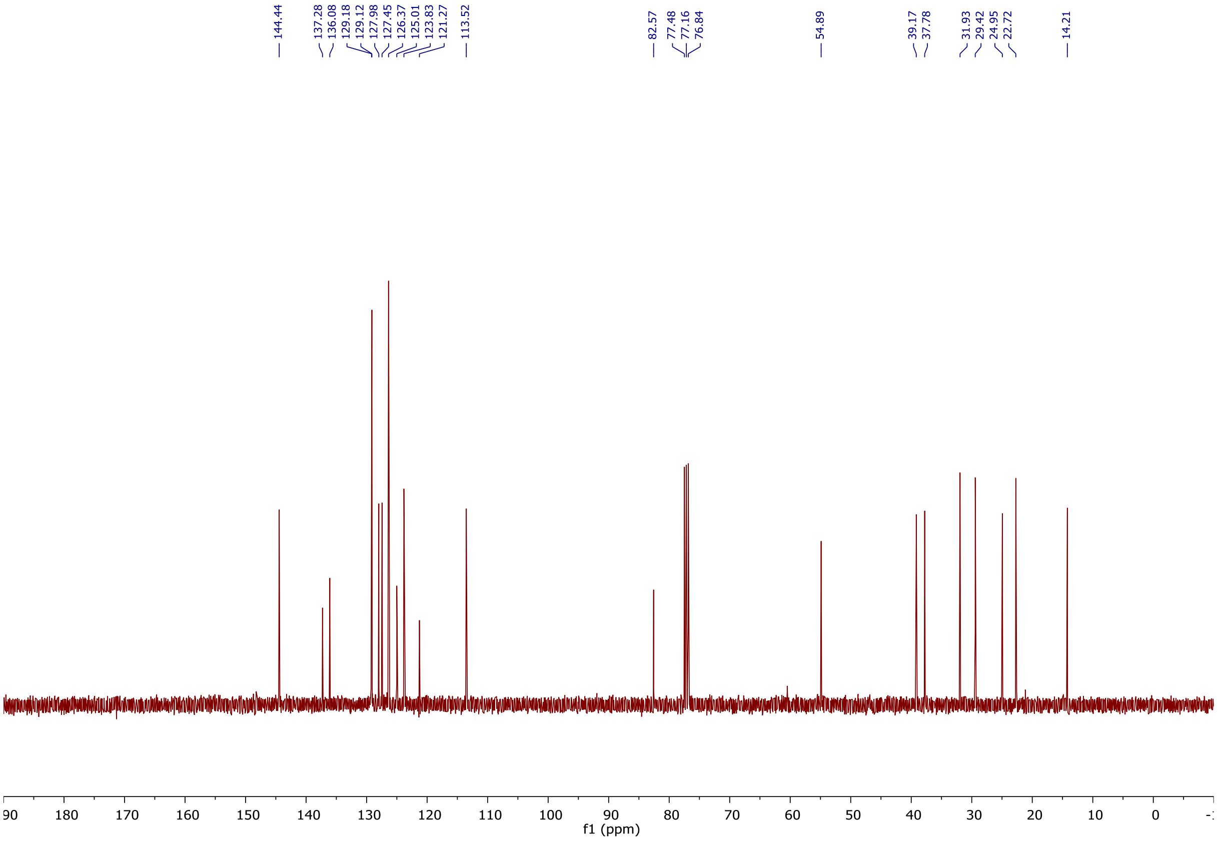

NMR spectra of (4*S*,5*R*)-*N*-Benzyl-5-fluoro-4-hexyl-1,4,5,6-tetrahydropyridine-3-carbonitrile (6)

^{1}H NMR with $CDCl_3$, 600 MHz

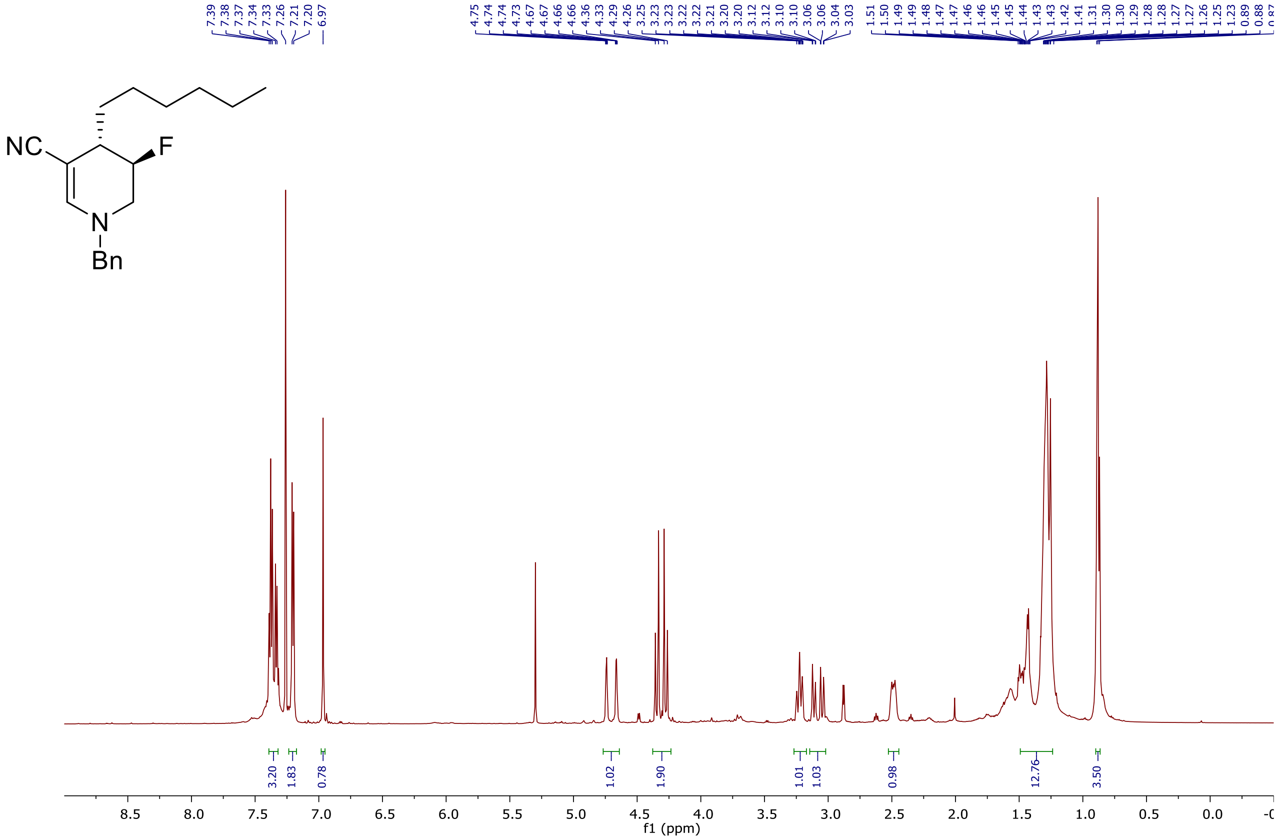

^{13}C NMR with $CDCl_3$, 151 MHz

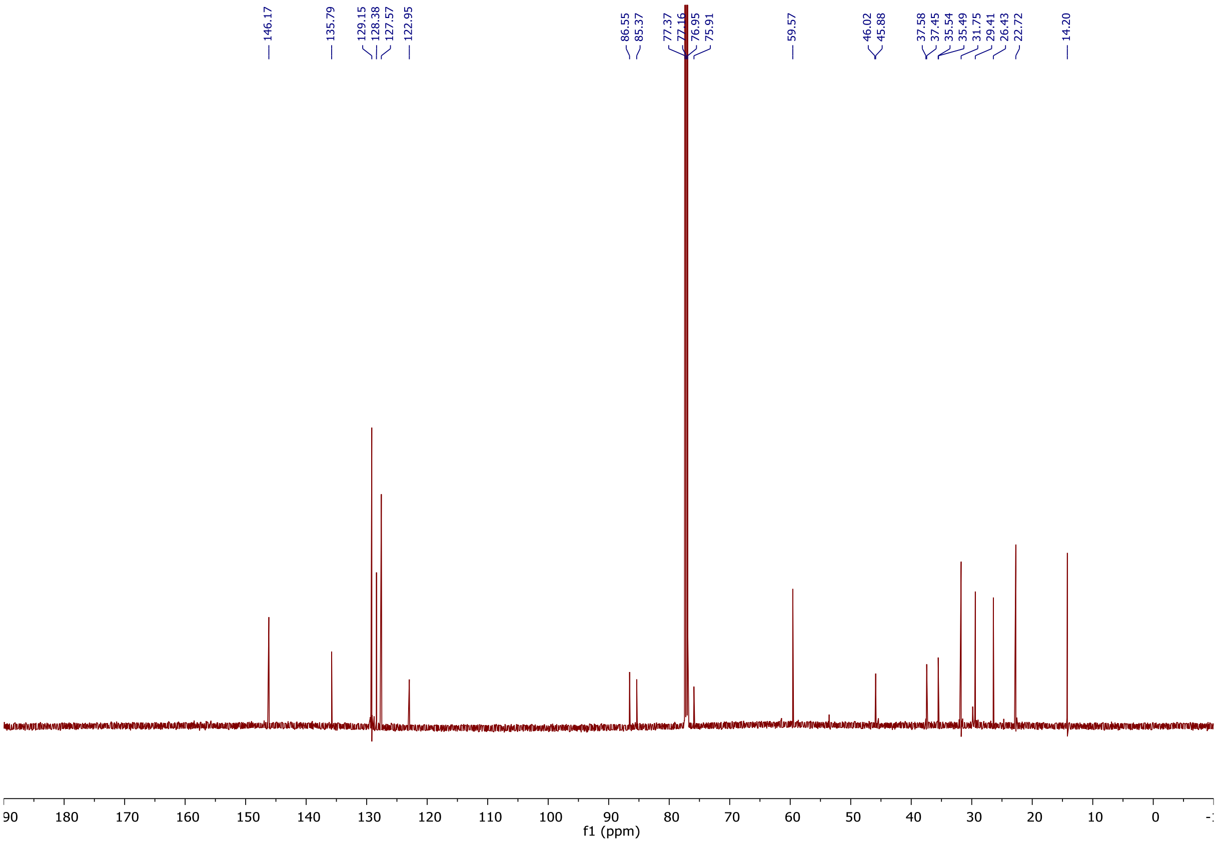

^{19}F NMR with $CDCl_3$, 565 MHz

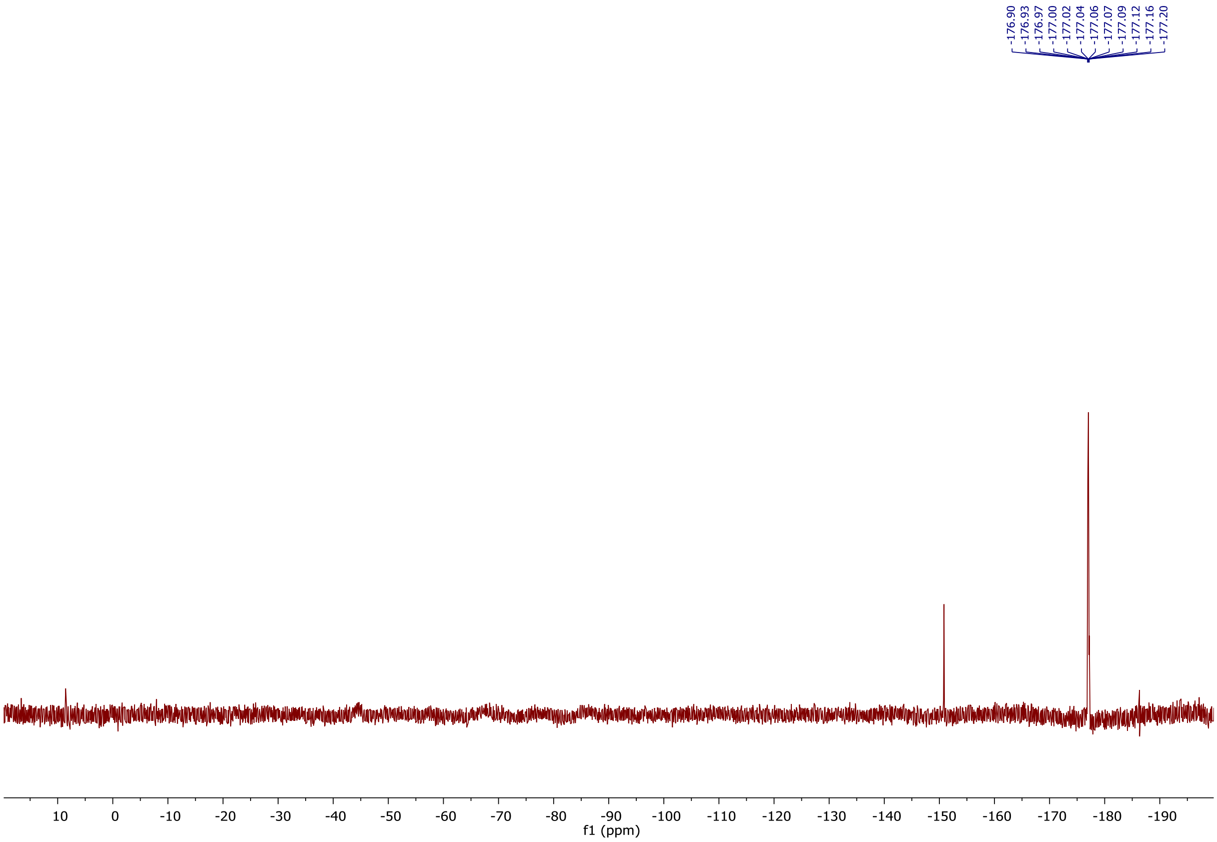

NMR spectra of (*R*)-*N*-benzyl-4-hexyl-1,4,5,6-tetrahydropyridine-3-carbonitrile (7)

^{1}H NMR with $CDCl_3$, 400 MHz

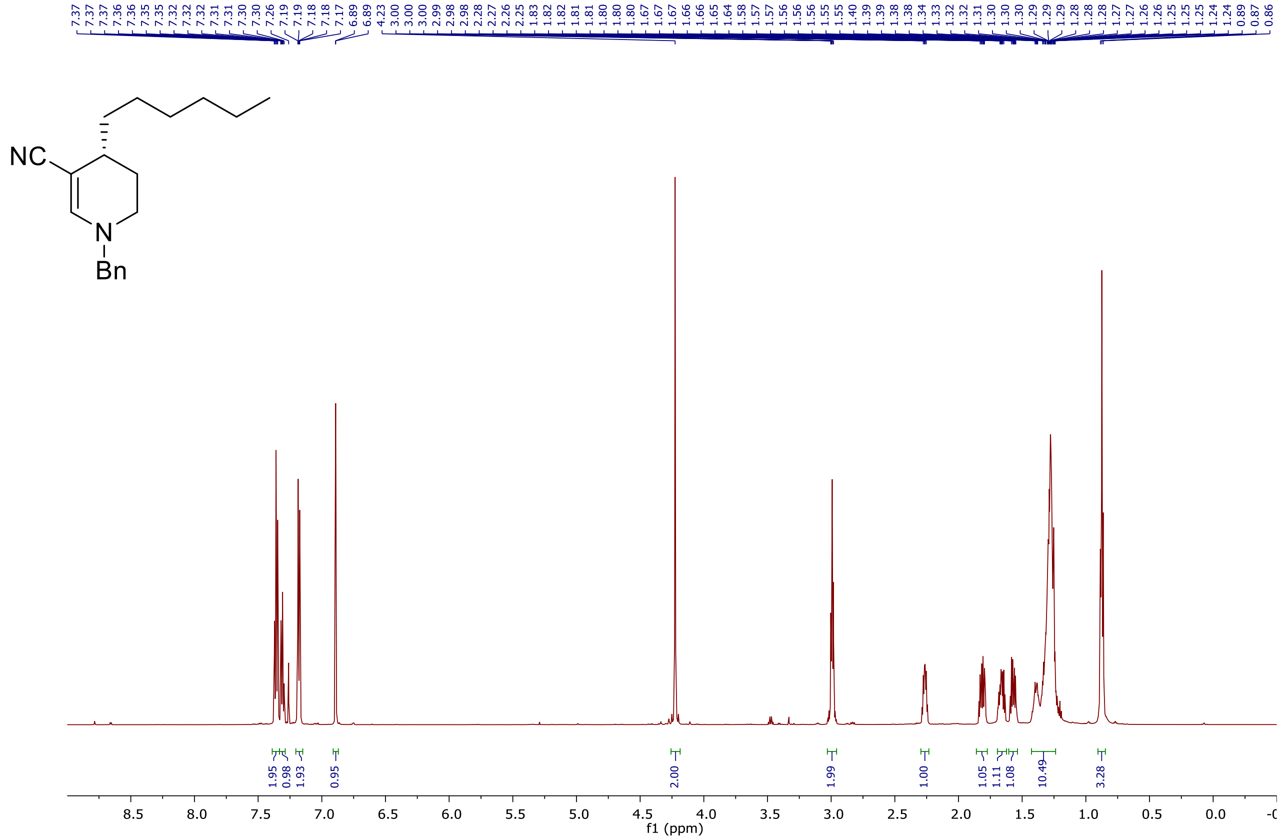

^{13}C NMR with $CDCl_3$, 101 MHz

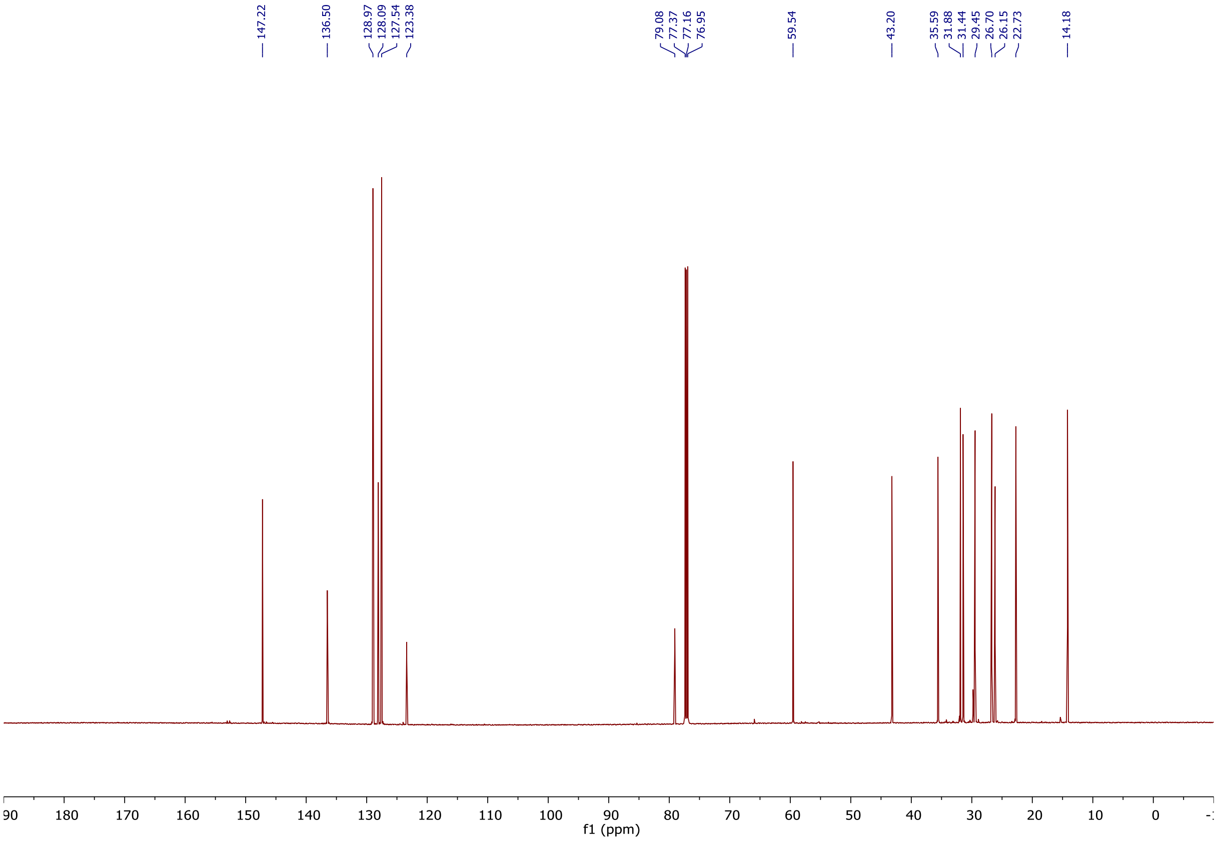

NMR spectra of dimethyl (1*S*,5*R*,6*S*)-2-benzyl-4-cyano-5-hexyl-2-azabicyclo[4.2.0]octa-3,7-diene-7,8-dicarboxylate (8)

^{1}H NMR with $CDCl_3$, 400 MHz

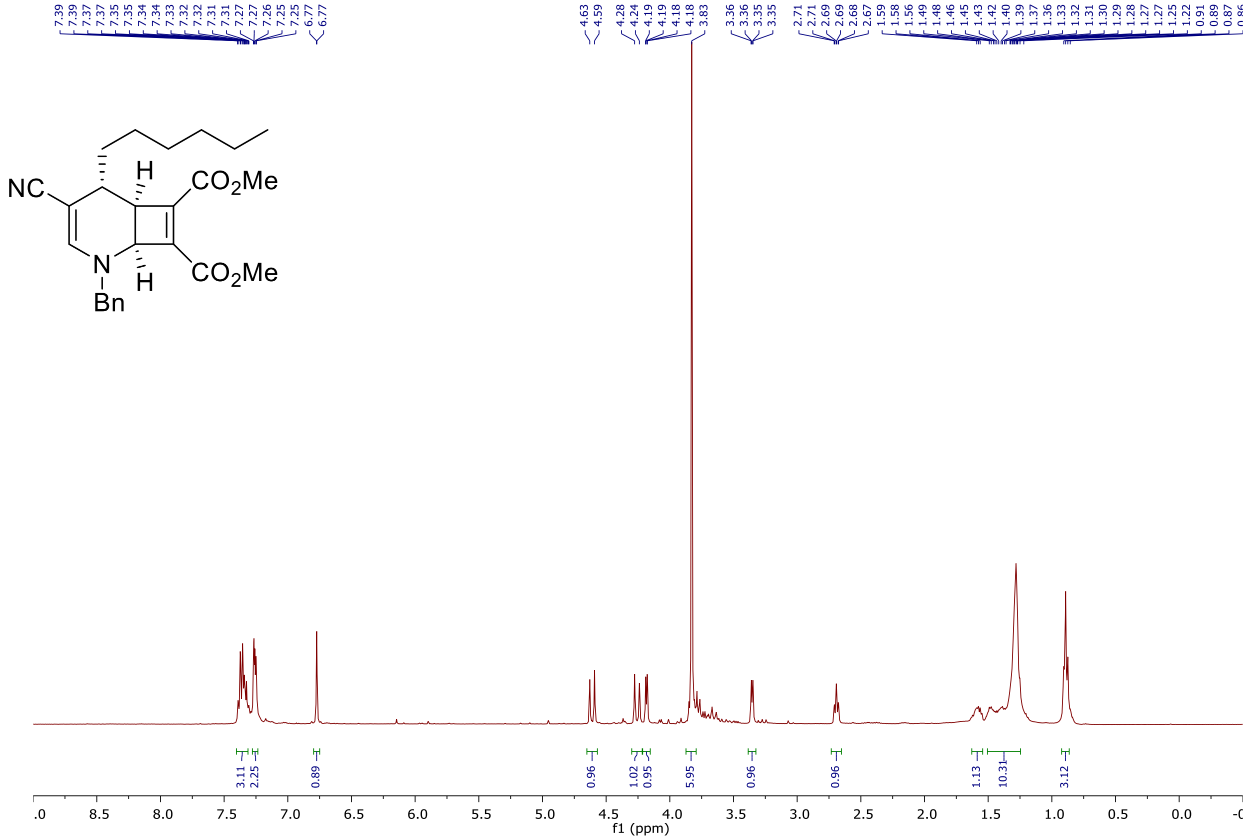

^{13}C NMR with $CDCl_3$, 101 MHz

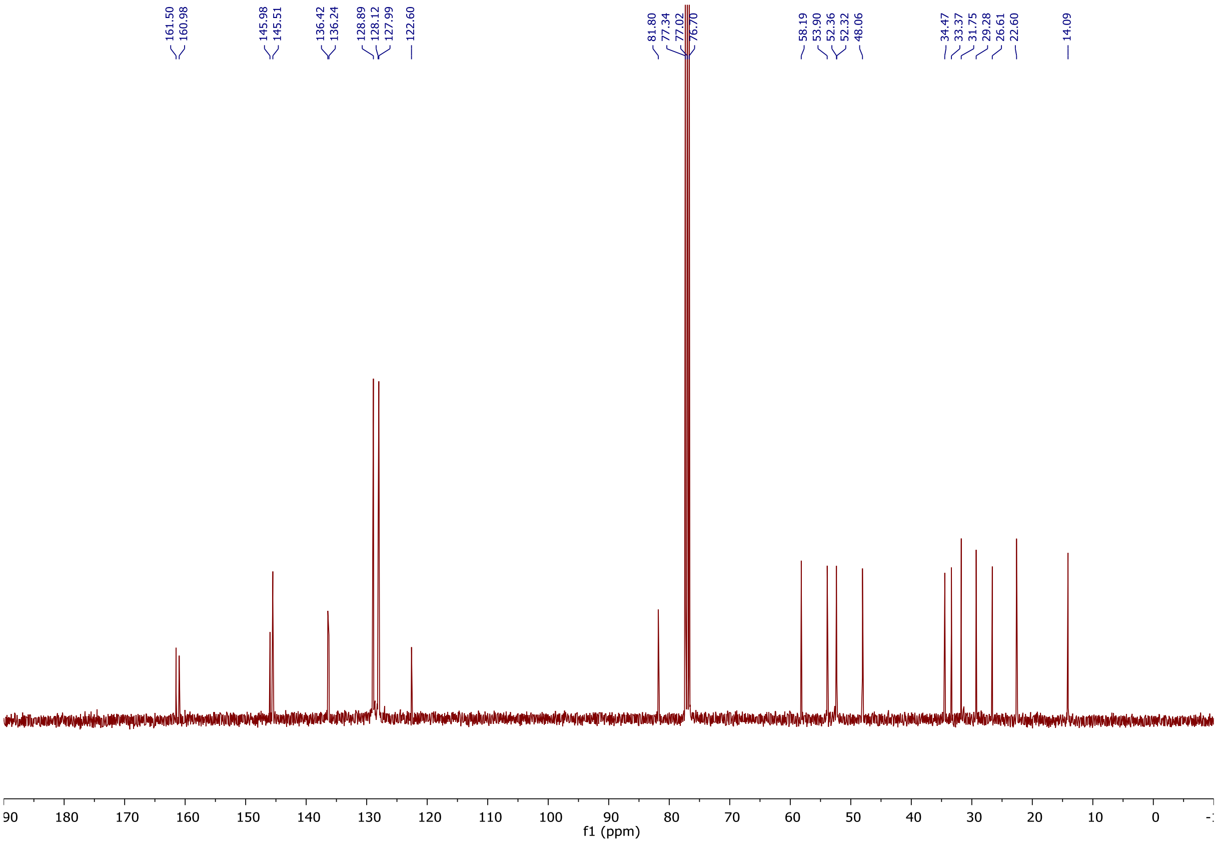

NMR spectra of *N*-benzyl-5-bromo-4-hexyl-6-oxo-1,6-dihydropyridine-3-carbonitrile (9)

^{1}H NMR with $CDCl_3$, 400 MHz

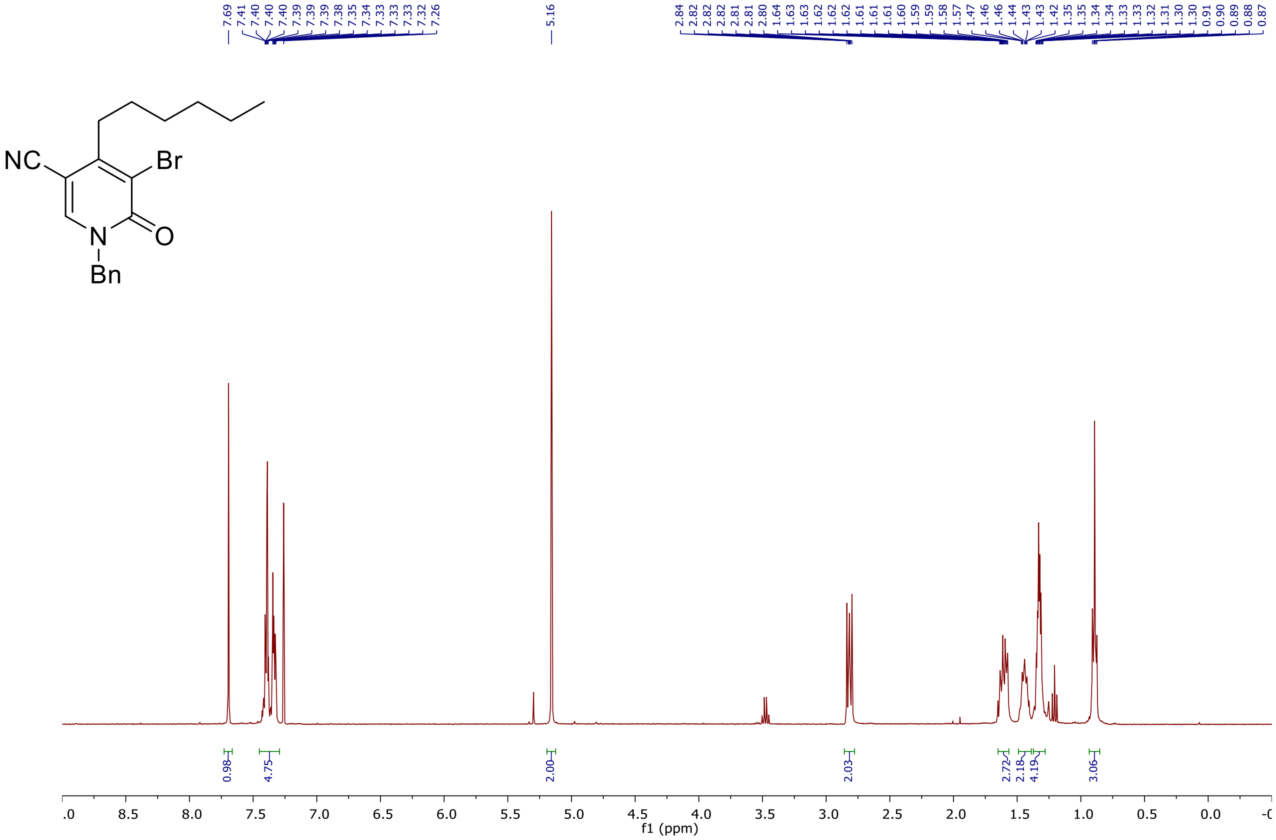

^{13}C NMR with $CDCl_3$, 101 MHz

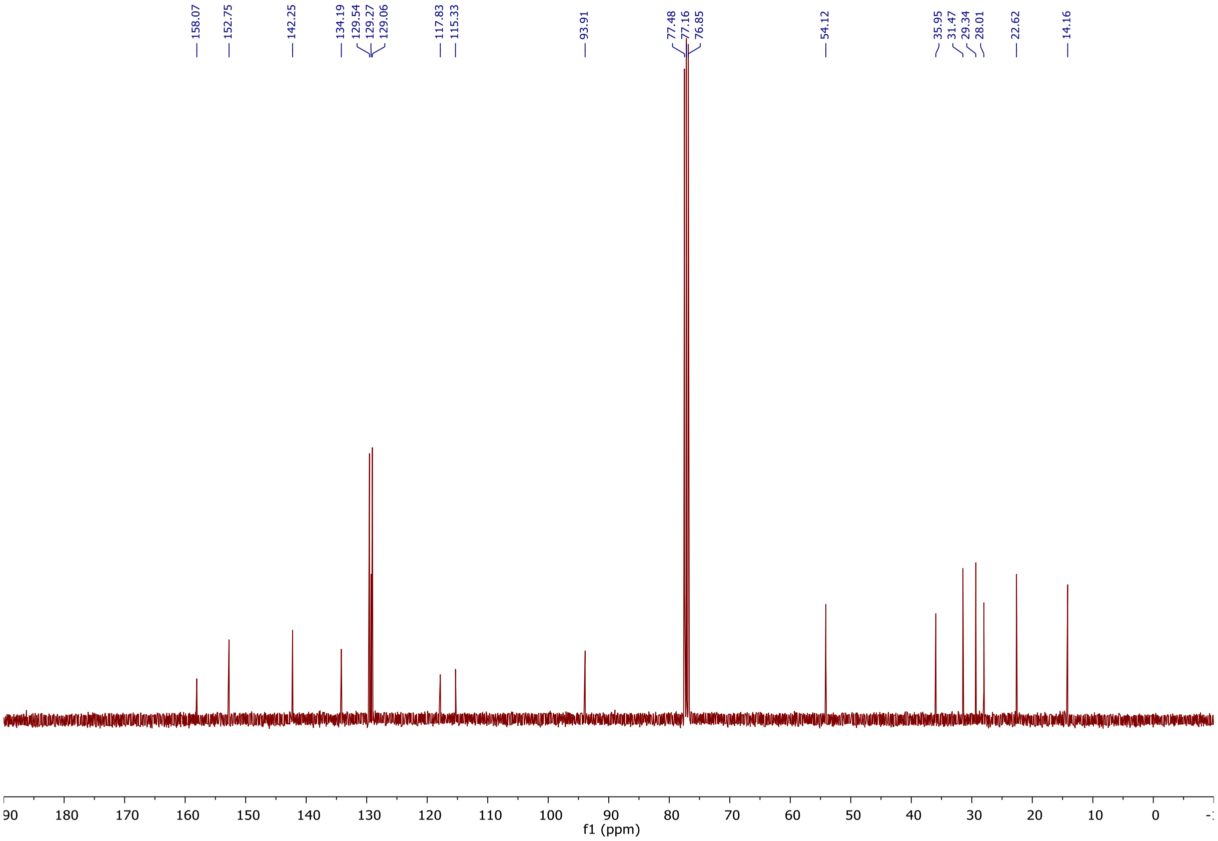

9. Cartesian coordinates

II-56

C	5.571046	3.456824	-2.083835
C	4.897375	4.001302	-1.009161
C	3.680189	3.427449	-0.546611
C	3.154691	2.268752	-1.210135
C	3.870069	1.741660	-2.324474
C	5.049516	2.318080	-2.747956
H	3.371891	4.851302	1.069227
H	6.511054	3.900805	-2.424966
H	5.292686	4.881855	-0.493450
C	2.972953	3.966770	0.563588
C	1.949263	1.659093	-0.719040
H	3.487423	0.856298	-2.834570
H	5.593001	1.885601	-3.592699
C	1.282416	2.223029	0.366692
C	1.804557	3.388659	1.001879
H	1.273054	3.821146	1.853309
C	1.477642	0.399664	-1.381641
C	0.530253	0.472592	-2.457290
C	2.014394	-0.827985	-0.995960
C	-0.048904	1.704070	-2.882444
C	0.134065	-0.736932	-3.123745
C	1.625612	-2.011256	-1.687012
C	-0.989570	1.736201	-3.893702
H	0.258276	2.631552	-2.393248
C	-0.838528	-0.670399	-4.161017
C	0.720725	-1.967948	-2.722439
H	2.039234	-2.972670	-1.372687
C	-1.395453	0.537309	-4.535190
H	-1.425372	2.690684	-4.202782
H	-1.139616	-1.597724	-4.658178
H	0.424891	-2.887595	-3.235719
H	-2.143438	0.574802	-5.332427
P	3.219086	-0.844338	0.429073
P	-0.236359	1.448911	1.045469
C	-1.294328	2.881217	1.496875
C	-2.104457	2.868486	2.643870
C	-1.440975	3.937845	0.581250
C	-3.045456	3.878836	2.857635
H	-2.015428	2.062662	3.376768

C	-2.376571	4.950249	0.808660
H	-0.827871	3.971448	-0.324561
C	-3.203177	4.936246	1.945771
H	-3.677340	3.838191	3.750326
H	-2.472461	5.762634	0.081447
C	0.249283	0.634886	2.616514
C	-0.680541	-0.250766	3.192616
C	1.521313	0.746791	3.196289
C	-0.345543	-1.005077	4.316281
H	-1.672091	-0.369141	2.743482
C	1.854199	-0.024880	4.313211
H	2.270678	1.411454	2.762993
C	0.937392	-0.920965	4.884811
H	-1.083746	-1.691981	4.742147
H	2.859822	0.059334	4.737013
C	2.982827	-2.564247	1.047994
C	1.817548	-2.795570	1.806720
C	3.883684	-3.624439	0.872444
C	1.555468	-4.052593	2.349346
H	1.104515	-1.982844	1.974856
C	3.620129	-4.881805	1.431312
H	4.798673	-3.476429	0.291516
C	2.454853	-5.120980	2.175552
H	0.636263	-4.204593	2.925679
H	4.337415	-5.695411	1.279158
C	4.829691	-0.852083	-0.475750
C	5.857288	-0.054232	0.054356
C	5.061160	-1.506067	-1.698768
C	7.072001	0.094002	-0.619487
H	5.696217	0.479239	0.996873
C	6.275449	-1.353466	-2.370470
H	4.282267	-2.129815	-2.145969
C	7.298578	-0.541967	-1.849965
H	7.851992	0.730985	-0.189300
H	6.429455	-1.867526	-3.325239
C	1.331597	-1.814596	6.033665
H	0.480335	-2.007778	6.709039
H	1.677699	-2.794540	5.651492
H	2.156337	-1.380619	6.623938
C	-4.218056	6.023703	2.198305
H	-3.876291	6.699738	3.004691
H	-4.390773	6.637024	1.298221

H	-5.184868	5.598322	2.520741
C	2.178791	-6.468205	2.799206
H	2.812230	-7.257914	2.360714
H	2.378142	-6.448016	3.887813
H	1.121610	-6.761805	2.670859
C	8.582239	-0.324488	-2.613478
H	9.431094	-0.131640	-1.935027
H	8.832036	-1.193822	-3.245887
H	8.492027	0.553172	-3.282656
Cu	-1.358313	-0.236115	-0.123353
C	-1.409968	-2.226666	-0.112014
H	-0.338525	-2.482450	-0.050119
H	-1.778171	-2.633022	-1.069376
C	-2.168993	-2.797429	1.082876
H	-3.174738	-2.354844	1.212197
H	-2.314592	-3.895435	0.997750
H	-1.623313	-2.617051	2.024846
C	-4.814713	0.818546	0.693639
C	-3.928099	1.706566	0.024227
C	-3.135708	1.221705	-0.998382
C	-3.183059	-0.166904	-1.332342
H	-3.893111	2.758373	0.309853
H	-2.766670	-0.516572	-2.279473
C	-4.978179	-0.471489	0.190598
H	-5.736021	-1.153162	0.580917
H	-2.487892	1.881831	-1.576643
C	-5.585015	1.256205	1.815490
N	-6.188240	1.629203	2.735122
N	-4.241167	-0.916437	-0.839907
C	-4.540047	-2.251915	-1.434161
H	-3.841429	-2.379496	-2.271956
H	-4.304157	-3.018377	-0.681722
C	-5.975359	-2.352647	-1.893783
C	-6.421402	-1.588671	-2.985361
C	-6.878897	-3.190929	-1.226036
C	-7.752842	-1.662393	-3.399636
H	-5.722714	-0.931647	-3.513987
C	-8.213125	-3.268265	-1.643587
H	-6.538020	-3.790491	-0.375893
C	-8.651304	-2.503444	-2.728814
H	-8.091843	-1.065459	-4.251478
H	-8.909811	-3.927619	-1.117737

H	-9.693874	-2.561825	-3.055430

L11CuEt

C	-2.001641	-4.989524	1.802986
C	-1.075293	-4.359394	2.607686
C	-0.375253	-3.207628	2.148599
C	-0.646333	-2.699835	0.833672
C	-1.602807	-3.381335	0.027343
C	-2.264698	-4.494501	0.499735
H	0.784334	-2.921036	3.965279
H	-2.538748	-5.870723	2.167036
H	-0.865270	-4.735394	3.613997
C	0.583012	-2.540944	2.958793
C	0.040457	-1.527766	0.373936
H	-1.820514	-2.999854	-0.971699
H	-3.007299	-4.993337	-0.129551
C	1.005378	-0.928474	1.183559
C	1.260525	-1.437872	2.487407
H	2.001198	-0.946551	3.123194
C	-0.298689	-0.952633	-0.970055
C	0.402039	-1.416526	-2.130111
C	-1.282099	0.035531	-1.084089
C	1.410555	-2.421364	-2.057381
C	0.105895	-0.835276	-3.408710
C	-1.582906	0.575044	-2.366677
C	2.107658	-2.811428	-3.181527
H	1.642650	-2.871390	-1.090503
C	0.841109	-1.261814	-4.551257
C	-0.910473	0.154018	-3.492480
H	-2.344025	1.353334	-2.452878
C	1.824241	-2.224192	-4.442065
H	2.890811	-3.570827	-3.099354
H	0.613221	-0.807948	-5.521001
H	-1.143113	0.593781	-4.467387
H	2.387325	-2.538136	-5.326333
P	-2.091480	0.651925	0.480875
P	1.979558	0.491314	0.510967
C	3.372415	-0.357030	-0.336958
C	3.838198	0.176499	-1.549492
C	3.935719	-1.555952	0.131814
C	4.833423	-0.477302	-2.277972
H	3.398811	1.101258	-1.938089

C	4.931956	-2.203414	-0.600870
H	3.583450	-2.002722	1.065992
C	5.390178	-1.683429	-1.823647
H	5.171734	-0.049730	-3.227344
H	5.353701	-3.140071	-0.221525
C	2.680587	1.284151	2.009231
C	3.980736	1.068300	2.486972
C	1.840774	2.172506	2.706319
C	4.425199	1.720179	3.642782
H	4.655275	0.387329	1.960727
C	2.287796	2.809545	3.863417
H	0.826435	2.362747	2.337746
C	3.590321	2.597777	4.351724
H	5.443844	1.538330	4.001040
H	1.616151	3.490968	4.396091
C	-3.058412	2.111285	-0.111705
C	-4.448844	2.142748	-0.301861
C	-2.335298	3.300962	-0.324129
C	-5.088190	3.321186	-0.706688
H	-5.049009	1.244218	-0.138406
C	-2.974129	4.466565	-0.744431
H	-1.249734	3.305544	-0.183245
C	-4.366550	4.501140	-0.940963
H	-6.174268	3.317071	-0.849309
H	-2.378224	5.368339	-0.921666
C	-3.372258	-0.659872	0.720313
C	-4.118671	-1.213550	-0.334616
C	-3.560027	-1.177206	2.011302
C	-5.021992	-2.251736	-0.101484
H	-3.982268	-0.839600	-1.354052
C	-4.459300	-2.221870	2.239222
H	-2.977778	-0.774051	2.846150
C	-5.198968	-2.783306	1.187112
H	-5.587827	-2.671726	-0.940016
H	-4.574878	-2.621219	3.252279
C	4.078264	3.322447	5.582865
H	3.287433	3.389543	6.350615
H	4.954227	2.821821	6.029208
H	4.378562	4.358694	5.335308
C	6.414801	-2.423259	-2.648217
H	7.073062	-3.045368	-2.017813
H	5.915802	-3.098456	-3.370193

H	7.043980	-1.729436	-3.232047
C	-5.054283	5.777657	-1.362682
H	-4.488367	6.295099	-2.157894
H	-6.075675	5.586754	-1.733873
H	-5.135183	6.483151	-0.513521
C	-6.108470	-3.963457	1.425727
H	-6.903853	-4.027748	0.663412
H	-5.532276	-4.908478	1.382632
H	-6.582855	-3.917285	2.421499
Cu	1.057091	2.040629	-0.857304
C	0.679085	3.505455	-2.119522
H	-0.344893	3.368785	-2.526125
H	1.356236	3.404260	-2.996634
C	0.816784	4.926322	-1.547161
H	1.842982	5.118823	-1.178105
H	0.585067	5.730844	-2.281365
H	0.143815	5.088993	-0.682673

1a

C	0.432463	1.031715	0.000143
C	1.481713	1.943775	0.000092
C	2.795050	1.482317	0.000013
C	3.023657	0.092264	-0.000010
C	1.932463	-0.775327	0.000062
H	-0.613329	1.339647	0.000210
H	1.256786	3.011703	0.000118
H	3.635703	2.179564	-0.000031
H	2.047223	-1.860506	0.000075
C	4.354859	-0.445858	-0.000119
N	5.432304	-0.873180	-0.000220
N	0.671039	-0.296874	0.000124
C	-0.454342	-1.317961	0.000307
H	-0.285973	-1.937339	-0.892992
H	-0.285999	-1.936954	0.893879
C	-1.830274	-0.715859	0.000114
C	-2.476181	-0.425621	-1.213205
C	-2.476492	-0.425508	1.213236
C	-3.750216	0.149133	-1.212910
H	-1.980424	-0.653020	-2.162272
C	-3.750533	0.149234	1.212567
H	-1.980966	-0.652791	2.162451
C	-4.387566	0.437688	-0.000266

H	-4.248706	0.367614	-2.161597
H	-4.249267	0.367790	2.161108
H	-5.386060	0.884729	-0.000416

II-*proS*

C	3.005341	5.671419	1.043773
C	3.807976	4.728276	0.436307
C	3.431927	3.356301	0.414130
C	2.196533	2.951199	1.017716
C	1.400813	3.950895	1.650783
C	1.793413	5.273631	1.662624
H	5.216607	2.663325	-0.614185
H	3.301661	6.724492	1.052858
H	4.749307	5.021738	-0.038646
C	4.254341	2.367933	-0.185136
C	1.781959	1.576326	0.941110
H	0.456876	3.666584	2.118902
H	1.161083	6.023515	2.147207
C	2.598685	0.638636	0.300456
C	3.854916	1.052775	-0.229776
H	4.515711	0.317091	-0.689099
C	0.489806	1.216270	1.620879
C	0.508535	1.059349	3.053672
C	-0.723991	1.121344	0.935534
C	1.697849	1.222124	3.821820
C	-0.702904	0.739984	3.754547
C	-1.922045	0.845647	1.658837
C	1.696762	1.043538	5.190358
H	2.625799	1.492885	3.317292
C	-0.672187	0.550167	5.164165
C	-1.914110	0.646601	3.018285
H	-2.871771	0.787570	1.126646
C	0.504142	0.693233	5.871298
H	2.626221	1.168606	5.753610
H	-1.604028	0.296446	5.679034
H	-2.850553	0.427061	3.541005
H	0.518034	0.547415	6.955440
P	-0.816203	1.028421	-0.905664
P	2.006420	-1.101047	0.029919
C	2.238058	-1.926129	1.657040
C	3.100024	-1.412016	2.638762
C	1.573383	-3.135749	1.924438

C	3.254503	-2.062730	3.863333
H	3.658897	-0.493540	2.447515
C	1.735688	-3.786228	3.150260
H	0.942168	-3.603206	1.163980
C	2.565430	-3.252646	4.149688
H	3.921838	-1.631259	4.616308
H	1.205404	-4.726504	3.331653
C	3.307234	-1.892666	-1.012827
C	4.005306	-3.040539	-0.605135
C	3.551156	-1.395909	-2.307678
C	4.907337	-3.671867	-1.468910
H	3.855227	-3.453031	0.395247
C	4.461524	-2.021177	-3.157843
H	3.045101	-0.490539	-2.650641
C	5.151217	-3.180259	-2.759488
H	5.437893	-4.565074	-1.123002
H	4.637771	-1.602213	-4.153916
C	-2.545867	1.469148	-1.340701
C	-3.204737	0.672671	-2.289520
C	-3.215324	2.599428	-0.834506
C	-4.489410	1.002854	-2.734498
H	-2.704960	-0.209630	-2.695457
C	-4.496162	2.923228	-1.280669
H	-2.745637	3.226547	-0.072379
C	-5.157730	2.133196	-2.240299
H	-4.977940	0.368634	-3.480906
H	-4.998869	3.805506	-0.871877
C	0.192285	2.385737	-1.609830
C	1.181034	2.049066	-2.545909
C	0.030559	3.735555	-1.255902
C	2.008745	3.032006	-3.092593
H	1.309398	1.004542	-2.840636
C	0.857473	4.712404	-1.807480
H	-0.720302	4.032404	-0.521198
C	1.870550	4.378502	-2.722976
H	2.784060	2.742490	-3.808832
H	0.731752	5.754822	-1.499762
C	6.099863	-3.879191	-3.702368
H	6.661274	-3.156001	-4.319419
H	6.824367	-4.508399	-3.157894
H	5.545962	-4.538434	-4.398085
C	2.696492	-3.916563	5.498126

H	2.428143	-4.985734	5.454243
H	3.724065	-3.830517	5.892324
H	2.024310	-3.435190	6.234290
C	-6.559873	2.472335	-2.679653
H	-6.691690	3.561888	-2.797691
H	-6.817505	1.983410	-3.633992
H	-7.294024	2.135940	-1.922687
C	2.811450	5.433827	-3.247429
H	3.279102	5.129041	-4.198967
H	2.294782	6.396708	-3.403871
H	3.624063	5.615025	-2.517778
Cu	-0.094860	-1.142194	-1.457469
C	0.273908	-1.693897	-3.370002
H	0.552206	-2.758699	-3.442137
H	1.180362	-1.104747	-3.590693
C	-0.850573	-1.329362	-4.321380
H	-1.819897	-1.774712	-4.022713
H	-0.994783	-0.235078	-4.371096
H	-0.659810	-1.689789	-5.354702
C	-2.002564	-3.499915	-1.992678
C	-0.937474	-3.077296	-1.103641
C	-1.384172	-2.437269	0.106006
C	-2.706687	-2.084669	0.267287
H	-0.010196	-3.655638	-1.111056
H	-3.055304	-1.594379	1.173212
C	-3.301354	-3.099332	-1.773569
H	-4.113438	-3.370686	-2.449608
H	-0.718012	-2.265362	0.950126
C	-1.721480	-4.343656	-3.113317
N	-1.482086	-5.040389	-4.010813
N	-3.650287	-2.376359	-0.666931
C	-5.094746	-2.008817	-0.501131
H	-5.351351	-1.360722	-1.353558
H	-5.667716	-2.945108	-0.591464
C	-5.397059	-1.317525	0.802459
C	-5.408191	0.083471	0.870392
C	-5.626945	-2.062410	1.970857
C	-5.632512	0.733329	2.087903
H	-5.227966	0.669953	-0.032749
C	-5.849437	-1.414395	3.189347
H	-5.623529	-3.156082	1.927042
C	-5.848366	-0.014847	3.250446

H	-5.631352	1.826443	2.125877
H	-6.025730	-2.003136	4.094247
H	-6.020689	0.491495	4.204859

II-*proR*

C	5.989347	-2.507499	1.887903
C	5.372240	-2.980803	0.748797
C	4.028081	-2.627311	0.447592
C	3.306184	-1.760287	1.330960
C	3.980118	-1.282074	2.492862
C	5.281629	-1.650703	2.767004
H	3.938666	-3.722791	-1.426529
H	7.023873	-2.784666	2.111163
H	5.909920	-3.634088	0.054789
C	3.382617	-3.091579	-0.726849
C	1.951805	-1.383660	1.014778
H	3.460858	-0.607698	3.175080
H	5.774846	-1.269752	3.666250
C	1.322558	-1.926207	-0.110782
C	2.076699	-2.756070	-0.994137
H	1.620374	-3.124859	-1.913070
C	1.295008	-0.381001	1.918851
C	0.800771	-0.820811	3.193916
C	1.193380	0.968180	1.559261
C	0.828015	-2.191698	3.581946
C	0.218742	0.127643	4.097757
C	0.566052	1.886448	2.453384
C	0.312189	-2.597352	4.795918
H	1.250555	-2.930074	2.897708
C	-0.291049	-0.317498	5.348942
C	0.112833	1.483450	3.689163
H	0.440908	2.929666	2.158512
C	-0.249745	-1.652728	5.692887
H	0.332936	-3.656722	5.068351
H	-0.734490	0.417992	6.027176
H	-0.352314	2.208151	4.364447
H	-0.654214	-1.987925	6.652563
P	1.615123	1.525855	-0.153212
P	-0.392387	-1.433799	-0.653220
C	-1.535913	-1.704296	0.777594
C	-2.172553	-2.925528	1.061868
C	-1.815574	-0.612920	1.614165

C	-3.022969	-3.047842	2.164012
H	-2.021170	-3.797456	0.424889
C	-2.660356	-0.739568	2.716038
H	-1.342320	0.349161	1.424549
C	-3.269928	-1.964593	3.023672
H	-3.499520	-4.013176	2.362745
H	-2.836409	0.131516	3.353439
C	-0.883710	-2.719317	-1.869960
C	-1.455975	-2.274402	-3.070529
C	-0.735643	-4.107985	-1.679880
C	-1.894599	-3.178556	-4.042276
H	-1.543430	-1.202873	-3.252259
C	-1.169952	-5.007042	-2.653289
H	-0.253406	-4.494334	-0.777752
C	-1.764909	-4.561424	-3.849205
H	-2.333517	-2.795019	-4.968786
H	-1.038641	-6.081145	-2.486486
C	1.764652	3.356970	-0.080309
C	1.227953	4.082447	-1.154978
C	2.437123	4.067836	0.932633
C	1.355576	5.473790	-1.221284
H	0.718841	3.551172	-1.962849
C	2.556805	5.455005	0.864925
H	2.860180	3.542402	1.792410
C	2.021316	6.185264	-0.213363
H	0.928329	6.011356	-2.073535
H	3.078503	5.986637	1.667529
C	3.340098	0.971039	-0.454037
C	3.629788	0.251749	-1.620742
C	4.385219	1.244931	0.446025
C	4.931392	-0.185373	-1.882618
H	2.829934	0.002411	-2.318085
C	5.678447	0.802185	0.180683
H	4.188512	1.778752	1.378706
C	5.976166	0.074605	-0.986109
H	5.128450	-0.759661	-2.793209
H	6.473245	1.005623	0.905177
C	-2.253628	-5.550105	-4.878691
H	-3.219905	-5.991337	-4.568007
H	-2.405557	-5.072975	-5.861357
H	-1.542342	-6.385518	-5.002657
C	-4.124702	-2.119828	4.255656

H	-4.607611	-1.168918	4.539691
H	-4.908053	-2.884246	4.116595
H	-3.500185	-2.435709	5.113511
C	2.182445	7.683995	-0.278140
H	1.550189	8.128594	-1.064842
H	1.921821	8.156358	0.685978
H	3.232738	7.955224	-0.496678
C	7.372354	-0.442643	-1.226774
H	7.492462	-0.834698	-2.250930
H	8.128332	0.347149	-1.066649
H	7.603034	-1.262700	-0.520587
Cu	-0.059874	0.728267	-1.714828
C	0.579250	0.620208	-3.643419
H	-0.232130	0.383747	-4.348743
H	1.199546	-0.291965	-3.542443
C	1.392383	1.810797	-4.121717
H	0.770936	2.720339	-4.229019
H	2.216981	2.058149	-3.430175
H	1.845649	1.625758	-5.118694
C	-2.718973	1.128249	-3.144216
C	-1.670321	1.916473	-2.519974
C	-1.882335	2.200369	-1.123566
C	-2.878888	1.557637	-0.424082
H	-1.148105	2.645391	-3.141407
H	-3.067233	1.754323	0.630731
C	-3.677130	0.503629	-2.378674
H	-4.438111	-0.141028	-2.819458
H	-1.298653	2.966417	-0.609994
C	-2.756742	0.945303	-4.561136
N	-2.781708	0.791661	-5.712365
N	-3.738198	0.682815	-1.028606
C	-4.755725	-0.033229	-0.222786
H	-5.369416	-0.608119	-0.932456
H	-4.230506	-0.755645	0.420735
C	-5.617640	0.899711	0.601055
C	-6.163677	2.063230	0.035758
C	-5.902957	0.588826	1.937418
C	-6.978374	2.903812	0.798849
H	-5.946717	2.319416	-1.006260
C	-6.725115	1.425832	2.700412
H	-5.478971	-0.314067	2.383738
C	-7.261516	2.586520	2.133732

H	-7.396484	3.809315	0.349107
H	-6.939335	1.171314	3.742745
H	-7.899049	3.245702	2.730541

TSI-*proS*

C	3.252528	5.577822	0.981397
C	4.026932	4.595313	0.400387
C	3.597325	3.238939	0.390610
C	2.337786	2.891907	0.980963
C	1.571572	3.931085	1.586155
C	2.015796	5.237353	1.585390
H	5.364340	2.464322	-0.609569
H	3.589975	6.618481	0.980753
H	4.986149	4.844921	-0.063600
C	4.387264	2.211798	-0.186786
C	1.870346	1.533875	0.917926
H	0.609878	3.690328	2.042419
H	1.405872	6.018800	2.048323
C	2.659512	0.557644	0.301690
C	3.935964	0.913121	-0.221973
H	4.567556	0.145876	-0.671289
C	0.555504	1.233677	1.583395
C	0.553546	1.081088	3.016105
C	-0.654801	1.188995	0.885967
C	1.741953	1.192376	3.795201
C	-0.677599	0.815569	3.705167
C	-1.870483	0.967753	1.597310
C	1.720196	1.014279	5.163494
H	2.685682	1.421885	3.299126
C	-0.668097	0.625534	5.115259
C	-1.884316	0.772913	2.957538
H	-2.816007	0.946640	1.055270
C	0.506726	0.716826	5.833091
H	2.648787	1.098837	5.735674
H	-1.614888	0.412992	5.621400
H	-2.834240	0.593932	3.471178
H	0.504084	0.570599	6.917271
P	-0.732333	1.078467	-0.957546
P	2.005791	-1.156059	0.024934
C	2.134510	-2.000059	1.645507
C	2.995258	-1.546423	2.657238
C	1.386037	-3.166609	1.875919

C	3.071461	-2.218220	3.877733
H	3.612570	-0.660612	2.493264
C	1.471332	-3.838372	3.097506
H	0.746027	-3.578191	1.090897
C	2.302874	-3.367393	4.126959
H	3.739181	-1.836326	4.656620
H	0.877386	-4.744469	3.253108
C	3.290553	-1.991082	-0.998239
C	4.043658	-3.077943	-0.529098
C	3.482998	-1.573598	-2.329887
C	4.955554	-3.727008	-1.368919
H	3.927435	-3.426097	0.500066
C	4.401767	-2.217284	-3.157227
H	2.932060	-0.714240	-2.722793
C	5.151480	-3.314024	-2.695011
H	5.531266	-4.571710	-0.976898
H	4.538371	-1.862798	-4.184017
C	-2.432191	1.599297	-1.413830
C	-3.124183	0.823358	-2.355236
C	-3.048421	2.766639	-0.924357
C	-4.387852	1.211415	-2.812763
H	-2.670598	-0.091787	-2.740649
C	-4.310089	3.146428	-1.380968
H	-2.553974	3.378147	-0.165216
C	-5.003661	2.378015	-2.335352
H	-4.902603	0.591794	-3.553850
H	-4.772152	4.055948	-0.984008
C	0.355206	2.371166	-1.666329
C	1.358620	1.962211	-2.556359
C	0.245817	3.737363	-1.357612
C	2.249253	2.887489	-3.104132
H	1.451933	0.902394	-2.809887
C	1.135245	4.657527	-1.909497
H	-0.513496	4.091280	-0.657784
C	2.160954	4.249775	-2.780045
H	3.034661	2.540295	-3.782681
H	1.048576	5.713760	-1.637418
C	6.113744	-4.031619	-3.609345
H	6.646405	-3.323863	-4.268380
H	6.862433	-4.608081	-3.040111
H	5.574963	-4.742749	-4.264490
C	2.353134	-4.053425	5.469708

H	2.008506	-5.099452	5.406469
H	3.374657	-4.046946	5.888238
H	1.700724	-3.531633	6.195952
C	-6.386415	2.776646	-2.786228
H	-6.471637	3.870684	-2.905173
H	-6.656678	2.298571	-3.742633
H	-7.140386	2.471150	-2.035641
C	3.166358	5.244721	-3.302529
H	3.659242	4.885026	-4.221558
H	2.697622	6.221140	-3.515660
H	3.955091	5.420372	-2.545838
Cu	-0.035073	-1.101760	-1.231821
C	-0.037098	-2.328657	-2.972725
H	0.118197	-3.403413	-3.123171
H	0.980619	-1.894125	-2.943383
C	-0.903938	-1.707206	-4.045776
H	-1.975846	-1.938294	-3.896283
H	-0.796897	-0.610396	-4.073216
H	-0.642128	-2.102565	-5.047556
C	-2.370366	-3.551451	-1.974919
C	-1.188180	-3.083782	-1.219695
C	-1.603657	-2.373238	0.001549
C	-2.875012	-1.918081	0.183151
H	-0.406381	-3.831628	-1.063624
H	-3.163436	-1.391850	1.090000
C	-3.621898	-3.034249	-1.742646
H	-4.480086	-3.334335	-2.347934
H	-0.918348	-2.253026	0.842546
C	-2.230368	-4.556208	-2.979397
N	-2.090246	-5.376592	-3.791745
N	-3.881637	-2.172106	-0.720651
C	-5.263184	-1.632481	-0.571238
H	-5.427413	-0.914428	-1.390362
H	-5.954368	-2.478743	-0.711639
C	-5.506697	-0.967967	0.760276
C	-5.412310	0.426042	0.880611
C	-5.781870	-1.736449	1.903128
C	-5.577592	1.044854	2.124180
H	-5.195655	1.030986	-0.002948
C	-5.945491	-1.120576	3.147170
H	-5.860016	-2.825154	1.818243
C	-5.839305	0.271924	3.260575

H	-5.493942	2.132631	2.203600
H	-6.157665	-1.728334	4.031738
H	-5.965116	0.753260	4.235071

TSI-*proR*

C	-6.132163	-2.807792	0.808326
C	-5.305017	-2.599418	1.892354
C	-3.943586	-2.230294	1.710580
C	-3.426201	-2.064268	0.383912
C	-4.311603	-2.280891	-0.711784
C	-5.625923	-2.647719	-0.505589
H	-3.475791	-2.109685	3.829589
H	-7.178329	-3.090060	0.959014
H	-5.686150	-2.711320	2.912024
C	-3.080494	-2.005117	2.814693
C	-2.054990	-1.669226	0.198290
H	-3.945022	-2.146058	-1.730596
H	-6.285802	-2.806725	-1.363650
C	-1.223330	-1.509414	1.310056
C	-1.763888	-1.659040	2.621441
H	-1.127481	-1.490355	3.491843
C	-1.576841	-1.439698	-1.206604
C	-1.284093	-2.585882	-2.024246
C	-1.440845	-0.152834	-1.738647
C	-1.387773	-3.917605	-1.528149
C	-0.849450	-2.401356	-3.377895
C	-0.972488	0.008311	-3.076473
C	-1.090616	-5.001447	-2.329292
H	-1.698663	-4.078229	-0.494219
C	-0.549070	-3.536849	-4.179740
C	-0.698800	-1.079384	-3.872889
H	-0.821033	1.010702	-3.480813
C	-0.667805	-4.812926	-3.669390
H	-1.173499	-6.014596	-1.924828
H	-0.211691	-3.378294	-5.208593
H	-0.347485	-0.932094	-4.898654
H	-0.430336	-5.680269	-4.292491
P	-1.606936	1.353207	-0.674103
P	0.509420	-0.870060	1.158359
C	1.345098	-1.873603	-0.137421
C	1.659944	-3.235376	-0.002718
C	1.672551	-1.233499	-1.342328

C	2.283010	-3.923376	-1.046271
H	1.415179	-3.779717	0.910503
C	2.289529	-1.924469	-2.382033
H	1.395721	-0.190449	-1.482169
C	2.605436	-3.285450	-2.255229
H	2.512470	-4.986216	-0.921034
H	2.523181	-1.396840	-3.311739
C	1.324220	-1.301208	2.743802
C	2.022006	-0.272633	3.394078
C	1.306022	-2.576294	3.342670
C	2.711161	-0.509948	4.587435
H	2.024767	0.730211	2.961725
C	1.992553	-2.807799	4.533416
H	0.731385	-3.391632	2.896065
C	2.713734	-1.782376	5.175801
H	3.249414	0.312680	5.068299
H	1.966267	-3.806947	4.980453
C	-1.861989	2.768477	-1.817200
C	-1.214470	3.974093	-1.503542
C	-2.717597	2.743591	-2.934857
C	-1.418471	5.122424	-2.274400
H	-0.555268	4.025512	-0.633830
C	-2.912916	3.890245	-3.704047
H	-3.229534	1.821551	-3.221735
C	-2.268656	5.101381	-3.389853
H	-0.905661	6.049706	-2.000255
H	-3.583841	3.846005	-4.568125
C	-3.218365	1.202814	0.196963
C	-3.248406	1.295926	1.595794
C	-4.427702	0.994056	-0.487562
C	-4.452168	1.176674	2.293247
H	-2.323120	1.437811	2.156236
C	-5.625392	0.873436	0.214565
H	-4.440379	0.895653	-1.575295
C	-5.660713	0.955336	1.617592
H	-4.444865	1.237958	3.385947
H	-6.552715	0.692069	-0.337515
C	3.440882	-2.054551	6.469380
H	4.099793	-2.936660	6.376959
H	4.056474	-1.194094	6.780316
H	2.725171	-2.271606	7.284265
C	3.234335	-4.038625	-3.399660

H	3.938321	-3.399217	-3.961264
H	3.775349	-4.934989	-3.052203
H	2.453337	-4.373482	-4.109087
C	-2.468443	6.326405	-4.247544
H	-3.508069	6.397546	-4.611632
H	-2.227460	7.251396	-3.697076
H	-1.813309	6.290014	-5.138977
C	-6.951826	0.753531	2.370281
H	-6.925197	1.234097	3.363098
H	-7.814996	1.153691	1.810732
H	-7.134475	-0.326973	2.526157
Cu	0.292055	1.434218	0.722194
C	0.186854	2.864383	2.352285
H	0.899912	3.092373	3.149507
H	-0.278808	1.895821	2.629762
C	-0.833496	3.975620	2.199795
H	-0.370602	4.916925	1.852519
H	-1.639073	3.713502	1.495853
H	-1.305221	4.193777	3.177264
C	2.938349	3.052912	1.909190
C	1.720951	3.186781	1.053330
C	1.974683	2.581866	-0.283049
C	2.959838	1.658799	-0.464446
H	1.341887	4.208687	0.989639
H	3.168759	1.214303	-1.437465
C	3.868763	2.077913	1.666772
H	4.699963	1.899354	2.351715
H	1.428031	2.933229	-1.161091
C	3.131042	3.921247	3.022478
N	3.262963	4.644255	3.925003
N	3.831206	1.299935	0.547755
C	4.738757	0.148554	0.386660
H	5.408502	0.153107	1.260823
H	4.142126	-0.777427	0.437286
C	5.537986	0.185860	-0.898238
C	6.255773	1.335975	-1.264392
C	5.579841	-0.941388	-1.729165
C	6.994366	1.358177	-2.450470
H	6.231857	2.222495	-0.621474
C	6.324743	-0.923384	-2.914108
H	5.023182	-1.839150	-1.446914
C	7.028995	0.228348	-3.279419

H	7.548429	2.259582	-2.729426
H	6.346899	-1.811708	-3.552726
H	7.606439	0.248217	-4.208703

EtMgBr·2Et$_2$O

C	-1.030087	-1.175203	2.534386
H	-0.135683	-1.725345	2.908486
H	-1.297855	-0.491881	3.369898
C	-2.174320	-2.187893	2.346358
H	-1.987073	-2.878006	1.498201
H	-2.365124	-2.830561	3.234620
H	-3.134951	-1.682688	2.123831
Mg	-0.264322	-0.024079	0.879344
Br	0.654447	2.317684	0.974117
O	-1.298393	-0.212864	-0.929908
O	1.367053	-1.131827	0.151372
C	-2.702479	-0.535727	-0.904148
H	-2.877547	-1.376752	-1.599938
H	-2.885850	-0.903033	0.115647
C	-3.607295	0.645405	-1.216993
H	-3.527433	0.962858	-2.270066
H	-4.657468	0.358452	-1.030031
H	-3.358933	1.503804	-0.568657
C	-0.680245	-0.028974	-2.223620
H	-1.255084	-0.612574	-2.964212
H	0.315831	-0.485052	-2.132442
C	-0.553997	1.432045	-2.625636
H	0.016095	1.498648	-3.569926
H	-1.536261	1.906241	-2.781349
H	-0.014015	1.995215	-1.846284
C	1.231920	-2.565804	0.149658
H	0.190671	-2.758565	0.450725
H	1.885846	-2.981055	0.938475
C	1.528125	-3.193737	-1.203012
H	1.368137	-4.284819	-1.142290
H	0.859289	-2.788290	-1.981286
H	2.573187	-3.023228	-1.513493
C	2.711462	-0.602926	0.073658
H	3.421201	-1.425400	0.266380
H	2.802943	0.132095	0.888933
C	2.973315	0.062593	-1.266174
H	4.000765	0.468351	-1.283730

H	2.865671	-0.650191	-2.101167
H	2.272205	0.900626	-1.415111

III-*S*

C	-5.729362	-3.167752	-1.341428
C	-5.745101	-1.811397	-1.593943
C	-4.729624	-0.961591	-1.073698
C	-3.674028	-1.528882	-0.285442
C	-3.701050	-2.929712	-0.025462
C	-4.699819	-3.728638	-0.543111
H	-5.559039	0.880548	-1.875211
H	-6.511946	-3.813135	-1.751138
H	-6.540281	-1.368597	-2.201620
C	-4.733038	0.438079	-1.310381
C	-2.608795	-0.689729	0.178906
H	-2.907352	-3.375166	0.577920
H	-4.696343	-4.803926	-0.341501
C	-2.616157	0.673147	-0.130607
C	-3.710029	1.233552	-0.846865
H	-3.739685	2.307027	-1.040688
C	-1.555705	-1.298522	1.062640
C	-1.875337	-1.438107	2.461307
C	-0.308478	-1.722559	0.598239
C	-3.120155	-1.002427	3.000722
C	-0.908999	-1.994850	3.363804
C	0.653104	-2.239058	1.520898
C	-3.393307	-1.120019	4.348551
H	-3.862575	-0.553856	2.338768
C	-1.222415	-2.112815	4.745559
C	0.360131	-2.380424	2.855385
H	1.646996	-2.518368	1.168472
C	-2.440507	-1.685243	5.232128
H	-4.351291	-0.765452	4.739858
H	-0.472593	-2.539931	5.418520
H	1.116022	-2.781337	3.537706
H	-2.671215	-1.773702	6.297831
P	0.344802	-1.324530	-1.084317
P	-1.139870	1.688850	0.311279
C	-1.278227	1.908091	2.120142
C	-2.412967	2.498784	2.701916
C	-0.289053	1.369850	2.955730
C	-2.550144	2.538499	4.088804

H	-3.201011	2.917474	2.068319
C	-0.436436	1.407520	4.343032
H	0.584999	0.874067	2.523394
C	-1.572645	1.981343	4.933151
H	-3.444103	2.993530	4.527067
H	0.335981	0.960885	4.976190
C	-1.347013	3.344779	-0.440003
C	-1.040947	4.504741	0.290212
C	-1.546410	3.464561	-1.830109
C	-0.934327	5.742288	-0.352759
H	-0.869517	4.448350	1.368217
C	-1.449541	4.704208	-2.460042
H	-1.766244	2.581816	-2.436192
C	-1.126930	5.866231	-1.736805
H	-0.689923	6.629802	0.239543
H	-1.610745	4.767967	-3.540858
C	1.462486	-2.697628	-1.549638
C	1.317185	-4.017145	-1.090772
C	2.473166	-2.414787	-2.483296
C	2.184686	-5.017570	-1.535122
H	0.536379	-4.270192	-0.368537
C	3.330731	-3.422153	-2.929954
H	2.593768	-1.394804	-2.863353
C	3.207956	-4.740206	-2.457943
H	2.065577	-6.036169	-1.152507
H	4.118364	-3.177000	-3.649373
C	-0.988344	-1.457458	-2.325942
C	-1.346058	-0.314101	-3.053146
C	-1.653782	-2.666649	-2.585769
C	-2.381753	-0.364489	-3.987695
H	-0.813535	0.625410	-2.875660
C	-2.687192	-2.710109	-3.519901
H	-1.379990	-3.575551	-2.043500
C	-3.081994	-1.558304	-4.223244
H	-2.658745	0.543367	-4.532729
H	-3.214182	-3.653351	-3.692206
C	-0.948465	7.188812	-2.439506
H	-1.075297	8.038572	-1.747766
H	0.068554	7.260866	-2.871062
H	-1.664182	7.305090	-3.271703
C	-1.767677	1.943301	6.427381
H	-2.244897	0.988173	6.720929

H	-0.806188	2.006962	6.965199
H	-2.420161	2.761569	6.776354
C	4.127256	-5.829973	-2.951022
H	5.129489	-5.435793	-3.190934
H	4.237773	-6.635092	-2.205026
H	3.725631	-6.289031	-3.874657
C	-4.257979	-1.597055	-5.165991
H	-4.201369	-0.797972	-5.924565
H	-4.331989	-2.568520	-5.684546
H	-5.199969	-1.455601	-4.601692
Cu	0.916798	0.791565	-0.402576
C	2.058100	3.254367	-1.822433
H	1.000376	3.329139	-1.522728
H	2.187449	2.274049	-2.323355
C	2.366316	4.386244	-2.803707
H	3.391393	4.321685	-3.207044
H	1.663145	4.356191	-3.654408
H	2.254864	5.369847	-2.312059
C	4.383267	2.973537	-0.792225
C	2.906108	3.254868	-0.527353
C	2.415623	2.213646	0.473736
C	3.004629	0.973690	0.555876
H	2.799907	4.251882	-0.066458
H	2.738328	0.262124	1.344638
C	4.887964	1.712281	-0.676497
H	5.914290	1.478147	-0.970092
H	1.768123	2.542674	1.291396
C	5.243177	4.030675	-1.199945
N	5.922117	4.918248	-1.529162
N	4.179845	0.673298	-0.124577
C	4.684110	-0.703557	-0.232747
H	4.160425	-1.215592	-1.055207
H	5.744091	-0.627852	-0.526723
C	4.561703	-1.530797	1.030295
C	4.159316	-2.870895	0.936532
C	4.872168	-0.999493	2.292294
C	4.054967	-3.665152	2.083876
H	3.911816	-3.296466	-0.039812
C	4.756412	-1.787684	3.441112
H	5.193868	0.043356	2.378515
C	4.344079	-3.122894	3.340456
H	3.730381	-4.706016	1.993196

H	4.991075	-1.358418	4.419874
H	4.251348	-3.738330	4.240377

III-*R*

C	-3.876558	-5.463432	0.462056
C	-4.451318	-4.385759	-0.179033
C	-3.859813	-3.093718	-0.105443
C	-2.646566	-2.915329	0.637302
C	-2.085685	-4.048831	1.294762
C	-2.683961	-5.288884	1.209405
H	-5.384182	-2.090519	-1.285072
H	-4.337750	-6.453342	0.396793
H	-5.373207	-4.508565	-0.755766
C	-4.442722	-1.967202	-0.741260
C	-2.023041	-1.623458	0.679521
H	-1.160178	-3.932110	1.861918
H	-2.231823	-6.146921	1.715659
C	-2.609204	-0.543938	0.009371
C	-3.839789	-0.731154	-0.681172
H	-4.318711	0.114760	-1.176052
C	-0.785277	-1.462229	1.514456
C	-0.958294	-1.321561	2.938062
C	0.501061	-1.453760	0.970936
C	-2.240044	-1.379340	3.558125
C	0.184909	-1.108545	3.777548
C	1.620502	-1.206244	1.821047
C	-2.378470	-1.249323	4.925292
H	-3.127356	-1.522252	2.939541
C	0.011031	-0.967159	5.181552
C	1.469584	-1.036184	3.176427
H	2.617620	-1.156006	1.383016
C	-1.244550	-1.040836	5.748779
H	-3.374151	-1.296874	5.375931
H	0.894161	-0.797484	5.805108
H	2.344073	-0.849555	3.807685
H	-1.368494	-0.933310	6.830381
P	0.824283	-1.436856	-0.850888
P	-1.707217	1.075300	-0.114267
C	-1.563204	1.674199	1.602294
C	-2.664739	1.743302	2.471528
C	-0.292567	2.005176	2.091857
C	-2.485712	2.133698	3.797523

H	-3.663683	1.472149	2.117354
C	-0.119025	2.390820	3.420949
H	0.574038	1.927584	1.432939
C	-1.211150	2.453625	4.299813
H	-3.350769	2.170069	4.467012
H	0.886403	2.627428	3.783211
C	-2.822977	2.224027	-1.000202
C	-3.304755	3.411591	-0.431581
C	-3.062211	1.987608	-2.368655
C	-4.014544	4.331725	-1.211345
H	-3.116315	3.639295	0.619963
C	-3.784209	2.900532	-3.132745
H	-2.665997	1.086686	-2.848398
C	-4.268163	4.096412	-2.570095
H	-4.373176	5.256111	-0.747827
H	-3.960009	2.692573	-4.193081
C	2.519013	-2.099887	-1.063968
C	3.314898	-1.530514	-2.073526
C	3.060222	-3.143332	-0.292724
C	4.618921	-1.978219	-2.292307
H	2.910400	-0.731350	-2.703477
C	4.367053	-3.582193	-0.513796
H	2.471076	-3.605299	0.504513
C	5.173017	-3.003066	-1.508631
H	5.221809	-1.513371	-3.078594
H	4.771691	-4.389362	0.105097
C	-0.275975	-2.643362	-1.670064
C	-1.215688	-2.155419	-2.590712
C	-0.223462	-4.023942	-1.420117
C	-2.105801	-3.024189	-3.223183
H	-1.263275	-1.081468	-2.796929
C	-1.115371	-4.886140	-2.056644
H	0.503889	-4.431847	-0.713533
C	-2.079430	-4.402549	-2.958235
H	-2.842829	-2.621424	-3.924759
H	-1.074510	-5.957172	-1.835932
C	-5.004995	5.100317	-3.421864
H	-5.767568	4.608739	-4.051829
H	-5.504386	5.867675	-2.807227
H	-4.306850	5.619849	-4.105312
C	-1.024221	2.796063	5.755930
H	-0.090259	3.357610	5.927059

H	-1.867916	3.392506	6.144309
H	-0.972352	1.868398	6.357491
C	6.603769	-3.435223	-1.706212
H	6.784144	-4.447447	-1.306969
H	6.885523	-3.423783	-2.773310
H	7.287924	-2.741683	-1.181155
C	-3.085034	-5.334531	-3.586184
H	-3.472941	-4.934943	-4.538648
H	-2.652033	-6.331991	-3.775245
H	-3.947476	-5.475341	-2.907019
Cu	0.359257	0.811647	-1.145416
C	-0.115700	3.919413	-2.711328
H	-0.322665	4.999531	-2.809725
H	-0.878649	3.515726	-2.027177
C	-0.246904	3.250906	-4.080165
H	0.568392	3.558679	-4.761318
H	-0.227076	2.148553	-4.001173
H	-1.207730	3.522641	-4.548684
C	1.319472	4.315351	-0.634384
C	1.270253	3.753407	-2.058733
C	1.759258	2.315445	-2.001786
C	2.560696	1.861125	-0.987155
H	1.989472	4.323390	-2.686136
H	3.106473	0.917987	-1.052841
C	2.088941	3.760758	0.340374
H	2.155609	4.205970	1.335116
H	1.783700	1.750870	-2.941257
C	0.577983	5.490834	-0.321989
N	-0.048578	6.443819	-0.088459
N	2.793413	2.588643	0.162430
C	3.565936	1.999322	1.271539
H	3.806929	2.823134	1.962334
H	2.932055	1.283999	1.825402
C	4.838830	1.319416	0.805573
C	5.685307	1.943404	-0.125838
C	5.193641	0.058501	1.304607
C	6.856524	1.313287	-0.554125
H	5.418339	2.925915	-0.527244
C	6.367579	-0.573804	0.879260
H	4.552509	-0.438457	2.037802
C	7.200574	0.051158	-0.052901
H	7.504875	1.809753	-1.282411

H	6.621977	-1.563011	1.270341
H	8.115870	-0.443336	-0.391994

10. References

[1] Kohn, W.; Sham, L. J. Self-Consistent Equations Including Exchange and Correlation Effects. *Phys. Rev. A* **1965**, *140,* 1133–1138.

[2] (a) Becke, A. D. Density-Functional Thermochemistry. III. The Role of Exact Exchange. *J. Chem. Phys.* **1993**, 98, 5648−5652. (b) Becke, A. D. A new Mixing of Hartree‑Fock and Local Density-Functional Theories. *J. Chem. Phys.* **1993**, 98, 1372−1377. (c) Lee, C.; Yang, W.; Parr, R. G. Development of the Colle-Salvetti Correlation-energy Formula into a Functional of the Electron Density. *Phys. Rev. B: Condens. Matter Mater. Phys.* **1988**, 37, 785−789. (d) Weigend, F.; Ahlrichs, R. Balanced Basis Sets of Split Valence, Triple Zeta Valence and Quadruple Zeta Valence Quality for H to Rn: Design and Assessment of Accuracy. *Phys. Chem. Chem. Phys.* **2005**, 7, 3297−3305.

[3] Tomasi, J.; Mennucci, B.; Cammi, R. Quantum Mechanical Continuum Solvation Models. *Chem. Rev.* **2005**, *105,* 2999–3093.

[4] Gaussian 16, Revision C.01, Frisch, M. J.; Trucks, G. W.; Schlegel, H. B.; Scuseria, G. E.; Robb, M. A.; Cheeseman, J. R.; Scalmani, G.; Barone, V.; Petersson, G. A.; Nakatsuji, H.; Li, X.; Caricato, M.; Marenich, A. V.; Bloino, J.; Janesko, B. G.; Gomperts, R.; Mennucci, B.; Hratchian, H. P.; Ortiz, J. V.; Izmaylov, A. F.; Sonnenberg, J. L.; Williams-Young, D.; Ding, F.; Lipparini, F.; Egidi, F.; Goings, J.; Peng, B.; Petrone, A.; Henderson, T.; Ranasinghe, D.; Zakrzewski, V. G.; Gao, J.; Rega, N.; Zheng, G.; Liang, W.; Hada, M.; Ehara, M.; Toyota, K.; Fukuda, R.; Hasegawa, J.; Ishida, M.; Nakajima, T.; Honda, Y.; Kitao, O.; Nakai, H.; Vreven, T.; Throssell, K.; Montgomery, J. A., Jr.; Peralta, J. E.; Ogliaro, F.; Bearpark, M. J.; Heyd, J. J.; Brothers, E. N.; Kudin, K. N.; Staroverov, V. N.; Keith, T. A.; Kobayashi, R.; Normand, J.; Raghavachari, K.; Rendell, A. P.; Burant, J. C.; Iyengar, S. S.; Tomasi, J.; Cossi, M.; Millam, J. M.; Klene, M.; Adamo, C.; Cammi, R.; Ochterski, J. W.; Martin, R. L.; Morokuma, K.; Farkas, O.; Foresman, J. B.; Fox, D. J. Gaussian, Inc., Wallingford CT, 2016.

[5] (a) Bauernschmitt, R.; Ahlrichs, R. Stability Analysis for Solutions of the Closed Shell Kohn–Sham Equation. *J. Chem. Phys.* **1996**, 104, 9047–9052. (b) Schlegel, H. B.; McDouall, J. J. W. Do you have SCF stability and convergence problems? in *Computational Advances in Organic Chemistry: Molecular Structure and Reactivity* 167–185 (Springer Netherlands, **1991**). doi:10.1007/978-94-011-3262-6_2. (c) Seeger, R.; Pople, J. A. Self-consistent molecular orbital methods. XVIII. Constraints and stability in Hartree–Fock theory. *J. Chem. Phys.* **1977**, 66, 3045–3050.

[6] (a) Fukui, K. The Path of Chemical Reactions - The IRC Approach. *Acc. Chem. Res.* **1981**, 14, 363–368. (b) Maeda, S.; Harabuchi, Y.; Ono, Y.; Taketsugu, T.; Morokuma, K. Intrinsic Reaction Coordinate: Calculation, Bifurcation, andAutomated Search. *International Journal of Quantum Chemistry* **2015**, 115, 258–269.

[7] Schaftenaar, G.; Vlieg, E.; Vriend, G. Molden 2.0: quantum chemistry meets proteins. *J. Comput. Aided. Mol. Des.* **2017**, 31, 789–800.

[8] CYLview, 1.0b; Legault, C. Y., Université de Sherbrooke, **2009**, http://www.cylview.org

[9] Anderson, T.L.; Kwan, E.E. PyQuiver, **2020**, www.github.com/ekwan/PyQuiver

[10] Yanai, T.; Pew, D. T.; Handy, N. C. A New Hybrid Exchange–correlation Functional Using the Coulomb-attenuating Method (CAM-B3LYP). *Chem. Phys. Lett.* **2004**, 393, 51–57.

[11] Paul, C. E.; Gargiulo, S.; Opperman, D. J.; Lavandera, I.; Gotor-Fernandez, V.; Gotor, V.; Taglieber, A.; Arends, I. W. C. E.; Hollmann, F. Mimicking Nature: Synthetic Nicotinamide Cofactors for C═C Bioreduction Using Enoate Reductases. *Org. Lett.* **2013**, 15, 1, 180–183.

[12] Dittmer, D. C.; Lombardo, A.; Batzold, F. H.; Greene, C. S. Reactions of Perhaloacetones with Dihydropyridines and Other Electron Donors. *J. Org. Chem.* **1976**, 41, 2976–2981.

[13] Bertuzzi, G.; Sinisi, A.; Caruana, L.; Mazzanti, A.; Fochi, M.; Bernardi, L. Catalytic Enantioselective Addition of Indoles to Activated N-Benzylpyridinium Salts: Nucleophilic Dearomatization of Pyridines with Unusual C-4 Regioselectivity. *ACS Catal.* **2016**, 6, 6473−6477

[14] Song, X.; Yan, R.-J.; Du, W.; Chen, Y.-C. Asymmetric Dearomative Cascade Multiple Functionalizations of Activated *N*-Alkylpyridinium and *N*-Alkylquinolinium Salts. *Org. Lett.* **2020**, 22, 7617−7621.

[15] Nadeau, C.; Aly, S.; Belyk, K. Rhodium-Catalyzed Enantioselective Addition of Boronic Acids to *N*-Benzylnicotinate Salts. *J. Am. Chem. Soc.* **2011**, 133, 2878–2880.

[16] (a) Yan, X.; Ge, L.; Castiñeira Reis, M.; Harutyunyan, S. R. Nucleophilic Dearomatization of N-Heteroaromatics Enabled by Lewis Acids and Copper Catalysis. *J. Am. Chem. Soc.* **2020**, 142, 20247–20256. (b) Guo, Y.; Castiñeira Reis, M.; Kootstra, J.; Harutyunyan, S. R. Enantioselective Catalytic Dearomative Addition of Grignard Reagents to 4-Methoxypyridinium Ions. *ACS Catal.* **2021**, 11, 8476–8483.

[17] (a) Yan, X.; Ge, L.; Castiñeira Reis, M.; Harutyunyan, S. R. Nucleophilic Dearomatization of N-Heteroaromatics Enabled by Lewis Acids and Copper Catalysis. *J. Am. Chem. Soc.* **2020**, 142, 20247–20256. (b) Guo, Y.; Castiñeira Reis, M.; Kootstra, J.; Harutyunyan, S. R. Enantioselective Catalytic Dearomative Addition of Grignard Reagents to 4-Methoxypyridinium Ions. *ACS Catal.* **2021**, 11, 8476–8483.

[18] https://cccbdb.nist.gov/expbondlengths2x.asp?descript=rC=C&all=0 [02/02/2014]

[19] https://cccbdb.nist.gov/expbondlengths2x.asp?descript=rCN&all=0 [02/02/2014]
